# New loci for body fat percentage reveal link between adiposity and cardiometabolic disease risk

**DOI:** 10.1038/ncomms10495

**Published:** 2016-02-01

**Authors:** Yingchang Lu, Felix R. Day, Stefan Gustafsson, Martin L. Buchkovich, Jianbo Na, Veronique Bataille, Diana L. Cousminer, Zari Dastani, Alexander W. Drong, Tõnu Esko, David M. Evans, Mario Falchi, Mary F. Feitosa, Teresa Ferreira, Åsa K. Hedman, Robin Haring, Pirro G. Hysi, Mark M. Iles, Anne E. Justice, Stavroula Kanoni, Vasiliki Lagou, Rui Li, Xin Li, Adam Locke, Chen Lu, Reedik Mägi, John R. B. Perry, Tune H. Pers, Qibin Qi, Marianna Sanna, Ellen M. Schmidt, William R. Scott, Dmitry Shungin, Alexander Teumer, Anna A. E. Vinkhuyzen, Ryan W. Walker, Harm-Jan Westra, Mingfeng Zhang, Weihua Zhang, Jing Hua Zhao, Zhihong Zhu, Uzma Afzal, Tarunveer Singh Ahluwalia, Stephan J. L. Bakker, Claire Bellis, Amélie Bonnefond, Katja Borodulin, Aron S. Buchman, Tommy Cederholm, Audrey C. Choh, Hyung Jin Choi, Joanne E. Curran, Lisette C. P. G. M. de Groot, Philip L. De Jager, Rosalie A. M. Dhonukshe-Rutten, Anke W. Enneman, Elodie Eury, Daniel S. Evans, Tom Forsen, Nele Friedrich, Frédéric Fumeron, Melissa E. Garcia, Simone Gärtner, Bok-Ghee Han, Aki S. Havulinna, Caroline Hayward, Dena Hernandez, Hans Hillege, Till Ittermann, Jack W. Kent, Ivana Kolcic, Tiina Laatikainen, Jari Lahti, Irene Mateo Leach, Christine G. Lee, Jong-Young Lee, Tian Liu, Youfang Liu, Stéphane Lobbens, Marie Loh, Leo-Pekka Lyytikäinen, Carolina Medina-Gomez, Karl Michaëlsson, Mike A. Nalls, Carrie M. Nielson, Laticia Oozageer, Laura Pascoe, Lavinia Paternoster, Ozren Polašek, Samuli Ripatti, Mark A. Sarzynski, Chan Soo Shin, Nina Smolej Narančić, Dominik Spira, Priya Srikanth, Elisabeth Steinhagen-Thiessen, Yun Ju Sung, Karin M. A. Swart, Leena Taittonen, Toshiko Tanaka, Emmi Tikkanen, Nathalie van der Velde, Natasja M. van Schoor, Niek Verweij, Alan F. Wright, Lei Yu, Joseph M. Zmuda, Niina Eklund, Terrence Forrester, Niels Grarup, Anne U. Jackson, Kati Kristiansson, Teemu Kuulasmaa, Johanna Kuusisto, Peter Lichtner, Jian'an Luan, Anubha Mahajan, Satu Männistö, Cameron D. Palmer, Janina S. Ried, Robert A. Scott, Alena Stancáková, Peter J. Wagner, Ayse Demirkan, Angela Döring, Vilmundur Gudnason, Douglas P. Kiel, Brigitte Kühnel, Massimo Mangino, Barbara Mcknight, Cristina Menni, Jeffrey R. O'Connell, Ben A. Oostra, Alan R. Shuldiner, Kijoung Song, Liesbeth Vandenput, Cornelia M. van Duijn, Peter Vollenweider, Charles C. White, Michael Boehnke, Yvonne Boettcher, Richard S. Cooper, Nita G. Forouhi, Christian Gieger, Harald Grallert, Aroon Hingorani, Torben Jørgensen, Pekka Jousilahti, Mika Kivimaki, Meena Kumari, Markku Laakso, Claudia Langenberg, Allan Linneberg, Amy Luke, Colin A. Mckenzie, Aarno Palotie, Oluf Pedersen, Annette Peters, Konstantin Strauch, Bamidele O. Tayo, Nicholas J. Wareham, David A. Bennett, Lars Bertram, John Blangero, Matthias Blüher, Claude Bouchard, Harry Campbell, Nam H. Cho, Steven R. Cummings, Stefan A. Czerwinski, Ilja Demuth, Rahel Eckardt, Johan G. Eriksson, Luigi Ferrucci, Oscar H. Franco, Philippe Froguel, Ron T. Gansevoort, Torben Hansen, Tamara B. Harris, Nicholas Hastie, Markku Heliövaara, Albert Hofman, Joanne M. Jordan, Antti Jula, Mika Kähönen, Eero Kajantie, Paul B. Knekt, Seppo Koskinen, Peter Kovacs, Terho Lehtimäki, Lars Lind, Yongmei Liu, Eric S. Orwoll, Clive Osmond, Markus Perola, Louis Pérusse, Olli T. Raitakari, Tuomo Rankinen, D. C. Rao, Treva K. Rice, Fernando Rivadeneira, Igor Rudan, Veikko Salomaa, Thorkild I. A. Sørensen, Michael Stumvoll, Anke Tönjes, Bradford Towne, Gregory J. Tranah, Angelo Tremblay, André G. Uitterlinden, Pim van der Harst, Erkki Vartiainen, Jorma S. Viikari, Veronique Vitart, Marie-Claude Vohl, Henry Völzke, Mark Walker, Henri Wallaschofski, Sarah Wild, James F. Wilson, Loïc Yengo, D. Timothy Bishop, Ingrid B. Borecki, John C. Chambers, L. Adrienne Cupples, Abbas Dehghan, Panos Deloukas, Ghazaleh Fatemifar, Caroline Fox, Terrence S. Furey, Lude Franke, Jiali Han, David J. Hunter, Juha Karjalainen, Fredrik Karpe, Robert C. Kaplan, Jaspal S. Kooner, Mark I. McCarthy, Joanne M. Murabito, Andrew P. Morris, Julia A. N. Bishop, Kari E. North, Claes Ohlsson, Ken K. Ong, Inga Prokopenko, J. Brent Richards, Eric E. Schadt, Tim D. Spector, Elisabeth Widén, Cristen J. Willer, Jian Yang, Erik Ingelsson, Karen L. Mohlke, Joel N. Hirschhorn, John Andrew Pospisilik, M. Carola Zillikens, Cecilia Lindgren, Tuomas Oskari Kilpeläinen, Ruth J. F. Loos

**Affiliations:** 1 The Charles Bronfman Institute for Personalized Medicine, The Icahn School of Medicine at Mount Sinai, New York, New York 10029, USA; 2 The Department of Preventive Medicine, The Icahn School of Medicine at Mount Sinai, New York, New York 10029, USA; 3 MRC Epidemiology Unit, University of Cambridge School of Clinical Medicine, Institute of Metabolic Science, University of Cambridge, Cambridge Biomedical Campus, Cambridge CB2 0QQ, UK; 4 Science for Life Laboratory, Uppsala University, 750 85 Uppsala, Sweden; 5 Department of Medical Sciences, Molecular Epidemiology, Uppsala University, 751 85 Uppsala, Sweden; 6 Department of Genetics, University of North Carolina, Chapel Hill, North Carolina 27599, USA; 7 Department of Developmental and Regenerative Biology, The Icahn School of Medicine at Mount Sinai, New York, New York 10029, USA; 8 West Herts NHS Trust, Herts HP2 4AD, UK; 9 Department of Twin Research and Genetic Epidemiology, King's College London, London SE1 7EH, UK; 10 Institute for Molecular Medicine Finland, University of Helsinki, FI-00290 Helsinki, Finland; 11 Department Epidemiology, Biostatistics and Human Genetics, Lady Davis Institute, Jewish General Hospital, McGill University, Montréal, Quebec, Canada H3T1E2; 12 Wellcome Trust Centre for Human Genetics, University of Oxford, Oxford OX3 7BN, UK; 13 Estonian Genome Center, Univeristy of Tartu, Tartu, 51010, Estonia; 14 Broad Institute of the Massachusetts Institute of Technology and Harvard University, Cambridge 2142, USA; 15 Divisions of Endocrinology and Genetics and Center for Basic and Translational Obesity Research, Boston Children's Hospital, Boston, Massachusetts 02115, USA; 16 Department of Genetics, Harvard Medical School, Boston, Massachusetts 02115, USA; 17 University of Queensland Diamantina Institute, Translational Research Institute, Brisbane, Queensland 4102, Australia; 18 MRC Integrative Epidemiology Unit, School of Social and Community Medicine, University of Bristol, Bristol BS82BN, UKnited; 19 Department of Genomics of Common Disease, School of Public Health, Imperial College London, London W12 0NN, UK; 20 Division of Statistical Genomics, Department of Genetics, Washington University School of Medicine, St Louis, Missouri 63108, USA; 21 Institute of Clinical Chemistry and Laboratory Medicine, University Medicine Greifswald, 17475 Greifswald, Germany; 22 European University of Applied Sciences, Faculty of Applied Public Health, 18055 Rostock, Germany; 23 Leeds Institute of Cancer and Pathology, Cancer Research UK Leeds Centre, University of Leeds, Leeds LS9 7TF, UK; 24 Department of Epidemiology, University of North Carolina at Chapel Hill, Chapel Hill, North Carolina 27599, USA; 25 William Harvey Research Institute, Barts and The London School of Medicine and Dentistry, Queen Mary University of London, London EC1M 6BQ, UK; 26 Wellcome Trust Sanger Institute, Human Genetics, Hinxton, Cambridge CB10 1SA, UK; 27 Oxford Centre for Diabetes, Endocrinology and Metabolism, University of Oxford, Churchill Hospital, Oxford OX3 7LJ, UK; 28 Department of Epidemiology, Harvard School of Public Health, Boston, Massachusetts 02115, USA; 29 Center for Statistical Genetics, Department of Biostatistics, University of Michigan, Ann Arbor, Michigan 48109, USA; 30 Department of Biostatistics, Boston University School of Public Health, Boston, Massachusetts 02118, USA; 31 Novo Nordisk Foundation Center for Basic Metabolic Research, Section of Metabolic Genetics, Faculty of Health and Medical Sciences, University of Copenhagen, 2100 Copenhagen, Denmark; 32 Medical and Population Genetics Program, Broad Institute of MIT and Harvard, Cambridge 02142, USA; 33 Department of Epidemiology Research, Statens Serum Institut, 2100 Copenhagen, Denmark; 34 Department of Epidemiology and Popualtion Health, Albert Einstein College of Medicine, Bronx, New York 10461, USA; 35 Department of Computational Medicine and Bioinformatics, University of Michigan, Ann Arbor, Michigan 48109, USA; 36 Department of Epidemiology and Biostatistics, Imperial College London, London W2 1PG, UK; 37 Ealing Hospital NHS Trust, Middlesex UB1 3HW, UK; 38 Lund University Diabetes Centre, Department of Clinical Science, Genetic and Molecular Epidemiology Unit, Skåne University Hosptial, 205 02 Malmö, Sweden; 39 Department of Public Health and Clinical Medicine, Unit of Medicine, Umeå University, 901 87 Umeå, Sweden; 40 Department of Odontology, Umeå University, 901 85 Umeå, Sweden; 41 Institute for Community Medicine, University Medicine Greifswald, 17475 Greifswald, Germany; 42 Interfaculty Institute for Genetics and Functional Genomics, University Medicine Greifswald, 17475 Greifswald, Germany; 43 Queensland Brain Institute, The University of Queensland, Brisbane 4072, Australia; 44 Program in Medical and Population Genetics, Broad Institute of Harvard and Massachusetts Institute of Technology, Cambridge, Massachusetts 02142, USA; 45 Divisions of Genetics and Rheumatology, Department of Medicine, Brigham and Women's Hospital and Harvard Medical School, Boston, Massachusetts 02446, USA; 46 Partners Center for Personalized Genetic Medicine, Boston, Massachusetts 02446, USA; 47 Department of Dermatology, Brigham and Women's Hospital, Boston, Massachusetts 02115, USA; 48 Copenhagen Prospective Studies on Asthma in Childhood, Faculty of Health and Medical Sceinces, University of Copenhagen, 2200 Copenhagen, Denmark; 49 Danish Pediatric Asthma Center, Gentofte Hospital, The Capital Region, 2200 Copenhagen, Denmark; 50 Steno Diabetes Center A/S, DK-2820 Gentofte, Denmark; 51 University of Groningen, University Medical Center Groningen, Department of Medicine, 9700 RB Groningen, The Netherlands; 52 Department of Genetics, Texas Biomedical Research Institute, San Antonio, Texas 78245, USA; 53 CNRS UMR 8199, F-59019 Lille, France; 54 European Genomic Institute for Diabetes, 59000 Lille, France; 55 Université de Lille 2, 59000 Lille, France; 56 National Institute for Health and Welfare, FI-00271 Helsinki, Finland; 57 Rush Alzheimer's Disease Center, Rush University Medical Center, Chicago, Illinois 60612, USA; 58 Department of Public Health and Caring Sciences, Clinical Nutrition and Metabolism, Uppsala University, 751 85 Uppsala, Sweden; 59 Lifespan Health Research Center, Wright State University Boonshoft School of Medicine, Dayton, Ohio 45420, USA; 60 Department of Anatomy, Seoul National University College of Medicine, Seoul 03080, Korea; 61 South Texas Diabetes and Obesity Institute, University of Texas Rio Grande Valley, Brownsville, Texas 78520; 62 Department of Human Nutrition, Wageningen University, 6700 EV Wageningen, The Netherlands; 63 Harvard Medical School, Boston, Massachusetts 02115, USA; 64 Program in Translational NeuroPsychiatric Genomics, Department of Neurology, Brigham and Women's Hospital, Boston, Massachusetts 02115, USA; 65 Department of Internal Medicine, Erasmus Medical Center, 3015GE Rotterdam, The Netherlands; 66 California Pacific Medical Center Research Institute, San Francisco, California 94107, USA; 67 Department of General Practice and Primary Health Care, University of Helsinki, FI-00014 Helsinki, Finland; 68 INSERM, UMR_S 1138, Centre de Recherche des Cordeliers, F-75006 Paris, France; 69 Sorbonne Universités, UPMC Univ Paris 06, UMR_S 1138, Centre de Recherche des Cordeliers, F-75006 Paris, France; 70 Université Paris Descartes, Sorbonne Paris Cité, UMR_S 1138, Centre de Recherche des Cordeliers, F-75006 Paris, France; 71 Univ Paris Diderot, Sorbonne Paris Cité, UMR_S 1138, Centre de Recherche des Cordeliers, F-75006 Paris, France; 72 Laboratory of Epidemiology and Population Sciences, National Institute on Aging, Bethesda, Maryland 20892, USA; 73 Department of Medicine A, University Medicine Greifswald, 17475 Greifswald, Germany; 74 Center for Genome Science, National Institute of Health, Osong Health Technology Administration Complex, Chungcheongbuk-do 370914, Korea; 75 MRC Human Genetics Unit, Institute of Genetics and Molecular Medicine, University of Edinburgh, Edinburgh EH4 2XU, UK; 76 Laboratory of Neurogenetics, National Institute on Aging, National Institutes of Health, Bethesda, Maryland 20892, USA; 77 University of Groningen, University Medical Center Groningen, Department of Cardiology, 9700 RB Groningen, The Netherlands; 78 Department of Public Health, Faculty of Medicine, University of Split, Split 21000, Croatia; 79 Hospital District of North Karelia, FI-80210 Joensuu, Finland; 80 Institute of Public Health and Clinical Nutrition, University of Eastern Finland, FI-70211 Kuopio, Finland; 81 Folkhälsan Research Centre, FI-00290 Helsinki, Finland; 82 Institute of Behavioural Sciences, University of Helsinki, FI-00014 Helsinki, Finland; 83 Department of Medicine, Oregon Health and Science University, Portland, Oregon 97239, USA; 84 Research Service, Veterans Affairs Medical Center, Portland, Oregon 97239, USA; 85 Max Planck Institute for Molecular Genetics, Department of Vertebrate Genomics, 14195 Berlin, Germany; 86 Max Planck Institute for Human Development, 14194 Berlin, Germany; 87 Thurston Arthritis Research Center, University of North Carolina at Chapel Hill, Chaper Hill, North Carolina 27599-7280, USA; 88 Translational Laboratory in Genetic Medicine (TLGM), Agency for Science, Technology and Research (A*STAR), 8A Biomedical Grove, Immunos, Level 5, Singapore 138648, Singapore; 89 Department of Clinical Chemistry, University of Tampere School of Medicine, FI-33014 Tampere, Finland; 90 Department of Clinical Chemistry, Fimlab Laboratories and School of Medicine, University of Tampere, FI-33520 Tampere, Finland; 91 Netherlands Genomics Initiative (NGI)-sponsored Netherlands Consortium for Healthy Aging (NCHA), Rotterdam The Netherlands; 92 Department of Epidemiology, Erasmus Medical Center, 3015GE Rotterdam, The Netherlands; 93 Department of Surgical Sciences, Orthopedics, Uppsala University, 751 85 Uppsala, Sweden; 94 School of Public Health, Oregon Health & Science University, Portland, Oregon 97239, USA; 95 Bone & Mineral Unit, Oregon Health & Science University, Portland, Oregon 97239, USA; 96 Institute of Cell & Molecular Biosciences, Newcastle University, Newcastle NE1 7RU, UK; 97 Centre for Global Health Research, Usher Institute of Population Health Sciences and Informatics, University of Edinburgh, Teviot Place, Edinburgh EH8 9AG, UK; 98 Hjelt Institute, University of Helsinki, FI-00014 Helsinki, Finland; 99 Human Genomics Laboratory, Pennington Biomedical Research Center, Baton Rouge, Los Angeles 70808, USA; 100 Department of Internal Medicine, Seoul National University College of Medicine, Seoul 03080, Korea; 101 Institute for Anthropological Research, Zagreb 10000, Croatia; 102 The Berlin Aging Study II; Research Group on Geriatrics; Charité—Universitätsmedizin Berlin, 13347 Berlin, Germany; 103 Lipid Clinic at the Interdisciplinary Metabolism Center, Charité-Universitätsmedizin Berlin, 13353 Berlin, Germany; 104 Division of Biostatistics, Washington University School of Medicine, St Louis, Missouri 63110, USA; 105 EMGO Institute for Health and Care Research, VU University Medical Center, 1081 BT Amsterdam, The Netherlands; 106 VUMC, Department of Epidemiology and Biostatistics, 1081 BT Amsterdam, The Netherlands; 107 Department of Pediatrics, University of Oulu, FI-90014 Oulu, Finland; 108 Department of Pediatrics, Vaasa Central Hospital, FI-65100 Vaasa, Finland; 109 Translational Gerontology Branch, National Institute on Aging, Baltimore, Maryland 21225, USA; 110 Department of Epidemiology; University of Pittsburgh, Pittsburgh, Pennsylvania 15261, USA; 111 Tropical Metabolism Research Unit, Tropical Medicine Research Institute, University of the West Indies, Mona JMAAW15, Jamaica; 112 Faculty of Health Sciences, Institute of Clinical Medicine, Internal Medicine, University of Eastern Finland, 70210 Kuopio, Finland; 113 Department of Medicine, University of Eastern Finland, 70210 Kuopio, Finland; 114 Kuopio University Hospital, 70029 Kuopio, Finland; 115 Institute of Human Genetics, Helmholtz Zentrum München—German Research Center for Environmental Health, 85764 Neuherberg, Germany; 116 Institute of Genetic Epidemiology, Helmholtz Zentrum München—German Research Center for Environmental Health, 85764 Neuherberg, Germany; 117 Department of Medicine, University of Eastern Finland and Kuopio University Hospital, 70210 Kuopio, Finland; 118 Genetic Epidemiology Unit, Department of Epidemiology, Erasmus University Medical Center, 3015GE Rotterdam, The Netherlands; 119 Institute of Epidemiology I, Helmholtz Zentrum München—German Research Center for Environmental Health, 85764 Neuherberg, Germany; 120 Institute of Epidemiology II, Helmholtz Zentrum München—German Research Center for Environmental Health, 85764 Neuherberg, Germany; 121 Icelandic Heart Association, Kopavogur 201, Iceland; 122 University of Iceland, Faculty of Medicine, Reykjavik 101, Iceland; 123 Department of Medicine Beth Israel Deaconess Medical Center and Harvard Medical School, Boston, Massachusetts 02115; 124 Institute for Aging Research Hebrew Senior Life, Boston, Massachusetts 02131, USA; 125 Research Unit of Molecular Epidemiology, Helmholtz Zentrum München—German Research Center for Environmental Health, 85764 Neuherberg, Germany; 126 Cardiovascular Health Research Unit, University of Washington, Seattle, Washington 98101, USA; 127 Program in Biostatistics and Biomathematics, Divison of Public Health Sciences, Fred Hutchinson Cancer Research Center, Seattle, Washington 98109, USA; 128 Department of Biostatistics, University of Washington, Seattle, Washington 98195, USA; 129 Program for Personalized and Genomic Medicine, Division of Endocrinology, Diabetes and Nutrition, Department of Medicine, University of Maryland School of Medicine, Baltimore, Maryland 21201, USA; 130 Geriatric Research and Education Clinical Center, Vetrans Administration Medical Center, Baltimore, Maryland 21042, USA; 131 Genetics, Projects Clinical Platforms and Sciences, GlaxoSmithKline, Philadelphia, Pennsylvania 19112, USA; 132 Centre for Bone and Arthritis Research, Department of Internal Medicine and Clinical Nutrition, Institute of Medicine, Sahlgrenska Academy, University of Gothenburg, 413 45 Gothenburg, Sweden; 133 Center for Medical Systems Biology, 2300 Leiden, The Netherlands; 134 Department of Internal Medicine, University Hospital Lausanne (CHUV) and University of Lausanne, 1011 Lausanne, Switzerland; 135 University of Leipzig, IFB Adiposity Diseases, 04103 Leipzig, Germany; 136 University of Leipzig, Department of Medicine, 04103 Leipzig, Germany; 137 Department of Public Health Sciences, Stritch School of Medicine, Loyola University Chicago, Maywood, Illinois 61053, USA; 138 German Center for Diabetes Research (DZD), 85764 Neuherberg, Germany; 139 Institute of Cardiovascular Science, University College London, London WC1E 6BT, UK; 140 Department of Clinical Medicine, Faculty of Health and Medical Sciences, University of Copenhagen, 2200 Copenhagen, Denmark; 141 Faculty of Medicine, University of Aalborg, 9220 Aalborg, Denmark; 142 Research Centre for Prevention and Health, DK2600 Capital Region of Denmark, Denmark; 143 Department of Epidemiology and Public Health, UCL, London WC1E 6BT, UK; 144 Research Centre for Prevention and Health, Glostrup Hospital, 2600 Glostrup, Denmark; 145 Massachusetts General Hospital, Center for Human Genetic Research, Psychiatric and Neurodevelopmental Genetics Unit, Boston, Massachusetts 02114, USA; 146 Institute of Medical Informatics, Biometry and Epidemiology, Chair of Genetic Epidemiology, Ludwig-Maximilians-Universität, 81377 Munich, Germany; 147 School of Public Health, Faculty of Medicine, Imperial College London, London W6 8RP, UK; 148 Lübeck Interdisciplinary Platform for Genome Analytics, Institutes of Neurogenetics and Integrative and Experimental Genomics, University of Lübeck, 23562 Lübeck, Germany; 149 Ajou University School of Medicine, Department of Preventive Medicine, Suwon Kyoung-gi 443-721, Korea; 150 Institute of Medical and Human Genetics, Charité—Universitätsmedizin Berlin, 13353 Berlin, Germany; 151 Faculty of Health Sciences, University of Southern Denmark, 5000 Odense, Denmark; 152 Department of Clinical Physiology, Tampere University Hospital, FI-33521 Tampere, Finland; 153 Department of Clinical Physiology, University of Tampere School of Medicine, FI-33014 Tampere, Finland; 154 Children's Hospital, Helsinki University Hospital and University of Helsinki, FI-00029 Helsinki, Finland; 155 Department of Obstetrics and Gynecology, MRC Oulu, Oulu University Hospital and University of Oulu, FI-90029 Oulu, Finland; 156 Department of Medical Sciences, Uppsala University, 751 85 Uppsala, Sweden; 157 Center for Human Genetics, Division of Public Health Sciences, Wake Forest School of Medicine, Winston-Salem, North Carolina 27157, USA; 158 MRC Lifecourse Epidemiology Unit, University of Southampton, Southampton General Hospital, Southampton SO16 6YD, UK; 159 Department of Kinesiology, Laval University, Québec City, Quebec, Canada G1V 0A6; 160 Institute of Nutrition and Functional Foods, Laval University, Québec City, Quebec, Canada G1V 0A6; 161 Department of Clinical Physiology and Nuclear Medicine, Turku University Hospital, FI-20521 Turku, Finland; 162 Research Centre of Applied and Preventive Cardiovascular Medicine, University of Turku, FI-20520 Turku, Finland; 163 Department of Psychiatry, Washington University School of Medicine, St Louis, Missouri 63110, USA; 164 Institute of Preventive Medicine, Bispebjerg and Frederiksberg Hospital, The Capital Region, 2000 Frederiksberg, Denmark; 165 Durrer Center for Cardiogenetic Research, Interuniversity Cardiology Institute Netherlands-Netherlands Heart Institute, 3501 DG Utrecht, The Netherlands; 166 Department of Genetics, University Medical Center Groningen, University of Groningen, 9700 RB Groningen, The Netherlands; 167 Department of Medicine, University of Turku, FI-20521 Turku, Finland; 168 School of Nutrition, Laval University, Québec City, Quebec, Canada G1V 0A6; 169 DZHK (German Centre for Cardiovascular Research), partner site Greifswald, 17475 Greifswald, Germany; 170 DZD (German Centre for Diabetes Research), partner site Greifswald, 17475 Greifswald, Germany; 171 Institute of Cellular Medicine, Newcastle University, Newcastle NE2 4HH, UK; 172 Centre for Population Health Sciences, Usher Institute of Population Health Sciences and Informatics, University of Edinburgh, Edinburgh EH8 9AG, UK; 173 Analytical Genetics Group, Regeneron Genetics Center, Regeneron Pharmaceuticals, Inc., Tarrytown, New York 10591, USA; 174 Imperial College Healthcare NHS Trust, London W12 0HS, UK; 175 National Heart, Lung, and Blood Institute, the Framingham Heart Study, Framingham, Massachusetts 01702, USA; 176 Department of Epidemiology, Erasmus Medical Center, 3000CA Rotterdam/Zuidholland, The Netherlands; 177 Princess Al-Jawhara Al-Brahim Centre of Excellence in Research of Hereditary Disorders (PACER-HD), King Abdulaziz University, Jeddah 21589, Saudi Arabia; 178 Department of Biology, University of North Carolina, Chapel Hill, North Carolina 27599, USA; 179 Department of Epidemiology, Richard M. Fairbanks School of Public Health, Melvin and Bren Simon Cancer Center, Indianapolis, Indiana 46202, USA; 180 Channing Division of Network Medicine, Department of Medicine, Brigham and Women's Hospital and Harvard Medical School, Boston, Massachusetts 02115, USA; 181 Department of Nutrition, Harvard School of Public Health, Boston, Massachusetts 02115, USA; 182 Oxford NIHR Biomedical Research Centre, Oxford OX3 7LJ, UK; 183 National Heart and Lung Institute, Imperial College London, London W12 0NN, UK; 184 Boston University School of Medicine, Department of Medicine, Section of General Internal Medicine, Boston, Massachusetts 02118, USA; 185 NHLBI's and Boston University's Framingham Heart Study, Framingham, Massachusetts 01702, USA; 186 Department of Biostatistics, University of Liverpool, Liverpool L69 3GA, UK; 187 Carolina Center for Genome Sciences and Department of Epidemiology, University of North Carolina at Chapel Hill, Chapel Hill, North Carolina 27599-7400, USA; 188 MRC Unit for Lifelong Health and Ageing at UCL, London WC1B 5JU, UK; 189 Department of Paediatrics, University of Cambridge, Cambridge CB2 0QQ, UK; 190 Department of Medicine, Lady Davis Institute, Jewish General Hospital, McGill University, Montréal, Quebec, Canada H3T1E2; 191 Department of Twin Research, King's College London, London SE1 1E7, UK; 192 Division of Endocrinology, Lady Davis Institute, Jewish General Hospital, McGill University, Montréal, Quebec, Canada H3T1E2; 193 Icahn Institute for Genomics and Multiscale Biology, Icahn School of Medicine at Mount Sinai, New York, New York 10029, USA; 194 Department of Genetics and Genomic Sciences, Icahn School of Medicine at Mount Sinai, New York, New York 10029, USA; 195 Department of Human Genetics, University of Michigan, Ann Arbor, Michigan 48109, USA; 196 Department of Internal Medicine, Division of Cardiovascular Medicine, University of Michigan, Ann Arbor, Michigan 48109, USA; 197 Department of Medicine, Division of Cardiovascular Medicine, Stanford University School of Medicine, Stanford, California 94305, USA; 198 Department of Epigenetics, Max Planck Institute of Immunobiology and Epigenetics, D-76108 Freiburg, Germany; 199 The Big Data Institute, University of Oxford, Oxford OX3 7LJ, UK; 200 The Genetics of Obesity and Related Metabolic Traits Program, The Icahn School of Medicine at Mount Sinai, New York, New York, 10029, USA; 201 The Mindich Child Health and Development Institute, The Icahn School of Medicine at Mount Sinai, New York, New York 10029, USA

## Abstract

To increase our understanding of the genetic basis of adiposity and its links to cardiometabolic disease risk, we conducted a genome-wide association meta-analysis of body fat percentage (BF%) in up to 100,716 individuals. Twelve loci reached genome-wide significance (*P*<5 × 10^−8^), of which eight were previously associated with increased overall adiposity (BMI, BF%) and four (in or near *COBLL1/GRB14*, *IGF2BP1*, *PLA2G6*, *CRTC1*) were novel associations with BF%. Seven loci showed a larger effect on BF% than on BMI, suggestive of a primary association with adiposity, while five loci showed larger effects on BMI than on BF%, suggesting association with both fat and lean mass. In particular, the loci more strongly associated with BF% showed distinct cross-phenotype association signatures with a range of cardiometabolic traits revealing new insights in the link between adiposity and disease risk.

## Abstract (web summary)

A genome-wide association meta-analysis study here shows novel genetic loci to be associated to body fat percentage, and describes cross-phenotype association that further demonstrate a close relationship between adiposity and cardiovascular disease risk.

## Graphical abstract

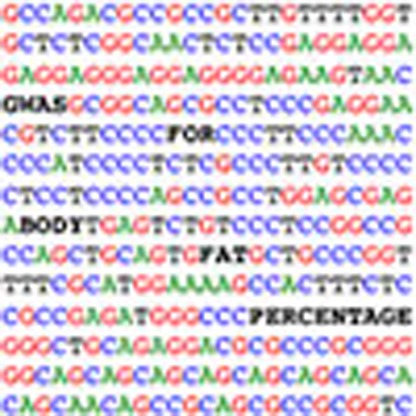

Large-scale meta-analyses of genome-wide association studies (GWAS) for adiposity traits and obesity risk have identified at least 160 loci that contribute to body weight and fat distribution in adults and children of diverse ancestry^1^,^2^,^3^,^4^,^5^,^6^,^7^,^8^,^9^,^10^,^11^,^12^,^13^,^14^,^15^,^16^,^17^,^18^,^19^,^20^. Studies of overall adiposity, assessed by body mass index (BMI), have mainly implicated genes that provide support for a role of the central nervous system (CNS) in obesity susceptibility^1^,^2^,^3^,^4^,^5^,^6^,^10^,^19^, whereas genetic loci associated with body fat distribution, assessed by waist-to-hip ratio (WHR), seem enriched for genes involved in adipocyte metabolism^9^,^11^,^20^. Although these commonly studied adiposity traits are easily collected in large populations and thus allow statistically well-powered meta-analyses, they represent heterogeneous phenotypes, for example, people with the same BMI or WHR may vary in BF%, translating in differences in cardiometabolic risk^21^,^22^.

To assess the genetic contribution to adiposity, we previously performed the first GWAS for BF% in nearly 40,000 individuals and identified two new loci (near *IRS1* and *SPRY2*), not identified in earlier large-scale GWAS for BMI^13^. Follow-up analyses of these loci provided strong evidence for *IRS1* to be involved in tissue-specific body fat storage and subsequent effects on cardiometabolic disease, such as type 2 diabetes (T2D) and coronary artery disease (CAD)^13^. While little is known about *SPRY2*, the Spry1 homolog in mice has been implicated in adipose tissue differentiation^23^. Taken together, these loci for BF% pointed towards new mechanisms involved in adipocyte metabolism that differ from the BMI-associated loci that suggested a role for the CNS^13^,^19^.

Here, we have extended our study to include more than 100,000 individuals and continue to discover novel genetic loci associated with BF% that have not been identified before for any of the commonly studied adiposity traits^1^,^2^,^3^,^4^,^5^,^6^,^7^,^8^,^9^,^10^,^11^,^12^,^13^,^14^,^15^,^16^,^17^,^18^,^19^,^20^. Through an in-depth integrative characterization, including cross-trait association analyses, expression quantitative trait loci (eQTL), pathway and network analyses, regulome analyses and transgenic drosophila models, we show that these loci provide new insights into the biology that underlies adiposity and related cardiometabolic health, by specifically highlighting peripheral physiological mechanisms.

## Results

### Analyses in >100,000 individuals identify 12 loci for BF%

In our primary meta-analysis, we combined results of genetic associations with BF% for up to 100,716 individuals from 43 GWAS (*n* up to 76,137) and 13 MetaboChip studies (*n* up to 24,582), predominantly of European ancestry (*n* up to 89,297), but also of non-European ancestry (*n* up to 11,419) populations (Supplementary Table 1 and Supplementary Fig. 1). As women have on average a higher BF% than men, we also stratified meta-analyses by sex (*n*_men_ up to 52,416; *n*_women_ up to 48,956). In secondary meta-analyses, we combined data from European-ancestry populations only (*n* up to 89,297; *n*_men_ up to 44,429; *n*_women_ up to 45,525) to reduce genotypic and phenotypic heterogeneity that may have been introduced in the overall analyses by combining diverse ancestries.

In our primary meta-analysis of men and women combined, single-nucleotide polymorphisms (SNPs) in 10 independent loci reached genome-wide significance (GWS, *P*<5 × 10^−8^; Table 1 and Supplementary Fig. 2), including the three loci that we identified before^13^. Two additional loci, near *PLA2G6* and in *CRTC1*, were identified in men-specific and women-specific analyses, respectively (Table 1 and Supplementary Fig. 3). The European-ancestry-only analyses revealed the same loci, but no additional ones (Supplementary Tables 4–6, Supplementary Figs 4 and 5). We did not identify evidence of secondary signals at any of the 12 loci.

**Figure 1:**
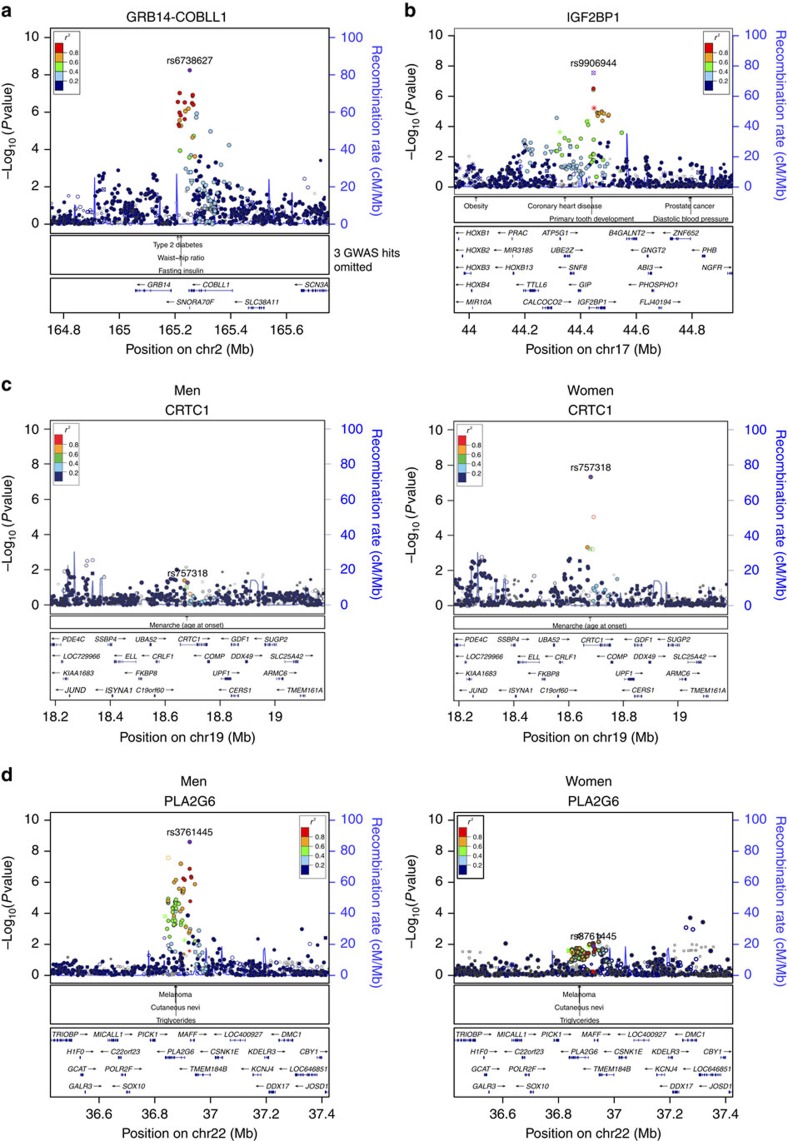
Regional plots of the four newly identified loci that reached genome-wide significant association with body fat percentage. Regional plots of the four newly identified loci that reached genome-wide significant association with body fat percentage in all-ancestry analyses, in men and women combined for the *COBLL1/GRB14* and *IGF2BP1* loci (**a**,**b**), and separately for the *CRTC1* and *PLA2G6* (**c**,**d**). Each symbol represents the significance (*P* value on a −log10 scale) of a SNP with BF% as a function of the SNP's genomic position (NCBI Build 36). For each locus, the index SNP is represented in the purple colour. The colour of all other SNPs indicates LD with the index SNP (estimated by CEU *r*^2^ from the HapMap Project data Phase II CEU). Recombination rates are also estimated from International HapMap Project data, and gene annotations are obtained from the UCSC Genome Browser. GWAS catalogues SNPs with *P* value <5 × 10^−8^ are shown in the middle panel. Different shapes denote the different categories of the SNPs: up-triangle for framestop or splice SNPs, down-triangle for nonsynonymous SNPs, square for coding or untranslated region (UTR) SNPs; star for SNPs in tfbscons region, square filled with ‘X' symbol for SNPs located in mcs44placental region and circle for SNPs with no annotation information.

Two (near *IRS1* and *SPRY2*) of the 12 loci had been first identified in our previous genome-wide screen for BF% (ref. 13), and six loci (in/near *FTO*, *MC4R*, *TMEM18*, *TOMM40/APOE*, *TUFM*/*SH2B1* and *SEC16B*) had been first reported for association with BMI^1^,^2^,^3^,^4^,^5^,^6^,^10^. Four of the 12 loci, in or near *COBLL1/GRB14*, *IGF2BP1*, *PLA2G6* and *CRTC1*, have not been associated with an overall adiposity trait (such as BMI, BF%, obesity risk) before (Fig. 1 and Supplementary Fig. 6). Of note, the *COBLL1/GRB14* locus was previously established as a locus for body fat distribution independent of overall adiposity, assessed by WHR_adjBMI_^11^, and the *CRTC1* locus has been first reported for its association with age at menarche^24^ (Table 2, Supplementary Table 7, See also ‘Cross-phenotype association' section).

### Effect sizes and explained variance

**Figure 2:**
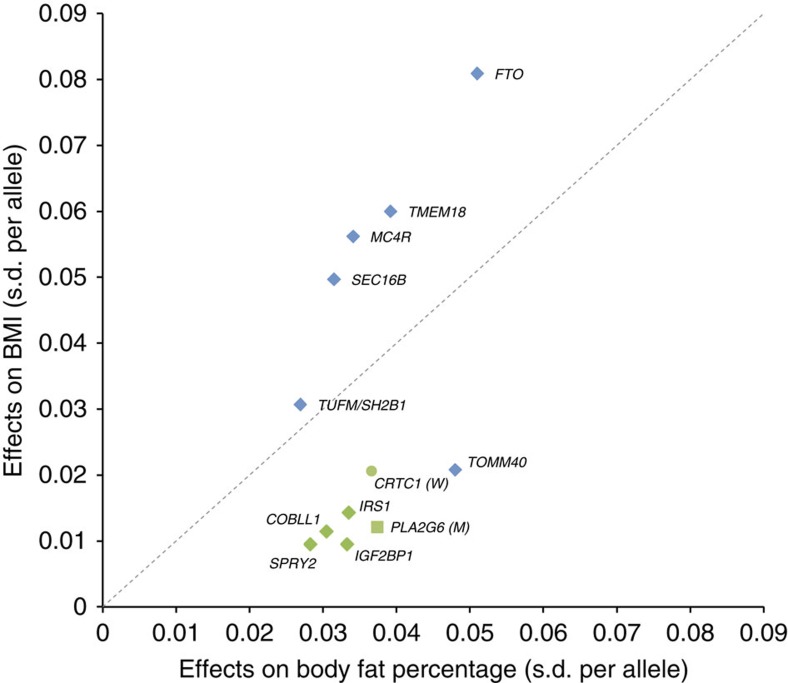
Comparison of effects of the 12 loci on body fat percentage (*x* axis) and on BMI (*y* axis). Both outcomes (BMI and BF%) were inverse normally transformed (mean 0, s.d. 1) such that effects sizes are at the same scales and directly comparable. Effect sizes for BMI were obtained from Locke *et al.*^19^. The allele effects for the *PLA2G6* (square) and *CRTC1* (round) loci were derived, respectively, from the men- and women-based meta-analyses. Six loci had first been identified for BMI (blue), whereas six others were first identified for BF% (green).

Index SNPs in the 12 established loci increase BF% by 0.024 to 0.051 s.d. per allele (equivalent to 0.16 to 0.33% in BF%, Table 1, Fig. 2). Given the high correlation between BF% and BMI, the BF% increasing alleles of each of the 12 loci are associated with increased BMI (Fig. 2, Table 2, and Supplementary Table 7). However, loci that had been previously identified for BMI, have larger effects (expressed in s.d. per allele) on BMI than on BF%, except the *TOMM40/APOE* locus, which has a substantially more pronounced effect on BF% than on BMI^25^ (Fig. 2). The *TOMM40/APOE* locus, together with the loci previously (*IRS1* and *SPRY2*) and newly (*COBLL1*/*GRB14*, *IGF2BP1*, *PLA2G6* and *CRCT1*) identified for BF% all have larger effects on BF% than on BMI (Fig. 2). This division based on effect sizes, illustrated in Fig. 2, suggests that *IRS1, SPRY2, COBLL1/GRB14, TOMM40/APOE, IGF2BP1, PLA2G6* and *CRTC1* affect adiposity in particular, which is not fully captured by BMI (which represents both lean and fat mass).

Of the 12 loci, four showed significant sex-specific effects. For the loci near *IRS1* and *PLA2G6,* the effect in men was twice as large as in women, whereas for the *TMEM18* and *CRTC1* loci the effect was two- to threefold larger in women than in men (Table 1). As the European-ancestry-only populations represent the vast majority (90%) of the total sample, effects sizes from European only and all-ancestry analyses were similar (Supplementary Tables 5 and 8).

In aggregate, the 12 loci explained 0.58% of the variance in BF% in men and women combined. Because of the sex-specific effects of four loci, the explained variance was slightly higher, when estimated in men (0.62%) and women (0.61%) separately. Individually, the *FTO* locus explained the most variance of all identified loci (0.12%) (Table 1).

### Cross-phenotype association with cardiometabolic traits

To gain insight in how the BF% loci affect anthropometric and cardiometabolic traits and comorbidities, we performed look-ups in the most recent large-scale GWAS meta-analyses from the GIANT (Genetic Investigation of ANthropometric Traits) consortium (WHR_adjBMI_ and height)^20^,^26^, the SAT-VAT consortium (abdominal visceral adipose tissue (VAT) and subcutaneous adipose tissue (SAT))^27^, the LEPgen consortium (circulating leptin), the GLGC (high-density lipoprotein cholesterol (HDL-C), low-density lipoprotein cholesterol (LDL-C) and triglycerides (TG))^28^, the MAGIC (fasting glucose and fasting insulin)^29^, DIAGRAM (T2D)^30^ and CARDIoGRAMplusC4D (CAD)^31^. To account for multiple testing, associations were considered statistically significant if *P* values were <5.2 × 10^−4^ (Bonferroni-corrected *P*=0.05/96 (12 SNP * eight trait groups)).

*Associations with anthropometric and adiposity traits*. The BF% increasing alleles for 11 of the 12 loci were associated with increased circulating leptin levels (*P*_binomial_=0.006), of which four reached statistical significance and another four were nominally significant (Table 2, Supplementary Table 7). These results are consistent with the notion that leptin is secreted by adipocytes proportional to adipose tissue mass.

The BF% increasing alleles of all 12 loci were associated with increased SAT and VAT (*P*_binomial_=0.0005), two (*FTO* and *TMEM18*) of which reached significance for association with SAT, and two (*FTO* and *TOMM40/APOE*) with VAT. The BF% increasing allele of the locus near *IRS1* was associated with a lower VAT/SAT ratio, indicative of a proportionally greater subcutaneous than visceral fat storage, as we have shown previously^13^ (Table 2, Supplementary Table 7).

As expected, most of the identified BF% loci showed no association with WHR_adjBMI_, as this trait, because of the adjustment for BMI, does not correlate with overall adiposity. Nevertheless, associations with WHR_adjBMI_ for two loci (*COBLL1/GRB14* and *TOMM40/APOE*) did reach statistical significance. The *COBLL1/GRB14* locus was previously identified as a WHR_adjBMI_ locus^11^. We show that it is the BF% increasing allele that is associated with lower WHR_adjBMI_, suggestive of a preferential gluteal rather than abdominal fat storage. Although the *COBLL1/GRB14* association with WHR_adjBMI_ is five times stronger in women than in men^11^, we observed no sex difference for association with BF% (Table 1). For the *TOMM40/APOE* locus, it is the BF% increasing allele that is also associated with increased WHR_adjBMI_, suggesting that the *TOMM40/APOE* locus increases abdominal and overall fat accumulation, at least in part, in an additive and independent manner. Furthermore, the BF% increasing allele was also significantly associated with increased VAT (Table 2, Supplementary Table 7) and liver fat storage (*P*=3.4 × 10^−4^, *n*=5,550, Methods section).

SNPs in three loci (*MC4R*, *PLA2G6* and *IGF2BP1*) showed significant association with height, two of which (*PLA2G6* and *IGF2BP1*) have not been reported in large GWAS studies before. Similar to the *MC4R* locus, the BF% increasing allele of the *PLA2G6* (rs3761445) was associated with greater adult height (*P*=6.7 × 10^−5^; Table 2, Supplementary Table 7). Following up this variant in data from the Early Growth Genetics Consortium, we found that the BF% increasing allele was associated with higher birth weight (*P*=0.003, *n* up to 26,836; ref. 32) and greater prepubertal height (*P*=0.007, *n*=13,948; ref. 33), yet not with growth during or timing of puberty (Supplementary Table 10)^33^. In contrast, the BF% increasing allele in *IGF2BP1* (rs9906944) was associated with shorter height (Table 2, Supplementary Table 7), a cross-phenotype association pattern that is consistent with the effects of the GH/IGF1 axis^34^. SNPs in *IGF2BP1*, in linkage disequilibrium (LD) with rs9906944 (*r*^2^_EUR_=0.47), have been previously implicated with primary tooth development in infancy^35^. Consistently, the BF% increasing allele of *IGF2BP1* (rs9906944) showed association with a later eruption of the first tooth (*β*=0.16 months per allele; *P*=3.1 × 10^−8^) and reduced number of teeth at 1 year (*β*=−0.14 number of teeth at age 1 year per allele; *P*=1.1 × 10^−7^; ref. 35). Even though this suggests a role in maturation, we found no evidence for association with pre-pubertal height or pubertal growth and timing (Supplementary Table 10)^33^ or age at menarche (*β*=0.01 age of menarche (years) per allele; *P*=0.11; ref. 24). Although this locus harbours a number of genes, data in rodents suggest that *IGF2BP1* might be a potential candidate gene driving the associations observed here, as Igf2bp1 knockout mice demonstrate fetal and postnatal growth retardation^36^.

Taken together, alleles of each of the 12 loci are associated with increased BF%, yet their associations with other anthropometric traits differ, which in turn might result in varying impacts on cardiometabolic health.

*Associations with cardiometabolic traits*. Although phenotypic correlations observed in epidemiological studies have shown that increased adiposity is associated with increased cardiometabolic risk, the BF% increasing alleles of identified loci do not always associate with poorer health outcomes (Table 2 and Supplementary Table 11). For some loci, the BF% increasing allele may even have significant protective effects, as we have shown previously for the locus near-*IRS1* (ref. 13).

For the loci in/near *FTO, MC4R, TMEM18, TUFM/SH2B1 and SEC16B,* which were all five previously established for BMI, the observed cross-phenotype associations with cardiometabolic traits are generally directionally consistent with the phenotypic correlations. Specifically, their BF% increasing allele is typically associated with an unfavourable lipid profile and increased insulin resistance (Table 2, Supplementary Tables 12 and 13). These cross-phenotype associations translate in increased risk of T2D and CAD and higher CRP levels, at least for the *FTO, TMEM18 and MC4R* loci (Fig. 2, Table 2, Supplementary Tables 9,12 and 13).

For the remaining seven loci, which all have a larger effect on BF% than on BMI (Fig. 2), the cross-phenotype associations are not always consistent with the phenotypic correlation between BF% and cardiometabolic traits. For example, the *COBLL1/GRB14* locus was previously identified for its association with fasting insulin^29^, TG^37^, HDL-C^37^ and,T2D risk^30^ (Table 2, Supplementary Tables 12 and 13). However, we show for the first time that it is the BF% increasing allele that is associated with a protective effect on cardiometabolic health; that is, with significantly lower TG levels and higher HDL-C levels, and a reduced risk of T2D (Table 2, Supplementary Tables 12 and 13). This association signature of the *COBLL1*/*GRB14* locus is consistent with the observation that its BF% increasing allele is associated with a lower WHR_adjBMI_, corresponding to a proportionally lower abdominal and higher gluteal fat accumulation and, at nominal significance, with SAT but not with the metabolically more harmful VAT. The *COBLL1*/*GRB14* association signature is similar to that of the near-*IRS1* locus (Table 2, Supplementary Tables 12 and 13), and suggest that the beneficial cardiometabolic effects of the loci near *COBLL1/GRB14* and *IRS1* might be mediated through a favourable influence on body fat distribution, despite increased adiposity.

The BF% increasing allele of rs6857 near *TOMM40*/*APOE* is significantly associated with increased overall adiposity (BMI), abdominal adiposity (WHR_adjBMI_), visceral adipose tissue (VAT) and liver fat storage, which may be mediating the nominally significant association with increased fasting glucose and risk of T2D (Table 2 and Supplementary Table 13). However, most notably, the BF% increasing allele was also highly significantly associated with a favourable lipid profile and reduced risk of CAD (Table 2 and Supplementary Table 12). The associations with lipid levels seem to be only partially driven by the nearby *APOE* locus for which previously highly significant associations with LDL-C^37^, CRP^38^ (both rs4420638), HDL-C and TG (rs439401; ref. 37) levels have been reported (Supplementary Fig. 7). These two SNPs (rs4420638, rs439401) are in low LD with each other (*r*^2^_EUR_=0.13, D′_EUR_=0.96), and with the here-identified *TOMM40*-rs6857 (*r*^2^_EUR_=0.39, D′_EUR_=0.72 and *r*^2^_EUR_=0.06, D′_EUR_=0.77, respectively). Although the *APOE*-rs4420638 allele shows evidence of association with BF% (*P*=3.9 × 10^−5^), the association is completely abolished (*P*=1.00) after conditioning for *TOMM40*-rs6857 (Supplementary Table 14). The *APOE*-rs439401 SNP, previously associated with HDL-C levels, was not associated with BF% (*P*=0.72). Conversely, the *TOMM40*-rs6857 associations with TG (*P*=4.5 × 10^−19^; *P*_conditional_=3.6 × 10^−5^) and HDL-C (*P*=2.6 × 10^−17^; *P*_conditional_=8.4 × 10^−14^) remain significant after conditioning for the lipid-associated *APOE* SNPs (rs4420638, rs439401), whereas its association with LDL-C (*P*=5.1 × 10^−110^; *P*_conditional_=0.97) is completely abolished after adjusting for the *APOE-*rs4420638 (Supplementary Table 14). Taken together, these observations show that associations of *TOMM40*-rs6857 are independent from the HDL-C and TG-associated *APOE*-rs439401 and partially independent from the LDL-C-associated *APOE-*rs4420638 (Supplementary Table 14). Another SNP (rs2075650) in this region, in high LD (*r*^2^_EUR=_0.77, D′_EUR_=0.96) with the *TOMM40*-rs6857 and associated with BF% (*P*=1.4 × 10^−7^), has been previously identified for its association with Alzheimer's disease^39^, cognitive function^40^ and ageing^41^, with the BF% increasing allele being associated with reduced risk of Alzheimer's disease, slower cognitive decline and increased longevity.

Although we do not observe association of *IGF2BP1*-rs9906944 with circulating lipid levels or glycemic traits, interestingly, the BF% increasing allele is significantly associated with increased risk of T2D and CAD, and with higher CRP levels (Table 2, Supplementary Tables 9,12 and 13).

The sex-specific effect of *PICK1*/*PLA2G6*-rs3761445 does not translate in sexual dimorphic associations with other traits (Table 2, Supplementary Tables 12 and 13). Interestingly, the BF% increasing allele is associated with a favourable lipid profile; in particular with lower TG levels (*P*=8.1 × 10^−12^) and higher HDL-C levels (*P*=3.9 × 10^−6^, Supplementary Table 12), but no association with CAD risk was observed (Supplementary Table 12). The *PICK1*/*PLA6G2*-rs3761445 is in moderate LD with SNPs identified before for nevus count (rs2284063, *r*^2^_EUR_=0.67, D′_EUR_=0.90; ref. 42) and melanoma risk (rs738322, *r*^2^_EUR_=0.77, D′_EUR_=0.98; refs 42, 43). Consistently, the rs3761445 BF% increasing allele is associated with a lower number of cutaneous nevi (−0.067 nevi/allele, *P*=9.4 × 10^−6^; ref. 43) and reduced melanoma risk (OR=0.86 per allele, *P*=5.3 × 10^−10^; ref. 44).

The BF% increasing allele of *CRTC1*-rs757318, which showed a significantly stronger association in women than men, was not associated with any of the cardiometabolic traits in either sex-stratified or sex-combined results. Rs757318 is in moderate LD (*r*^2^_EUR_=0.57, D′_EUR_=1) with another *CRTC1* SNP (rs10423674) that was previously established for age at menarche^24^ and, consistently, also the rs757318 BF% increasing allele was significantly associated with earlier age at menarche (*β*=−0.03 years per allele; *P*=2.4 × 10^−10^; ref. 24).

### Functional annotation of genome-wide significant loci

The causal genes and/or variants underlying most of the BF% associated loci remain unknown. For the 12 genome-wide significant loci, and also for putative loci (*P*<1 × 10^−5^), we used multiple complementary approaches to prioritize candidate genes and/or variants and to elucidate the mechanisms involved in body fat regulation. These approaches include identification of nearby coding variants or copy-number variants (CNVs), *cis*-eQTL analysis, epigenetic marker and functional regulatory genomic element analysis, pathway and tissue enrichment analysis, and a transgenic *Drosophila* model.

*Coding variants and CNV analysis*. Among the 12 index SNPs, only rs4788099 near *SH2B1* was in high LD with seven coding variants (*r*^2^_EUR_>0.7) in nearby genes (*APOBR, SH2B1* and *ATP2A1*; Supplementary Table 15, Methods section). Two of these seven variants were non-synonymous, of which, one, Thr484Ala (rs7498665) in *SH2B1*, was in perfect LD with our index SNP. Thr484Ala shows a high degree of conservation, but was predicted to be functionally benign by PolyPhen and tolerated by SIFT. None of the other 11 index SNPs were in high LD with coding or CNVs.

*eQTL analysis*. We examined *cis*-associations between each index SNP and gene expression of transcripts within 1 Mb-region flanking the respective SNP (Supplementary Tables 16 and 17, Methods section). As shown previously^13^, the BF% increasing allele of rs2943652 near *IRS1* is associated with increased IRS1 expression in omental and subcutaneous fat. SNPs within the same locus (LD *r*^2^_EUR_>0.95) have also been shown to be associated with increased IRS1 expression in skeletal muscle^45^. We also identified significant (*P*<1 × 10^−5^ or 5% FDR) eQTLs for other BF% associated loci, even after conditioning for the most significant SNP-transcript association in the regions. The BF% increasing allele of *COBLL1*/*GRB14*-rs6738627 is associated with lower expression of GRB14, whereas there is no evidence of association with COBLL1 expression. The BF% increasing allele for *PLA2G6*/*MAFF*-rs3761445 is associated with lower expression of MAFF and TMEM184B in omental and subcutaneous fat. *TUFM*/*SH2B1-*rs4788099 is associated with the expression of a number of genes, such as TUFM (blood), APOBR (blood), SBK1 (blood), SULT1A2 (omental and subcutaneous fat) and SH2B1 (omental fat).

*Epigenetic marker and functional regulatory genomic element analysis*. We examined the overlap of 746 variants in LD (*r*^2^_CEU_>0.70) with the 12 index SNPs with regulatory elements in brain, blood, liver, adipose and pancreatic islets from the ENCODE Consortium and Roadmap Epigenomic Projects (Supplementary Table 18). Across loci, 179 (24%) variants showed evidence of being located in a regulatory element as defined by overlapping variants in two or more data sets from the same tissue (Supplementary Table 19). Promoter variants, located within 2 kb of a transcription start site, overlapped with an average of 22 regulatory elements, while more distal variants (>2 kb) overlapped with an average of nine elements.

**Figure 3:**
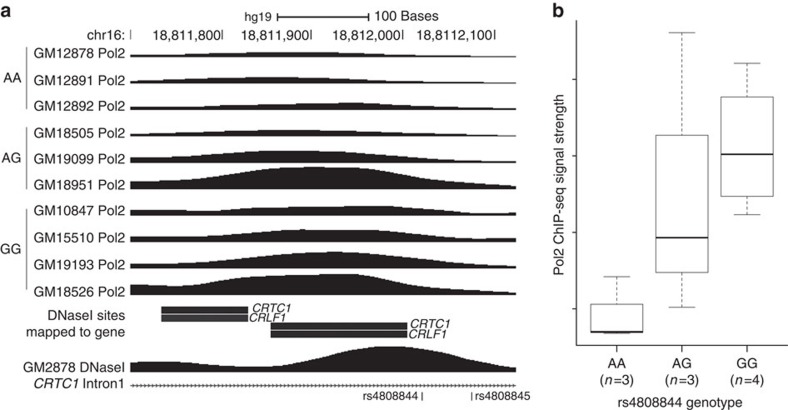
Genotype influences Pol2 binding at rs4808844. (**a**) UCSC Genome browser track (hg19) of chromosome 16 displaying Pol2 binding signal in 10 lymphoblastoid cell lines (LCLs), grouped by genotype and correlations between DNaseI hypersensitivity and nearby gene transcription. (**b**) Binding signals from Pol2 ChIP-seq from 10 LCLs, grouped by genotype.

Two of the distal variants with the greatest amount of regulatory overlap were rs4808844 and rs4808845 (43 and 41 elements, respectively; Supplementary Table 19). These variants are located 58 bp apart in intron 1 of *CRTC1* and overlap evidence of open chromatin, histone marks that are characteristic of active transcription regulation and Pol2 binding (Fig. 3a). We found that rs4808844 was significantly associated (*P*=0.036) with Pol2 binding signal strength (Fig. 3b). In addition, DNaseI hypersensitivity signal in this region has been shown to negatively correlate with *CRTC1* and *CRLF1* transcription levels across many cell types^46^. These data suggest that rs4808844 and rs4808845, both in high LD (*r*^2^_CEU_=0.76 and 0.79, respectively) with our index SNP (rs757318), may influence the transcription of these and/or other nearby genes.

We further characterized variants overlapping with regulatory elements at each of the 12 loci using RegulomeDB, and two loci stood out. In the *TUFM-SH2B1* region, three SNPs (rs4788084, rs1074631 and rs149299) in LD (*r*^2^_CEU_=0.82, 0.76 and 0.75, respectively) with rs4788099 are located in an EBF1-binding protein ChIP-seq signal in lymphoblastoid cells. In addition, rs4788084 is located within an EBF1-binding motif. EBF1 is involved in the thalamic axon projection into the neocortex^47^ and the genetic variants around rs4788099 might affect the regulation of EBF1 of the nearby *SH2B1* (ref. 48). In the *PLA2G6/PICK1* region, rs4384 in LD with rs3761445 (*r*^2^_EUR_=0.73) overlapped with more elements (50 elements in four tissues, Supplementary Table 19) than any other distal variant. This variant is located in a HEN1-binding motif with evidence of a DNase footprint in multiple cell types (Supplementary Fig. 8). HEN1 is a transcription factor potentially involved in the CNS development^49^.

*Pathway, network and tissue-enrichment analysis*. To test for enrichment and define pathways and networks between the genes harboured by the 12 GW-significant loci and 31 loci with putative evidence (*P*<1 × 10^−5^) of association with BF%, we applied a number of approaches (see Methods section). Neither DEPICT (data-driven enrichment prioritized integration for complex traits)^50^ nor Ingenuity IPA identified pathways, tissues or networks that were significantly enriched among the genes across the 43 loci (Supplementary Tables 20–22). Also, GRAIL (Gene Relationships Among Implicated Loci), which searches the published literature to identify relationships between genes, and DAPPLE (Disease Association Protein–protein Link Evaluator), which tests for protein–protein interactions, did not identify significant connection between any of the genes in the identified loci. Their limited power may be due to the relatively small number of loci identified in this meta-analyses or to limited knowledge related to adipogenesis^51^.

### Experimental follow-up of candidate genes in *Drosophila*

We used *Drosophila* as a fast and inexpensive model to help prioritize which genes within the identified loci are the most likely candidates to underlie the observed associations.

To gain first insights in the potential candidacy of the genes located within the 12 BF% associated loci, we performed a look-up in data from a genome-wide transgenic RNAi screen for fat content in adult *Drosophila*^52^. In that screen, whole-body TG, also in *Drosophila* the major lipid storage form, were used as a direct measure of fly adiposity upon activation of a heat shock-inducible Hsp70-GAL4 system. As such, transgenic fly lines were made to test the adiposity regulating potential of 10,489 of the ∼14,000 annotated *Drosophila* protein coding genes. Of the 80 genes located within a 1 Mb-window of each of the 12 index SNPs, 44 *Drosophila* orthologues were available, yet, 12 of these 44 transgenic RNAi fly lines were too weak to be screened. Of the remaining 32 fly lines, 15 fly lines had substantially lower (>2 s.d. less) whole-body TG than the wild-type flies, whereas five fly lines showed higher TG (>2 s.d. more) (Supplementary Table 23). Next, we selected one to three candidate genes within each of the 12 loci based on their potential role in adipocyte metabolism. We knocked down their corresponding orthologues in *Drosophila* that were subsequently exposed to a high-sugar diet (Supplementary Table 24), as described before^53^. Both *Drosophila* experiments pinpoint the *SPRY2* (or *sty*) as the potential causal gene within the locus; that is, knockdown flies for *sty* have significantly lower whole-body TG levels than wild-type flies. While the genome-wide transgenic RNAi screen pointed towards the *CRTC1* gene in the *CRTC1* locus, we could not confirm a role for *CRTC1* in the knockdown experiment.

### Established loci and body fat percentage

The most recent GWAS meta-analysis for BMI, including nearly 340,000 individuals, identified 97 loci that reached GWS^19^. Each of the 97 BMI-associated SNPs showed directionally consistent association with BF% (*P*_binomal_<1 × 10^−4^), 71 of which also reached nominal statistical significance (Supplementary Table 25). One of the reasons for the non-significance for the remaining loci might be insufficient power as the current final meta-analysis sample size for BF% was only one-third of that for BMI.

Of the 12 loci previously identified through GWAS for extreme and early-onset obesity^7^,^12^,^54^,^55^, 11 showed directionally consistent association with BF% (*P*_binomal_<0.006), of which five also reached nominal statistical significance (Supplementary Table 25).

## Discussion

Our meta-analysis of data from more than 100,000 individuals identified 12 loci significantly associated with BF%. While a recent GWAS including more than 340,000 individuals reported nearly 100 loci associated with BMI, a commonly used proxy measure for overall adiposity, four (*SPRY2*, *IGF2BP1*, *PLA2G6* and *CRTC1*) of the 12 BF% associated loci did not reach GWS for BMI, despite the enormous sample size^19^. This observation most likely reflects the heterogeneity of BMI as a marker of overall adiposity and emphasizes the increased statistical power of more precisely measured phenotypes.

The 12 BF% associated loci divide into two distinct groups. The first group comprises the five loci (*FTO*, *MC4R*, *TMEM18*, *SEC16B* and *SH2B1*) of which the association is stronger with BMI than with BF%, suggesting that they affect both fat mass and lean mass. All five loci have been identified and described in detail before in relation with BMI^5^,^10^,^19^. Their associations with cardiometabolic outcomes are predictable, reflecting the phenotypic correlations with BF%; that is, their BF% increasing alleles are associated with an unfavourable glycemic and lipid profile and with an increased risk of T2D and CVD.

The second group, comprising the remaining seven loci (*IRS1*, *SPRY2*, *TOMM40*/*APOE*, *CRCT1*, *PLA2G6*, *IGB2BP1* and *COBLL1*/*GRB14*), all show a more pronounced effect on BF% than on BMI, suggesting a specific effect on adiposity rather than on overall body mass. Most notably, the association patterns with cardiometabolic traits of this group of loci, as opposed to the first group, often do not reflect the phenotypic correlations. For example, as we have described before, the BF% increasing allele of the index SNP 500 kb upstream of *IRS1*, which affects IRS1 expression, is associated with a favourable cardiometabolic risk profile, including a reduced risk of T2D and CVD^13^. We showed that this association signature, which goes against the phenotypic correlations, could be explained by an effect on fat distribution, as the BF% increasing allele was associated with increased subcutaneous, but not with the metabolically more harmful visceral fat^13^. The locus between *GRB14* and *COBLL1* shows a similar association signature. In fact, this locus was first described for its association with a lower WHR_adjBMI_^11^ and reduced risk of T2D^30^. Here, we show that the same allele is associated with increased BF%, suggesting that the association with WHR_adjBMI_ likely reflects a proportionally greater fat accumulation at hip and thighs rather than at the waist. Although this locus requires further experimental follow-up, current observations point towards *GRB14* as the candidate gene in this locus. *GRB14* encodes a protein that binds directly to the insulin receptor (IR), and the BF% increasing allele of the index SNP is associated with reduced GRB14 expression in adipose tissue. This is consistent with previous observations showing that Grb14/GRB14 expression is increased in adipose tissue of insulin-resistant rodents and in obese patients with T2D^56^. Furthermore, Grb14-deficient mice show improved glucose homeostasis and enhanced insulin action through increased IR-mediated IRS1 phosphorylation in the liver and skeletal muscle^57^. The similar cross-phenotype association signatures of the *IRS1* and *GRB14/COBLL1* loci might be a reflection of the close interaction between IRS1 and GRB14 in the IR-signalling pathway.

The BF% increasing allele of the *PLA2G6* locus is associated with lower insulin and TG levels and reduced T2D risk, particularly in men. *PLA2G6* is the nearest gene and encodes a calcium-independent phospholipase A2 involved in the hydrolysis of phospholipids. However, this locus harbours a number of other genes that would make plausible candidates for driving the cross-phenotype associations, including *PICK1*, which is membrane sculpting BAR domain protein. PICK1-deficient mice and flies display marked growth retardation, which at least in mice, might be due to impaired storage and secretion of growth hormone from the pituitary and possibly insulin from the pancreas^58^. PICK1-deficient mice, despite their smaller size, demonstrate increased body fat and reduced lean mass, reduced TG levels and impaired insulin secretion, which was compensated by increased insulin sensitivity^58^. Given the locus' association with nevus count, SOX10, which encodes a member of the SOX (SRY-related HMG-box) family of transcription factors, is another candidate gene in this locus. SOX genes are involved in the regulation of embryonic development and *SOX10* in particular is important for the development of neural crest and peripheral nervous system. Mutations in *SOX10* have been implicated in uveal melanoma and Waardenburg syndrome, which presents with pigmentation abnormalities and hearing loss, and Kallmann syndrome, which presents with failure to start or complete puberty and hypogonadotropic hypogonadism (short stature, absence of puberty and sex hormones, among others) and absence of smell^59^,^60^. The phenotype similarity of these syndromes and the association signature may suggest that *SOX10* could be driving the associations observed for the *PLA2G6* locus.

The *TOMM40*/*APOE* locus is another locus with an intriguing association signature; while the BF% increasing allele has an unfavourable effect on glycemic traits and T2D risk, it is associated with a favourable lipid profile and reduced risk of CVD. The high LD in this region poses a major challenge to elucidate whether the association with lipid traits is due to a ‘spillover' effect from nearby lipid-associated loci in *APOE*. Using conditional analyses, we provide evidence suggesting that at least the association with lower TG and high HDL-C levels might be distinct from previously reported loci. Of interest is that the BF% increasing allele seems to be associated with markers of increased longevity^41^.

The *CRTC1* locus is another gene-rich locus, but given the epigenetic marks in this gene and data from animal models, *CRTC1* poses to be a good candidate gene. *CRTC1* is primarily expressed in the brain, and it may affect leptin anorexic effect in the hypothalamus^61^. *CRTC* knockout mice demonstrated hyperphagia, increased white adipose tissue and infertility^61^.

Our meta-analysis was limited by the fact that participating studies all had imputed HapMap reference panels for autosomal chromosomes and that the analysis model assumed additive effects. Future discovery efforts based on genome-wide imputation of 1000 Genomes reference panels, that include X- and Y-chromosomes and that also test recessive and dominant inheritance, will allow for the discovery of more and lower-frequency variants and for refining association signatures of already established BF%-associated loci.

Taken together, our expanded genome-wide meta-analyses of BF% has identified a number of loci with distinct cross-phenotype association signature that, together with our functional follow-up analyses, facilitated the identification of strong positional candidates. Particularly striking is that two of the 12 loci harbour genes (*IRS1*, *GRB14*) that influence insulin receptor signalling, and two other loci contain genes (*IGF2BP1*, *PICK1)* that are involved in the GH/IGF1 pathway, that in turn also relates to insulin receptor signalling.

## Methods

### Discovery of new loci

*Study design*. A two-stage meta-analysis was performed to identify loci associated with BF%. In Stage 1, we conducted two parallel meta-analyses; one meta-analysis combined summary statistics from 43 GWAS, totalling up to 76,137 adult individuals (65,831 European ancestry, 7,557 South Asian ancestry, 2,333 East Asian ancestry and 416 African Americans), and the other meta-analysis combined summary statistics from 13 additional studies genotyped using the Metabochip, totalling up to 24,582 individuals (23,469 Europeans and 1,113 African Americans). In Stage 2, we combined the GWAS meta-analysis results and Metabochip meta-analysis results from Stage 1 (Supplementary Table 1 and Supplementary Figs 1 and 2) in one final meta-analysis, including 100,716 individuals from 56 studies. All the studies were approved by their local institutional review boards and written consent was obtained from all the study participants.

Although our primary analysis, described above, combined all the data available to us, in the secondary analyses, we conducted stratified analyses for (1) all-ancestry men-only, (2) all-ancestry women-only, (3) European ancestry, (4) European ancestry men-only and (5) European ancestry women-only (Supplementary Tables 4–6 and Supplementary Figs 2–5).

*Phenotype*. BF% in each cohort was measured either with bioimpedance analysis (BIA) or dual energy X-ray absorptiometry (DEXA) as described in detail before^13^. For each study, BF% was adjusted for age, age^2^ and study-specific covariates (for example, genotype-based principle components, study centre and others), if necessary. For studies of unrelated individuals, the residuals were calculated separately in men and women, and in cases and controls. For studies of family-based design, the residuals were calculated in men and women together, and sex was additionally adjusted in the model. The residuals were then inverse normally transformed for association testing. For studies of family-based design, the family relatedness was additionally adjusted in the association testing.

*Sample quality control, imputation and association*. Each study did the study-specific quality control (QC) (Supplementary Table 2). The GWAS common SNPs were imputed in each study using the respective HapMap Phase II (Release 22) reference panels (EUR for studies of European-ancestry populations, CHB+JPT for studies of Eastern Asian ancestry populations, and CEU+YRI+CHB+JPT for studies of Indian Asian ancestry populations and African American populations). Individual SNPs were associated with inverse normally transformed BF% residuals using linear regression with an additive model. All the SNPs with low imputation scores (MACH *r*^*2*^-hat <0.3, IMPUTE proper_info <0.4 or PLINK info <0.8) and a MAC ≤3 were removed. The EasyQC software was used for detailed QC of study level analyses and meta-level analysis, as described elsewhere^62^.

*Meta-analysis*. Meta-analyses were performed using inverse variance-weighted fixed-effect method in METAL. Inflation before genomic control (GC)-correction was generally low in all-ancestry (*λ*_men+women_=1.13; *λ*_men_=1.07; *λ*_women_=1.09) and European-only (*λ*_men+women_=1.13; *λ*_men_=1.07; *λ*_women_=1.10) analyses. To reduce the inflation of the test statistics from potential population structure, individual GWAS results and GWAS meta-analysis results were corrected for GC using all SNPs. Individual Metabochip results and Metabochip meta-analysis results were GC-corrected using 4,425 SNPs, which are derived from pruning of QT-interval replication SNPs within 500 kb of an anthropometry replication SNP on the Metabochip. The GC-corrected GWAS and Metabochip meta-analysis results were finally meta-analysed (Supplementary Fig. 1).

Using the LD score regression method in the European-only meta-analyses suggests that the observed inflation is not due to population substructure^63^. The regression intercept, which estimates inflation after removing polygenic signals, was 1.0045 (with *λ*_GC_=1.136 and mean *χ*^2^=1.16) for sex-combined, 0.999 (*λ*_GC_=1.062 and mean *χ*^2^=1.079) for men-only and 1.014 (*λ*_GC_=1.105 and mean *χ*^2^=1.112) for women-only analyses. Using these regression intercepts, rather than the *λ*_GC_, to correct our meta-analyses, results in more significant associations (for example, for the rs1558902-*FTO* SNP, *P*=3.24 × 10^−27^ in the modified European sex-combined meta-analysis compared with *P*=1.1 × 10^−25^ (Supplementary Table 6)). Overall, however, the less stringent correction did not result in the identification of novel loci.

*Identification of novel loci*. Each unique locus was defined as ±500 kb on either side of the most significant SNP that reached a GWS threshold (*P*<5 × 10^−8^) in the meta-analysis. These GWS-index SNP loci from the primary analysis as well as from secondary analyses were highlighted for further analyses (Table 1 and Supplementary Tables 4–6). The genotype data for the genome-wide significant SNPs was of high quality with a median imputation score of ≥0.95 (Supplementary Table 26). The fifth percentile for all SNPs was ≥0.80, except for the previously established *TOMM40* SNP (P5=0.52).

*Joint and conditional multiple SNP association analysis*. We used the GCTA approach to identify potential additional signals in regions of GWS-index SNP. This approach uses summary meta-analysis statistics and a LD matrix from an ancestry-matched sample to perform approximate joint and conditional SNP association analysis. Although our primary analyses were based on all ancestry populations, the 12 GWS-index SNPs were strongly associated with BF% in European populations, 6 of them reaching the GWS (Supplementary Table 5). The estimated LD matrix based on 6,654 unrelated individuals of European ancestry in ARIC cohort was used in the analysis.

*Heterogeneity among studies*. The potential heterogeneity in the effect estimates for our GWS-index SNPs were investigated between men and women in all-ancestry populations and in European populations, and between individuals of European ancestry and individuals of all ancestry. We also tested for heterogeneity between results from studies that used BIA for BF% assessment and that used DEXA. Heterogeneity was assessed using a *t*-statistic, *t*=(*β*_1_−*β*_2_)/(se_1_^2^+se_2_^2^−2*r* se_1_*se_2_)^½^ to account for relatedness, where *β*_1_ and *β*_2_ are the effect size estimates, se_1_ and se_2_ are the corresponding standard errors and *r* is Spearman's correlation coefficient of beta values between men and women or between European ancestry and all ancestry.

*Variance explained*. The variance explained by each GWS-index SNP was calculated using the effect allele frequency (*f*) and beta (*β*) from the respective meta analyses using the formula^6^ of Explained variance =2*f*(1−*f*)*β*^2^.

### Cross-trait association lookups

*Cardiometabolic consortia*. To explore the relationship between BF% and an array of cardiometabolic traits and diseases, the association results for the 12 GWS-index SNPs were requested from seven primary cardiometabolic genetic consortia: the LEPgen consortium (circulating leptin, Kilpeläinen *et al.*, in preparation), VATGen consortium^27^, GIANT (BMI, height and WHR_adjBMI_)^19^,^20^,^26^, GLGC (HDL-C, LDL-C, TG, TC)^28^, MAGIC^29^, DIAGRAM (T2D)^30^ and CARDIoGRAMplusC4D (CAD)^31^. On the basis of known correlations among these cardiometabolic traits, we considered circulating leptin levels, abdominal adipose tissue storage, height, WHR_adjBMI_, plasma lipid levels, plasma glycemic traits, T2D and CAD as eight independent trait groups. In addition, the associations for these 12 SNPs were also looked up in four consortia that examined phenotypes more distantly related to BF%: ADIPOGen (BMI-adjusted adiponectin)^64^, ReproGen (age at menarche)^24^, liver enzyme meta-analysis^65^ and CRP meta-analysis^38^. For certain GWAS-index SNPs, we also did specific lookups: rs6857 association in liver fat storage, rs3761445 associations in cutaneous nevi and melanoma risk meta-analysis^42^,^43^,^44^, early growth genetics (birth weight^32^ and pubertal height^33^), insulin-like growth factor 1 meta-analysis (Teumer *et al.* under review) and CHARGE testosterone meta-analysis^66^, and rs9906944 associations in tooth development meta-analysis^35^ and Early Growth Genetics Consortium (birth weight^32^ and pubertal height^33^).

*NHGRI GWAS catalogue lookups*. We manually curated and searched the National Human Genome Research Institute (NHGRI) GWAS Catalogue ( www.genome.gov/gwastudies) for previously reported associations for SNPs within 500 kb and *r*^*2*^>0.7 (1000 Genomes Pilot1 EUR population based on SNAP: http://www.broadinstitute.org/mpg/snap/ldsearch.php) with each of the 12 GWS-index SNPs. All previously reported associations that reached *P*<5 × 10^−8^ were retained (Supplementary Table 11).

### Coding variants and CNVs

To determine whether any of our 12 GWS-index SNPs might be tagging potentially functional variants, we identified all variants within 500 kb and in LD (*r*^2^>0.7, HapMap release 22/1000 Genomes Pilot1 EUR) with our GWS-index SNPs. As such, we identified 776 variants and annotated each of them using Annovar ( http://www.openbioinformatics.org/annovar/). The predicted functional impacts for coding variants were accessed via the Exome Variant Server ( http://evs.gs.washington.edu/EVS/) for PhastCon, Grantham, GERP and PolyPhen, and were also from SIFT ( http://sift.jcvi.org/). To determine whether any of the 12 GWS-index SNPs tagged (*r*^2^>0.7) CNVs, all genetic variants (SNV, Indel and SVS) within a 1 Mb window of the index SNPs from the 1000 Genomes Project EUR population (Phase 1) were downloaded. The LD indexes were calculated between each of the 12 GWS-index SNPs and any nearby CNV variants.

### Analyses of eQTLs

The *cis*-associations between 12 GWS-index SNPs and expression of nearby genes (±500 kb of the index-SNP) were examined in the whole blood (*n*=2,360) from the eQTL meta-analysis study^67^, the abdominal fat tissue (*n*=742 for omental fat and *n*=610 for subcutaneous fat) from the bariatric surgery study^68^, the abdominal subcutaneous fat tissue (*n*=54) and gluteal subcutaneous fat tissue (*n*=65) from the MolOBB study^69^, and the brain tissue from the cortical brain study (*n*=193; ref. 70). Conditional analyses were conducted by including both GWS-index SNP and the most significant *cis*-associated SNP for the given transcript in the model to examine whether observed associations were driven by our GWS-index SNP or by other nearby variants. Conditional analyses were conducted for all tissues except the brain tissue.

### Regulatory annotation using ENCODE and Roadmap

*Regulatory element overlap*. We identified variants in LD (*r*^2^>0.7, 1000 Genomes Project Pilot, EUR) with each of the 12 GWS-index SNPs and tested for overlap between these variants and elements from regulatory datasets. In total, 746 variants at the 12 GWS-index loci were examined for overlap with regulatory elements in 181 data sets (Supplementary Tables 18 and 19) from five tissues (blood, brain, liver, adipose tissue and pancreatic islets). These data sets, downloaded from the ENCODE Consortium and Roadmap Epigenomics Projects, identify regions of open chromatin (DNase-seq, FAIRE-seq), histone modification signal enrichment (H3K4me1, H3K27ac, H3K4me3, H3K9ac and H3K4me2), and transcription factor binding in cell lines and tissues believed to influence BF%. When available, we downloaded data processed as a part of the ENCODE Integrative Analysis. Roadmap Epigenomics sequencing data were processed with MACS2 and the same irreproducible discovery rate pipeline used in the ENCODE Integrative analysis when multiple data sets were available, or MACS2 alone when only a single replicate was available.

*Pol2 binding*. We tested for correlation between Pol2 binding strength and genotype in lymphoblastoid cell lines at two SNPs, rs4808844 and rs4808845 that are in LD with GWS-index SNP of rs757318 in *CRTC1*. Pol2 binding data uniformly processed as part of the ENCODE Integrative analysis were download for 10 lymphoblastoid cell lines (GM10847, GM12878, GM12891, GM12892, GM15510, GM18505, GM18526, GM18951, GM19099, GM19193). We examined the alleles present at these variants in Pol2 ChIP-seq alignment BAM files to determine sample genotypes and compared these with genotypes generated by the 1000 Genomes Project for the same samples. For the eight samples also genotyped by the 1000 Genomes Project, genotype calls were 100% concordant. Correlation between genotype and Pol2 binding signal at each SNP was calculated in R using a linear model (signal∼genotype).

*RegulomeDB annotation*. We further characterized the variants at selected loci using the web-based tool RegulomeDB ( http://regulomedb.org/). The reference sequence identifiers of variants that overlap two or more regulatory elements in the same tissue were used to conduct the RegulomeDB search.

### Pathway, network and tissue-enrichment analysis

To define pathways, networks and tissue enrichment, we extended the list of genome-wide significant loci to also include loci that showed putative (*P*<1 × 10^−5^) association with BF% (using the same criteria described above to define independent loci). As such loci, represented by 43 index SNPs, were considered for gene prioritization, pathway enrichment (DEPICT, Ingenuity Pathway Analyses), gene relationship analysis (GRAIL) and protein–protein interaction analyses (DAPPLE).

*Data-driven enrichment prioritized integration for complex traits*. Details of this method are provided in Pers *et al.*^50^ DEPICT is designed to systematically identify the most likely causal gene at a given locus, to test gene sets for enrichment for genetic associations, and to identify tissues and cell types in which genes from associated loci are highly expressed.

DEPICT assigned genes to the 43 associated loci if the genes resided within the associated LD region (*r*^2^>0.5) of a given associated SNP. After merging overlapping regions and discarding regions that mapped within the extended major histocompatibility complex locus, we were left with 42 non-overlapping regions that covered a total of 82 genes. We then used DEPICT to test enrichment at these loci for a total of 14,461 reconstituted gene sets, and for 209 tissue and cell type annotations.

*Ingenuity pathway analyses*. We used HaploReg v2 ( http://www.broadinstitute.org/mammals/haploreg/haploreg.php) and adopted a stringent LD (*r*^2^>0.8 in 1000 Genome phase 1 EUR) to extract all the nearby genes (88 genes in total) of the index SNPs based on both GENCODE and RefSeq. For 65 out of them, they were successfully mapped to the Ingenuity Knowledge Base, and those unmapped genes are mainly lincRNA, miRNA, antisense or processed transcript genes derived from GENCODE. The 65 genes were incorporated into Ingenuity Canonical pathway enrichment analysis. The *P* values are calculated based on Fisher's right-tailed exact test. The default settings were used for Ingenuity Interaction network analysis.

*Gene relationships among implicated loci*. The GRAIL was used to examine relationships between genes. For each query and seed SNP, we adopted the default methods implemented in GRAIL to extract the genes around each index SNP: that is, (1) we first identified neighboring SNPs in the 3′ and 5′ direction in LD (*r*^2^>0.5, CEU HapMap), proceeding outwards in each direction to the nearest recombination hotspots to define an interval region, and extracted all the genes in this interval; (2) if there are no genes in that interval region, the interval is extended an additional 250 kb in either direction. The 12 GWS-index SNP regions were input as seed regions, and the regions for the remaining 31 SNPs were input as query regions. Connections between genes were inferred from textual relationships based on published scientific text using PubMed abstracts as of December 2006. The significant gene similarity was declared based on *P*_GRAIL_<0.01.

*Disease association protein–protein link evaluator*. The DAPPLE package was used to examine the potential encoded protein–protein interaction evidence for the genes located in the 43 associated loci. Genes from 32 of the 43 loci were annotated in the high-confidence pair-wise interaction InWeb database. Both the direct and indirect interactions were considered. The running settings were 1,000 permutation, common interactor binding degree =2, and 110 kb upstream and 40 kb downstream to define a gene' residence.

### *Drosophila* knockdown experiments

*Genome-wide screen*. We first identified all genes within ±500 kb of the 12 GWS-index SNPs, and subsequently identified the corresponding *Drosophila* orthologues available in the ensembl orthologue database ( www.ensembl.org, Supplementary Table 23). *Drosophila* triglyceride content values were mined from a publicly available genome-wide obesity screen data set^52^. Estimated values represent fractional changes in triglyceride content in adult male flies. Data are from male progeny resulting from crosses of male UAS-RNAi flies from the VDRC and Hsp70-GAL4; Tub-GAL8ts virgins females. Two-to-five-day-old males were sorted into groups of 20 and subjected to two 1-h wet heatshocks 4 days apart. On the seventh day, flies were picked in groups of eight, manually crushed and sonicated, and the lysates heat-inactivated for 10 min in a thermocycler at 95 °C. Centrifuge-cleared supernatants were then used for triglyceride (GPO Trinder, Sigma) and protein (Pierce) determination. Triglyceride values from these adult-induced ubiquitous RNAi knockdown individuals were normalized to those obtained in parallel from non-heatshocked progeny from the very same crosses.

*Targeted follow-up*. Based on known biology, one to three potential candidate genes within ±500 kb of the 12 GWS-index SNPs were selected. Corresponding *Drosophila* orthologues were available for 11 loci, but no orthologue exists for *FTO* (Supplementary Table 24, http://www.flyrnai.org/cgi-bin/DRSC_orthologs.pl). The respective fly RNAi stocks for each *Drosophila* orthologue were acquired from the Vienna *Drosophila* Resource Center, as well as genetic background controls w1118 (for GD lines, VDRC #60000); tub-gal4/TM6 and w; tub-gal80ts/TM6 is available from the Bloomington *Drosophila* Stock Center. For fly triglyceride assay in the adult, male RNAi flies were crossed with w; tub-gal4 tub-gal80ts/TM6 virgins. Progenies were kept in 16 °C until enclosure. Adults were transferred to 25 °C for 2 weeks. Whole-animal triglycerides were measured as previously described^53^. Briefly, triglycerides were measured using the Infinity Triglycerides Reagent kit (Thermo Fisher #TR22321) on whole-animal homogenates of groups of three animals. Proteins from the same homogenates were measured using the Pierce BCA protein Assay kit (Thermo Scientific #23227). Triglycerides were normalized by proteins. Data were average of three experiments. The fractional changes in triglyceride content in adult male flies between knockdown group and the control groups were compared using the two-tailed *t*-tests in SAS version 9.2 software (SAS Institute, Cary, NC).

## Additional information

**How to cite this article:** Lu, Y. *et al.* New loci for body fat percentage reveal link between adiposity and cardiometabolic disease risk. *Nat. Commun.* 7:10495 doi: 10.1038/ncomms10495 (2016).

## Supplementary Material

Supplementary Information Supplementary Figures 1-8, Supplementary Tables 1-26 and Supplementary Notes 1-3.

## Figures and Tables

**Table 1:**
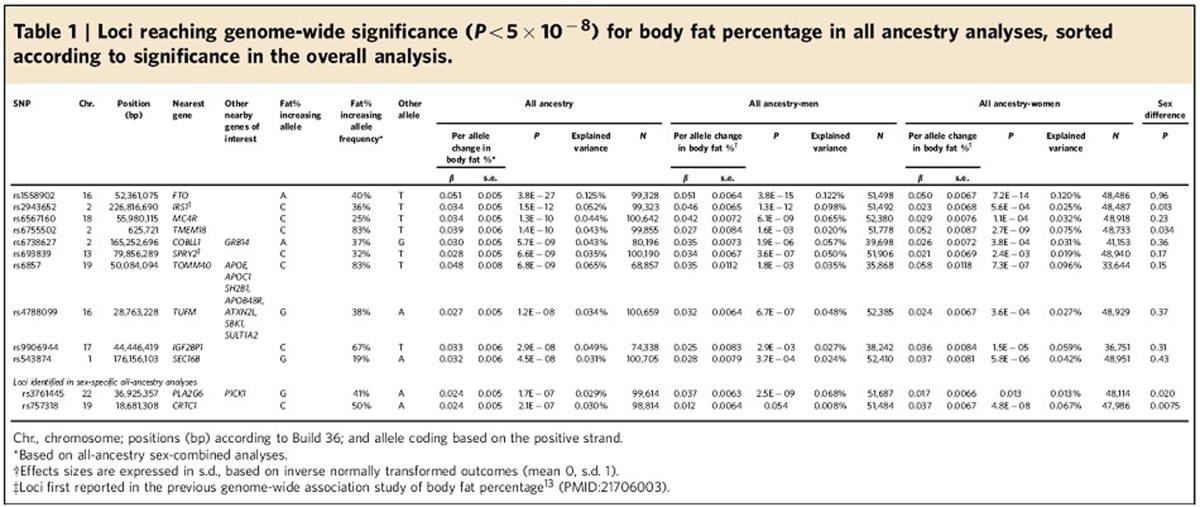
Loci reaching genome-wide significance (*P*<5 × 10^−8^) for body fat percentage in all ancestry analyses, sorted according to significance in the overall analysis.

**Table 2:**
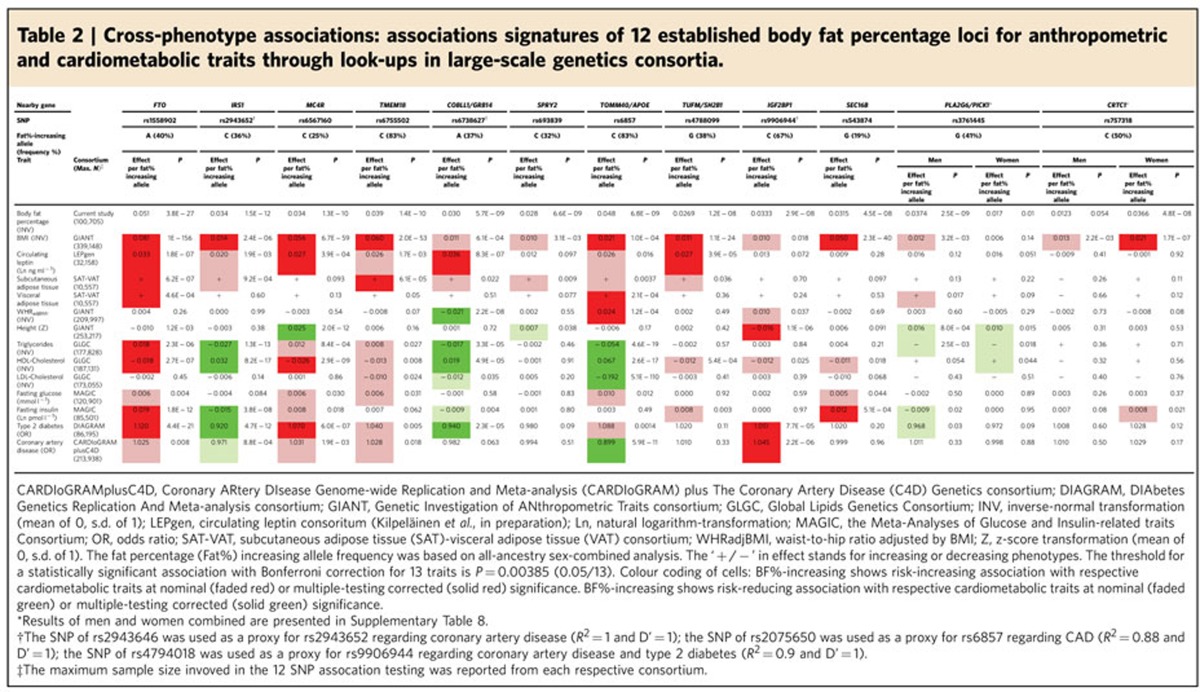
Cross-phenotype associations: associations signatures of 12 established body fat percentage loci for anthropometric and cardiometabolic traits through look-ups in large-scale genetics consortia.
