# Supplementary material for: New loci for body fat percentage reveal link between adiposity and cardiometabolic disease risk
Source: Nat Commun. 2016 Feb 1;7:10495. doi: 10.1038/ncomms10495 (PMC4740398; doi:10.1038/ncomms10495)
Supplement: Supplementary Information — Supplementary Figures 1-8, Supplementary Tables 1-26 and Supplementary Notes 1-3. [file ncomms10495-s1.pdf]

Supplementary Figures

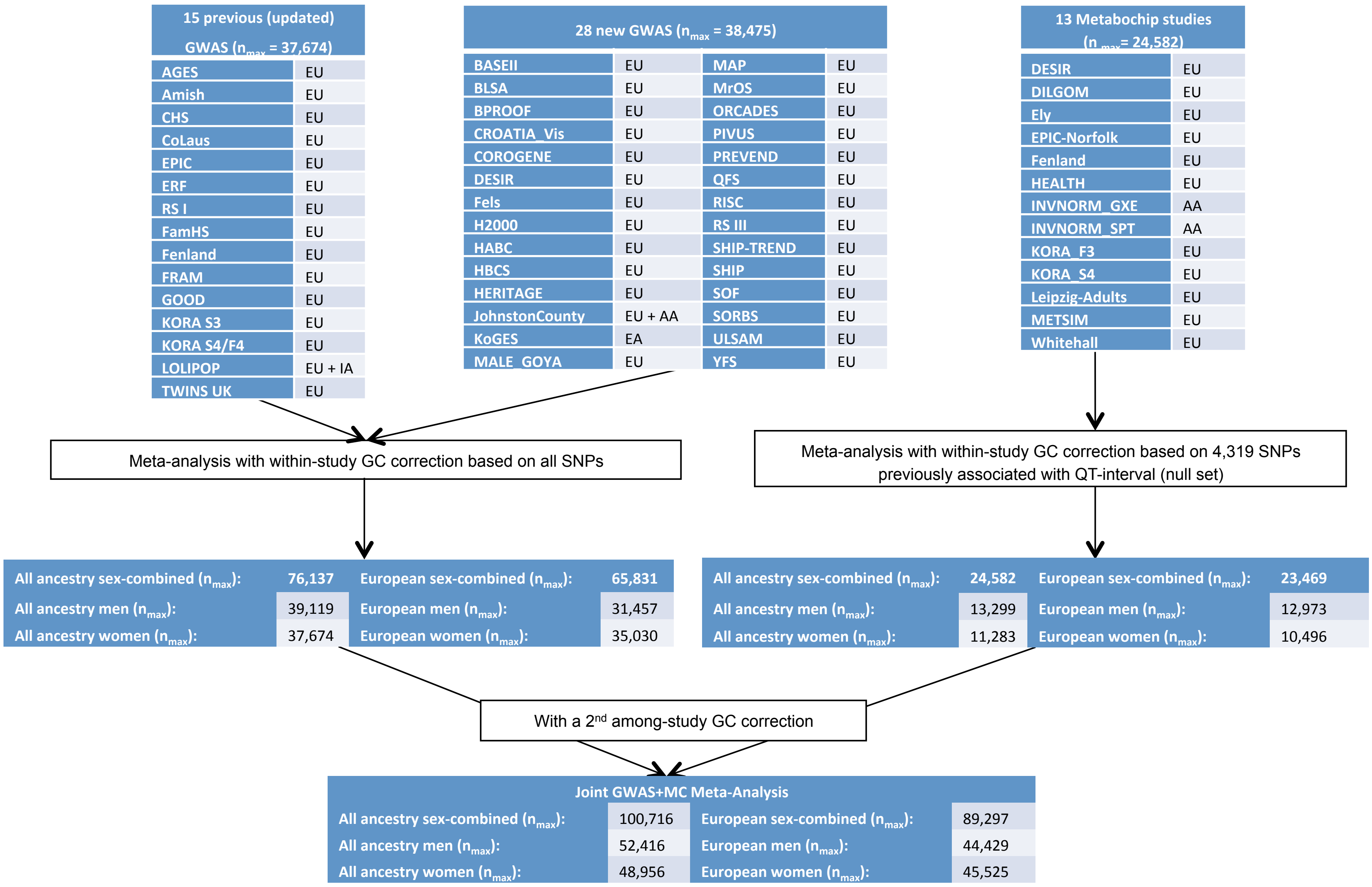

Supplementary Figure 1. Study design and participating cohorts. Counts represented the maximum number in the respective strata of analysis. EU: European or European American, IA: Indian Asian, AA: African American, EA: Eastern Asian.

A

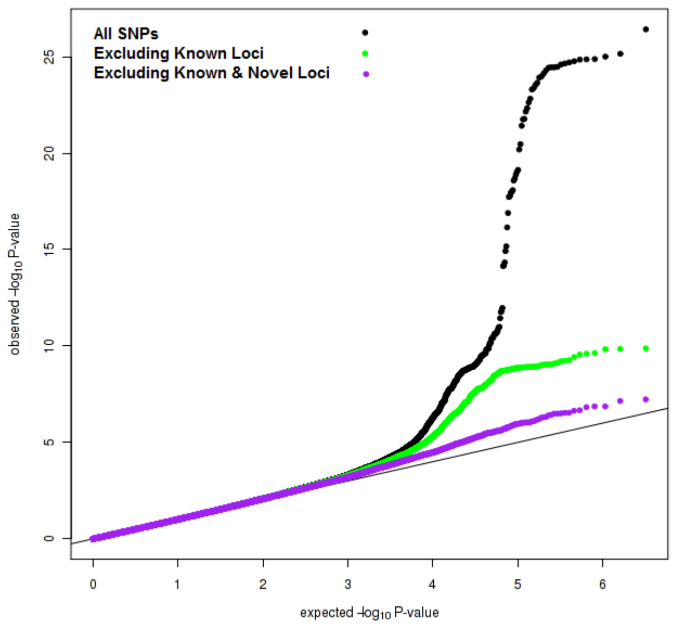

B

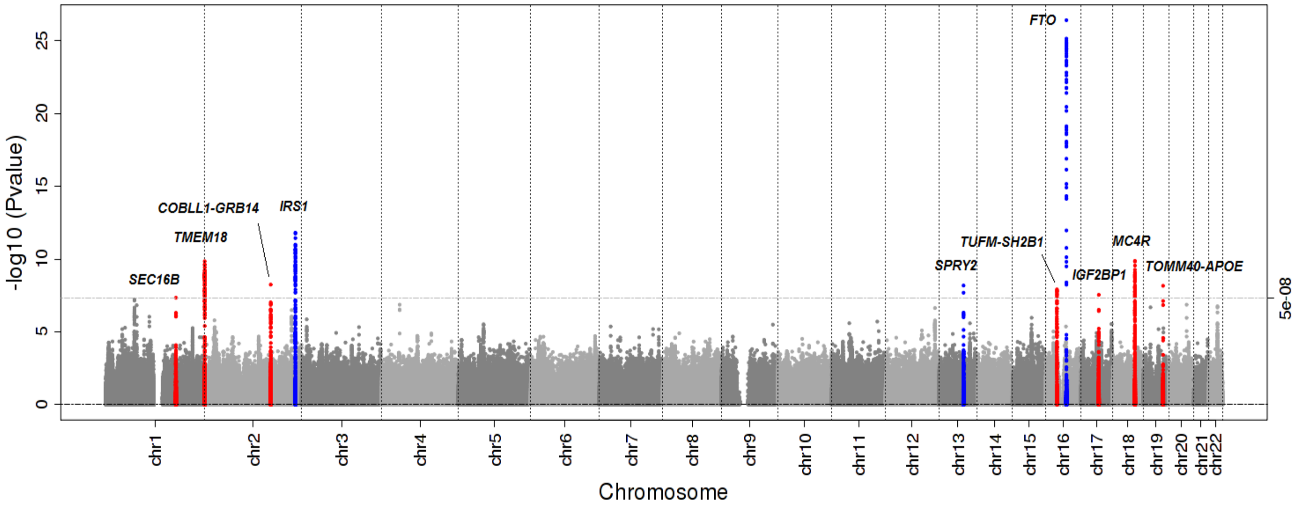

Supplemental Figure 2. Summary plots of all-ancestry sex-combined body fat percentage meta-analysis. A. Quantile-quantile plot of SNP associations. All SNPs were plotted in black; after removing all SNPs within 500 kb of the previously reported loci, the remaining SNPs were plotted in green; and after removing all SNPs within 500 kb of the previously reported loci and also novel loci, the remaining SNPs were plotted in purple. The genomic control value (λGC) was 1.002. The uniform null distribution was marked with a solid black line. B. Manhattan plot of association statistics (-log10(*P* values)) showing previously reported loci in blue and novel loci in red.

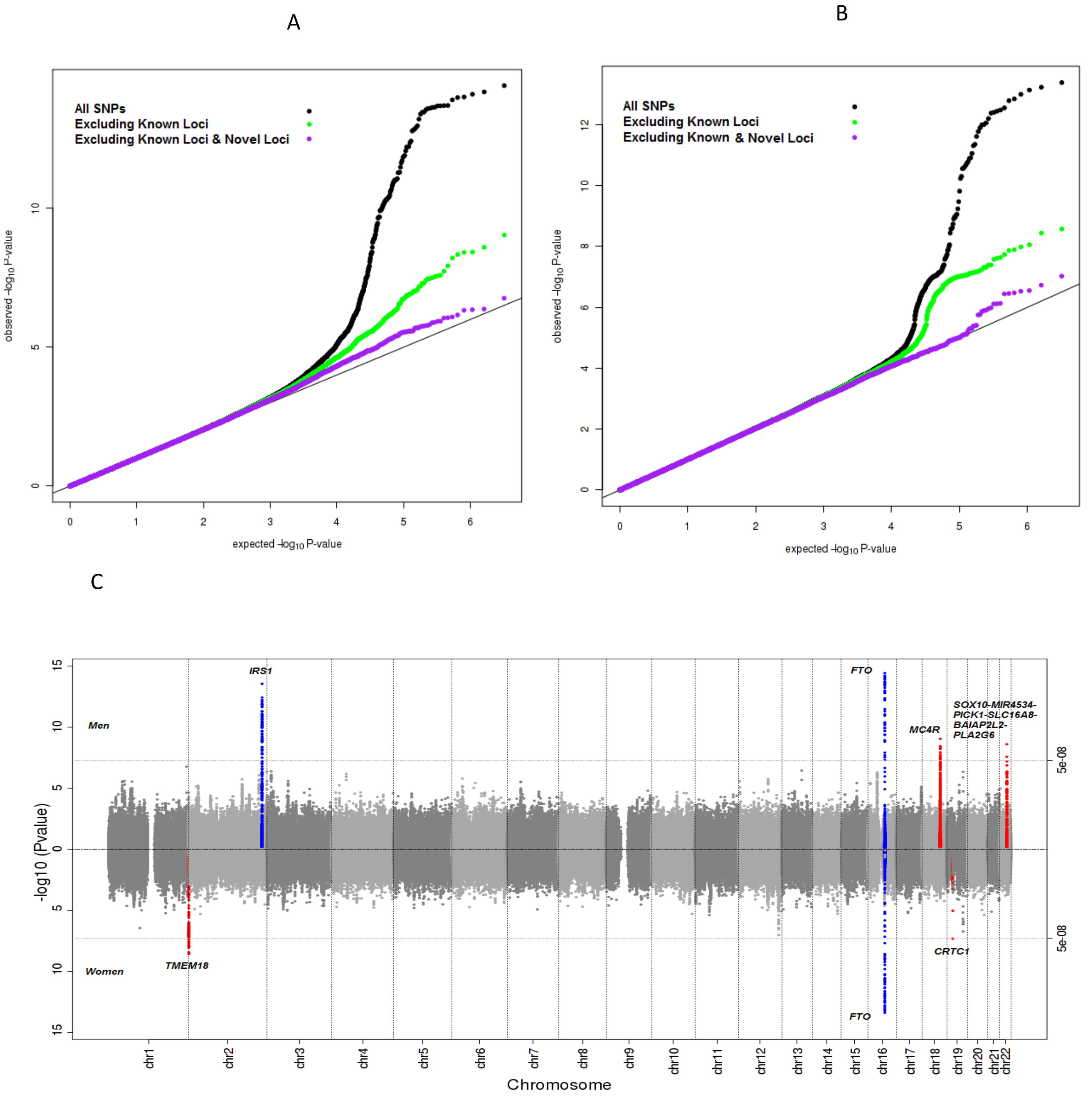

Supplemental Figure 3. Summary plots of all-ancestry sex-specific body fat percentage meta-analysis. A. Quantile-quantile plot of SNP associations for men-specific meta-analysis (λGC = 1.002). B. Quantile-quantile plot of SNP associations for women-specific meta-analysis (λGC = 1.001). All SNPs were plotted in black; after removing all SNPs within 500 kb of the previously reported loci, the remaining SNPs were plotted in green; and after removing all SNPs within 500 kb of the previously reported loci and also novel loci, the remaining SNPs were plotted in purple. The uniform null distribution was marked with a solid black line. C. Manhattan plot of association statistics (-log10(*P* values)) for men and women. Previously reported loci were in blue, and novel associations identified in the sex-specific analysis were in red.

A

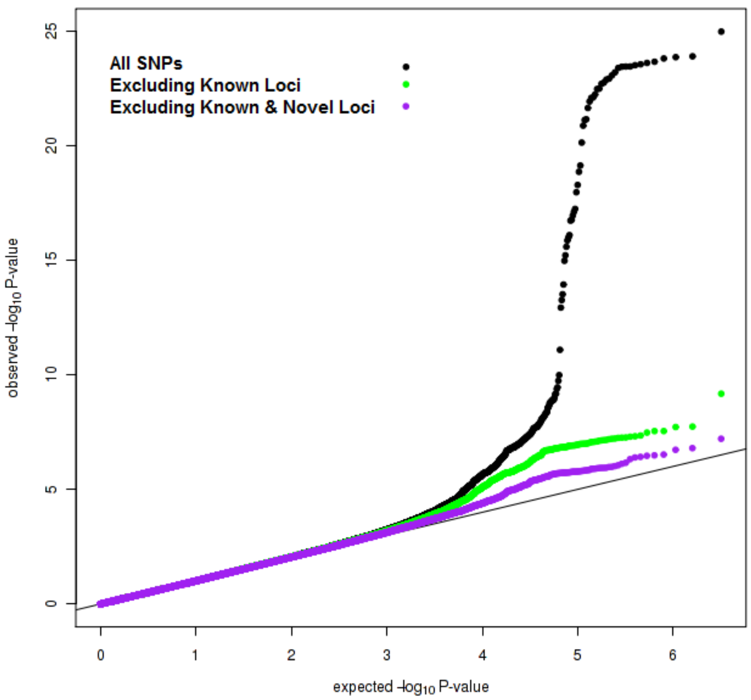

B

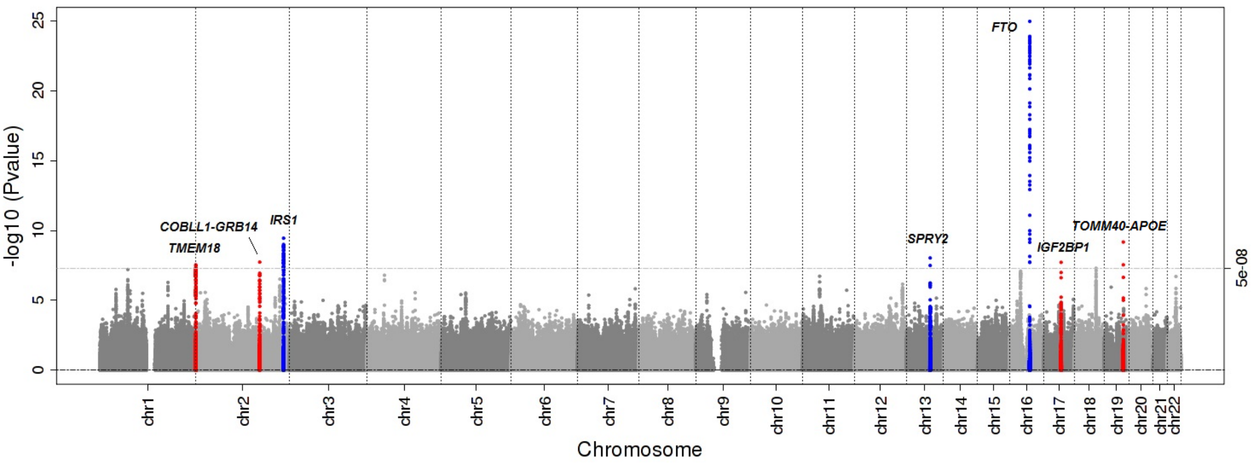

Supplemental Figure 4. Summary plots of European sex-combined body fat percentage meta-analysis. A. Quantile-quantile plot of SNP associations. All SNPs were plotted in black; after removing all SNPs within 500 kb of the previously reported loci, the remaining SNPs were plotted in green; and after removing all SNPs within 500 kb of the previously reported loci and also novel loci, the remaining SNPs were plotted in purple. The genomic control value (λGC) was 1.006. The uniform null distribution was marked with a solid black line. B. Manhattan plot of association statistics (-log10(*P* values)) showing previously reported loci in blue and novel loci in red.

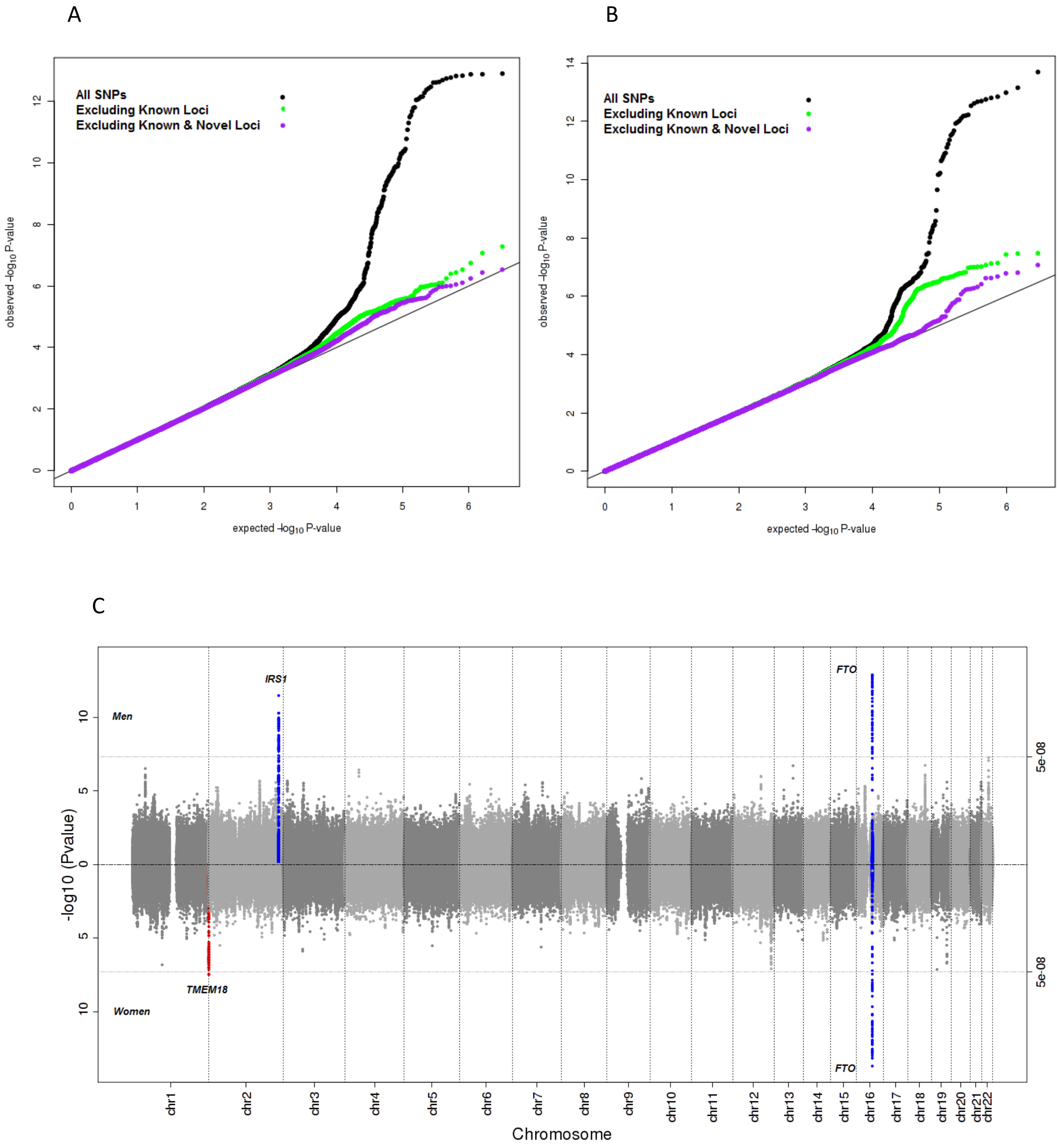

Supplemental Figure 5. Summary plots of European sex-specific body fat percentage meta-analysis. A. Quantile-quantile plot of SNP associations for men-specific meta-analysis (λGC = 1.001). B. Quantile-quantile plot of SNP associations for women-specific meta-analysis (λGC = 1.003). All SNPs were plotted in black; after removing all SNPs within 500 kb of the previously reported loci, the remaining SNPs were plotted in green; and after removing all SNPs within 500 kb of the previously reported loci and also novel loci, the remaining SNPs were plotted in purple. The uniform null distribution was marked with a solid black line. C. Manhattan plot of association statistics (-log10(*P* values)) for men and women. Previously reported loci were in blue, and novel associations identified in the sex-specific analysis were in red.

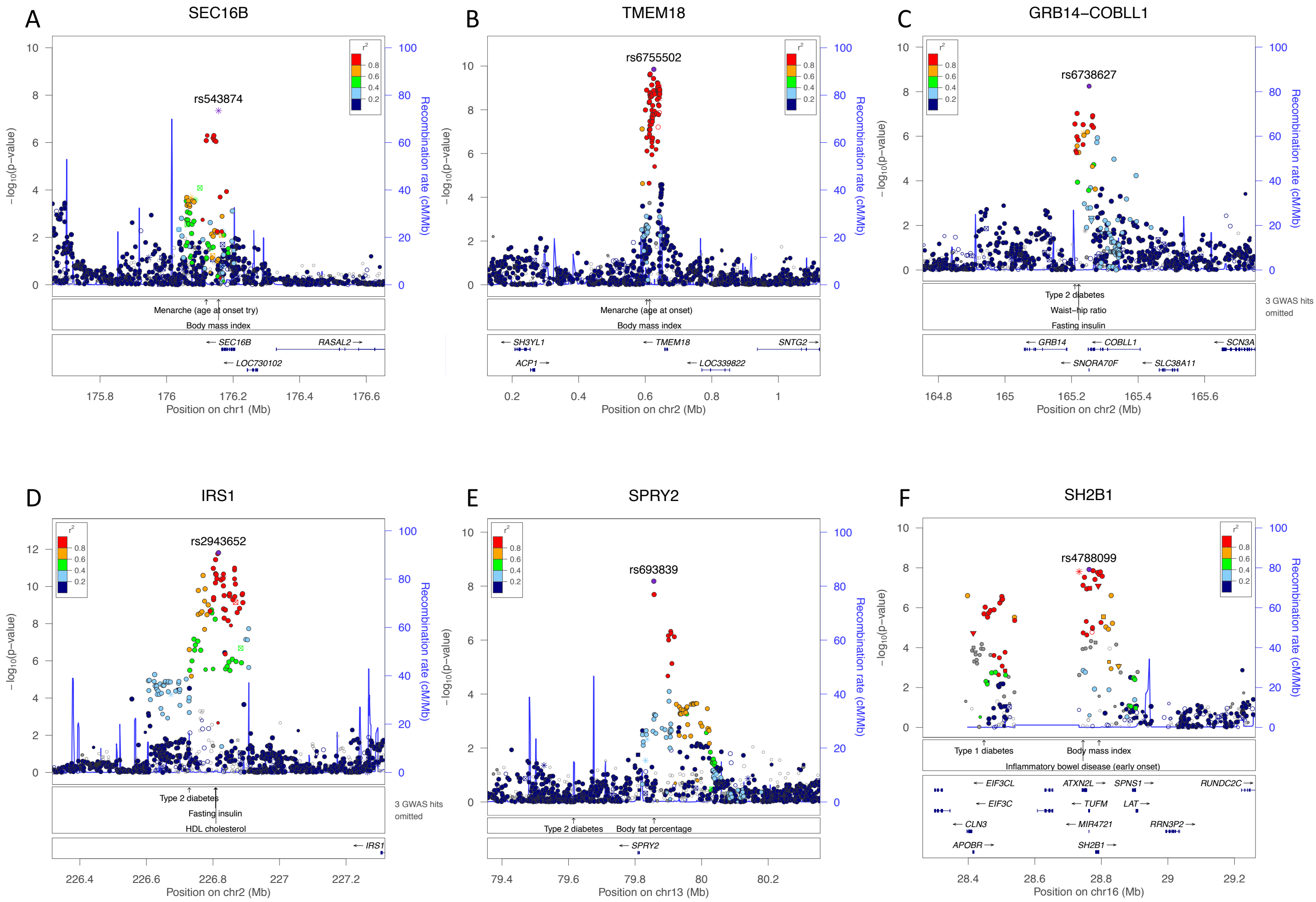

Supplemental Figure 6. Regional plots of the 12 identified loci that reached genome-wide significant association with body fat percentage in all-ancestry analyses (sex-combined: A-I, K; in men for the PLA2G6 loci: L; and in women for the CRTC1 loci: J). Each symbol represents the significance (*P* value on a −log10 scale) of a SNP with body fat percentage as a function of the SNP's genomic position (NCBI Build 36). For each locus, the index SNP is represented in the purple color. The color of all other SNPs indicates LD with the index SNP (estimated by CEU *r2* from the HapMap Project data Phase II CEU). Recombination rates are also estimated from International HapMap Project data, and gene annotations are obtained from the UCSC Genome Browser. GWAS catalogs SNPs with *P* value < 5×10-8 are shown in the middle panel. Different shapes denote the different categories of the SNPs: up-triangle for framestop or splice SNPs, down-triangle for nonsynonymous SNPs, square for coding or UTR SNPs; star for SNPs in tfbscons region, square filled with "X" symbol for SNPs located in mcs44placental region, and circle for SNPs with no annotation information.

Supplementary Figure 6 (Continued)

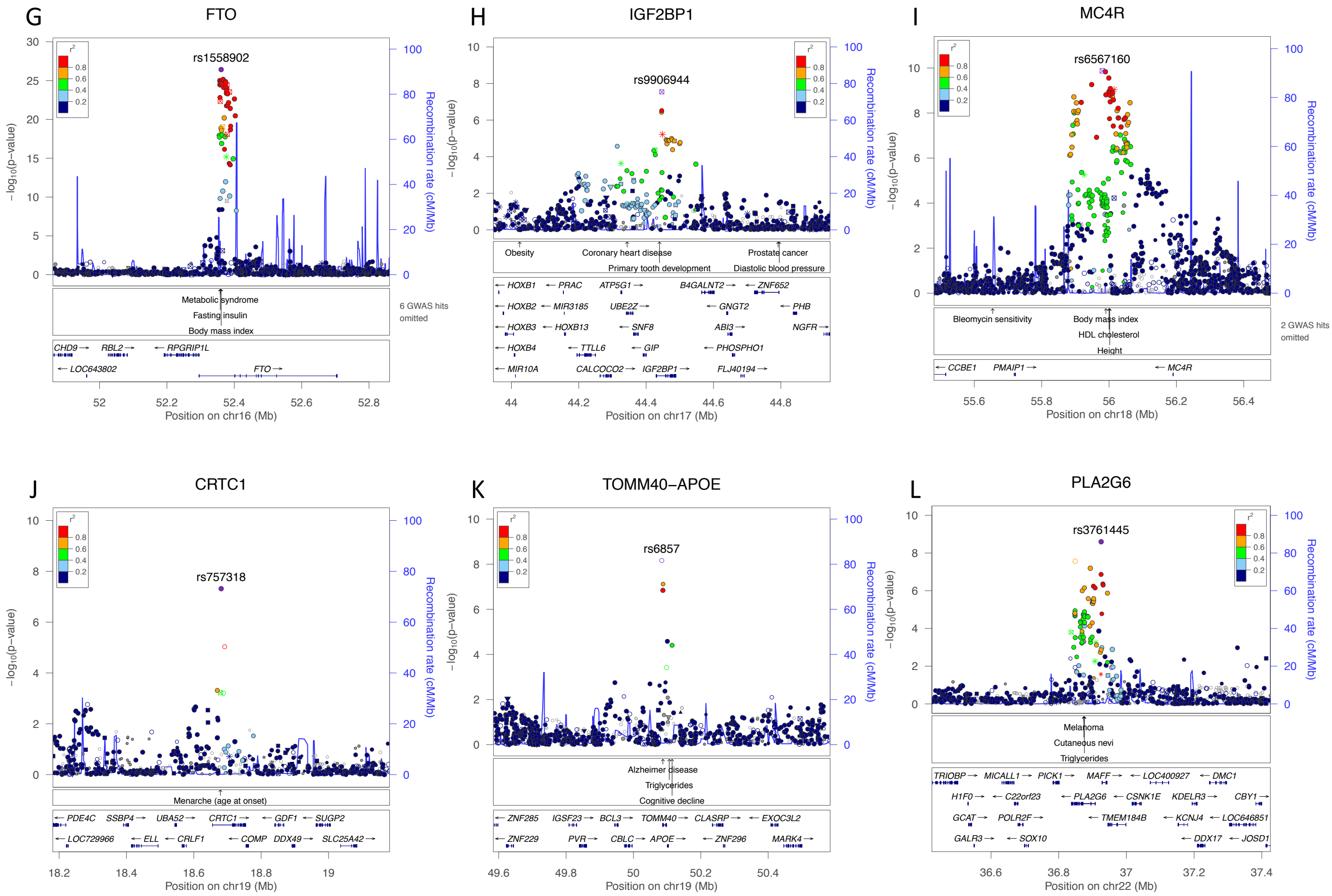

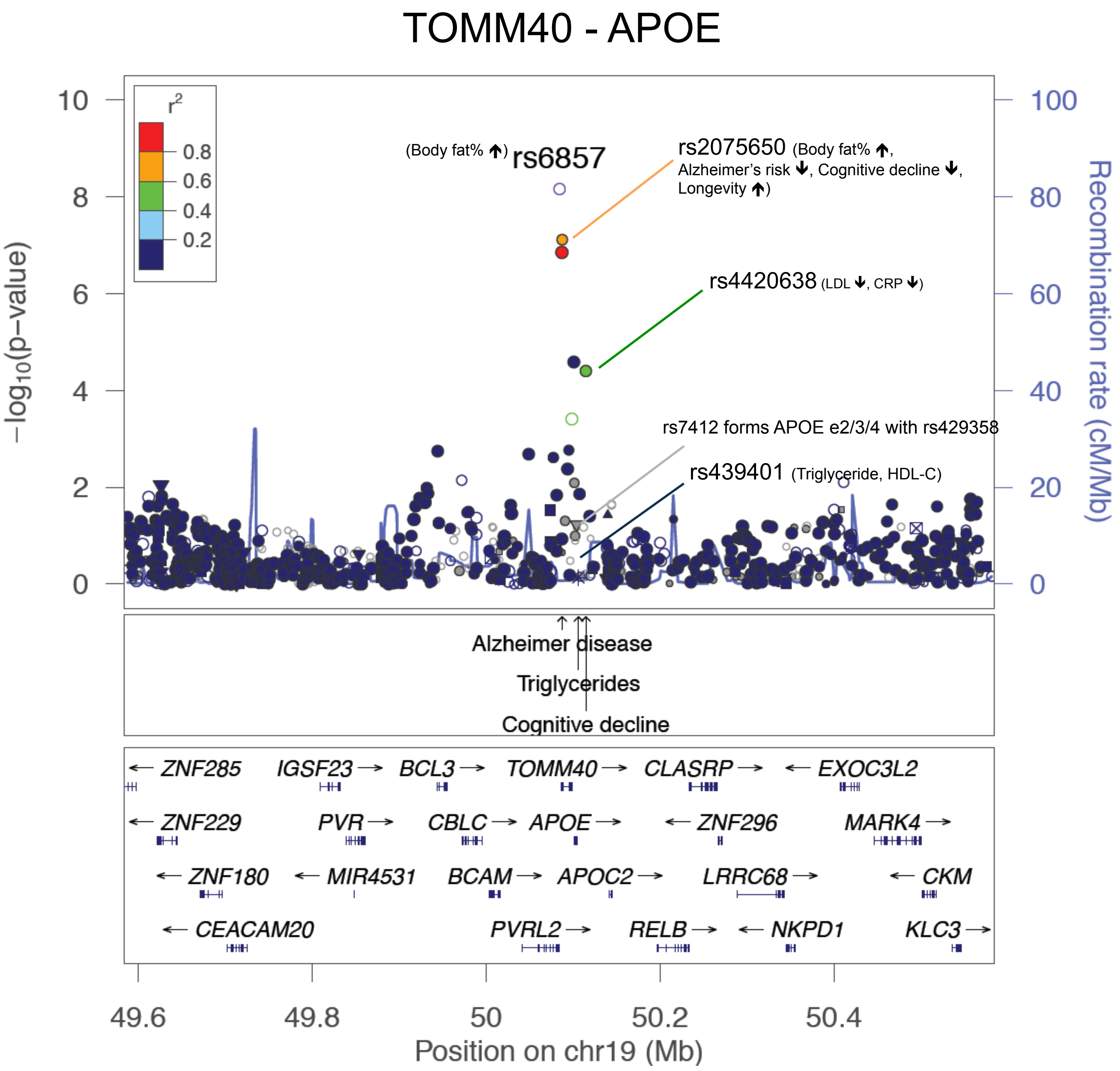

Supplemental Figure 7. Regional plots of the TOMM40-APOE loci that reached genome-wide significant association with body fat percentage in all-ancestry sex-combined analyses. Each symbol represents the significance (*P* value on a −log10 scale) of a SNP with body fat percentage as a function of the SNP's genomic position (NCBI Build 36). For each locus, the index SNP is represented in the purple color. The color of all other SNPs indicates LD with the index SNP (estimated by CEU *r2* from the HapMap Project data Phase II CEU). Recombination rates are also estimated from International HapMap Project data, and gene annotations are obtained from the UCSC Genome Browser. The known SNPs with genome-wide significant associations with different traits are also shown in the upper panel. GWAS catalogs SNPs with *P* value < 5×10-8 are shown in the middle panel. Different shapes denote the different categories of the SNPs: up-triangle for framestop or splice SNPs, down-triangle for nonsynonymous SNPs, square for coding or UTR SNPs; star for SNPs in tfbscons region, square filled with "X" symbol for SNPs located in mcs44placental region, and circle for SNPs with no annotation information.

Motifs Filter:

Method	Location	Motif	? Cell Type	PWM	Reference
Footprinting	chr22:38572437..38572459	HEN1	Melano	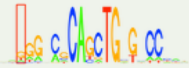	21106904
Footprinting	chr22:38572437..38572459	HEN1	Panisd	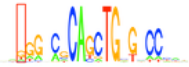	21106904
Footprinting	chr22:38572437..38572459	HEN1	Huvec	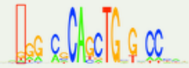	21106904
Footprinting	chr22:38572437..38572459	HEN1	Hsmmt	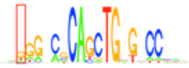	21106904
Footprinting	chr22:38572437..38572459	HEN1	Hsmm	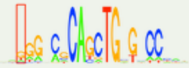	21106904
Footprinting	chr22:38572437..38572459	HEN1	Hmec	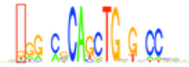	21106904
Footprinting	chr22:38572437..38572459	HEN1	Gm18507	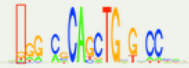	21106904
Footprinting	chr22:38572437..38572459	HEN1	AosmcSerumfree	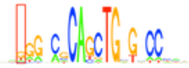	21106904
Footprinting	chr22:38572437..38572459	HEN1	Fibrop	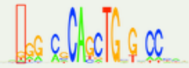	21106904
Footprinting	chr22:38572437..38572459	HEN1	Gm12891	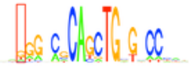	21106904
Footprinting	chr22:38572437..38572459	HEN1	Gm12892	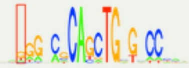	21106904
PWM	chr22:38572437..38572459	HEN1		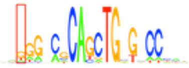	16381825

Supplemental Figure 8. HEN1 binding motif at rs4383. RegulomeDB was searched for SNPs in high LD (*r2* > 0.7, 1000 Genomes Project) with rs3761445 (*PLA2G6* region). The SNP of rs4843 is located in a HEN binding motif and DNaseI footprints in multiple cell types.

Supplementary Table 1. Study design, number of individuals and sample quality control for genome-wide association studies and metabochip studies

Study	Full name	Study design	Ethnicity	Total sample size (N)	Sample QC: Call rate*	Sample QC: Other exclusions	Samples in analyses (N)	Fat % assessment	Instrument	References
Genome-wide Association Studies										
AGES	Age, Gene/Environment Susceptibility-	Population-based	European descent	3,219	≥ 97%	1) mismatch with previous genotypes<br>2) remove A/T & G/C SNPs<br>3) remove SNPs not in HapMap	2,375	Bioimpedance	A Xitron HYDRA ECF/ICF, Model 4200	[PMID: 17351290]<br>Harris T, et al. Age, Gene/Environment Susceptibility-Reykjavik Study: multidisciplinary applied phenomics. American Journal of Epidemiology 165, 1076–1087 (2007).
Amish	The Old Order Amish	Founder population	European descent	1,186	≥ 96%	1) failed Mendelian check<br>2) failed duplicate check<br>3) missing body fat percentage data	850	DEXA	Hologic QDR-4500W	[PMID: PMC2443415]<br>Mitchell BD, et al. The genetic response to short-term interventions affecting cardiovascular function: Rationale and design of the Heredity and Phenotype Intervention (HAPI) Heart Study. Am Heart J 823, 828 (2008).
BASE-II	Berlin Aging Study II	Population-based	European descent	2,085	≥ 90%	1) heterozygosity <20% or >34%;<br>2) gender mismatches;<br>3) ethnic outliers;<br>4) related individuals and duplicates;<br>5) Missing body weight and height.	624	DEXA	Hologic QDR Discovery	[PMID: 23505255]<br>Bertram L. et al. Cohort Profile: The Berlin Aging Study II (BASE-II). Int J Epidemiol. 2014;43:703-12.
BLSA	Baltimore Longitudinal Study of Aging	Population-based	European descent	1,230	≥ 98.5	1) ethnic outliers;<br>2) Missing body weight and height.<br>3) Sex mismatch	844	DEXA	GE Lunar Prodixy ADVANCE	Shock, N.W., et al., Normal Human Aging: The Baltimore Study of Aging. 1984.: NIH Publication No. 84-2450 (http://health-equity.pitt.edu/2557/)
B-PROOF	B-vitamins for the prevention of osteoporotic fractures	RCT	European descent	2,891	>97.5%	1) Excess heterozygosity (F-values smaller than the mean–(4*SD))<br>2) Gender mismatch<br>3)Non-Caucasian<br>4) Familiar relationships 5) No body composition data available	1,075	DEXA	GE Lunar Prodigy/Hologic Delphi	[PMID:22136481]<br>Rationale and design of the B-PROOF study, a randomized controlled trial on the effect of supplemental intake of vitamin B12 and folic acid on fracture incidence. van Wijngaarden JP, Dhonukshe-Rutten RA, van Schoor NM, van der Velde N, Swart KM, Enneman AW, van Dijk SC, Brouwer-Brolsma EM, Zillikens MC, van Meurs JB, Brug J, Uitterlinden AG, Lips P, de Groot LC. BMC Geriatr. 2011;11:80.
CHS	Cardiovascular Health Study	Population-based cohort st	Caucasian	4925	>95%	1) prevalent clinical CVD<br>2) sex mismatch<br>3) discordance with prior genotyping<br>4) no DEXA scan done	921	DEXA	Hologic QDR-2000 Bone densitometer	[PMID: 11454111]<br>Robbins J, et al. The association of bone mineral density and depression in an older population. J Am Geriatr Soc 49, 732-736 (2001).<br>[PMID: 1669507]<br>Fried LP, et al. The Cardiovascular Health Study: design and rationale. Ann Epidemiol 1, 263-276 (1991).
CoLaus	Cohort Lausannoise	Population-based	European descent	6,188	≥ 90%	1) ethnic outliers<br>2) related individuals and duplicates<br>3) missing body fat percentage data	5,389	Bioimpedance	Bodystat 1500 Analyzer	[PMID: 18366642]<br>Firmann M, et al. The CoLaus study: a population-based study to investigate the epidemiology and genetic determinants of cardiovascular risk factors and metabolic syndrome. BMC Cardiovasc Dis, 8, 6 (2008).
COROGENE CONTROLS	COROGENE-STUDY Genetic Predisposition of Coronary Heart Disease in Patients Verified with Coronary Angiogram. CONTROLS (The FINRISK study). *Corogene controls are picked from the National	Population-based	European descent	2,051	≥ 95%	1) heterozygosity, gender check and relatedness checks have been performed and any discrepanies have been removed.<br>2) Missing phenotype.	641	Bioimpedance	TANITA TBF-300MA	Peltonen, M., Harald, K., Männistö, S., Saarikoski, L., Peltomäki, P., Lund, L., et al. (2008). The National FINRISK 2007 Study. Helsinki: Publications of the National Public Health Institute, B 34/2008.
CROATIA-VIS	The CROATIA study, Vis Island cohort	Population-based	European descent	924	97%	ethnic outliers, people with missing phenotype data	903	Bioimpedance	Akern bioresearch soft tissue analyzer - STA BIA (Florence, Italy)	[PMID:18327257]<br>Vitart V. et al. SLC2A9 is a newly identified urate transporter influencing serum urate concentration, urate excretion and gout.Nat Genet. 2008;40:437-42
DESIR	Data from an Epidemiological Study on the Insulin Resistance syndrome	Population-based	white European	731	≥ 90%	1) Missing data;<br>2) Ethnic outliers (N=15).	628	Bioimpedance	TBF 300P balance from Tanita (Neuilly-sur-Seine, France)	[PMID: 8927780]<br>Balkau B (1996) An epidemiologic survey from a network of French Health Examination Centres, (D.E.S.I.R.): epidemiologic data on the insulin resistance syndrome, Rev Epidemiol Sante Publique 44(4):373-5.
EPIC-Obesity Study	European Prospective Investigation into Cancer and Nutrition - Obesity Study	Population-based	European descent	2,566	≥ 94%	1) heterozygosity <23% or >30%;<br>2) >5.0% discordance in SNP pairs with r2= 1 in HapMap;<br>3) ethnic outliers;<br>4) related individuals and duplicates;<br>5) Missing body weight and height.	2,543	Bioimpedance	Tanita BC-531 Body Composition Monitor	[PMID: 10466767]<br>Day, N.E. et al. EPIC-Norfolk: study design and characteristics of the cohort. European Prospective Investigation of Cancer. British Journal of Cancer 80, 95-103 (1999).<br>[PMID: 18454148]<br>Loos, R.J. et al. Common variants near MC4R are associated with fat mass, weight and risk of obesity. Nat Genet 40, 768-775 (2008).
ERF	Erasmus Rucphen Family Study	Family-based	European descent	2,315	≥98%	1) gender mismatch;<br>2) ethnic outliers;<br>3) missing body fat percentage data.	2,087	DEXA	GE Lunar Prodigy	[PMID:15054401]<br>Aulchenko YS, Heutink P, Mackay I, Bertoli-Avella AM, Pullen J, et al. Linkage disequilibrium in young genetically isolated Dutch population. Eur J Hum Genet 12: 527-534 (2004).
Fels	Fels Longitudinal Study	Family-based	European descent	1,304	≥ 90%	1) < 1 heterozygote<br>2) monomorphic after imputation<br>3) ethnic outliers<br>4) missing DEXA data	916	DEXA	Hologic Discovery A	Roche AF. Growth, maturation, and body composition : the Fels Longitudinal Study, 1929-1991. Cambridge ; New York, NY: Cambridge University Press; 1992.
FamHS	Family Heart Study	Population-based	white European	2,337	≥ 98%	1) technical errors;<br>2) discrepancies between reported sex and sex-diagnostic markers;<br>3) SNPs were deleted if Mendelian errors.	2,337	Bioimpedance	Weight Manager Version 2.05a	[PMID: 8651220]<br>Higgins M, et.al. NHLBI Family Heart Study: objectives and design. Am J Epidemiol. 143,1219-28 (1996).
Fenland	Fenland Stud	Population-based	European descent	1,500	≥ 95%	1) failed heterozygosity check: upperbound 0.2882, lowerbound 0.2735;<br>2) failed relatedness check (sample with lower call rate in related samples);<br>3) failed duplicate check (sample with lower call rate in duplicates);<br>4) missing body fat percentage data.	1,402	DEXA	GE Lunar Prodigy	[PMID: 20519560]<br>De Lucia Rolfe, E. et al. Association between birth weight and visceral fat in adults. Am J Clin Nutr. 2010;92:347-52..<br>[PMID: 19079261]<br>Willer CJ, Speliotes EK, Loos RJ, et al. Siz new loci associated with body mass index highlight a neuronal influence on body weight regulation. Nat Genet 41, 25-34 (2009).
Framingham	Framingham Heart Study	Population-based, multi-generational	European descent	3,780	≥ 97%	1) autosomal heterozygosity <0.33 or >0.37<br>2) ethnic outliers (using Eigenstrata)<br>3) missing body fat percentage data	2,748	DEXA	GE Lunar DPX-L	[PMID: 10466767]<br>Visser, M . et al. Body fat and skeletal muscle mass in relation to physical disability in very old men and women of the Framingham Heart Study. J Gerontol 53A, M214-M221 (1998).

GOYA	Males from Genetics of Overweight Young Adults Study	Case Cohort	European descent	1,714	≥ 94%	NA	1,714	Bioimpedance	BIA-103 RJL-system-analyser (RJL-system Detroit)	[PMID: 21935397] Paternoster L, Evans DM, Nohr EA, Holst C, Gaborieau V, Brennan P, Gjesing AP, Grarup N, Witte DR, Jørgensen T, Linneberg A, Lauritzen T, Sandbaek A, Hansen T, Pedersen O, Elliott KS, Kemp JP, St Pourcain B, McMahon G, Zelenika D, Hager J, Lathrop M, Timpson NJ, Smith GD, Sørensen TI. Genome-wide population-based association study of extremely overweight young adults--the GOYA study. PLoS One. 6; 9:e24303 (2011)
GOOD	Gothenburg Osteoporosis and Obesity Determinants Study	Population-based	European descent	1,056	≥ 97.5%	1) heterozygosity >33%<br>2) ethnic outliers<br>3) related individuals and duplicates	940	DEXA	GE Lunar Prodigy	[PMID: 16007330] Lorentzon, M. et al Free testosterone is a positive whereas free estradiol is a negative predictor of cortical bone size in young Swedish men-The GOOD Study. J Bone Miner Res 20, 1334-1341 (2005).
H2000 CASES	Health 2000 cases	Case-control	European descent	1,022	≥ 95%	1) heterozygosity, gender check and relatedness checks have been performed and any discrepansies have been removed;<br>2) Missing phenotype.	934	Bioimpedance	InBody 3.0	http://www.terveys2000.fi/indexe.html HEALTH AND FUNCTIONAL CAPACITY IN FINLAND, Baseline Results of the Health 2000 Health Examination Survey. In: Aromaa A, Koskinen S, eds. Vol KTL B12/2004. Helsinki: National Public Health Institute; 2004. Available in http://www.terveys2000.fi/julkaisut/baseline.pdf.<br>[PMID:20673644] Pajunen P, Rissanen H, Härkänen T, Jula A, Reunanen A, Salomaa V. The metabolic syndrome as a predictor of incident diabetes and cardiovascular events in the Health 2000 Study. Diabetes Metab. 2010;36:395-401.
H2000 CTRLS	Health 2000 controls	Case-control	European descent	1,081	≥ 95%	1) heterozygosity, gender check and relatedness checks have been performed and any discrepansies have been removed.<br>2) Missing phenotype.	974	Bioimpedance	InBody 3.0	http://www.terveys2000.fi/indexe.html HEALTH AND FUNCTIONAL CAPACITY IN FINLAND, Baseline Results of the Health 2000 Health Examination Survey. In: Aromaa A, Koskinen S, eds. Vol KTL B12/2004. Helsinki: National Public Health Institute; 2004. Available in http://www.terveys2000.fi/julkaisut/baseline.pdf.<br>[PMID:20673644] Pajunen P, Rissanen H, Härkänen T, Jula A, Reunanen A, Salomaa V. The metabolic syndrome as a predictor of incident diabetes and cardiovascular events in the Health 2000 Study. Diabetes Metab. 2010;36:395-401.
Health ABC	Health, Aging, and Body Composition Study	Longitudinal cohort study	European descent	1,655	≥ 97%	1) related individuals and duplicates;<br>2) Missing body weight , height, or DEXA.	1,509	DEXA	Hologic QDR-4500	[PMID: 10865790] Harris, T.B. et al. Waist circumference and sagittal diameter reflect total body fat better than visceral fat in older men and women. The Health, Aging and Body Composition Study. Ann N Y Acad Sci. 2000;904:462-73.
HBCS	Helsinki Birth Cohort Study	Birth-cohort	European descent	1,872	≥ 95%	1) heterozygosity, gender check and relatedness checks have been performed and any discrepansies have been removed;<br>2) Missing phenotype.	1,556	Bioimpedance	InBody 3.0 eight-polar tactile electrode system, Biospace Co, Ltd, Seoul, Korea	[PMID: 17356523] Ylihärsilä H, Kajantie E, Osmond C, Forsén T, Barker DJ, Eriksson JG. Birth size, adult body composition and muscle strength in later life. Int J Obes (Lond). 2007 Sep;31(9):1392-9.
HERITAGE Family Study	Health, Risk Factors, Training and Genetics (HERITAGE) Family Study	Family Study, baseline data from an exercise training intervention	European descent	500	>99%	1) gender mismatch<br>2) Mendelian check<br>3) Missing trait	500	Under water weighing	Residual lung volume: SensorMedics 2900 MMC	[PMID:7674877] Bouchard C, Leon AS, Rao DC, Skinner JS, Wilmore JH and Gagnon J. The HERITAGE Family Study: Aims, design, and measurement protocol. Medicine and Science in Sports and Exercise 27:721-729, 1995<br>[PMID:21183627] Bouchard C, Sarzynski MA, Rice T, Kraus W, Church T, Sung YJ, Rao DC, Rankinen T. Genomic predictors of maximal oxygen uptake response to standardized exercise training programs. Journal of Applied Physiology 110:1160-1170, 2011
Johnston County Osteoarthritis Project	Johnston County Osteoarthritis Project	Population-based	European and African Americans	2,583	≥ 98%	1) sex/gender mismatch<br>2) first-degree relative<br>3) disagreement between reported and genotypic race<br>4) missing height and/or weight or other covariate	1,412	DEXA	Hologic	[PMID:17216685] Joanne M Jordan, Charles G Helmick, Jordan B Renner, Gheorghe Luta, Anca D Dragomir, Janice Woodard, Fang Fang, Todd A Schwartz, Lauren M Abbate, leigh F Callahan, William D Kalsbeek and Marc C Hochberg (2007) Prevalence of knee symptoms and radiographic and symptomatic knee osteoarthritis in African Americans and Caucasians: the Johnston County Osteoarthritis Project. J Rheumatol ;34;172-180
KoGES	Korean Genome Epidemiology Study	Population-based	Korean	2,333	≥ 99%	1) gender mismatch with typed X-linked markers	2,333	DEXA	GE Lunar Prodigy	[PMID: 19396169] YS Cho et al. A large-scale genome-wide association study of Asian populations uncovers genetic factors influencing eight quantitative traits. Nature Genetics 41, 527 - 534 (2009)
KORA F3	Cooperative Health Research in the Augsburg Region	Population-based	European descent	NA	≥ 93%	1) gender mismatch<br>2) missing body fat percentage data	1,560	Bioimpedance	DATA-INPUT GmbH BIA 2000-S	[PMID: 20031538] Heid IM, et al. Genome-wide association analysis of highdensity lipoprotein cholesterol in the population-basedKORA study sheds new light on intergenic regions. Circ Cardiovasc Genet 1, 10-20 (2008).
KORA S4/F4	Cooperative Health Research in the Augsburg Region	Population-based	European descent	NA	≥ 93%	1) gender mismatch<br>2) missing body fat percentage data	1,794	Bioimpedance	DATA-INPUT GmbH BIA 2000-S	[PMID: 16032514] Wichmann HE, et al. KORA-gen - resource for population genetics, controls and a broad spectrum of disease phenotypes. Gesundheitswesen, 67 Suppl 1, S26-30 (2005).
LOLIPOP_EW610	London Life Sciences Prospective Population Study	Population-based	European descent	945	≥ 95%	Duplicates, gender discrepancy, contaminated samples, relatedness	927	Bioimpedance	Tanita TBF-401	[PMID: 21909110] Wain LV,Verwoert GC, O'Reilly PF, et al. Genome-wide association study identifies six new loci influencing pulse pressure and mean arterial pressure. Nat Genet 2001, 43:1005-1011. 2.<br>[PMID:21909115] International Consortium for Blood Pressure Genome-Wide Association Studies, et al. Genetic Variants in novel pathways influence blood pressure and cardiovascular disease risk. Nature 2011, 478:103-109.
LOLIPOP_IA610	London Life Sciences Prospective Population Study	CHD case-control study	Indian Asian	7,032	≥ 95%	Duplicates, gender discrepancy, ethnic outliers, contaminated samples, relatedness	6,549	Bioimpedance	Tanita TBF-401	[PMID: 19820698] Chambers JC, Zhang W, Li Y, et al. Genome-wide association study identifies variants in TMPRSS6 associated with hemoglobin levels. Nat Genet 2009, 41:1170-1172.<br>[PMID: 19651812] Chambers JC, Zhang W, Zabaneh D, et al. Common genetic variation near melatonin receptor MTNR1B contributes to raised plasma glucose and increased risk of type 2 diabetes among Indian Asians and European Caucasians. Diabetes 2009, 58:2703-2708.
LOLIPOP_IA317	London Life Sciences Prospective Population Study	Population-based with some enrichment	Indian Asian	2,694	≥ 95%	Duplicates, gender discrepancy, ethnic outliers, contaminated samples, relatedness, samples already in IA610	2,120	Bioimpedance	Tanita TBF-401	[PMID: 18454146] Chambers JC, Elliott P, Zabaneh D, et al. Common Genetic variation near MC4R is associated with waist circumference and insulin resistance. Nat Genet 2008, 40: 716-718.
LOLIPOP_IA_P	London Life Sciences Prospective Population Study	Population-based with some enrichment	Indian Asian	1,005	≥ 95%	Duplicates, contaminated samples, samples already in IA610	612	Bioimpedance	Tanita TBF-401	[PMID: 18193046] Kooner JS, Chambers JC, Aguilar-Salinas CA, et al. Genome-wide scan identifies variation in MLXIPL associated with plasma triglycerides. Nat Genet 2008, 40:149-151.

MAP	Rush Memory and Aging Project	Longitudinal Cohort Study	European descent	891	95%	1) genotype-derived gender discordant with reported gender;<br>2) inbreeding coefficient F>0.04;<br>3) missing phenotypes	627	BIA	Portable Body Comp Scale by American Weights & Measure, California	[PMID: 22471867]<br>Bennett DA, Schneider JA, Buchman AS, Barnes LL, Boyle PA, Wilson RS. Overview and findings from the rush memory and aging project. Curr Alzheimer Res. 2012;9:646-63.
MrOS	Osteoporotic Fractures in Men Study	Population-based	European descent	5,994	≥97%	1) genotypic sex mismatch,<br>2) relatedness by estimated IBD,<br>3) gross chromosomal abnormalities by BAF in > 5 chromosomes,<br>4) non-European ethnic outliers from PCA	4,550	DEXA	Hologic QDR 4500	[PMID: 16084776] Orwoll, E., et al. Design and baseline characteristics of the osteoporotic fractures in men (MrOS) study -- a large observational study of the determinants of fracture in older men. Contemp Clin Trials. 2005;26:569-85.
ORCADES	Orkney Complex Disease Study	Population-based	European descent	875	97%	ethnic outliers, people with missing phenotype data	860	Bioimpedance	Tanita bodyfat scales	[PMID: 18760389]<br>McQuillan R, et al. Runs of homozygosity in European populations. Am J Hum Genet. 2008;83:359-72
PIVUS	The Prospective Investigation of the Vasculature in Uppsala Seniors	Population-based	European descent	1,016	≥ 95%	1) heterozygosity >3 SD;<br>2) gender discordance;<br>3) ethnic outliers;<br>4) related individuals and duplicates;<br>5) missing phenotype data.	826	DXA	GE Lunar Prodigy	[PMID: 16141402]<br>Lind, L. et al. A comparison of three different methods to evaluate endothelium-dependent vasodilation in the elderly: the Prospective Investigation of the Vasculature in Uppsala Seniors (PIVUS) study. Arterioscler Thromb Vasc Biol 25, 2368-75 (2005).
PREVEND	Prevention of renal and vascular end-stage disease	Population-based	European descent	3,649	≥ 95%	1) related individuals<br>2) sample outliers based on 16,842 independent SNPs. Samples were excluded when Z-score > 3 on the first 5 PCA's .<br>3) sex inconsistencies	3,622	Bioimpedance		[PMID: 12356629]<br>Hillege HL, et al. Urinary albumin excretion predicts cardiovascular and noncardiovascular mortality in general population.Circulation. 2002;106:1777-82.
QFS	Quebec Family Study	Population-based	European descent	951	≥ 95%	NA	860	Underwater weighing		[PMID: 3391737]<br>Bouchard et al., Inheritance of the amount and distribution of human body fat. International Journal of Obesity 12, 205-215 (1988).
RISC	Relationship between Insulin Sensitivity and Cardiovascular disease Study	Population-based	European descent	1,566	>95%	1) Non-European descent<br>2) Sex mismatches	1,247	Bioimpedance	Tanita TBF-300 Body Composition Analyser	[PMID:14968294]<br>Hills, S.A. et. al, The EGIR-RISC STUDY (The European group for the study of insulin resistance: relationship between insulin sensitivity and cardiovascular disease risk): I. Methodology and objectives, Diabetologia, 47, 556-570 (2004)
RS I	Rotterdam Study I	Population-based	European descent	7,983	≥ 97.5%	1) missing DNA;<br>2) gender mismatch with typed X-linked mark ers;<br>3) excess autosomal heterozygosity >0.336~ FDR>0.1%;<br>4) duplicates and/or 1st or 2nd degree relativ es using IBS probabilities >97% from PLINK;<br>5) ethnic outliers using IBS distances >3SD f rom PLINK;<br>6) missing phenotype.	2,438	DEXA	Lunar Prodigy, GE Healthcare	[PMID: 24258680]<br>Hofman A et al. The Rotterdam Study: 2014 objectives and design update. Eur J Epidemiol. 2013 Nov;28(11):889-926<br>[PMID: 19700477]
RS III	Rotterdam Study III	Population-based	European descent	3,932	≥ 97.5%	1) gender mismatch with typed X-linked markers;<br>2) excess autosomal heterozygosity (F<-0.055);<br>3) duplicates and/or 1st degree relatives using IBD PiHAT >40% from PLINK;<br>4) ethnic outliers IBS distances > 4SD mean HaMAP CEU cluster from PLINK;<br>5) Missing body weight and height.	1,594	DXA	GE-Lunar Prodigy	[PMID: 24258680]<br>Hofman A et al. The Rotterdam Study: 2014 objectives and design update. Eur J Epidemiol. 2013 Nov;28(11):889-926
SHIP	Study of Health in Pomerania (follow-up 2)	Population-based	European descent	4,310	≥ 92%	1) duplicate samples (by estimated IBD)<br>2) reported/genotyped gender mismatch	2,013	Bioimpedance	Data Input Nutriguard M	[PMID: 20167617]<br>Völzke H, et al. Cohort profile: the study of health in Pomerania. Int J Epidemiol. 2011;40:294-307.
SHIP-TREND	Study of Health in Pomerania - TREND	Population-based	European descent	997	≥ 94%	1) duplicate samples (by estimated IBD)<br>2) reported/genotyped gender mismatch	961	Bioimpedance	Data Input Nutriguard M	[PMID: 20167617]<br>Völzke H, et al. Cohort profile: the study of health in Pomerania. Int J Epidemiol. 2011;40:294-307.
SOF	Study of Osteoporotic Fractures	Population-based	European descent	9,704	≥97%	1) genotypic sex mismatch,<br>2) relatedness by estimated IBD,<br>3) gross chromosomal abnormalities by BAF in > 5 chromosomes,<br>4) non-European ethnic outliers from PCA	3,422	DEXA	Hologic QDR 1000	[PMID: 7862179]<br>Cummings SR, Nevitt MC, Browner WS, et al. Risk factors for hip fracture in white women. Study of Osteoporotic Fractures Research Group. The New England journal of medicine. 1995;332:767-773.<br>[PMID: 1414480]<br>Steiger P, Cummings SR, Black DM, Spencer NE, Genant HK. Age-related decrements in bone mineral density in women over 65. Journal of bone and mineral research : the official journal of the American Society for Bone and Mineral Research. 1992;7:625-632.
SORBS	Sorbs are self-contained population from Eastern Germany, European Descent	Population-based	European descent	1,097	≥ 94%	1) gender mismatch;<br>2) ethnic outliers;<br>3) duplicates;<br>4) missing body weight and height.	876	BIA	BIA-2000-S (Data Input GmbH, Darmstadt, Germany)	[PMID:19584900]<br>Tönjes A et al. Association of FTO variants with BMI and fat mass in the self-contained population of Sorbs in Germany. Eur J Hum Genet. 2010;18:104-10
TwinsUK	TwinsUK	Twins Study	European descent	5,654	≥ 98%	1) heterozygosity ≥2 s.d. from the sample mean;<br>2) ethnic outliers;<br>3) related individuals and duplicates;<br>4) missing body weight and height.	2,204	DEXA	Hologic Discovery W' - QDR software version 12.6	[PMID: 22253318]<br>Moayyeri, A. et al. Cohort Profile: TwinsUK and healthy ageing twin study. Int J Epidemiol. 42, 76-85 (2013)
ULSAM	The Uppsala Longitudinal Study of Adult Men	Population-based	European descent	1,221	≥ 95%	1) heterozygosity >3 SD;<br>2) gender discordance;<br>3) ethnic outliers;<br>4) related individuals and duplicates;<br>5) missing phenotype data.	483	DEXA	GE Lunar Prodigy	[PMID: 16030278]<br>Ingelsson, E. et al. Insulin resistance and risk of congestive heart failure. JAMA 20, 334-41 (2005).
YFS	The Cardiovascular Risk in Young Finns Study	Population-based	European descent	2,556	≥ 95%	1) heterozygosity, gender check and relatedness checks have been performed and any discrepansies have been removed.<br>2) missing phenotype.	337	Bioimpedance	InBody 3.0	[PMID:18263651]<br>Raitakari OT, Juonala M, Rönnemaa T, Keltikangas-Järvinen L, Räsänen L, Pietikäinen M, Hutri-Kähönen N, Taittonen L, Jokinen E, Marniemi J, Jula A, Telama R, Kähönen M, Lehtimäki T, Akerblom HK, Viikari JS.Cohort profile: the cardiovascular risk in Young Finns Study. Int J Epidemiol. 2008;37:1220-6.
Metabochip studies										
DESIR	Data from an Epidemiological Study on the Insulin Resistance syndrome	Population-based	European descent	4,993	≥ 90%	1) Missing data	3,925	Bioimpedance	TBF 300P balance from Tanita (Neuilly-sur-Seine, France)	[PMID: 8927780]<br>Balkau B (1996) An epidemiologic survey from a network of French Health Examination Centres, (D.E.S.I.R.): epidemiologic data on the insulin resistance syndrome, Rev Epidemiol Sante Publique 44(4):373-5.

DILGOM	Dietary, life style, and genetic determinants of obesity and metabolic syndrome	Population-based	European descent	3,997	≥ 95%	1) heterozygosity <23.9% or >27.6%;<br>2) ethnic outliers;<br>3) related individuals and duplicates.	3,835	Bioimpedance	TANITA TBF-300MA	[PMID: 20138944]<br>Konttinen H, Männistö S, Sarlio-Lähteenkorva S, Silventoinen K, Haukkala A. Emotional eating, depressive symptoms and self-reported food consumption. A population-based study. Appetite. 2010;54:473-9.<br>[PMID: 20844574]<br>Inouye M, Silander K, Hamalainen E, Salomaa V, Harald K, Jousilahti P, Männistö S, Eriksson JG, Saarela J, Ripatti S, Perola M, van Ommen GJ, Taskinen MR, Palotie A, Dermitzakis ET, Peltonen L. An immune response network associated with blood lipid levels. PLoS Genet. 2010;6(9).<br>Peltonen, M., Harald, K., Männistö, S., Saarikoski, L., Peltomäki, P., Lund, L., et al. (2008). The National FINRISK 2007 Study. Helsinki: Publications of the National Public Health Institute, B 34/2008.
Ely Study	MRC Ely Study	Population-based	European descent	1,625	≥ 95%	1) missing phenotype data<br>2) gender check;<br>3) duplicates check;	1,582	Bioimpedance	BODY STAT 1500	[PMID: 17257284]<br>Forouhi NG. et al.Incidence of Type 2 diabetes in England and its association with baseline impaired fasting glucose: the Ely study 1990-2000.Diabet Med. 2007;24:200-7.
EPIC-Norfolk T2D	EPIC (European Prospective Investigation into Cancer) Norfolk T2D case-Cohort Study	Population-based	European descent	963 - cohort;<br>727 -T2D	≥ 95%	1) missing body weight and height, or case-control status.<br>2) heterozygosity<br>3) gender check	583 - cohort;<br>402 - T2D	Bioimpedance	Tanita TBF300	[PMID: 10466767]<br>Day,N.E. et al. EPIC-Norfolk: study design and characteristics of the cohort. European Prospective Investigation of Cancer. British Journal of Cancer 80, 95-103 (1999).<br>[PMID: 18454148]<br>Loos,R.J. et al. Common variants near MC4R are associated with fat mass, weight and risk of obesity. Nat Genet 40, 768-775 (2008).
Fenland	The Fenland Study	Population-based	European descent	3,217	≥ 95%	1) missing phenotype data<br>2) gender check;<br>3) duplicates check;	3,132	DEXA	GE Lunar Prodigy Advance	[PMID: 20519560]<br>Rolfe Ede L, Loos RJ, Druet C, Stolk RP, Ekelund U, Griffin SJ, Forouhi NG, Wareham NJ, Ong KK. Association between birth weight and visceral fat in adults. Am J Clin Nutr 2010;92:347-52.
HEALTH2006	HEALTH2006	Population-based	white European	2,374	≥ 95%	1) Missing body weight and height.<br>2) Heterozygosity<br>3) Gender check<br>4) Duplicates check	2,277	Bioimpedance	foot-to-foot Tanita Body Composition /	[PMID: 23615486]<br>Thuesen BH, Cerqueira C, Aadahl M, Ebstrup JF, Toft U, Thyssen JP, Fenger RV, Hersoug LG, Elberling J, Pedersen O, Hansen T, Johansen JD, Jørgensen T, Linneberg A.Cohort Profile: The Health2006 cohort, Research Centre for Prevention and Health.Int J Epidemiol. 2014;43:568-75.<br>[PMID: 25532602]<br>Fenger RV, Gonzalez-Quintela A, Vidal C, Husemoen LL, Skaaby T, Thuesen BH, Aadahl M, Madsen F, Linneberg A. The longitudinal relationship of changes of adiposity to changes in pulmonary function and risk of asthma in a general adult population.BMC Pulm Med. 2014;14:208
GXE	Gene By Environment	Population-based	Jamaican	1,039	≥ 95%	1) Missing phenotype data;<br>2) heterozygosity;<br>3) PCA outliers;<br>4) IBD cryptic relateds	613	Bioimpedance	BIA 101Q (RJL Systems)	[PMID:9103091]<br>Cooper R, Rotimi C, Ataman S, McGee D, Osotimehin B, Kadiri S, Muna W, Kingue S, Fraser H, Forrester T, Bennett F, Wilks R. The prevalence of hypertension in seven populations of west African origin. Am J Public Health. 1997;87:160-8.<br>[PMID:9098179]<br>Luke A, Durazo-Arvizu R, Rotimi C, Prewitt TE, Forrester T, Wilks R, Ogunbiyi OJ, Schoeller DA, McGee D, Cooper RS. Relation between body mass index and body fat in black population samples from Nigeria, Jamaica, and the United States. Am J Epidemiol. 1997;145:620-8.<br>[PMID:20400458]<br>Kang SJ, Chiang CW, Palmer CD, Tayo BO, Lettre G, Butler JL, Hackett R, Adeyemo AA, Guiducci C, Berzins I, Nguyen TT, Feng T, Luke A, Shriner D, Ardlie K, Rotimi C, Wilks R, Forrester T, McKenzie CA, Lyon HN, Cooper RS, Zhu X, Hirschhorn JN. Genome-wide association of anthropometric traits in African- and African-derived populations. Hum Mol Genet. 2010;19:2725-38.
SPT	Spanish Town	Population-based	Jamaican	1,831	≥ 95%	1) Missing phenotype data;<br>2) heterozygosity;<br>3) PCA outliers;<br>4) IBD cryptic relateds	476	Bioimpedance	BIA 101Q (RJL Systems)	[PMID:9103091]<br>Cooper R, Rotimi C, Ataman S, McGee D, Osotimehin B, Kadiri S, Muna W, Kingue S, Fraser H, Forrester T, Bennett F, Wilks R. The prevalence of hypertension in seven populations of west African origin. Am J Public Health. 1997;87:160-8.<br>[PMID:9098179]<br>Luke A, Durazo-Arvizu R, Rotimi C, Prewitt TE, Forrester T, Wilks R, Ogunbiyi OJ, Schoeller DA, McGee D, Cooper RS. Relation between body mass index and body fat in black population samples from Nigeria, Jamaica, and the United States. Am J Epidemiol. 1997 145:620-8.<br>[PMID:20400458]<br>Kang SJ, Chiang CW, Palmer CD, Tayo BO, Lettre G, Butler JL, Hackett R, Adeyemo AA, Guiducci C, Berzins I, Nguyen TT, Feng T, Luke A, Shriner D, Ardlie K, Rotimi C, Wilks R, Forrester T, McKenzie CA, Lyon HN, Cooper RS, Zhu X, Hirschhorn JN. Genome-wide association of anthropometric traits in African- and African-derived populations. Hum Mol Genet. 2010;19:2725-38.
KORA F3	Cooperative Health Research in the Region of Augsburg (third survey), KOoperative Gesundheitsforschung in der Region Augsburg (dritte Studie)	Population-based	European descent	1,482 (not included in GWAS stage)	none	none	1,381	Bioimpedance	DATA-INPUT GmbH BIA 2000-S	Wichmann H.E. et al. KORA-gen--resource for population genetics, controls and a broad spectrum of disease phenotypes. Gesundheitswesen (2005) 67 Suppl 1, S26-30.
KORA S4/F4	Cooperative Health Research in the Region of Augsburg (forth survey), KOoperative Gesundheitsforschung in der Region Augsburg (vierte Studie)	Population-based	European descent	1,222 (not included in GWAS stage)	none	none	1,191	Bioimpedance	DATA-INPUT GmbH BIA 2000-S	Wichmann H.E. et al. KORA-gen--resource for population genetics, controls and a broad spectrum of disease phenotypes. Gesundheitswesen (2005) 67 Suppl 1, S26-30.
Leipzig Adults	Leipzig adults	Population-based	European descent	1,005	≥ 95%	ethnic outliers / gender discordance	1,005	Bioimpedance	BIA-Nutriguard-MS with the software Nutri3	[PMID: 20935630]<br>Speliotes EK, Willer CJ, Berndt SI, Monda KL, Thorleifsson G, Jackson AU et al. Association analyses of 249,796 individuals reveal 18 new loci associated with body mass index. Nat Genet. 2010;42:937-48.
METSIM	Metabolic Syndrome In Men	Population-based, although T2D case-control samples selected for metabochip typing	European descent	2,176	≥ 98.15%	1) Missing BMI, age, age<18;<br>2) Gender discrepancy or anomaly;<br>3) Unexplained duplicate sample.	1,204 cases, 957 controls	Bioimpedance	Bioimpedance Analyzer Model BIA101 (Akern Srl, Florence Italy)	[PMID: 19223598]<br>Stancakova A, Javorsky M, Kuulasmaa T, Haffner SM, Kuusisto J, Laakso M: Changes in insulin sensitivity and insulin release in relation to glycemia and glucose tolerance in 6416 Finnish men. Diabetes 58:1212-1221, 2009.
Whitehall	The Whitehall II study	Cohort of London-based civil servants	European descent	3,413	≥ 95%	1) missing phenotype data<br>2) gender check;<br>3) duplicates check;	2,223	Bioimpedance	Tanita TBF-300 Body Composition Analyser	[PMID: 15576467]<br>Marmot M, Brunner E. Cohort Profile: the Whitehall II study. Int J Epidemiol. 2005; 34:251-6.<br>[PMID: 21441441]<br>Jensen AC et al. Associations of common genetic variants with age-related changes in fasting and postload glucose: evidence from 18 years of follow-up of the Whitehall II cohort. Diabetes. 2011; 60:1617-23.

* Call rate to exclude individuals for whom genotyping success rate is less than a certain percentage (to exclude 'bad' samples/DNA)

Supplementary Table 2. Information on genotyping methods, quality control of SNPs, imputation, and statistical analysis for genome-wide association studies and metabochip studies

	Genotyping						Imputation			Association analyses	
			Inclusion criteria					Inclusion criteria			
Study	Platform	Genotype calling algorithm	MAF	Call rate*	*p* for HWE	SNPs that met QC criteria	Imputation software	MAF	Imputation quality*	SNPs in meta-analysis	Analyses software
Genome-wide association studies											
AGES	Illumina 370K	BeadStudio	-	≥ 95%	$> 10^{-6}$	315,410	MACH	-	r^2-hat ≥ 0.30	2,465,997	ProbABEL
Amish	Affymetrix 500K	BRLMM	-	≥ 96%	$> 10^{-6}$	382,935	MACH	-	r^2-hat ≥ 0.30	2,302,463	ITSNBN
BASE-II	Affymetrix Genome-Wide Human SNP Array 6.0	BRLMM	≥ 1%	≥ 98%	$> 10^{-6}$	706,329	IMPUTE2	≥5%	proper-info ≥ 0.80	5,762,436	SNPtest
BLSA	Illumina 550K	Beadstudio	≥ 1%	≥ 99%	$> 10^{-4}$	514,027	MACH	≥1%	r^2 ≥ 0.30	2,543,602	MERLIN-OFFLINE
BPROOF	Illumina Omni Express	GenomeStudio	-	> 95%	-	722,053	MACH	>1%	r^2 ≥ 0.3	2,450,937	GRIMP
CHS	Illumina 370-CNV	BeadStudio		≥ 97%	$\geq 10^{-5}$	306,655**	BimBam v0.99	-	observed/expected variance ≥ 0.30	2,195,299	R
CoLaus	Affymetrix 500K	BRLMM	-	> 70%	$> 10^{-7}$	390,631	IMPUTE	-	proper-info ≥ 0.40	2,439,888	QUICKTEST
COROGENE CONTROLS	Illumina BeadChip Human 610-Quad	Illuminus	≥ 5%	≥ 95%	$> 10^{-6}$	554,988	MACH 1.0 (Hapmap r22 CEU)	-	-	2,543,887	ProbABEL
CROATIA-Vis	Illumina HumanHap300v1	BeadStudio	≥ 1%	≥ 98%	$> 10^{-6}$	308,996	MACH	NA	NA	2,543,888	ProbABEL
DESIR	Illumina Human CNV370-Duo Array and Illumina HAP300 array	Illumina Beadstation Genotyping Solution GenomeStudio v2010.2	≥ 1%	≥ 95%	$> 10^{-4}$	291,609	IMPUTE	≥1%	proper-info ≥ 0.40	2,544,187	SNPtest
EPIC-Obesity Study (obese cases)	Affymetrix 500K	BRLMM	-	≥ 90%	$> 10^{-6}$	397,438	IMPUTE	-	proper-info ≥ 0.40	2,381,011	SNPtest
EPIC-Obesity Study (controls)	Affymetrix 500K	BRLMM	-	≥ 90%	$> 10^{-6}$	397,438	IMPUTE	-	proper-info ≥ 0.40	2,428,445	SNPtest
Erasmus Ruchphen Family Study (ERF)	Illumina 318K, Illumina 370K and Affymetrix 250K	Illumina Bead Studio, BRLMM	-	≥ 96%	$> 10^{-6}$	487,573	MACH	-	r^2-hat ≥ 0.30	2,468,052	ProbABLE
ERGO(ROTTERDAM)	Illumina Infinium 550	BeadStudio	-	≥ 97.5%	$> 10^{-6}$	512,349	MACH	-	r^2-hat ≥ 0.30	2,488,215	MACH2QTL
Fels Longitudinal Study	Illumina Human 610 Quad v1_B	Illumina's GenomeStudio (v1.5.16)	≥ 1%	≥ 90%	$> 10^{-6}$	542,711	MACH1	≥ 0.02% (SNP has at least 1 heterzygote)	r^2-hat ≥ 0.001	2,547,310	SOLAR
FamHS	Illumina HumMap 550k, Human610-Quadv1, Human 1M-Duov3	BeadStudio	≥ 1%	≥ 98%	$> 10^{-6}$	501,404	MaCH	≥1%	r^2-hat ≥ 0.30	2,543,887	SAS, R
Fenland	Affymetrix 500K	BRLMM	-	≥ 90%	$> 10^{-6}$	362,055	IMPUTE	-	proper-info ≥ 0.40	2,427,084	SNPtest
Framingham	Affymetrix 500K Affymetrix 50K supplemental	BRLMM	-	≥ 97%	$> 10^{-6}$	378,163	MACH	-	r^2-hat ≥ 0.30	2,455,944	R
GOYA	Illumina 610K Quad CHIP	GenCall	≥ 1%	≥ 95%	$> 10^{-7}$	545,349	MACH	≥1%	proper-info ≥ 0.30	2,543,887	QUICKTEST
GOOD	Illumina 610K	BeadStudio	-	≥ 98%	$> 10^{-6}$	521,160	MACH	-	r^2-hat ≥ 0.30	2,503,211	MACH2QTL
H2000 CASES	Illumina Human610-Quad BeadChip	Illuminus	≥ 5%	≥ 95%	$> 10^{-6}$	555,388	MACH 1.0 (Hapmap r22 CEU)	-	-	2,543,887	ProbABEL
H2000 CTRLS	Illumina Human610-Quad BeadChip	Illuminus	≥ 5%	≥ 95%	$> 10^{-6}$	555,388	MACH 1.0 (Hapmap r22 CEU)	-	-	2,543,887	ProbABEL
Health ABC	Illumina 1M-Duo	Beadchip	≥ 1%	≥ 97%	$> 10^{-6}$	914,263	MACH	n/a	r^2-hat ≥ 0.30	2,543,887	R
HBCS	Illumina custom made BeadChip Human 670-Quad	Illuminus	≥ 5%	≥ 95%	$> 10^{-6}$	533,491	MACH 1.0 (Hapmap r22 CEU)	-	-	2,543,887	ProbABEL
HERITAGE Family Study	Illumina HumanCNV370-Quad v3.0	Illumina Genome Studio	≥ 1%	≥ 99%	$> 10^{-6}$	324,607	MACH	≥1%	r^2-hat ≥ 0.30	2,453,887	ProbABEL
Johnston County Osteoarthritis Project	Illumina Infinium 1M-Duo bead arrays	BeadStudio	>0.5%	>98%	$> 10^{-4}$	1,065,734	MACH	None	None	2,543,887 (European Americans) 2,823,316 (African American)	R, ProbABEL
KoGES	Affymetrix Genechip 5.0	birdseed-v2	≥ 1%	≥ 95%	$> 10^{-7}$	352,228	IMPUTE	≥1%	-	3,031,006	PLINK
KORA F3	Affymetrix 500K	BRLMM	-	≥ 90%	-	490,032	MACH	-	r^2-hat ≥ 0.30	2,416,530	MACH2QTL
KORA S4/F4	Affymetrix 6.0	Birdseed2	-	-	-	909,622	IMPUTE	-	proper-info ≥ 0.40	2,040,316	SNPtest
LOLIPOP_EW610	Illumina Human610	BeadStudio	≥ 1%	≥ 90%	$> 10^{-6}$	544,620	MACH	All	All	2,543,887	mach2qtl
LOLIPOP_IA610	Illumina Human610	BeadStudio	≥ 1%	≥ 90%	$> 10^{-6}$	544,390	MACH	All	All	2,177,742	mach2qtl
LOLIPOP_IA317	Illumina HumanHap300K	BeadStudio	≥ 1%	≥ 90%	$> 10^{-6}$	245,892	MACH	All	All	1,958,375	mach2qtl
LOLIPOP_IA_P	Perlegen custom	Perlegen custom	≥ 1%	≥ 90%	$> 10^{-6}$	170,055	MACH	All	All	1,958,375	mach2qtl
MAP	Affymetrix 6.0	Birdsuite, Broad Institute	≥ 1%	≥ 95%	$> 10^{-6}$	750,173	MACH (version 1.0.16a)	-	-	2,543,887	plink
MrOS	Illumina HumanOmni1_Quad_v1-0 B	BeadStudio	≥ 1%	≥ 97%	$> 10^{-4}$	740713	MINIMAC	≥ 1%	R2MACH ≥ 0.3	2391250	R
ORCADES	Illumina HumanHap300v2	BeadStudio	≥ 1%	≥ 98%	$> 10^{-6}$	293,687	MACH	NA	NA	2,543,888	ProbABEL
PIVUS	Illumina OmniExpress+Metabochip	GenomeStudio 2010.3	≥ 1%	≥99% (MAF<5%) or ≥95% (MAF≥5%)	$> 10^{-6}$	738,879	IMPUTE2	≥1%	info ≥ 0.40	2,611,259	PLINK
PREVEND	Illumina CytoSNP12 v2	GenomeStudio	≥ 1%	≥ 95%	$> 10^{-5}$	232,571	beagle	-	-	2,269,099	Plink 1.07
QFS	Illumina 610 Quad chip	Bead Studio Genotyping software	≥ 1%	≥ 95%	$> 10^{-4}$	543,713	MACH	≥1%	r^2-hat ≥ 0.30	2,460,214	GWAF
RISC	Affymetrix 6.0	Birdseed	>1%	≥98%	$> 10^{-4}$	747,423	MACH	>1%	r^2-hat ≥ 0.30	2,500,497	MACH2QTL
RSIII	Illumina / HumanHap 610 QUAD	Genomestudio Genecall	≥ 1%	≥ 97.5%	$> 10^{-6}$	514,073	MACH	≥1%	MACH R^2 ≥ 0.3	2,543,887	MACHQTL
SHIP	Affymetrix Human SNP Array 6.0	Birdseed V2	≥ 0%	≥ 0%	≥ 0	869,224	IMPUTE	NA	NA	2,748,910	QUICKTEST
SHIP-TREND	Illumina Human Omni 2.5	GenCall	> 0%	> 90%	$> 10^{-4}$	1,782,967	IMPUTE	NA	NA	3,437,411	QUICKTEST
SOF (Study of Osteoporotic Fractures)	Illumina HumanOmni1_Quad_v1-0 B	BeadStudio	≥ 1%	≥ 97%	$> 10^{-4}$	740,713	MINIMAC	≥ 1%	R2MACH ≥ 0.3	2,391,250	R
SORBS	500K Affymetrix GeneChip (250K Sty and 250K Nsp arrays, Affymetrix, Inc) and Affymetrix Genome-Wide Human SNP Array 6.0	BRLMM algorithm (Affymetrix, Inc) for 500K and Birdseed Algorithm for Genome-Wide Human SNP Array 6.0	>1%	≥ 95%	$> 10^{-4}$	378,513	IMPUTE	>1%	proper-info ≥ 0.40	2,514,716	ProbABEL
TwinsUK	Illumina 317, Illumina 660, Illumina Omni Express 1M	Illuminus	≥ 1%	≥ 98%	$> 10^{-6}$	up to 874,733	IMPUTE V2	≥1%	-	3,044,097	SNPtest
ULSAM	Illumina Omni2.5+Metabochip	GenomeStudio 2010.3	≥ 1%	≥99% (MAF<5%) or ≥95% (MAF≥5%)	$> 10^{-6}$	1,587,454	IMPUTE2	≥1%	info ≥ 0.40	3,189,521	PLINK
YFS	Illumina custom made BeadChip Human 670-Quad	Illuminus	≥ 5%	≥ 95%	$> 10^{-6}$	546,677	MACH 1.0 (Hapmap r22 CEU)	-	-	2,543,887	ProbABEL
Metabochip studies											

DESIR	Illumina Metabochip	GeneCall				194,763	NA	NA	NA		PLINK
DILGOM	Metabochip	GenCall	none	≥ 95%	none	183,872	NA	NA	NA		PLINK
Ely Study	Illumina Metabochip	GeneCall	> 0%	≥ 90%	$> 10^{-6}$	149,302	NA	NA	NA	149,302	PLINK
EPIC-Norfolk T2D	Illumina Metabochip	GeneCall	> 0%	≥ 90%	$> 10^{-6}$	143,294	NA	NA	NA	143,294	PLINK
Fenland	Illumina Metabochip	GeneCall	> 0%	≥ 90%	$> 10^{-6}$	167,085	NA	NA	NA	167,085	PLINK
HEALTH2006	Illumina Metabochip	GeneCall	> 0%	≥ 90%	$> 10^{-7}$	196,725	NA	NA	NA	NA	PLINK
INVNORM_GXE	Illumina Metabochip	Birdseed	> 0%	≥ 90%	$> 10^{-6}$	195,531	NA	NA	NA	195,531	PLINK
INVNORM_SPT	Illumina Metabochip	Birdseed	> 0%	≥ 90%	$> 10^{-6}$	195,567	NA	NA	NA	195,567	PLINK
KORA F3	Metabochip	Genome Studio	none	none	none	185,781	NA	NA	NA	142,780	PLINK
KORA S4/F4	Metabochip	Genome Studio	none	none	none	185,781	NA	NA	NA	144,412	PLINK
Leipzig Adults	Illumina Metabochip	GeneCall	> 0%	≥ 95%	for MAF>=0.05, HWE p value $<5.7x10^{-7}$ / for	141,071	NA	NA	NA	141,071	SNPTEST
METSIM	Illumina Metabochip	BeadStudio version 3.3.7, Gentrain version 1.0	> 0%	≥ 98.15%	provided p for all SNPs but did not exclude	194,729	NA	NA	NA	139,044(controls), 135,976 (cases)	EMMAX
Whitehall metabochip	Illumina Metabochip	GeneCall	> 0%	≥ 90%	$> 10^{-6}$	171,257	NA	NA	NA	171,257	PLINK

* Call rate to exclude SNPs for which less than a certain percentage of individuals were successfully genotyped (i.e. to exclude 'bad' SNPs)
**Further exclusions in CHS are: > 2 duplicate errors or Mendelian inconsistencies, heterozygote frequency = 0.

Supplementary Table 3. Study-specific descriptive statistics of study cohorts

Study	Men													Women												
		Age, yrs		BMI, kg/m2		Height, m		weight, kg		Total fat mass, kg		Fat %			Age, yrs		BMI, kg/m2		Height, m		weight, kg		Total fat mass, kg		Fat %	
	n	mean	SD	mean	SD	mean	SD	mean	SD	mean	SD	mean	SD	n	mean	SD	mean	SD	mean	SD	mean	SD	mean	SD	mean	SD
Genome-wide association studies																										
AGES	1,019	49.7	5.9	25.6	3.1	1.78	0.06	81.3	11.4	-	-	22.0	5.5	1,356	52.0	6.5	24.9	3.8	1.64	0.05	67.1	10.5	-	-	34.0	5.0
Amish	406	50.7	15.0	26.5	3.4	1.72	0.06	78.2	12.1	15.9	7.5	19.7	6.5	444	52.2	14.4	28.4	5.4	1.60	0.06	72.4	14.4	25.8	8.9	35.4	6.5
BASE II	202	58.1	16.0	26.5	4.2	1.80	0.10	82.3	13.8	23.3	7.9	28.1	6.1	422	57.9	15.2	25.7	5.1	1.60	0.07	68.8	13.2	26.4	8.5	38.2	6.5
BLSA	462	71.3	15.7	27.6	4.5	1.70	0.10	84.2	16.0	26.1	9.9	30.1	6.6	382	66.5	17.2	25.9	4.8	1.60	0.07	68.3	14.0	26.8	10.3	38.1	7.4
BPROOF	562	73.4	6.4	26.8	3.2	1.80	0.10	83.4	11.2	23.1	7.2	27.3	5.9	513	73.4	5.8	27.1	4.3	1.60	0.10	72.8	12.2	28.2	8.9	38.0	6.7
CHS	349	77.6	5.0	26.5	3.4	1.73	0.06	79.4	10.9	23.1	7.7	29.4	6.6	572	76.9	4.3	26.1	4.4	1.59	0.10	66.2	12.1	28.4	9.6	42.6	7.5
CoLaus	2,539	52.9	10.8	26.6	4.2	1.75	0.07	81.5	13.4	-	-	23.9	6.0	2,850	53.9	10.7	25.2	4.9	1.62	0.07	66.4	13.0	-	-	34.4	8.2
COROGENE CONTROLS	312	54.2	12.9	26.8	3.8	1.80	0.10	83.8	12.1	20.6	7.9	23.9	6.3	329	52.9	13.9	26.4	5.4	1.60	0.00	70.4	14.0	25.2	10.0	34.6	8.0
CROATIA-Vis	382	55.9	14.9	27.5	3.7	1.76	0.07	85.1	13.2	22.2	7.6	25.5	5.9	521	56.7	16.0	27.2	4.6	1.62	0.07	71.0	12.5	25.9	8.5	35.8	6.9
DESIR	147	58.6	5.5	23.9	1.7	1.73	0.06	71.8	7.7	14.8	4.1	20.5	4.2	481	54.6	8.7	22.1	2.3	1.60	0.06	56.6	6.9	16.4	5.1	28.3	5.8
EPIC-Obesity Study	1,121	63.2	8.9	28.4	3.9	1.74	0.07	85.9	13.0	19.9	7.6	26.4	7.1	1,422	62.2	8.7	28.5	5.2	1.61	0.06	74.1	13.8	28.4	11.1	43.8	10.5
Erasmus Ruchphen Family Study (ERF)	1,145	48.9	14.2	27.2	4.2	1.75	0.07	83.1	14.0	22.1	8.8	26.8	7.1	1,430	47.9	14.4	26.5	5.0	1.62	0.07	69.3	13.7	26,5182	9.9	38.7	7.6
RS I	1,043	63.9	5.6	25.9	2.8	1.76	0.06	80.5	10.0	23.4	7.6	28.9	6.2	1,395	64.2	6.0	26.3	3.8	1.63	0.06	70.2	10.7	28.4	8.8	29.0	6.6
Fels Longitudinal Study	430	48.4	18.7	27.2	4.8	1.80	0.10	86.8	16.2	20.9	8.7	23.1	6.6	486	49.4	18.1	26.8	5.6	1.60	0.10	72.7	16.0	26.2	10.0	34.9	6.9
FamHS	1,043	55.7	13.3	29.3	4.7	1.76	0.07	90.9	15.7	29.9	10.2	27.3	7.5	1,294	56.6	12.9	28.4	6.2	1.62	0.06	74.7	17.0	33.4	13.0	37.7	8.0
Fenland	572	44.4	7.4	27.4	3.6	1.77	0.07	86.2	13.1	24.7	8.6	28.0	6.7	740	45.2	7.2	26.5	5.3	1.64	0.06	71.1	14.7	27.4	10.7	37.6	7.7
Framingham	1,003	64.1	11.2	26.7	2.9	1.73	0.07	80.1	10.2	22.8	6.1	28.2	5.5	1,745	64.3	11.4	26.3	4.3	1.60	0.07	67.1	11.6	27.9	8.8	41.1	7.3
GOYA CASE	793	43.1	6.2	35.7	5.7	1.78	0.06	113.6	19.6	38.6	6.0	34.5	5.1	-	-	-	-	-	-	-	-	-	-	-	-	-
GOYA COHORT	921	47.8	8.6	26.1	3.6	1.77	0.07	82.2	12.5	32.9	4.3	40.4	5.2	-	-	-	-	-	-	-	-	-	-	-	-	-
GOOD	940	18.9	0.6	22.4	3.2	1.82	0.07	73.9	11.6	13.2	7.9	17.1	7.4	-	-	-	-	-	-	-	-	-	-	-	-	-
H2000 CASES	475	49.4	10.4	29.6	3.6	1.80	0.10	91.4	12.5	23.4	7.5	25.3	5.4	459	52.7	11.3	29.7	4.8	1.60	0.10	76.7	14.0	28.3	9.1	36.2	6.1
H2000 CTRLS	463	48.0	10.0	25.4	3.0	1.80	0.10	78.4	9.9	15.6	5.4	19.6	5.1	511	51.0	11.2	24.9	4.0	1.60	0.10	65.4	10.3	19.8	7.2	29.6	6.5
Health ABC	796	73.9	2.9	27.0	3.7	1.70	0.10	81.4	12.4	24.7	6.9	30.0	4.6	713	73.7	2.8	26.1	4.4	1.60	0.10	66.3	11.7	27.1	7.6	40.2	5.3
HBCS	671	61.4	2.7	27.6	4.3	1.80	0.10	86.4	14.4	21.1	8.8	23.8	6.0	885	61.5	3.0	27.8	5.1	1.60	0.00	74.0	14.0	25.9	10.0	34.0	7.0
HERITAGE Family Study	244	36.5	15.0	26.7	4.9	1.80	0.06	84.4	16.2	20.0	10.8	22.7	9.0	256	35.1	14.1	25.0	4.9	1.60	0.06	67.1	13.7	21.0	10.8	30.0	9.8
Johnston County Osteoarthritis Project (European Americans)	373	65.4	10.2	29.6	4.3	1.70	0.10	89.7	14.9	25.7	7.9	28.0	4.9	623	64.4	10.5	29.4	6.4	1.60	0.10	75.2	17.0	30.7	11.0	39.4	6.0
Johnston County Osteoarthritis Project(African American)	154	62.2	10.2	29.4	5.3	1.70	0.10	89.2	18.2	22.0	9.1	23.8	6.2	262	61.6	11.2	32.6	7.1	1.60	0.10	84.6	19.0	34.8	12.0	39.5	6.0
KoGES	973	64.0	8.3	23.7	3.1	1.70	0.10	65.0	9.9	13.5	5.7	20.1	6.6	1,360	63.8	8.6	24.7	3.3	1.50	0.10	57.1	8.7	19.0	6.1	32.4	6.5
KORA F3	773	62.5	10.0	28.2	3.5	1.73	0.07	84.9	12.1	24.3	7.3	28.1	5.0	787	61.6	9.9	27.9	5.0	1.61	0.06	71.9	13.2	28.1	8.5	38.4	5.2
KORA S4/F4	874	54.1	8.9	28.4	4.2	1.74	0.07	86.5	14.1	24.3	7.6	28.0	4.9	920	53.6	8.8	27.9	5.3	1.61	0.06	72.6	13.7	27.3	8.6	37.5	5.4
LOLIPOP_EW610	-	-	-	-	-	-	-	-	-	-	-	-	-	249	55.7	9.7	26.6	5.1	1.60	0.07	70.6	13.8	26.8	9.8	36.9	6.7
LOLIPOP_IA610	5,527	55.1	10.7	26.8	4.1	1.70	0.10	77.6	13.1	22.2	9.0	27.6	7.1	1,030	56.8	10.0	28.7	5.3	1.60	0.06	69.4	12.7	27.8	9.1	39.1	6.4
LOLIPOP_IA317	2,120	48.3	10.5	26.8	4.3	1.71	0.07	78.7	14.4	22.2	9.9	27.1	7.7	-	-	-	-	-	-	-	-	-	-	-	-	-
LOLIPOP_IA_P	612	51.1	8.3	27.1	4.0	1.70	0.10	79.4	13.8	22.5	9.0	27.7	6.9	-	-	-	-	-	-	-	-	-	-	-	-	-
MAP	156	82.7	6.0	26.5	4.0	1.70	0.10	80.9	13.4	55.0	9.1	31.6	7.2	471	82.7	6.7	26.6	5.4	1.60	0.10	66.9	14.0	40.2	5.7	38.8	6.6
MrOS	4,550	74.0	5.9	27.4	3.8	1.70	0.07	83.5	13.0	21.9	7.1	26.3	5.4	-	-	-	-	-	-	-	-	-	-	-	-	-
ORCADES	391	54.8	15.4	28.1	4.2	1.70	0.07	85.8	13.1	23.2	8.7	26.5	6.9	469	53.1	15.5	27.3	5.3	1.61	0.06	70.7	13.6	26.5	10.1	36.3	7.3
PIVUS	396	71.9	0.8	27.1	3.6	1.76	0.06	83.0	12.4	23.4	8.2	27.6	6.5	430	72.1	0.9	27.0	4.7	1.62	0.06	70.1	12.8	27.9	9.3	38.7	7.3
PREVEND	1,545	62.9	8.1	27.3	3.8	1.78	0.07	86.0	12.8	24.8	8.7	28.4	7.7	1,458	61.2	7.6	27.7	5.0	1.65	0.07	75.5	13.8	29.4	10.9	38.1	8.9
QFS	370	42.5	16.3	27.5	6.4	1.70	0.10	82.0	19.8	20.3	13.2	23.1	9.2	490	42.9	16.9	27.9	8.5	1.60	0.10	71.3	21.8	24.6	14.8	32.5	10.2
RISC	678	43.4	8.5	26.6	3.5	1.80	0.10	84.5	12.6	-	-	34.9	7.1	825	44.5	8.2	25.0	4.4	1.60	0.10	67.6	12.5	NA	NA	55.1	4.5
RS III	680	55.9	5.5	27.6	3.8	1.79	0.07	88.3	11.3	25.2	9.2	27.8	6.7	914	56.3	6.0	27.2	4.7	1.65	0.06	73.9	13.2	29.5	10.4	38.9	7.5
SHIP	940	58.3	13.5	28.7	4.0	1.80	0.10	88.4	14.1	21.9	7.3	24.3	5.2	1,073	56.8	13.3	27.9	5.5	1.60	0.00	73.9	14.0	26.1	10.0	34.3	7.0
SHIP-TREND	424	50.5	14.1	27.9	3.7	1.80	0.10	87.1	12.8	20.8	6.8	23.5	5.1	537	50.3	13.2	26.9	5.1	1.60	0.00	72.6	14.0	25.2	9.0	33.8	7.0
SOF	-	-	-	-	-	-	-	-	-	-	-	-	-	3,422	73.5	5.3	26.4	4.6	1.60	0.06	67.1	12.4	27.1	8.6	39.5	5.8
SORBS	361	48.1	16.7	27.2	4.0	1.77	0.07	85.4	12.7	18.1	7.6	21.2	6.4	515	48.0	15.9	16.9	5.5	1.64	0.07	72.1	14.0	23.6	9.9	32.7	6.5
TwinsUK	-	-	-	-	-	-	-	-	-	-	-	-	-	2,704	47.1	13.0	25.1	5.0	1.62	0.06	66.1	12.0	23.2	9.0	34.2	8.0
ULSAM	483	81.7	0.9	26.0	3.4	1.73	0.06	77.9	10.9	22.5	7.6	28.2	7.0	-	-	-	-	-	-	-	-	-	-	-	-	-
YFS	164	32.1	4.9	26.1	4.3	1.80	0.10	86.0	15.7	15.0	10.1	16.1	6.1	173	32.1	4.9	25.0	5.3	1.70	0.10	69.3	15.2	19.6	9.9	27.0	7.4
Metabochip studies																										
DESIR	1,690	52.8	10.0	26.3	3.5	1.73	0.07	78.2	11.4	18.8	7.3	23.5	6.2	1,409	52.9	10.2	26.0	4.5	1.59	0.06	65.7	11.6	23.2	8.6	34.2	7.2
DILGOM	1,754	53.0	13.5	27.2	4.1	1.80	0.10	84.0	14.1	21.8	9.6	25.1	6.7	2,081	51.8	13.5	26.8	5.4	1.60	0.10	70.8	14.0	26.1	11.0	35.5	8.0
Ely Study	737	61.5	9.1	27.4	4.0	1.70	0.10	83.1	13.3	22.4	7.0	26.6	5.2	845	60.8	9.2	27.3	5.4	1.60	0.06	71.1	14.4	29.0	10.2	39.9	6.5
EPIC-Norfolk T2D cases	242	62.4	7.7	28.9	3.4	1.74	0.06	87.5	11.5	24.3	8.5	27.2	6.6	160	61.2	8.5	29.8	5.1	1.60	0.06	76.5	14.0	35.9	12.7	45.8	9.8
EPIC-Norfolk T2D sub-cohort	257	60.1	8.7	26.2	3.1	1.75	0.07	79.9	11.0	18.7	7.1	22.9	5.9	326	57.6	9.1	25.7	3.7	1.61	0.06	67.0	10.6	26.9	9.4	39.2	8.5
Fenland Study	1,465	46.9	7.2	27.0	4.1	1.80	0.10	85.4	14.1	20.8	8.0	23.7	5.7	1,667	46.9	7.0	26.5	5.4	1.64	0.06	71.1	15.3	26.3	10.9	35.7	7.2
HEALTH2006	1,033	50.5	13.1	26.6	4.2	1.79	0.07	85.2	14.9	21.2	9.6	24.0	6.8	1,298	49.1	13.2	25.4	5.0	1.66	0.06	69.6	14.2	25.2	10.5	34.8	7.6
INVNORM_GXE	140	38.7	8.9	27.7	6.9	1.80	0.10	85.4	23.0	28.6	14.2	31.7	8.6	473	40.0	8.1	32.3	7.5	1.60	0.10	86.3	21.0	39.0	14.0	44.0	7.1
INVNORM_SPT	175	46.9	14.1	24.9	4.5	1.70	0.10	75.2	16.1	18.3	10.0	23.2	8.9	301	46.3	13.2	28.9	5.9	1.60	0.10	75.3	16.8	28.4	11.2	36.4	8.0
KORA F3	650	50.2	12.4	27.7	4.0	1.76	0.07	86.0	13.6	27.0	5.3	31.5	4.7	731	50.8	12.1	26.3	5.1	1.63	0.07	69.5	13.3	36.1	5.8	52.6	6.1
KORA S4/F4	580	42.4	15.7	26.6	3.6	1.76	0.07	82.6	11.7	26.4	5.2	31.9	4.7	611	41.4	14.8	26.0	5.1	1.63	0.07	68.9	13.5	35.7	5.9	52.4	6.0
Leipzig Adults	222	42.6	16.9	37.4	14.0	1.80	0.07	120.8	45.3	36.0	12.6	28.7	5.0	393	47.2	15.1	40.3	12.4	1.70	0.70	110.9	35.6	40.1	12.8	36.5	6.8
METSIM cases	1,204	60.5	6.6	30.2	5.2	1.75	0.06	92.4	17.1	26.5	9.7	28.2	6.6	-	-	-	-	-	-	-	-	-	-	-	-	-
METSIM controls	957	53.9	5.0	26.4	3.4	1.77	0.06	82.5	11.9	17.3	5.9	20.5	4.6	-	-	-	-	-	-	-	-	-	-	-	-	-
Whitehall metabochip	1,689	60.5	5.8	26.7	3.6	1.75	0.07	81.9	12.4	20.4	7.2	24.3	5.4	534	60.6	5.8	27.1	5.3	1.62	0.06	70.8	14.5	26.5	9.9	36.3	6.8

* only report descrptives for the individuals included in the GWAS/MetaboChip analyses

Supplementary Table 4. Loci reaching genome-wide significance ($P < 5 \times 10^{-8}$) for body fat percentage in all ancestry analysis

SNP	Chr.	Position (bp)	Nearby gene	Effect (Fat% increasing) allele	Effect allele freqency[b]	Other allele	All ancestry GWAS Per allele change in body fat %[c] β	All ancestry GWAS Per allele change in body fat %[c] SE	All ancestry GWAS P	All ancestry GWAS N	All ancestry GWAS Heterogeneity P	All ancestry Metabochip Per allele change in body fat %[c] β	All ancestry Metabochip Per allele change in body fat %[c] SE	All ancestry Metabochip P	All ancestry Metabochip N	All ancestry Metabochip Heterogeneity P
rs1558902	16	52,361,075	*FTO*	A	40%	T	0.048	0.006	7.0E-18	74,802	0.04	0.058	0.009	4.0E-11	24,526	0.28
rs2943652	2	226,816,690	*IRS1*[a]	C	36%	T	0.040	0.006	1.2E-12	74,808	0.02	0.018	0.009	0.04	24,515	0.01
rs6567160	18	55,980,115	*MC4R*	C	25%	T	0.037	0.006	3.5E-09	76,082	0.70	0.027	0.010	0.009	24,560	0.90
rs6755502	2	625,721	*TMEM18*	C	83%	T	0.042	0.007	3.5E-09	75,306	0.09	0.031	0.012	0.007	24,549	0.52
rs6738627	2	165,252,696	*COBLL1-GRB14*	A	37%	G	0.032	0.006	5.4E-07	55,628	0.27	0.027	0.009	0.003	24,568	0.58
rs693839	13	79,856,289	*SPRY2*[a]	C	32%	T	0.029	0.006	5.0E-07	75,643	0.31	0.027	0.009	0.004	24,547	0.19
rs6857	19	50,084,094	*TOMM40-APOE*	C	83%	T	0.050	0.009	8.8E-09	65,417	0.01	0.026	0.027	0.34	3,440	0.91
rs4788099	16	28,763,228	*TUFM-SH2B1*	G	38%	A	0.025	0.006	1.1E-05	76,108	0.89	0.033	0.009	1.9E-04	24,551	0.99
rs9906944	17	44,446,419	*IGF2BP1*	C	67%	T	0.033	0.006	2.9E-08	74,338	0.05	-	-	-	-	
rs543874	1	176,156,103	*SEC16B*	G	19%	A	0.024	0.007	3.7E-04	76,135	0.74	0.049	0.011	5.0E-06	24,570	0.58
**Loci identified in sex-specific all ancestry analyses**																
rs3761445	22	36,925,357	*PICK1-PLA2G6*	G	41%	A	0.022	0.006	4.3E-05	75,592	1.9E-04	0.029	0.009	8.4E-04	24,022	0.50
rs757318	19	18,681,308	*CRTC1*	C	50%	A	0.022	0.006	7.0E-05	74,310	0.37	0.030	0.009	6.0E-04	24,504	0.02

SNP	All ancestry - Men GWAS Per allele change in body fat %[c] β	All ancestry - Men GWAS Per allele change in body fat %[c] SE	All ancestry - Men GWAS P	All ancestry - Men GWAS N	All ancestry - Men GWAS Heterogeneity P	All ancestry - Men Metabochip Per allele change in body fat %[c] β	All ancestry - Men Metabochip Per allele change in body fat %[c] SE	All ancestry - Men Metabochip P	All ancestry - Men Metabochip N	All ancestry - Men Metabochip Heterogeneity P
rs1558902	0.049	0.008	9.6E-11	38,228	1.1E-03	0.054	0.012	7.1E-06	13,270	0.57
rs2943652	0.058	0.008	6.8E-14	38,228	0.01	0.018	0.012	0.15	13,264	0.19
rs6567160	0.047	0.008	1.1E-08	39,094	0.79	0.025	0.014	0.08	13,286	0.93
rs6755502	0.028	0.010	4.3E-03	38,495	0.32	0.022	0.016	0.17	13,283	0.56
rs6738627	0.033	0.009	3.5E-04	26,406	0.30	0.039	0.012	0.002	13,292	0.56
rs693839	0.033	0.008	2.4E-05	38,631	0.75	0.037	0.013	0.004	13,275	0.18
rs6857	0.036	0.012	1.9E-03	34,503	0.03	0.022	0.040	0.59	1,365	0.56
rs4788099	0.029	0.008	1.2E-04	39,107	0.86	0.039	0.012	1.2E-03	13,278	0.90
rs9906944	0.025	0.008	2.9E-03	38,242	0.04	-	-	-	-	
rs543874	0.016	0.009	8.1E-02	39,118	0.41	0.058	0.015	9.1E-05	13,292	0.83
**Loci identified in sex-specific all ancestry analyses**										
rs3761445	0.034	0.007	5.3E-06	38,605	0.01	0.047	0.012	6.8E-05	13,082	0.49
rs757318	0.013	0.008	7.7E-02	38,224	0.62	0.010	0.012	0.41	13,260	0.01

SNP	All ancestry - Women GWAS Per allele change in body fat %[c] β	All ancestry - Women GWAS Per allele change in body fat %[c] SE	All ancestry - Women GWAS P	All ancestry - Women GWAS N	All ancestry - Women GWAS Heterogeneity P	All ancestry - Women Metabochip Per allele change in body fat %[c] β	All ancestry - Women Metabochip Per allele change in body fat %[c] SE	All ancestry - Women Metabochip P	All ancestry - Women Metabochip N	All ancestry - Women Metabochip Heterogeneity P
rs1558902	0.045	0.008	5.5E-09	37,230	0.67	0.063	0.013	1.3E-06	11,256	0.12
rs2943652	0.025	0.008	1.5E-03	37,236	0.62	0.019	0.013	0.16	11,251	3.2E-03
rs6567160	0.029	0.009	8.6E-04	37,644	0.68	0.029	0.015	0.050	11,274	0.55
rs6755502	0.055	0.010	4.2E-08	37,467	0.11	0.042	0.017	0.014	11,266	0.38
rs6738627	0.032	0.009	3.0E-04	29,877	0.48	0.013	0.013	0.33	11,276	0.60
rs693839	0.022	0.008	5.1E-03	37,668	0.28	0.017	0.014	0.22	11,272	0.34
rs6857	0.062	0.012	6.4E-07	31,569	0.11	0.029	0.037	0.43	2,075	0.84
rs4788099	0.023	0.008	3.0E-03	37,656	0.83	0.026	0.013	0.046	11,273	0.98
rs9906944	0.036	0.008	1.5E-05	36,751	0.14	-	-	-	-	
rs543874	0.036	0.010	1.6E-04	37,673	0.88	0.039	0.016	0.013	11,278	0.24
**Loci identified in sex-specific all ancestry analyses**										
rs3761445	0.020	0.008	0.011	37,174	0.07	0.008	0.013	0.56	10,940	0.81
rs757318	0.031	0.008	1.1E-04	36,742	0.34	0.052	0.013	3.7E-05	11,244	0.85

Chr., Chromosome; Position(bp) according to Build 36; allele coding based on the positive strand; and β is the effect size expressed in SD/allele (i.e. following inverse normal transformation to mean of 0 and SD of 1)

[a]Loci first reported in the previous genome-wide association study of body fat percentage (Kilpeläinen etal, 2011. PMID: 21706003).

[b]Effect allele frequency from all ancestry GWAS and Metabochip joint meta-analysis.

Supplementary Table 5. Association between 12 loci (identified in all ancestry analyses reaching genome-wide significance) and body fat percentage in European-ancestry GWAS + ExomeChip analyses

							European-Ancestry Sex-combined					European-Ancetsry - Men					European-Ancestry - Women					Sex-difference
SNP	Chr.	Position (bp)	Nearest gene	Fat% increasing allele	Effect allele frequency[a]	Other allele	Per allele change in body fat %[c] β	SE	P	Explained variance	N	Per allele change in body fat %[c] β	SE	P	Explained variance	N	Per allele change in body fat %[c] β	SE	P	Explained variance	N	*P*
rs1558902	16	52,361,075	*FTO*	A	41%	T	0.051	0.005	1.1E-25	0.127%	87,924	0.051	0.007	1.5E-13	0.124%	43,514	0.051	0.007	1.0E-13	0.125%	45,066	1.00
rs2943652	2	226,816,690	*IRS1*	C	37%	T	0.030	0.005	1.3E-09	0.042%	87,914	0.044	0.007	2.0E-10	0.091%	43,507	0.020	0.007	3.3E-03	0.019%	45,063	0.013
rs6567160	18	55,980,115	*MC4R*	C	24%	T	0.030	0.006	7.6E-08	0.033%	89,234	0.038	0.008	1.4E-06	0.053%	44,398	0.027	0.008	7.2E-04	0.026%	45,492	0.32
rs6755502	2	625,721	*TMEM18*	C	83%	T	0.035	0.006	5.5E-08	0.034%	88,535	0.019	0.009	3.2E-02	0.010%	43,827	0.049	0.009	3.3E-08	0.068%	45,364	0.016
rs6738627	2	165,252,696	*COBLL1-GRB14*	A	37%	G	0.030	0.005	1.8E-08	0.042%	76,338	0.034	0.007	4.6E-06	0.053%	38,247	0.026	0.007	5.5E-04	0.030%	38,746	0.41
rs693839	13	79,856,289	*SPRY2*	C	31%	T	0.030	0.005	9.3E-09	0.038%	88,773	0.038	0.007	1.9E-07	0.061%	43,919	0.020	0.007	4.4E-03	0.018%	45,510	0.063
rs6857	19	50,084,094	*TOMM40-APOE*	C	83%	T	0.053	0.009	6.8E-10	0.077%	60,210	0.040	0.012	7.6E-04	0.044%	29,282	0.062	0.012	2.0E-07	0.106%	31,583	0.18
rs4788099	16	28,763,228	*TUFM-SH2B1*	G	40%	A	0.026	0.005	8.2E-08	0.033%	89,242	0.031	0.007	5.3E-06	0.046%	44,397	0.024	0.007	4.5E-04	0.027%	45,500	0.47
rs9906944	17	44,446,419	*IGF2BP1*	C	66%	T	0.035	0.006	1.9E-08	0.056%	64,032	0.028	0.009	1.7E-03	0.036%	30,580	0.036	0.009	3.5E-05	0.058%	34,107	0.52
rs543874	1	176,156,103	*SEC16B*	G	19%	A	0.031	0.006	5.3E-07	0.029%	89,287	0.026	0.009	2.4E-03	0.020%	44,422	0.038	0.009	9.8E-06	0.043%	45,521	0.33
**Loci identified in sex-specific analyses**																						
rs3761445	22	36,925,357	*PICK1-PLA2G6*	G	40%	A	0.024	0.005	1.4E-06	0.027%	88,707	0.037	0.007	8.6E-08	0.065%	43,886	0.018	0.007	8.3E-03	0.015%	45,008	0.050
rs757318	19	18,681,308	*CRTC1*	C	51%	A	0.024	0.005	1.2E-06	0.029%	87,402	0.010	0.007	1.4E-01	0.005%	43,498	0.037	0.007	7.5E-08	0.068%	44,560	0.005

Chr., Chromosome; Position(bp) according to Build 36; allele coding based on the positive strand; and β is the effect size expressed in SD/allele (i.e. following inverse normal transformation to mean of 0 and SD of 1)

[a]Based on European-ancestry sex-combined analysis

Supplementary Table 6. Loci reaching genome-wide significance ($P < 5 \times 10^{-8}$) for body fat percentage in European-ancestry only analysis

							European-Ancestry Sex-combined											
							GWAS				Metabochip				GWAS + Metabochip			
					Fat% increasing allele		Per allele change in body fat %[c]		P	N	Per allele change in body fat %[c]		P	N	Per allele change in body fat %[c]		P	N
SNP	Chr.	Position (bp)	Nearby gene	Fat% increasing allele	frequency[b]	Other allele	β	SE			β	SE			β	SE		
rs1558902	16	52,361,075	*FTO*	A	41%	T	0.048	0.006	1.1E-16	64,496	0.059	0.009	9.3E-11	23,428	0.051	0.005	1.1E-25	87,924
rs2943650	2	226,814,165	*IRS1*[a]	C	37%	T	0.037	0.006	3.4E-10	65,778	-	-	-	-	0.037	0.006	3.6E-10	65,778
rs6857	19	50,084,094	*TOMM40-APOE*	C	83%	T	0.055	0.009	9.2E-10	57,871	0.029	0.028	0.31	2,339	0.053	0.009	6.8E-10	60,210
rs693839	13	79,856,289	*SPRY2*[a]	C	31%	T	0.030	0.006	1.2E-06	65,337	0.030	0.010	0.002	23,436	0.030	0.005	9.3E-09	88,773
rs6738627	2	165,252,696	*COBLL1-GRB14*	A	37%	G	0.032	0.007	1.2E-06	52,879	0.026	0.009	0.005	23,459	0.030	0.005	1.8E-08	76,338
rs9906944	17	44,446,419	*IGF2BP1*	C	66%	T	0.035	0.006	2.1E-08	64,032	-	-	-	-	0.035	0.006	1.9E-08	64,032
rs6728726	2	613,976	*TMEM18*	C	84%	T	0.038	0.008	1.7E-06	65,823	0.034	0.012	0.005	23,443	0.037	0.007	2.9E-08	89,287

	European-Ancestry - Men											
	GWAS				Metabochip				GWAS + Metabochip			
	Per allele change in body fat %[c]		P	N	Per allele change in body fat %[c]		P	N	Per allele change in body fat %[c]		P	N
SNP	β	SE			β	SE			β	SE		
rs1558902	0.049	0.008	3.9E-09	30,566	0.054	0.012	7.2E-06	12,948	0.051	0.007	1.5E-13	43,514
rs2943650	0.058	0.008	3.1E-12	31,425	-	-	-	-	0.058	0.008	3.3E-12	31,425
rs6857	0.041	0.012	9.3E-04	28,241	0.029	0.040	0.479	1,041	0.040	0.012	7.6E-04	29,282
rs693839	0.038	0.009	1.1E-05	30,969	0.036	0.013	0.005	12,950	0.038	0.007	1.9E-07	43,919
rs6738627	0.032	0.009	5.8E-04	25,279	0.038	0.012	0.002	12,968	0.034	0.007	4.6E-06	38,247
rs9906944	0.028	0.009	0.002	30,580	-	-	-	-	0.028	0.009	0.002	30,580
rs6728726	0.023	0.011	0.042	31,453	0.027	0.016	0.098	12,962	0.024	0.009	0.009	44,415

	European-Ancestry - Women												Sex-difference
	GWAS				Metabochip				GWAS + Metabochip				
	Per allele change in body fat %[c]		P	N	Per allele change in body fat %[c]		P	N	Per allele change in body fat %[c]		P	N	*P*
SNP	β	SE			β	SE			β	SE			
rs1558902	0.046	0.008	1.3E-08	34,586	0.065	0.013	8.2E-07	10,480	0.051	0.007	1.0E-13	45,066	0.98
rs2943650	0.022	0.008	6.1E-03	35,009	-	-	-	-	0.022	0.008	0.006	35,009	0.001
rs6857	0.065	0.013	1.8E-07	30,285	0.028	0.039	0.462	1,298	0.062	0.012	2.0E-07	31,583	0.18
rs693839	0.020	0.008	1.7E-02	35,024	0.022	0.014	0.124	10,486	0.020	0.007	0.004	45,510	0.082
rs6738627	0.032	0.009	3.8E-04	28,255	0.012	0.013	0.359	10,491	0.026	0.007	5.5E-04	38,746	0.42
rs9906944	0.036	0.009	3.2E-05	34,107	-	-	-	-	0.036	0.009	3.5E-05	34,107	0.53
rs6728726	0.052	0.011	2.0E-06	35,027	0.042	0.017	0.015	10,481	0.049	0.009	9.8E-08	45,508	0.054

Chr., Chromosome; Position(bp) according to Build 36; allele coding based on the positive strand; and β is the effect size expressed in SD/allele (i.e. following inverse normal transformation to mean of 0 and SD of 1)

[a]Loci first reported in the previous genome-wide association study of body fat percentage (Kilpeläinen etal, 2011. PMID: 21706003)

[b]Effect allele frequency from European-ancestry sex-combined GWAS and Metabochip joint meta-analysis.

Supplementary Table 7. Association of genome-wide significant body fat percentage loci with adiposity and anthropometric traits

SNP	Chr.	Position (bp)	Nearby gene	Fat% increasing allele	Other allele	Analysis	BMI[a,b]				WHR$_{adjBMI}$[a,b]				SAT[c]			VAT[c]			VAT/SAT[c]			Leptin[d]				height[a,b]			
							β/allele	SE	P value	N	β/allele	SE	P value	N	Direction	P value	N	Direction	P value	N	Direction	P value	N	β/allele	SE	P value	N	β/allele	SE	P value	N
rs1558902	16	52,361,075	*FTO*	A	T	Sex-combined	0.081	0.003	***1.1E-156***	336,974	0.0039	0.004	0.26	208,248	+	***6.2E-07***	10,557	+	***4.6E-04***	10,557	-	0.17	10,556	0.033	0.006	***1.8E-07***	32,041	-0.010	0.003	0.0012	252,880
rs2943652	2	226,816,690	*IRS1*	C	T	Sex-combined	0.014	0.003	***2.4E-06***	335,472	0.0001	0.004	0.99	208,227	+	9.2E-04	10,557	+	0.60	10,557	-	***4.2E-04***	10,556	0.020	0.007	0.002	32,124	-0.003	0.003	0.38	253,041
rs6567160	18	55,980,115	*MC4R*	C	T	Sex-combined	0.056	0.004	***6.7E-59***	339,006	-0.0025	0.004	0.54	209,911	+	0.09	10,557	+	0.13	10,557	-	0.89	10,556	0.027	0.008	***3.9E-04***	32,057	0.025	0.004	***2.0E-12***	253,015
rs6755502	2	625,721	*TMEM18*	C	T	Sex-combined	0.060	0.004	***2.0E-53***	338,935	-0.0084	0.005	0.07	209,871	+	***6.1E-05***	10,557	+	0.05	10,557	-	0.34	10,556	0.026	0.008	0.002	32,030	0.006	0.004	0.16	251,807
rs6738627	2	165,252,696	*COBLL1-GRB14*	A	G	Sex-combined	0.011	0.003	6.1E-04	284,773	-0.0213	0.004	***2.2E-08***	174,672	+	0.02	10,557	+	0.51	10,557	-	0.06	10,556	0.036	0.007	***8.3E-07***	25,573	0.001	0.003	0.72	197,773
rs693839	13	79,856,289	*SPRY2*	C	T	Sex-combined	0.010	0.003	0.003	332,804	0.0022	0.004	0.55	203,655	+	0.009	10,557	+	0.08	10,557	-	0.60	10,556	0.012	0.007	0.10	29,472	0.007	0.003	0.04	252,245
rs6857	19	50,084,094	*TOMM40-APOE*	C	T	Sex-combined	0.021	0.005	***1.0E-04***	231,865	0.0235	0.006	***1.2E-04***	141,664	+	0.004	10,557	+	***2.1E-04***	10,557	+	0.37	10,556	0.026	0.011	0.016	26,284	-0.006	0.005	0.17	236,911
rs4788099	16	28,763,228	*TUFM-SH2B1*	G	A	Sex-combined	0.031	0.003	***1.1E-24***	339,148	0.0024	0.004	0.49	209,997	+	0.04	10,557	+	0.36	10,557	-	0.61	10,556	0.027	0.006	***3.9E-05***	32,158	0.002	0.003	0.42	253,217
rs9906944	17	44,446,419	*IGF2BP1*	C	T	Sex-combined	0.010	0.004	0.02	234,710	0.0096	0.005	0.04	141,155	+	0.70	10,557	+	0.24	10,557	+	0.19	10,556	0.013	0.007	0.07	29,470	-0.016	0.003	***1.1E-06***	245,664
rs543874	1	176,156,103	*SEC16B*	G	A	Sex-combined	0.050	0.004	***2.3E-40***	339,078	-0.0017	0.004	0.69	209,984	+	0.10	10,557	+	0.53	10,557	-	0.25	10,556	0.009	0.008	0.28	32,081	0.006	0.004	0.09	253,117
rs3761445	22	36,925,357	*PICK1-PLA2G6*	G	A	Sex-combined	0.009	0.003	0.002	335,397	-0.0015	0.004	0.66	209,748	+	0.04	10,557	+	0.003	10,557	+	0.25	10,556	0.016	0.006	0.015	32,153	0.012	0.003	***6.7E-05***	252,071
						Men	0.012	0.004	0.003	152,464	0.0027	0.005	0.60	93,329	+	0.13	4,997	+	0.02	4,997	+	0.11	4,997	0.016	0.010	0.12	13,459	0.016	0.005	8.0E-04	101,283
						Women	0.006	0.004	0.14	171,752	-0.0048	0.005	0.29	116,552	+	0.22	5,560	+	0.09	5,560	+	0.83	5,559	0.016	0.008	0.051	18,694	0.010	0.004	0.02	138,353
rs757318	19	18,681,308	*CRTC1*	C	A	Sex-combined	0.017	0.003	***3.2E-08***	338,517	-0.0049	0.003	0.15	208,780	+	0.52	10,557	+	0.56	10,557	+	0.56	10,556	-0.004	0.007	0.59	32,124	0.003	0.003	0.38	251,032
						Men	0.013	0.004	0.002	152,563	-0.0017	0.005	0.73	92,844	-	0.26	4,997	-	0.66	4,997	+	0.33	4,997	-0.009	0.011	0.41	13,454	0.005	0.005	0.31	100,758
						Women	0.021	0.004	***1.7E-07***	171,630	-0.0080	0.005	0.08	116,069	+	0.11	5,560	+	0.12	5,560	+	0.70	5,559	-0.001	0.008	0.92	18,670	0.003	0.004	0.53	137,848

Chr., Chromosome; EA, Effect-allele (body fat percentage increasing allele); NEA, non-effect allele; EAF, Effect allele frequency; Position(bp) according to Build 36; and allele coding based on the positive strand.

[a]Assocation results were derived from latest Genetic Investigation of ANthropometric Traits consortium (GIANT) GWAS and Metabochip meta analyses; β is the effect size expressed in SD/allele (i.e. following inverse normal transformation to mean of 0 and SD of 1) for BMI and WHRadjBMI (waist-to-hip ratio adjusted by BMI) and z-score transformed (i.e. mean of 0 and SD of 1) for height.

[b]Association results were derived from European-ancestry analyses except sex-combined BMI analysis (from all ancestry analysis).

[c]Association results for abdominal subcutaneous fat (SAT), viceral fat (VAT), and ratio of visceral fat to subcutaneous fat (VAT/SAT) were obtained from Subcutaneous adipose tissue (SAT)-Visceral adipose tissue (VAT) consortium (Fox et al., 2012 PMID:22589738). "Direction" indicates where the fat% increasing allele is associated with increased (+) or decreased (-) SAT, VAT or VAT/SAT.

[d]Association results for ciculating leptin were obtained from circulating leptin consoritum (Kilpeläinen et al, in preparation); β is effect size per allele expressed in natural logarithm-transformed (ng/ml).

Supplementaty Table 8. Heterogeneity between all ancestry analyses and European analyses for 12 loci idenitified in all ancestry analyses (*P* < 5E-8)

SNP	Chr.	Position (bp)	Nearby gene	Fat% increasing allele	Other allele	All ancestry sex-combined					European-ancestry sex-combined					Heterogeneity
						EAF	Per allele change in body fat %[c] β	SE	P	N	EAF	Per allele change in body fat %[c] β	SE	P	N	*P*
rs1558902	16	52,361,075	*FTO*	A	T	40%	0.051	0.005	3.8E-27	99,328	41%	0.051	0.005	1.1E-25	87,924	1.00
rs2943652	2	226,816,690	*IRS1*	C	T	36%	0.034	0.005	1.5E-12	99,323	37%	0.030	0.005	1.3E-09	87,914	0.30
rs6567160	18	55,980,115	*MC4R*	C	T	25%	0.034	0.005	1.3E-10	100,642	24%	0.030	0.006	7.6E-08	89,234	0.36
rs6755502	2	625,721	*TMEM18*	C	T	83%	0.039	0.006	1.4E-10	99,855	83%	0.035	0.006	5.5E-08	88,535	0.39
rs6738627	2	165,252,696	*COBLL1-GRB14*	A	G	37%	0.030	0.005	5.7E-09	80,196	37%	0.030	0.005	1.8E-08	76,338	1.00
rs693839	13	79,856,289	*SPRY2*	C	T	32%	0.028	0.005	6.6E-09	100,190	31%	0.030	0.005	9.3E-09	88,773	0.61
rs6857	19	50,084,094	*TOMM40-APOE*	C	T	83%	0.048	0.008	6.8E-09	68,857	83%	0.053	0.009	6.8E-10	60,210	0.45
rs4788099	16	28,763,228	*TUFM-SH2B1*	G	A	38%	0.027	0.005	1.2E-08	100,659	40%	0.026	0.005	8.2E-08	89,242	0.80
rs9906944	17	44,446,419	*IGF2BP1*	C	T	67%	0.033	0.006	2.9E-08	74,338	66%	0.035	0.006	1.9E-08	64,032	0.67
rs543874	1	176,156,103	*SEC16B*	G	A	19%	0.032	0.006	4.5E-08	100,705	19%	0.031	0.006	5.3E-07	89,287	0.83
rs3761445	22	36,925,357	*PICK1-PLA2G6*	G	A	41%	0.024	0.005	1.7E-07	99,614	40%	0.024	0.005	1.4E-06	88,707	1.00
rs757318	19	18,681,308	*CRTC1*	C	A	50%	0.024	0.005	2.1E-07	98,814	51%	0.024	0.005	1.2E-06	87,402	1.00

SNP	All ancestry Men					European-Ancetsry Men					Heterogeneity
	EAF	Per allele change in body fat %[c] β	SE	P	N	EAF	Per allele change in body fat %[c] β	SE	P	N	*P*
rs1558902	40%	0.051	0.0064	3.8E-15	51,498	41%	0.051	0.007	1.5E-13	43,514	1.00
rs2943652	36%	0.046	0.0065	1.3E-12	51,492	36%	0.044	0.007	2.0E-10	43,507	0.70
rs6567160	26%	0.042	0.0072	6.1E-09	52,380	23%	0.038	0.008	1.4E-06	44,398	0.50
rs6755502	83%	0.027	0.0084	0.002	51,778	83%	0.019	0.009	3.2E-02	43,827	0.23
rs6738627	37%	0.035	0.0073	1.9E-06	39,698	37%	0.034	0.007	4.6E-06	38,247	0.86
rs693839	33%	0.034	0.0067	3.6E-07	51,906	31%	0.038	0.007	1.9E-07	43,919	0.45
rs6857	84%	0.035	0.0112	0.002	35,868	83%	0.040	0.012	7.6E-04	29,282	0.58
rs4788099	38%	0.032	0.0064	6.7E-07	52,385	40%	0.031	0.007	5.3E-06	44,397	0.85
rs9906944	68%	0.025	0.0083	0.003	38,242	66%	0.028	0.009	0.002	30,580	0.65
rs543874	19%	0.028	0.0079	3.7E-04	52,410	19%	0.026	0.009	0.002	44,422	0.76
rs3761445	42%	0.037	0.0063	2.5E-09	51,687	40%	0.037	0.007	8.6E-08	43,886	1.00
rs757318	50%	0.012	0.0064	0.05	51,484	50%	0.010	0.007	0.14	43,498	0.70

SNP	All ancestry Women					European-Ancestry Women					Heterogeneity
	EAF	Per allele change in body fat %[c] β	SE	P	N	EAF	Per allele change in body fat %[c] β	SE	P	N	*P*
rs1558902	41%	0.050	0.0067	7.2E-14	48,486	41%	0.051	0.007	1.0E-13	45,066	0.81
rs2943652	36%	0.023	0.0068	5.6E-04	48,487	37%	0.020	0.007	0.003	45,063	0.47
rs6567160	24%	0.029	0.0076	1.1E-04	48,918	24%	0.027	0.008	7.2E-04	45,492	0.67
rs6755502	83%	0.052	0.0087	2.7E-09	48,733	83%	0.049	0.009	3.3E-08	45,364	0.57
rs6738627	37%	0.026	0.0072	3.8E-04	41,153	37%	0.026	0.007	5.5E-04	38,746	1.00
rs693839	32%	0.021	0.0069	0.002	48,940	31%	0.020	0.007	0.004	45,510	0.81
rs6857	84%	0.058	0.0118	7.3E-07	33,644	84%	0.062	0.012	2.0E-07	31,583	0.58
rs4788099	39%	0.024	0.0067	3.6E-04	48,929	40%	0.024	0.007	4.5E-04	45,500	1.00
rs9906944	67%	0.036	0.0084	1.5E-05	36,751	66%	0.036	0.009	3.5E-05	34,107	1.00
rs543874	19%	0.037	0.0081	5.8E-06	48,951	19%	0.038	0.009	9.8E-06	45,521	0.85
rs3761445	40%	0.017	0.0066	0.013	48,114	40%	0.018	0.007	0.008	45,008	0.81
rs757318	50%	0.037	0.0067	4.8E-08	47,986	51%	0.037	0.007	7.5E-08	44,560	1.00

SNP	All ancestry BIA					All ancestry DEXA					Heterogeneity
	EAF	Per allele change in body fat %[c] β	SE	P	N	EAF	Per allele change in body fat %[c] β	SE	P	N	*P*
rs1558902	41%	0.049	0.0058	9.2E-18	62,832	40%	0.051	0.008	8.9E-11	35,323	0.86
rs2943652	36%	0.033	0.0058	1.1E-08	62,837	36%	0.035	0.008	9.2E-06	35,313	0.84
rs6567160	25%	0.037	0.0066	3.0E-08	62,837	24%	0.029	0.009	1.1E-03	36,632	0.45
rs6755502	83%	0.044	0.0077	1.5E-08	62,621	83%	0.030	0.010	2.9E-03	36,061	0.27
rs6738627	37%	0.032	0.0069	3.5E-06	44,615	37%	0.026	0.008	1.1E-03	34,408	0.61
rs693839	32%	0.025	0.006	4.6E-05	62,868	31%	0.036	0.008	8.2E-06	36,149	0.25
rs6857	84%	0.036	0.0107	6.8E-04	42,204	84%	0.068	0.013	9.9E-08	25,480	0.06
rs4788099	38%	0.030	0.0058	3.0E-07	62,853	38%	0.021	0.008	7.8E-03	36,633	0.35
rs9906944	68%	0.023	0.0082	4.5E-03	39,663	67%	0.046	0.009	1.4E-07	33,501	0.06
rs543874	19%	0.028	0.0072	9.8E-05	62,899	20%	0.039	0.010	4.0E-05	36,633	0.36
rs3761445	42%	0.028	0.0057	6.5E-07	62,296	40%	0.019	0.008	1.1E-02	36,150	0.34
rs757318	49%	0.023	0.0058	8.9E-05	61,024	50%	0.031	0.008	7.3E-05	36,623	0.42

Chr., Chromosome; Position(bp) according to Build 36; allele coding based on the positive strand; and β is the effect size expressed in SD/allele (i.e. following inverse normal transformation to mean of 0 and SD of 1)

EAF: Effect Allele Frequency

Supplementary Table 9. Association of genome-wide significant body fat percentage loci with plasma liver enzymes and C reative protein levels

SNP	Chr.	Position (bp)	Nearby gene	Fat% increasing allele	Other allele	Effect allele freq. (%)	ALP[a]				ALT[a]				AST[a]				GGT[a]				CRP[b]			
							β/allele	SE	P value	N	β/allele	SE	P value	N	β/allele	SE	P value	N	β/allele	SE	P value	N	β/allele	SE	P value	N_{max}
rs1558902	16	52,361,075	*FTO*	A	T	42%	0.002	0.001	0.25	32,573	0.003	0.002	0.10	55,382	-0.001	0.002	0.62	40,202	0.004	0.002	0.06	57,589	0.032	0.007	***2.2E-06***	66,185
rs2943652	2	226,816,690	*IRS1*	C	T	39%	0.002	0.001	0.14	32,631	-0.003	0.002	0.11	55,461	-0.002	0.002	0.35	40,214	-0.003	0.002	0.22	57,665	-0.010	0.007	0.14	66,185
rs6567160	18	55,980,115	*MC4R*	C	T	27%	0.003	0.001	0.06	32,541	0.001	0.002	0.50	55,349	0.002	0.002	0.35	40,170	0.000	0.003	0.97	57,573	0.029	0.008	***1.5E-04***	66,185
rs6755502	2	625,721	*TMEM18*	C	T	83%	0.002	0.002	0.31	32,554	0.001	0.002	0.56	55,351	0.004	0.003	0.09	40,128	0.003	0.003	0.34	57,561	0.038	0.008	***8.2E-06***	66,185
rs6738627	2	165,252,696	*COBLL1-GRB14*	A	G	38%	-0.001	0.003	0.78	12,031	-0.007	0.003	0.02	30,529	-0.006	0.002	0.02	30,191	-0.009	0.003	0.01	30,923	-0.002	0.009	0.84	66,185
rs693839	13	79,856,289	*SPRY2*	C	T	30%	0.000	0.001	0.97	32,535	0.003	0.002	0.15	55,318	0.002	0.002	0.47	40,204	-0.001	0.002	0.64	57,548	0.004	0.007	0.52	66,185
rs6857	19	50,084,094	*TOMM40-APOE*	C	T	84%	0.002	0.002	0.32	27,307	0.007	0.003	0.015	50,119	-0.001	0.003	0.74	35,558	-0.001	0.004	0.77	50,562	0.209	0.010	***7.4E-95***	66,185
rs4788099	16	28,763,228	*TUFM-SH2B1*	G	A	39%	-0.001	0.001	0.57	32,618	0.000	0.002	0.98	55,449	-0.001	0.002	0.79	40,203	0.000	0.002	0.89	57,653	0.004	0.006	0.56	66,185
rs9906944	17	44,446,419	*IGF2BP1*	C	T	69%	0.002	0.001	0.30	32,179	-0.003	0.002	0.12	50,660	0.000	0.002	0.92	35,566	-0.003	0.003	0.32	52,884	0.025	0.007	***4.5E-04***	66,185
rs543874	1	176,156,103	*SEC16B*	G	A	20%	0.004	0.002	0.040	32,574	0.002	0.002	0.29	55,387	0.000	0.003	0.93	40,220	0.005	0.003	0.09	57,606	0.020	0.008	0.011	66,185
rs3761445	22	36,925,357	*PICK1-PLA2G6*	G	A	41%	0.001	0.001	0.46	32,591	-0.003	0.002	0.07	55,424	-0.003	0.002	0.17	40,178	-0.002	0.002	0.42	57,630	0.017	0.006	0.011	66,185
rs757318	19	18,681,308	*CRTC1*	C	A	52%	0.000	0.001	0.90	32,304	0.002	0.002	0.16	55,135	-0.001	0.002	0.69	39,893	0.003	0.002	0.17	57,341	0.010	0.006	0.12	66,185

Chr., Chromosome; EA, Effect-allele (body fat percentage increasing allele); NEA, non-effect allele; EAF, Effect allele frequency; Position(bp) according to Build 36; allele coding based on the positive strand; the association results were derived from sex-combined analyses.

[a]Association results for liver enzymes (ALP, alkaline phosphatase; ALT, alanine transaminase; AST, aspartate aminotransferase; and GGT, gamma-glutamyl transferase) were obtained from Chambers et al., 2011 (PMID:22001757); β represents the per-allele change expressed in z-score (Mean of 0; SD of 1).

[b]Association results for C-Reactive protein levels (CRP) were derived from Dehghan et al., 2011 (PMID: 21300955); the β represents the per-allele change expressed as natural logarithmic transformation (mg/L).

Supplementary Table 10. Association of genome-wide significant body fat percentage loci with with pubertal height growth

SNP	Chr.	Position (bp)	Nearby gene	Fat% increasing allele	Other allele	Analysis[a]	Girls (10yrs) and boys (12yrs)			Girls (10yrs)			Boys (12yrs)		
							β/allele	P value	N_{max}	β/allele	P value	N_{max}	β/allele	P value	N_{max}
rs9906944	17	44,446,419	*IGF2BP1*	C	T	Pre-pubertal height	-0.013	0.34	13,956	-0.022	0.23	6,974	-0.003	0.87	6,982
						Pubertal growth	-0.013	0.37	10,799	-0.026	0.19	5,756	0.002	0.92	5,043
						Pubertal timing	-0.007	0.67	9,228	-0.008	0.73	4,946	-0.006	0.81	4,282
rs3761445	22	36,925,357	*PICK1-PLA2G6*	G	A	Pre-pubertal height	0.034	0.0072	13,948	0.029	0.09	6,968	0.038	0.03	6,980
						Pubertal growth	-0.004	0.77	10,786	0.003	0.86	5,750	-0.013	0.53	5,036
						Pubertal timing	0.024	0.12	9,224	0.044	0.03	4,946	0.000	0.99	4,278

Chr., Chromosome; EA, Effect-allele (body fat percentage increasing allele); NEA, non-effect allele; EAF, Effect allele frequency; Position(bp) according to Build 36; and allele coding based on the positive strand.

[a]Association results for pubertal height growth were obtained from the Early Growth Genetics Consortium (Cousminer et al., 2013. PMID:23449627); height in 10y-old girls and 12y-old boys; pubertal growth was calculated as the difference in height of 8y-old girls/boys and their adult height; pubertal timing was calculated as the difference in height of 14y-old girls/boys and their adult height. Height, growth and timing were all standerdized to the sex-specific population means.

Supplementary Table 11. Previously-reported genome-wide significant associations listed in the NHGRI GWAS Catalog that lie within 500 kb and r2 > 0.7 to each of the 12 body fat percentage lead SNPs.

										Body fat% SNP		Previously Identified SNP					
Region	Body Fat% SNP (BFS)	BFS Position	r^2	Previously Identified SNPs (PIS)	PIS Position	PMID	First Author	Disease/Trait	Previously Reported Gene(s)	Effect Allele	EAF	Effect Allele	EAF	OR/β	Direction of Effect for body fat % Increasing Allele (+/-)	PIS p-Value	Distance between BFS and PIS
1q25.2	rs543874	176,156,103	0.92	rs633715	176,119,203	21102462	Elks CE	Menarche (age at onset)	*SEC16B*	G	19%	C	20%	-2.6	-	2E-08	36,900
1q25.2	rs543874	176,156,103	1.00	rs543874	176,156,103	20935630	Speliotes EK	Body mass index	*SEC16B*	G	19%	G	19%	0.22	+	4E-23	0
1q25.2	rs543874	176,156,103	0.96	rs10913469	176,180,142	19079260	Thorleifsson G	Weight	*SEC16B, RASAL2*	G	19%	C	20%	3.82	+	1E-08	24,039
2p25.3	rs6755502	625,721	1.00	rs2947411	604,168	21102462	Elks CE	Menarche (age at onset)	*TMEM18*	C	83%	A	17%	2.8	-	2E-08	21,553
2p25.3	rs6755502	625,721	1.00	rs2867125	612,827	20935630	Speliotes EK	Body mass index	*TMEM18*	C	83%	C	83%	0.31	+	3E-49	12,894
2p25.3	rs6755502	625,721	0.85	rs6548238	624,905	19079261	Willer CJ	Body mass index	*TMEM18*	C	83%	C	84%	0.26	+	1E-18	816
2p25.3	rs6755502	625,721	1.00	rs7561317	634,953	19079260	Thorleifsson G	Body mass index	*TMEM18*	C	83%	G	84%	6.12	+	4E-17	9,232
2p25.3	rs6755502	625,721	1.00	rs7561317	634,953	19079260	Thorleifsson G	Weight	*TMEM18*	C	83%	G	84%	6.47	+	2E-18	9,232
2q24.3	rs6738627	165,252,696	0.93	rs3923113	165,210,095	21874001	Kooner JS	Type 2 diabetes	*GRB14*	A	37%	A	74%	1.09	-	1E-08	42,601
2q24.3	rs6738627	165,252,696	0.72	rs10195252	165,221,337	20935629	Heid IM	Waist-hip ratio	*GRB14*	A	37%	T	60%	0.05	-	4E-34	31,359
2q24.3	rs6738627	165,252,696	0.72	rs10195252	165,221,337	20686565	Teslovich TM	Triglycerides	*COBLL1*	A	37%	C	40%	-2.01	-	2E-10	31,359
2q36.3	rs2943652	226,816,690	0.77	rs2943634	226,776,324	22581228	Manning AK	Fasting insulin-related traits (interaction with BMI)	*IRS1*	C	36%	C	66%	0.018	-	2E-14	40,366
2q36.3	rs2943652	226,816,690	0.97	rs2943641	226,801,989	19734900	Rung J	Type 2 diabetes and other traits	*LOC64673, IRS1*	C	36%	C	63%	1.19	-	9E-12	14,701
2q36.3	rs2943652	226,816,690	1.00	rs2972146	226,808,942	20686565	Teslovich TM	HDL cholesterol	*IRS1*	C	36%	C	37%	0.46	+	2E-09	7,748
2q36.3	rs2943652	226,816,690	1.00	rs2972146	226,808,942	20686565	Teslovich TM	Triglycerides	*IRS1*	C	36%	C	37%	-1.89	-	2E-08	7,748
2q36.3	rs2943652	226,816,690	0.97	rs2943650	226,814,165	21706003	Kilpelainen TO	Adiposity	*IRS1*	C	36%	T	64%	-0.03	+	4E-11	2,525
2q36.3	rs2943652	226,816,690	0.93	rs925735	226,887,874	22479202	Dastani Z	Adiponectin levels	*IRS1*	C	36%	G	64%	-0.02	+	2E-08	71,184
16p11.2	rs4788099	28,763,228	0.76	rs151181	28,398,018	21102463	Franke A	Crohn's disease	*IL27, SH2B1, EIF3C, LAT, CD19*	G	38%	G	39%	1.07	+	2E-11	365,210
16p11.2	rs4788099	28,763,228	0.80	rs4788084	28,447,349	19430480	Barrett JC	Type 1 diabetes	*IL27*	G	38%	G	42%	1.09	+	3E-13	315,879
16p11.2	rs4788099	28,763,228	0.80	rs4788084	28,447,349	21829393	Plagnol V	Type 1 diabetes autoantibodies	*IL27*	G	38%	G	NR	1.15	+	1E-08	315,879
16p11.2	rs4788099	28,763,228	0.97	rs8049439	28,745,016	19915574	Imielinski M	Inflammatory bowel disease (early onset)	*IL27, CCDC101, CLN3, EIF3C, NUPR1, SULT1A1, SULT1A2*	G	38%	G	37%	1.14	+	2E-09	18,212
16p11.2	rs4788099	28,763,228	1.00	rs7498665	28,790,742	19079260	Thorleifsson G	Body mass index	*SH2B1, ATP2A1*	G	38%	G	44%	3.63	+	3E-10	27,514
16p11.2	rs4788099	28,763,228	1.00	rs7498665	28,790,742	19079260	Thorleifsson G	Weight	*SH2B1, ATP2A1*	G	38%	G	44%	3.69	+	1E-09	27,514
16p11.2	rs4788099	28,763,228	1.00	rs7498665	28,790,742	19079261	Willer CJ	Body mass index	*SH2B1*	G	38%	G	41%	0.15	+	5E-11	27,514
16p11.2	rs4788099	28,763,228	1.00	rs7359397	28,793,160	20935630	Speliotes EK	Body mass index	*SH2B1, APOB48R, SULT1A2, AC138894.2, ATXN2L, TUFM*	G	38%	T	40%	0.15	+	2E-20	29,932
16q12.2	rs1558902	52,361,075	0.90	rs9940128	52,358,255	22399527	Kristiansson K	Metabolic syndrome	*FTO*	A	40%	A	42%	0.09	+	2E-09	2,820
16q12.2	rs1558902	52,361,075	1.00	rs1421085	52,358,455	19151714	Meyre D	Obesity	*FTO*	A	40%	C	40%	1.39	+	1E-28	2,620
16q12.2	rs1558902	52,361,075	1.00	rs1558902	52,361,075	19557197	Heard-Costa NL	Waist circumference	*FTO*	A	40%	NR	NR	NR	N/A	5E-19	0
16q12.2	rs1558902	52,361,075	1.00	rs1558902	52,361,075	20421936	Scherag A	Obesity (extreme)	*FTO*	A	40%	A	NA	1.37	+	7E-13	0
16q12.2	rs1558902	52,361,075	1.00	rs1558902	52,361,075	20935630	Speliotes EK	Body mass index	*FTO*	A	40%	A	42%	0.39	+	5E-120	0
16q12.2	rs1558902	52,361,075	0.90	rs1121980	52,366,748	18454148	Loos RJ	Body mass index	*FTO*	A	40%	NR	NR	0.06	N/A	4E-08	5,673
16q12.2	rs1558902	52,361,075	0.93	rs17817449	52,370,868	21552555	Wang K	Obesity	*FTO*	A	40%	NR	NR	NR	N/A	2E-12	9,793
16q12.2	rs1558902	52,361,075	0.93	rs8050136	52,373,776	19079260	Thorleifsson G	Body mass index	*FTO*	A	40%	A	41%	8.04	+	1E-47	12,701
16q12.2	rs1558902	52,361,075	0.93	rs8050136	52,373,776	19079260	Thorleifsson G	Weight	*FTO*	A	40%	A	41%	7.05	+	5E-36	12,701
16q12.2	rs1558902	52,361,075	0.93	rs8050136	52,373,776	19056611	Timpson NJ	Type 2 diabetes	*FTO*	A	40%	NR	NR	1.3	N/A	2E-17	12,701
16q12.2	rs1558902	52,361,075	0.93	rs8050136	52,373,776	17463248	Scott LJ	Type 2 diabetes	*FTO*	A	40%	A	38%	1.17	+	1E-12	12,701
16q12.2	rs1558902	52,361,075	0.93	rs8050136	52,373,776	17463249	Zeggini E	Type 2 diabetes	*FTO*	A	40%	A	40%	1.23	+	7E-14	12,701
16q12.2	rs1558902	52,361,075	0.93	rs8050136	52,373,776	21037115	Wan ES	Body mass in chronic obstructive pulmonary disease (BMI)	*FTO*	A	40%	A	39%	+	+	4E-08	12,701
16q12.2	rs1558902	52,361,075	0.93	rs8050136	52,373,776	21706003	Kilpelainen TO	Adiposity	*FTO*	A	40%	C	60%	-0.06	+	3E-26	12,701
16q12.2	rs1558902	52,361,075	0.93	rs9939609	52,378,028	21102462	Elks CE	Menarche (age at onset)	*FTO*	A	40%	A	40%	-2.1	-	3E-08	16,953
16q12.2	rs1558902	52,361,075	0.93	rs9939609	52,378,028	17434869	Frayling TM	Body mass index	*FTO*	A	40%	A	39%	0.36	+	2E-20	16,953
16q12.2	rs1558902	52,361,075	0.93	rs9939609	52,378,028	19079261	Willer CJ	Body mass index	*FTO*	A	40%	A	41%	0.33	+	4E-51	16,953
16q12.2	rs1558902	52,361,075	0.93	rs9939609	52,378,028	22693455	Perry JR	Type 2 diabetes	*FTO*	A	40%	A	40%	1.25	+	1E-20	16,953
16q12.2	rs1558902	52,361,075	0.97	rs7202116	52,379,116	22982992	Yang J	Body mass index	*FTO*	A	40%	G	40%	0.04	+	2E-10	18,041
16q12.2	rs1558902	52,361,075	0.87	rs9941349	52,382,989	19553259	Cotsapas C	Obesity (extreme)	*FTO*	A	40%	T	43%	1.48	+	6E-12	21,914
16q12.2	rs1558902	52,361,075	0.84	rs9930506	52,387,966	17658951	Scuteri A	Obesity-related traits (hip)	*FTO*	A	40%	A	54%	-0.16	+	3E-08	26,891
16q12.2	rs1558902	52,361,075	0.93	rs12149832	52,400,409	22344221	Okada Y	Body mass index	*FTO*	A	40%	A	20%	0.07	+	5E-22	39,334
16q12.2	rs1558902	52,361,075	0.87	rs11642841	52,402,988	20581827	Voight BF	Type 2 diabetes	*FTO*	A	40%	A	NA	1.13	+	3E-08	41,913
18q21.32	rs6567160	55,980,115	1.00	rs571312	55,990,749	20935630	Speliotes EK	Body mass index	*MC4R*	C	25%	A	24%	0.23	+	6E-42	10,634
18q21.32	rs6567160	55,980,115	1.00	rs12967135	56,000,003	20686565	Teslovich TM	HDL cholesterol	*RPS3A, MC4R*	C	25%	A	23%	-0.42	-	7E-09	19,888
18q21.32	rs6567160	55,980,115	1.00	rs17782313	56,002,077	19079261	Willer CJ	Body mass index	*MC4R*	C	25%	C	21%	0.2	+	5E-18	21,962
18q21.32	rs6567160	55,980,115	1.00	rs17782313	56,002,077	18454148	Loos RJ	Body mass index	*MC4R*	C	25%	C	24%	0.05	+	3E-15	21,962
18q21.32	rs6567160	55,980,115	1.00	rs17782313	56,002,077	20881960	Lango Allen H	height	*MC4R*	C	25%	T	76%	-0.028	+	4E-11	21,962
18q21.32	rs6567160	55,980,115	1.00	rs17782313	56,002,077	19151714	Meyre D	Obesity	*MC4R*	C	25%	C	18%	1.22	+	5E-15	21,962
18q21.32	rs6567160	55,980,115	1.00	rs17782313	56,002,077	20881960	Lango Allen H	Height	*MC4R*	C	25%	T	76%	-0.03	+	4E-11	21,962
18q21.32	rs6567160	55,980,115	0.81	rs12970134	56,035,730	19079260	Thorleifsson G	Body mass index	*MC4R*	C	25%	A	30%	4.38	+	1E-12	55,615
18q21.32	rs6567160	55,980,115	0.81	rs12970134	56,035,730	19079260	Thorleifsson G	Weight	*MC4R*	C	25%	A	30%	4.66	+	5E-13	55,615
18q21.32	rs6567160	55,980,115	0.81	rs12970134	56,035,730	18454146	Chambers JC	Waist circumference and related phenotypes	*MC4R*	C	25%	A	36%	0.88	+	2E-09	55,615
19q13.32	rs6857	50,084,094	1.00	rs6857	50,084,094	22005931	Kamboh MI	Alzheimer's disease (age of onset)	*PVRL2*	C	83%	NR	NR	NR	N/A	2E-10	0
19q13.32	rs6857	50,084,094	0.88	rs2075650	50,087,459	23207651	Davies G	Cognitive decline	*TOMM40*	C	83%	G	NA	-0.2	+	2E-08	3,365
19q13.32	rs6857	50,084,094	0.88	rs2075650	50,087,459	21943158	Middelberg RP	Cardiovascular disease risk factors (LDL)	*TOMM40*	C	83%	G	15%	0.15	-	2E-14	3,365
19q13.32	rs6857	50,084,094	0.88	rs2075650	50,087,459	21943158	Middelberg RP	Cardiovascular disease risk factors (CRP)	*TOMM40*	C	83%	G	15%	-0.12	+	4E-08	3,365
19q13.32	rs6857	50,084,094	0.88	rs2075650	50,087,459	21418511	Deelen J	Longevity	*TOMM40, APOE*	C	83%	NA	86%	1.41	+	3E-17	3,365
19q13.32	rs6857	50,084,094	0.88	rs2075650	50,087,459	20885792	Naj AC	Alzheimer's disease (late onset)	*APOE, TOMM40*	C	83%	G	20%	2.94	-	5E-36	3,365
19q13.32	rs6857	50,084,094	0.88	rs2075650	50,087,459	20460622	Seshadri S	Alzheimer's disease	*APOE*	C	83%	G	14%	2.53	-	1E-295	3,365
19q13.32	rs6857	50,084,094	0.88	rs2075650	50,087,459	20061627	Heinzen EL	Alzheimer's disease	*TOMM40, APOE*	C	83%	NR	15%	NR	N/A	3E-11	3,365
19q13.32	rs6857	50,084,094	0.88	rs2075650	50,087,459	19734902	Harold D	Alzheimer's disease	*APOE, TOMM40*	C	83%	NR	15%	2.53	-	2E-157	3,365
19q13.32	rs6857	50,084,094	0.88	rs2075650	50,087,459	19734903	Lambert JC	Alzheimer's disease	*APOE*	C	83%	NR	90%	NR	NA	2E-16	3,365
19q13.32	rs6857	50,084,094	0.88	rs2075650	50,087,459	19060911	Aulchenko YS	Cholesterol, total	*TOMM40, APOE*	C	83%	G	15%	0.14	-	3E-19	3,365

NR - Not reported in previous publication. N/A - Not enough information was provided to determine direction of effect.

Supplementary Table 12. Association of genome-wide significant body fat percentage loci with lipid traits and coronary artery disease risk

SNP	Chr.	Position (bp)	Nearby gene	Fat% increasing allele	Other allele	Analysis	HDL-Cholesterol[a]				LDL-Cholesterol[a]				Total Cholesterol[a]				Triglycerides[a]				CAD[c]		
							β/allele	SE	P value	N	β/allele	SE	P value	N	β/allele	SE	P value	N	β/allele	SE	P value	N	OR/allele	P value	N
rs1558902	16	52,361,075	*FTO*	A	T	Sex-combined	-0.018	0.004	***2.7E-07***	177,879	-0.002	0.004	0.45	165,206	-0.005	0.004	0.13	177,865	0.018	0.004	***2.3E-06***	169,619	1.03	0.008	170,586
rs2943652	2	226,816,690	*IRS1*	C	T	Sex-combined	0.032	0.004	***8.2E-17***	184,147	-0.006	0.004	0.14	170,117	0.000	0.004	0.98	184,310	-0.027	0.003	***1.3E-13***	174,875	0.97	8.8E-04	213,752
rs6567160	18	55,980,115	*MC4R*	C	T	Sex-combined	-0.026	0.004	***2.9E-09***	185,608	0.001	0.004	0.86	171,573	-0.007	0.004	0.12	185,760	0.012	0.004	8.4E-04	176,270	1.03	0.0019	213,556
rs6755502	2	625,721	*TMEM18*	C	T	Sex-combined	-0.013	0.005	0.008	186,633	-0.010	0.005	0.02	172,577	-0.006	0.005	0.14	186,801	0.008	0.004	0.03	177,323	1.03	0.02	213,488
rs6738627	2	165,252,696	*COBLL1-GRB14*	A	G	Sex-combined	0.019	0.004	***4.9E-05***	129,391	-0.012	0.005	0.04	116,028	-0.013	0.004	0.02	129,330	-0.017	0.004	***3.3E-05***	119,979	0.98	0.06	199,002
rs693839	13	79,856,289	*SPRY2*	C	T	Sex-combined	-0.001	0.004	0.91	181,390	0.005	0.004	0.20	167,399	0.000	0.004	0.85	181,568	-0.002	0.004	0.46	172,078	0.99	0.51	206,738
rs6857	19	50,084,094	*TOMM40-APOE*	C	T	Sex-combined	0.067	0.008	***2.6E-17***	86,306	-0.192	0.008	***5.1E-110***	82,050	-0.164	0.008	***1.7E-86***	86,277	-0.054	0.007	***4.6E-19***	82,963	0.90	***5.9E-11***	103,603
rs4788099	16	28,763,228	*TUFM-SH2B1*	G	A	Sex-combined	-0.012	0.004	5.4E-04	185,489	-0.003	0.004	0.41	171,467	-0.008	0.004	0.03	185,637	-0.002	0.003	0.57	176,156	1.01	0.33	213,432
rs9906944	17	44,446,419	*IGF2BP1*	C	T	Sex-combined	-0.012	0.005	0.03	92,820	0.003	0.006	0.39	88,433	-0.001	0.006	0.70	93,067	0.003	0.005	0.84	89,485	1.04	***2.2E-06***	211,220
rs543874	1	176,156,103	*SEC16B*	G	A	Sex-combined	-0.011	0.004	0.02	187,131	-0.010	0.005	0.07	173,055	-0.011	0.004	0.04	187,324	0.004	0.004	0.21	177,828	1.00	0.96	213,938
rs3761445	22	36,925,357	*PICK1-PLA2G6*	G	A	Sex-combined	0.016	0.004	***3.9E-06***	185,166	-0.008	0.004	0.04	171,168	-0.010	0.004	0.01	185,291	-0.023	0.003	***8.1E-12***	175,846	1.00	0.83	201,748
						Men	+		0.05	38,970	-		0.43	36,840	-		0.38	39,104	-		0.0025	35,288	1.01	0.33	165,789
						Women	+		0.04	64,118	-		0.51	61,803	-		0.22	64,235	-		0.02	59,473	1.00	0.88	138,333
rs757318	19	18,681,308	*CRTC1*	C	A	Sex-combined	-0.003	0.004	0.54	183,160	-0.005	0.004	0.23	169,133	-0.006	0.004	0.14	183,303	0.003	0.004	0.31	173,822	1.02	0.18	95,655
						Men	-		0.32	38,970	-		0.40	36,840	-		0.55	39,104	+		0.36	35,288	1.01	0.50	58,594
						Women	-		0.56	64,118	-		0.76	61,803	-		0.96	64,235	+		0.71	59,473	1.03	0.17	35,033

Chr., Chromosome; EA, Effect-allele (body fat percentage increasing allele); NEA, non-effect allele; EAF, Effect allele frequency (EAF for lipid traits derived from the 1000 genome project); Position(bp) according to Build 36; and allele coding based on the positive strand.

[a]Association results for HDL cholesterol (HDL-C), LDL cholesterol (LDL-C), total cholesterol (TC) and triglyceride (TG) levels were obtained from the GLOBAL Lipids Genetics Consortium (GLGC) (Willer et al., 2013. PMID:24097068); β is the effect size expressed in SD/allele (i.e. following inverse normal transformation to mean of 0 and SD of 1). For sex-specific analyses, no effect sizes were available; the "+/-" indicates whether the fat%-increasing allele is associated with increased (+) or decreased (-) lipid levels.

[c]Association results (OR: odds ratio) for risk of coronary artery disease (CAD) were obtained from the CARDIoGRAMplusC4D Consortium (Deloukas et al., 2013. PMID:23202125); the unit in effect for coronary artery disease was odds ratio (OR); the SNP of rs2943646 was used as a proxy for rs2943652 (R^2=1, and D'=1); the SNP of rs2075650 was used as a proxy for rs6857 (R2=0.88, and D'=1); and the proxy SNP (rs4794018) was used for rs9906944 (R^2=0.9, and D'=1).

Supplementary Table 13. Association of genome-wide significant body fat percentage loci with glycemic traits and type 2 diabetes risk

SNP	Chr.	Position (bp)	Nearby gene	Fat% increasing allele	Other allele	Analysis	Fasting glucose[a]				Fasting insulin[a]				Adiponectin$_{adjBMI}$[b]				Type 2 Diabetes[c]		
							β/allele	SE	P value	N	β/allele	SE	P value	N	β/allele	SE	P value	N	OR/allele	P value	N
rs1558902	16	52,361,075	*FTO*	A	T	Sex-combined	0.006	0.002	0.0039	115,626	0.019	0.003	***1.8E-12***	82,970	0.003	0.005	0.47	29,297	1.12	***4.4E-21***	79,535
rs2943652	2	226,816,690	*IRS1*	C	T	Sex-combined	-0.004	0.002	0.084	115,530	-0.015	0.003	***3.8E-08***	82,506	0.022	0.004	***3.1E-06***	29,311	0.92	***4.7E-12***	79,290
rs6567160	18	55,980,115	*MC4R*	C	T	Sex-combined	0.006	0.003	0.030	119,317	0.008	0.003	0.018	85,501	-0.007	0.005	0.18	29,307	1.07	***6.0E-07***	80,620
rs6755502	2	625,721	*TMEM18*	C	T	Sex-combined	0.006	0.003	0.031	117,782	0.007	0.004	0.06	84,051	0.005	0.006	0.37	27,578	1.04	0.0052	86,188
rs6738627	2	165,252,696	*COBLL1-GRB14*	A	G	Sex-combined	-0.001	0.002	0.58	97,004	-0.009	0.003	0.0035	72,356	0.015	0.005	0.008	20,653	0.94	***2.3E-05***	54,640
rs693839	13	79,856,289	*SPRY2*	C	T	Sex-combined	-0.001	0.002	0.83	120,901	0.001	0.003	0.80	82,955	0.003	0.005	0.53	29,287	0.98	0.09	82,279
rs6857	19	50,084,094	*TOMM40-APOE*	C	T	Sex-combined	0.010	0.004	0.012	77,452	0.003	0.005	0.49	56,354	-0.012	0.007	0.09	25,623	1.09	0.0014	31,256
rs4788099	16	28,763,228	*TUFM-SH2B1*	G	A	Sex-combined	0.000	0.002	0.92	116,851	0.008	0.003	0.0028	83,233	-0.002	0.004	0.67	29,345	1.02	0.11	80,650
rs9906944	17	44,446,419	*IGF2BP1*	C	T	Sex-combined	0.002	0.003	0.59	80,010	0.000	0.004	0.97	58,124	0.000	0.005	0.95	29,203	1.05	***7.7E-05***	80,649
rs543874	1	176,156,103	*SEC16B*	G	A	Sex-combined	0.005	0.003	0.04	119,361	0.012	0.003	***5.1E-04***	85,499	0.003	0.006	0.57	29,319	1.02	0.20	86,195
rs3761445	22	36,925,357	*PICK1-PLA2G6*	G	A	Sex-combined	-0.001	0.002	0.72	123,325	-0.004	0.003	0.10	85,340	0.000	0.004	0.96	29,344	0.97	0.012	78,765
						Men	-0.002	0.003	0.50	59,155	-0.009	0.004	0.02	41,299	0.002	0.007	0.80	12,661	0.97	0.035	44,233
						Women	0.000	0.003	0.89	64,170	0.000	0.004	0.95	44,041	-0.002	0.006	0.79	16,683	0.97	0.09	35,771
rs757318	19	18,681,308	*CRTC1*	C	A	Sex-combined	0.003	0.002	0.16	123,332	0.008	0.003	0.0039	85,395	0.005	0.004	0.30	29,344	1.02	0.15	72,259
						Men	0.003	0.003	0.26	59,264	0.007	0.004	0.08	41,363	0.002	0.007	0.73	12,662	1.01	0.60	41,031
						Women	0.003	0.003	0.37	64,068	0.008	0.004	0.02	44,032	0.008	0.006	0.20	16,681	1.03	0.12	31,889

Chr., Chromosome; EA, Effect-allele (body fat percentage increasing allele); NEA, non-effect allele; EAF, Effect allele frequency; Position(bp) according to Build 36; and allele coding based on the positive strand.

[a]Association results for glycemic traits were obtained from the Meta-Analyses of Glucose and Insulin traits (MAGIC) consortium (Scott et al., 2012. PMID:22885924); the β represents the per-allele change in fasting glucose (mmol/L), fasting insulin (natural logarithm-transformed pmol/L).

[b]Association results for adiponectin levels were obtained from AdipoGen Consortium (Dastani et al., 2012. PMID:22479202); β represents the per-allele effect of adiponectin levels (natural logarithmic transformed) adjusted for age, sex, and BMI.

[c]Association results for risk of type 2 diabetes (T2D) were obtained from the DIAGRAM Consortium (Morris et al., 2012. PMID:22885922); the SNP of rs4794018 was used as a proxy for rs9906944 (R^2=0.9, and D'=1) for the sex-combined result.

Supplementary Table 14. Results of conditional analysis of body fat percentage and lipid associations in TOMM40/APOE locus.

	Original meta-analysis result (not conditioned)							Conditional analyses (GCTA)					
**Outcome Trait[a]**	**Target SNP**	**Primary association trait**	**Effect allele**	**Effect allele frequency (%)**	**beta**	**se**	***p*-value**	**Target SNP**	**Conditional on**	**r^2**	**beta**	**se**	***p*-value**
**Body fat percentage (INV) (n = 100,705)**	**rs6857**	**Body fat %**	C	84%	0.048	0.008	6.8E-09	**rs6857**	rs2075650	0.88	0.016	0.006	4.4E-03
	rs2075650	Alzheimer's	A	85%	0.037	0.007	1.4E-07	rs2075650	rs6857		0.008	0.005	0.10
	**rs6857**	**Body fat %**	C	84%	0.048	0.008	6.8E-09	**rs6857**	rs439401	0.04	0.047	0.008	4.2E-09
	rs439401	Triglyceride and HDL-	T	37%	0.002	0.005	0.72	rs439401	rs6857		-0.006	0.005	0.27
	**rs6857**	**Body fat %**	C	84%	0.048	0.008	6.8E-09	**rs6857**	rs4420638	0.39	0.024	0.006	4.4E-05
	rs4420638	LDL-C	A	82%	0.036	0.009	3.9E-05	rs4420638	rs6857		0.000	0.006	1.00
**Trigycerides (INV) (n = 177,828)**	rs439401	Triglyceride and HDL-	T	37%	-0.066	0.004	1.4E-66	rs439401	rs6857	0.04	-0.060	0.004	9.0E-58
	**rs6857**	**Body fat %**	C	84%	-0.054	0.007	4.5E-19	**rs6857**	rs439401		-0.028	0.007	3.6E-05
**HDL-cholesterol levels (INV) (n=187,131)**	rs439401	Triglyceride and HDL-	T	37%	0.027	0.004	2.3E-10	rs439401	rs6857	0.04	0.020	0.004	2.5E-07
	**rs6857**	**Body fat %**	C	84%	0.067	0.008	2.6E-17	**rs6857**	rs439401		0.056	0.008	8.4E-14
**LDL-cholesterol levels (INV) (n=173,055)**	rs4420638	LDL-C	A	82%	-0.225	0.008	1.5E-178	rs4420638	rs6857	0.39	-0.088	0.005	2.0E-73
	**rs6857**	**Body fat %**	C	84%	-0.192	0.008	5.1E-110	**rs6857**	rs4420638		0.000	0.005	0.97

Rs6857 was identified in our meta-analyses as the body fat percentage associated variant; rs439401 was previously identfied for its association with triglyceride and HDL-cholesterol levels; and rs4420638 was previously identified for its association with LDL-cholesterol levels.

INV, inverse-normal transformation (mean of 0, SD of 1);

[a]Summary statistics of lipid associatons were obtained from the GLOBAL Lipids Genetics Consortium (GLGC) (Willer et al., 2013. PMID:24097068)

Supplementary Table 15. Putative coding variants in linkage disequilibrium ($r^2 >$ 0.7) with body fat percentage loci identified in all ancestry analyses ($P < 5x10^{-8}$)

SNP	Chr.	Source	Gene	Position (bp)	Putative Coding Variant	r^2	Protein Alteration	PhastCons Score	GERP Score	Grantham Score	PolyPhen	SIFT Prediction	SIFT Score
rs4788099	16	1000G	*APOBR*	28,414,373	rs149271	0.72	E170E	0.88	2.1	NA	unknown	Tolerated	1
rs4788099	16	1000G	*APOBR*	28,415,570	rs151174	0.79	G569G	0.00	-9.6	NA	unknown	Tolerated	1
rs4788099	16	1000G	*APOBR*	28,415,145	rs180743	0.79	P428A	0.10	0.49	27	unknown	Tolerated	1
rs4788099	16	1000G	*APOBR*	28,415,549	rs180744	0.75	Q562Q	0.00	0.11	NA	unknown	Tolerated	1
rs4788099	16	1000G	*APOBR*	28,415,948	rs40831	0.72	A695A	0.00	0.39	NA	unknown	Tolerated	1
rs4788099	16	1000G	*SH2B1*	28,790,742	rs7498665	1.00	T484A	1.00	3.1	58	benign	Tolerated	1
rs4788099	16	Hapmap	*ATP2A1*	28,806,294	rs6565259	0.70	T226T	0.02	-11.0	NA	unknown	Tolerated	1

Chr., Chromosome; Position(bp) according to Build 36; allele coding based on the positive strand.

r^2 between body fat percentage SNP and putative coding variant

PhastCon Score ranges between 0 and 1 and describes the degree of sequence conservation among 17 vertebrate species [PMID: 16024819].

The Genomic Evolutionary Rate Profiling (GERP) score ranges from -12.3 to 6.17, with 6.17 being the most conserved [PMID: 15965027].

Grantham Score categorizes codon replacements into classes of increasing chemical dissimilarity [PMID: 4843792]

PolyPhen predicts the possible impact of an amino acid substitution on protein structure and function [PMID: 20354512]

SIFT predicts whether an amino acid substitution affects protein function based on the degree of conservation of amino acid residues in sequence alignments derived from closely related sequences [PMID: 19561590].

Supplementary Table 16. Descriptive information of studies used in cis-eQTL analyses

Study	Tissues (n)	Genotype Platform	Gene Expression Platform	Reference
Bariatric Surgery Study	Omental fat (n=742) and subcutaneous fat (n=610)	Illumina 650K	Custom Agilent 44,000 feature microarray composed of 39,280 oligonucleotide probes	PMID: 20346437
MolOBB	Abdominal adipose (n=54) and Gluteal fat (n=65)	Illumina 317K	Affymetrix Human Genome U133 Plus 2.0 gene-expression microarrays (hgu133plus2 arrays) with 17,726 non-overlapping probes.	PMID: 22383892
Eqtl meta-analysis	Whole blood (n=2,360)	Illumina HumanHap300, HumanHap370	Illumina HumanRef-8 v2 or Illumina HumanHT-12 arrays	PMID:24518929, 21829388, 24013639
Cortical brain study	Brain (n=193)	Affymetrix Human Mapping 500K	Illumina Human Mapping Refseq-8 Expression Beadchip	PMID: 17982457

Supplementary Table 17. Nominally significant associations (*P* < 0.05) of the 12 body fat percentage (BFP) index SNPs with *cis* gene expression (*cis*-eQTLs) in subcutaneous adipose tissue, omental adipose tissue, blood cell, and brain from four populations (Associations that reach $P < 10^{-5}$ are in bold italic)

SNP	Chr	BFP increasing allele	Nearest Gene	Tissue	Gene transcript	Direction of effect for BFP increasing allele	*P* for BFP SNP	P_{adj}^{a} for BFP SNP	Peak SNP[b]	$r^{2\,c}$	*P* for peak SNP	P_{adj}^{d} for peak SNP	Reference
rs543874	1	G	*SEC16B*	Omental fat	LZTR2	-	5.06E-04	0.43	rs8030	0.61	***3.68E-08***	0.005	PMID: 20346437
				Omental fat	XM_060215	+	0.007	0.01	rs10798508	0.00	3.38E-04	4.98E-04	PMID: 20346437
				Subcutaneous fat	LZTR2	-	0.007	0.30	rs10913472	0.12	***8.47E-07***	6.50E-05	PMID: 20346437
rs2943652	2	C	*IRS1*	Omental fat	Contig24493_RC	+	1.32E-04	0.79	rs10933137	0.66	1.14E-05	0.20	PMID: 20346437
				Omental fat	Contig50189_RC	+	0.004	0.002	rs3769647	0.00	***3.78E-06***	***2.34E-06***	PMID: 20346437
				Omental fat	ENST00000272907	+	0.01	-	-	-	-	-	PMID: 20346437
				Omental fat	IRS1	+	***3.42E-09***	0.99	rs908252	0.88	***3.98E-10***	0.50	PMID: 20346437
				Omental fat	RHBDD1	+	0.02	-	-	-	-	-	PMID: 20346437
				Subcutaneous fat	BC018684	-	0.001	-	-	-	-	-	PMID: 20346437
				Subcutaneous fat	Contig24493_RC	+	8.67E-05	0.98	rs2943653	0.82	1.20E-05	0.41	PMID: 20346437
				Subcutaneous fat	Contig50189_RC	+	0.03	-	-	-	-	-	PMID: 20346437
				Subcutaneous fat	IRS1	+	2.79E-04	0.67	rs2943653	0.82	***6.44E-06***	0.22	PMID: 20346437
rs6738627	2	A	*COBLL1/GRB14*	Omental fat	AK001808	+	0.02	0.02	rs17194412	0.00	***2.33E-06***	***3.27E-06***	PMID: 20346437
				Omental fat	COBLL1	+	0.03	0.71	rs10221833	0.18	3.13E-05	0.001	PMID: 20346437
				Omental fat	Contig21674_RC	+	5.64E-04	0.93	rs1509103	0.15	***5.17E-19***	***4.39E-14***	PMID: 20346437
				Omental fat	GRB14	-	***5.31E-12***	0.98	rs6717858	0.84	***3.42E-14***	0.21	PMID: 20346437
				Omental fat	SLC38A11	+	1.17E-05	0.94	rs10184126	0.10	***3.04E-46***	***2.23E-37***	PMID: 20346437
				Abdominal Subcutaneous Fat	COBLL1	+	0.04	0.02	rs12997787	-	0.009	5.81E-04	PMID: 22383892
				Subcutaneous fat	COBLL1	-	0.02	0.36	rs3731759	0.27	***1.65E-10***	***3.08E-07***	PMID: 20346437
				Subcutaneous fat	COBLL1	+	0.03	0.19	rs3820981	0.04	3.36E-05	2.16E-04	PMID: 20346437
				Subcutaneous fat	Contig21674_RC	+	2.33E-04	0.27	rs1509103	0.16	***4.79E-11***	***3.26E-07***	PMID: 20346437
				Subcutaneous fat	GRB14	-	3.12E-05	0.95	rs10184004	0.78	***1.66E-06***	0.27	PMID: 20346437
				Subcutaneous fat	SLC38A11	+	***2.29E-06***	0.68	rs10184126	0.11	***4.72E-45***	***2.27E-35***	PMID: 20346437
				Subcutaneous fat	SCN2A2	+	0.02	0.25	rs7601375	0.09	3.56E-05	5.49E-04	PMID: 20346437
				Subcutaneous fat	XM_115683	-	0.04	-	-	-	-	-	PMID: 20346437
rs6755502	2	C	*TMEM18*	Blood	AC092159.2	-	2.96E-04	0.90	rs2683992	0.82	3.84E-05	0.44	PMID:24518929, 21829388, 24013639
				Blood	SH3YL1	-	0.001	0.006	rs7566279	0.00	***7.78E-124***	***7.90E-123***	PMID:24518929, 21829388, 24013639
				Omental fat	Contig15639_RC	+	0.03	-	-	-	-	-	PMID: 20346437
				Omental fat	SH3YL1	-	0.02	0.03	rs10188763	0.00	5.65E-04	8.53E-04	PMID: 20346437
				Subcutaneous fat	BC038187	+	0.04	-	-	-	-	-	PMID: 20346437
				Subcutaneous fat	HSS00208712	-	0.02	-	-	-	-	-	PMID: 20346437
				Subcutaneous fat	PXDN	-	0.02	0.02	rs13419013	0.01	***3.78E-06***	***3.79E-06***	PMID: 20346437
rs693839	13	C	*SPRY2*	Omental fat	HSS00006179	-	0.04	-	-	-	-	-	PMID: 20346437
				Subcutaneous fat	HSS00020342	-	0.03	0.07	rs4885640	0.00	***2.43E-28***	***6.13E-28***	PMID: 20346437
rs4788099	16	G	*TUFM/SH2B1*	Blood	APOBR	+	***2.24E-13***	0.58	rs151181	0.72	***8.67E-18***	0.006	PMID:24518929, 21829388, 24013639
				Blood	ATXN2L	+	4.58E-05	0.98	rs8049439	0.99	3.44E-05	0.88	PMID:24518929, 21829388, 24013639
				Blood	CCDC101	-	***4.49E-58***	0.10	rs480400	0.54	***4.09E-92***	***2.92E-18***	PMID:24518929, 21829388, 24013639
				Blood	CCDC101	-	***7.44E-27***	0.93	rs480400	0.54	***1.74E-51***	***1.62E-13***	PMID:24518929, 21829388, 24013639
				Blood	RP11-264B17.3;RP11-264B17.5;LA	+	2.79E-04	0.15	rs4788115	0.14	***3.90E-06***	0.004	PMID:24518929, 21829388, 24013639
				Blood	RP11-264B17.3;SPNS1	+	***3.19E-164***	0.58	rs8045689	0.57	***9.81E-198***	***6.60E-70***	PMID:24518929, 21829388, 24013639
				Blood	SBK1	-	***2.32E-08***	***1.79E-11***	rs4255791	0.02	***7.59E-13***	***4.71E-16***	PMID:24518929, 21829388, 24013639
				Blood	SULT1A1	-	***1.43E-06***	0.19	rs4788073	0.70	***7.56E-15***	7.06E-05	PMID:24518929, 21829388, 24013639
				Blood	SULT1A1	+	***1.79E-06***	0.99	rs151226	0.54	***1.49E-11***	9.04E-04	PMID:24518929, 21829388, 24013639
				Blood	TUFM	+	***9.81E-198***	0.98	rs8049439	0.99	***9.81E-198***	0.15	PMID:24518929, 21829388, 24013639
				Brain	LOC112869	+	0.009	-	-	-	-	-	PMID: 17982457
				Omental fat	ATXN2L	-	5.11E-05	0.09	rs7201929	0.26	***3.47E-06***	0.01	PMID: 20346437
				Omental fat	NFATC2IP	-	3.14E-04	0.21	rs7201929	0.26	***2.70E-06***	0.004	PMID: 20346437
				Omental fat	SH2B1	-	***5.29E-07***	0.87	rs12928404	0.93	***4.65E-07***	0.83	PMID: 20346437
				Omental fat	SULT1A1	+	3.03E-04	0.14	rs11074904	0.07	***9.34E-19***	***2.20E-15***	PMID: 20346437
				Omental fat	SULT1A1	+	4.75E-04	0.16	rs11074904	0.07	***2.63E-18***	***4.16E-15***	PMID: 20346437
				Omental fat	SULT1A2	+	***7.03E-12***	0.74	rs151181	0.70	***5.63E-15***	4.07E-02	PMID: 20346437
				Omental fat	SULT1A2	+	***7.92E-08***	0.002	rs11074904	0.07	***1.43E-23***	***2.70E-18***	PMID: 20346437
				Omental fat	XPO6	-	0.02	-	-	-	-	-	PMID: 20346437
				Abdominal Subcutaneous Fat	CD19	-	0.005	0.16	16-29008330	-	6.00E-04	0.09	PMID: 22383892
				Gluteal Subcutaneous Fat	CLN3	+	0.03	0.10	rs4788054	-	0.005	0.57	PMID: 22383892
				Gluteal Subcutaneous Fat	IL27	+	0.04	0.02	rs34763945	-	0.02	0.26	PMID: 22383892
				Subcutaneous fat	ATXN2L	-	0.001	-	-	-	-	-	PMID: 20346437
				Subcutaneous fat	CCDC101	+	0.006	0.24	rs11074904	0.06	***2.76E-66***	***2.36E-60***	PMID: 20346437
				Subcutaneous fat	CLN3	+	0.04	-	-	-	-	-	PMID: 20346437
				Subcutaneous fat	EIF3S8	+	0.02	-	-	-	-	-	PMID: 20346437
				Subcutaneous fat	NFATC2IP	-	1.84E-04	0.40	rs7140	0.76	***1.37E-07***	0.05	PMID: 20346437
				Subcutaneous fat	SULT1A2	+	***3.36E-21***	0.52	rs1074631	0.80	***3.93E-23***	0.14	PMID: 20346437
				Subcutaneous fat	TUFM	+	3.12E-04	1.00	rs4788102	1.00	3.12E-04	1.00	PMID: 20346437
				Subcutaneous fat	XM_102475	-	0.001	-	-	-	-	-	PMID: 20346437
rs1558902	16	A	*FTO*	Omental fat	hCT1829686	+	0.04	-	-	-	-	-	PMID: 20346437
				Abdominal Subcutaneous Fat	RBL2	-	0.009	0.02	rs62033401	0.05	9.13E-04	0.10	PMID: 22383892
				Gluteal Subcutaneous Fat	AKTIP	+	0.004	0.25	rs9923147	0.91	0.003	0.31	PMID: 22383892
				Subcutaneous fat	HSS00017995	+	0.02	-	-	-	-	-	PMID: 20346437
				Subcutaneous fat	HSS00128973	-	3.62E-04	5.38E-05	rs3852741	0.01	***1.55E-11***	***2.59E-12***	PMID: 20346437
rs9906944	17	C	*IGF2BP1*	Blood	RP11-463M16.4;ATP5G1	+	***1.63E-13***	0.82	rs11079842	0.35	***2.04E-30***	***1.21E-11***	PMID:24518929, 21829388, 24013639
				Blood	UBE2Z	+	***1.54E-27***	0.14	rs11650158	0.34	***6.02E-89***	***2.87E-38***	PMID:24518929, 21829388, 24013639
				Omental fat	CALCOCO2	+	0.002	0.002	rs8080446	0.00	***4.67E-09***	***3.63E-09***	PMID: 20346437
				Omental fat	CALCOCO2	+	0.03	0.02	rs8080446	0.00	***1.39E-09***	***9.86E-10***	PMID: 20346437
				Omental fat	IGF2BP1	-	0.05	-	-	-	-	-	PMID: 20346437
				Omental fat	SLC35B1	-	0.04	-	-	-	-	-	PMID: 20346437
				Abdominal Subcutaneous Fat	ATP5G1	+	9.66E-04	0.24	17-47293150	-	2.71E-05	0.20	PMID: 22383892
				Abdominal Subcutaneous Fat	HOXB3	-	0.009	0.11	rs8075581	0.03	0.001	0.22	PMID: 22383892
				Gluteal Subcutaneous Fat	ATP5G1	+	0.05	0.50	rs28397663	-	0.02	0.11	PMID: 22383892
				Gluteal Subcutaneous Fat	HOXB2	+	0.01	0.23	rs4793979	0.02	1.98E-04	0.13	PMID: 22383892
				Gluteal Subcutaneous Fat	NGFR	-	0.01	0.48	rs2898857	0.00	0.002	0.09	PMID: 22383892
				Gluteal Subcutaneous Fat	TTLL6	+	0.05	0.20	rs6504411	0.00	0.02	0.52	PMID: 22383892
				Subcutaneous fat	HOXB3	+	0.04	-	-	-	-	-	PMID: 20346437
				Subcutaneous fat	HOXB6	+	0.007	0.003	rs11079830	0.00	***6.58E-08***	***3.55E-08***	PMID: 20346437
				Subcutaneous fat	HOXB7	+	0.05	0.04	rs15689	0.00	8.85E-05	7.36E-05	PMID: 20346437
				Subcutaneous fat	SPOP	-	0.02	-	-	-	-	-	PMID: 20346437
				Subcutaneous fat	TTLL6	+	0.01	0.74	rs12947660	0.23	***3.62E-06***	6.05E-04	PMID: 20346437
rs6567160	18	C	*MC4R*	Abdominal Subcutaneous Fat	MC4R	+	0.010	0.05	rs62095764	-	0.002	0.41	PMID: 22383892
				Brain	PMAIP1	+	0.006	-	-	-	-	-	PMID: 17982457
rs757318	19	C	*CRTC1*	Blood	AC005253.4;KXD1	+	0.002	1.00	rs757318	1.00	0.002	1.00	PMID:24518929, 21829388, 24013639
				Blood	CRLF1;UBA52	-	***5.07E-10***	0.09	rs12609505	0.08	***1.74E-62***	***3.48E-50***	PMID:24518929, 21829388, 24013639
				Blood	ZNF101	-	7.97E-04	1.00	rs757318	1.00	7.97E-04	1.00	PMID:24518929, 21829388, 24013639
				Omental fat	ARMC6	+	0.01	-	-	-	-	-	PMID: 20346437
				Omental fat	C19orf50	+	0.02	0.52	rs10409392	0.11	***1.28E-06***	4.24E-05	PMID: 20346437
				Omental fat	CRLF1	-	0.01	0.72	rs11880800	0.19	***2.50E-11***	***2.22E-08***	PMID: 20346437
				Omental fat	ELL	-	6.39E-04	0.22	rs2303695	0.08	***1.28E-15***	***2.05E-12***	PMID: 20346437

				Omental fat	ENST00000300979	-	0.01	-	-	-	-	-	PMID: 20346437
				Omental fat	KLHL26	+	0.002	0.79	rs3220	0.06	***4.32E-44***	***4.62E-39***	PMID: 20346437
				Omental fat	LRRC25	+	0.04	0.09	rs888663	0.01	***1.95E-06***	***4.42E-06***	PMID: 20346437
				Omental fat	RFXANK	+	0.007	-	-	-	-	-	PMID: 20346437
				Omental fat	SSBP4	+	0.005	-	-	-	-	-	PMID: 20346437
				Omental fat	TM6SF2	+	0.003	0.003	rs8108647	0.00	2.78E-05	2.21E-05	PMID: 20346437
				Omental fat	UBA52	+	***4.54E-10***	0.13	rs2057649	0.11	***1.19E-57***	***1.15E-43***	PMID: 20346437
				Abdominal Subcutaneous Fat	C19orf60	+	0.03	0.15	rs7252628	-	0.005	0.06	PMID: 22383892
				Abdominal Subcutaneous Fat	CRTC1	+	0.007	0.07	rs3795027	0.07	0.001	0.70	PMID: 22383892
				Abdominal Subcutaneous Fat	HOMER3	-	2.99E-05	0.94	rs17751061	-	***1.77E-06***	0.20	PMID: 22383892
				Abdominal Subcutaneous Fat	LSM4	+	0.04	0.51	rs4808743	-	0.02	0.28	PMID: 22383892
				Abdominal Subcutaneous Fat	RFXANK	+	0.005	0.23	rs10410509	0.10	9.85E-04	0.05	PMID: 22383892
				Abdominal Subcutaneous Fat	TMEM161A	-	0.03	0.96	rs10417974	0.10	0.003	0.34	PMID: 22383892
				Gluteal Subcutaneous Fat	C19orf60	+	0.01	0.04	rs11673466	0.01	0.001	0.07	PMID: 22383892
				Gluteal Subcutaneous Fat	LSM4	+	8.97E-04	0.38	rs34688943	-	3.06E-04	0.29	PMID: 22383892
				Subcutaneous fat	ELL	-	0.02	0.96	rs7258465	0.10	***8.04E-16***	***2.78E-13***	PMID: 20346437
				Subcutaneous fat	FCHO1	-	0.002	-	-	-	-	-	PMID: 20346437
				Subcutaneous fat	GDF15	-	0.04	-	-	-	-	-	PMID: 20346437
				Subcutaneous fat	HSS00041008	-	0.05	0.05	rs8108647	0.00	***1.50E-06***	***1.63E-06***	PMID: 20346437
				Subcutaneous fat	KLHL26	+	4.27E-05	0.15	rs3220	0.07	***6.21E-29***	***5.33E-24***	PMID: 20346437
				Subcutaneous fat	NM_016368	-	0.03	0.51	rs748609	0.10	***9.94E-07***	2.32E-05	PMID: 20346437
				Subcutaneous fat	UBA52	+	5.10E-05	0.49	rs2057649	0.12	***1.45E-46***	***2.57E-37***	PMID: 20346437
rs6857	19	C	*TOMM40*	Blood	CTB-129P6.4;PVRL2	+	3.68E-04	0.02	rs11879589	0.01	***9.76E-22***	***1.06E-19***	PMID:24518929, 21829388, 24013639
				Blood	PVRL2	+	5.59E-04	0.45	rs3852857	0.01	***2.91E-113***	***5.91E-109***	PMID:24518929, 21829388, 24013639
				Blood	PVRL2	+	0.001	0.40	rs11879589	0.01	***1.24E-74***	***1.04E-70***	PMID:24518929, 21829388, 24013639
				Omental fat	FOSB	-	0.02	-	-	-	-	-	PMID: 20346437
				Omental fat	TOMM40	+	***3.12E-08***	0.37	rs10119	0.44	***7.28E-13***	3.76E-04	PMID: 20346437
				Abdominal Subcutaneous Fat	BCL3	-	0.04	0.20	rs1661184	-	0.009	0.56	PMID: 22383892
				Abdominal Subcutaneous Fat	GEMIN7	+	0.01	0.06	rs34647054	-	0.001	0.39	PMID: 22383892
				Abdominal Subcutaneous Fat	RELB	+	0.02	0.11	rs439401	-	0.003	0.21	PMID: 22383892
				Abdominal Subcutaneous Fat	TRAPPC6A	+	0.04	0.32	19-45966736	-	0.007	0.62	PMID: 22383892
				Abdominal Subcutaneous Fat	ZNF229	+	0.001	2.29E-04	rs7507903	-	3.87E-05	0.08	PMID: 22383892
				Gluteal Subcutaneous Fat	EXOC3L2	+	0.02	0.02	rs4389234	-	0.001	0.13	PMID: 22383892
				Gluteal Subcutaneous Fat	MARK4	-	0.04	0.10	19-46164967	-	0.004	0.25	PMID: 22383892
				Gluteal Subcutaneous Fat	PPP1R13L	+	0.01	0.007	rs2302593	-	0.003	0.87	PMID: 22383892
				Gluteal Subcutaneous Fat	TRAPPC6A	+	0.03	0.09	rs8111589	-	0.001	0.06	PMID: 22383892
				Subcutaneous fat	GPR4	-	0.05	-	-	-	-	-	PMID: 20346437
				Subcutaneous fat	KLC3	+	0.03	-	-	-	-	-	PMID: 20346437
				Subcutaneous fat	NM_178494	+	0.005	0.01	rs4511641	0.00	***1.47E-06***	***3.04E-06***	PMID: 20346437
				Subcutaneous fat	OPA3	+	0.02	0.03	rs6509224	0.00	***1.43E-06***	***1.93E-06***	PMID: 20346437
				Subcutaneous fat	TOMM40	+	5.38E-05	0.60	rs10119	0.40	***2.22E-08***	0.002	PMID: 20346437
				Subcutaneous fat	ZNF225	-	0.05	0.04	rs3746319	0.00	***1.78E-17***	***1.61E-17***	PMID: 20346437
rs3761445	22	G	*PLA2G6*	Blood	CSNK1E	-	0.002	0.71	rs6001054	0.39	***4.07E-07***	0.003	PMID:24518929, 21829388, 24013639
				Blood	MAFF	+	1.76E-04	1.00	rs3761445	1.00	1.76E-04	1.00	PMID:24518929, 21829388, 24013639
				Blood	TMEM184B	-	***1.32E-11***	0.19	rs2018805	0.18	***3.88E-31***	***7.38E-17***	PMID:24518929, 21829388, 24013639
				Blood	Z83844.1;SH3BP1	-	0.002	0.71	rs732857	0.04	***2.29E-90***	***1.67E-85***	PMID:24518929, 21829388, 24013639
				Brain	FLJ22582	-	0.007	-	-	-	-	-	PMID: 17982457
				Omental fat	TMEM184B	-	***4.35E-09***	0.65	rs4821767	0.76	***4.03E-10***	0.25	PMID: 20346437
				Omental fat	DDX17	-	0.02	0.92	rs2235346	0.49	0.001	0.11	PMID: 20346437
				Omental fat	GGA1	+	0.05	-	-	-	-	-	PMID: 20346437
				Omental fat	H1F0	-	1.94E-04	0.70	rs2285178	0.03	***3.91E-239***	***6.41E-225***	PMID: 20346437
				Omental fat	hCT32985.2	+	0.04	-	-	-	-	-	PMID: 20346437
				Omental fat	HSS00043843	+	0.02	-	-	-	-	-	PMID: 20346437
				Omental fat	MAFF	-	***5.46E-08***	0.80	rs2267375	0.92	***2.73E-09***	0.46	PMID: 20346437
				Omental fat	SLC16A8	-	0.004	0.44	rs3026688	0.11	***6.25E-11***	***2.11E-08***	PMID: 20346437
				Abdominal Subcutaneous Fat	ANKRD54	-	0.003	0.08	rs11089864	-	9.20E-05	0.02	PMID: 22383892
				Abdominal Subcutaneous Fat	BAIAP2L2	-	0.05	0.29	rs4821767	0.65	0.01	0.38	PMID: 22383892
				Abdominal Subcutaneous Fat	H1F0	-	0.05	0.12	22-38647215	-	0.005	0.15	PMID: 22383892
				Abdominal Subcutaneous Fat	PICK1	-	5.32E-04	0.07	rs4821715	0.01	7.42E-05	0.20	PMID: 22383892
				Abdominal Subcutaneous Fat	SLC16A8	-	0.03	0.22	rs17753650	0.02	5.74E-04	0.03	PMID: 22383892
				Gluteal Subcutaneous Fat	C22orf23	-	0.01	0.07	rs2281098	-	0.003	0.68	PMID: 22383892
				Gluteal Subcutaneous Fat	EIF3L	+	0.004	0.22	22-38061115	-	7.77E-04	0.81	PMID: 22383892
				Gluteal Subcutaneous Fat	MAFF	-	0.05	0.51	rs12169607	-	0.009	0.24	PMID: 22383892
				Gluteal Subcutaneous Fat	PICK1	-	0.003	0.006	rs11570672	-	5.96E-04	0.84	PMID: 22383892
				Gluteal Subcutaneous Fat	POLR2F	-	0.008	0.08	22-38253113	-	2.78E-04	0.20	PMID: 22383892
				Gluteal Subcutaneous Fat	SLC16A8	+	5.46E-04	4.16E-04	rs2076372	0.05	***5.14E-06***	0.11	PMID: 22383892
				Gluteal Subcutaneous Fat	TOMM22	-	2.17E-04	0.18	rs6001473	-	2.07E-05	0.67	PMID: 22383892
				Subcutaneous fat	ANKRD54	-	0.006	0.24	rs13053681	0.11	***1.04E-06***	6.66E-05	PMID: 20346437
				Subcutaneous fat	APOBEC3A	+	0.03	-	-	-	-	-	PMID: 20346437
				Subcutaneous fat	BAIAP2L2	-	0.02	-	-	-	-	-	PMID: 20346437
				Subcutaneous fat	TMEM184B	-	***1.34E-10***	0.80	rs4608623	0.89	***1.18E-12***	0.29	PMID: 20346437
				Subcutaneous fat	Contig39180_RC	-	0.01	-	-	-	-	-	PMID: 20346437
				Subcutaneous fat	H1F0	-	5.23E-05	0.18	rs2285178	0.03	***7.68E-199***	***2.13E-185***	PMID: 20346437
				Subcutaneous fat	KDELR3	-	0.003	0.04	rs196084	0.02	***1.55E-12***	***1.92E-11***	PMID: 20346437
				Subcutaneous fat	LGALS2	+	6.10E-04	0.17	rs7291467	0.02	***3.01E-72***	***3.92E-68***	PMID: 20346437
				Subcutaneous fat	MAFF	-	***8.04E-08***	0.99	rs2277844	0.85	***4.98E-09***	0.36	PMID: 20346437
				Subcutaneous fat	NPTXR	-	0.02	0.006	rs13053206	0.01	***2.94E-06***	***1.12E-06***	PMID: 20346437
				Subcutaneous fat	SLC16A8	-	0.01	0.01	rs3952	0.00	***5.44E-07***	***4.09E-07***	PMID: 20346437
				Subcutaneous fat	TRIOBP	-	0.005	0.44	rs5756813	0.03	***8.19E-48***	***7.71E-45***	PMID: 20346437

[a] *P*-value for the body fat percentage SNP after adjusting for the peak SNP.

[b] Most significant SNP associated with the gene transcript.

[c] r^2 between the body fat percentage SNP and the peak SNP.

[d] *P*-value for the peak SNP after adjusting for the Fat% lead SNP.

[e] Specific tissue unknown and z score presented in effect.

Supplementary Table 18. Regulatory data used in body fat percentage epigenomic analysis

ENCODE Consortium

Sample	Tissue	DNaseI HS	H3K4me1	H3K27ac	H3K4me3	H3K9ac	FAIRE	H3K4me2	TF
GM12878	Blood	Integrative	Integrative	Integrative	Integrative (2)	Integrative	Integrative	Integrative	Integrative (75)
GM06990	Blood	Integrative	-	-	Integrative	-	-	-	Integrative(1)
GM12193	Blood	-	-	-	-	-	-	-	Integrative(2)
GM12801	Blood	-	-	-	-	-	-	-	Integrative(1)
GM12864	Blood	Integrative	-	-	-	-	-	-	Integrative(1)
GM12865	Blood	Integrative	-	-	-	-	-	-	Integrative(1)
GM12872	Blood	-	-	-	-	-	-	-	Integrative(1)
GM12873	Blood	-	-	-	-	-	-	-	Integrative(1)
GM12874	Blood	-	-	-	-	-	-	-	Integrative(1)
GM12875	Blood	-	-	-	-	-	-	-	Integrative(1)
GM12891	Blood	Integrative	-	-	-	-	Integrative	-	Integrative(10)
GM12892	Blood	Integrative	-	-	-	-	Integrative	-	Integrative(10)
GM15510	Blood	Integrative	-	-	-	-	-	-	Integrative(1)
GM18505	Blood	-	-	-	-	-	-	-	Integrative(2)
GM18507	Blood	Integrative	-	-	-	-	Integrative		Integrative(1)
GM18951	Blood	-	-	-	-	-	-	-	Integrative(2)
GM19099	Blood	-	-	-	-	-	-	-	Integrative(2)
GM19238	Blood	Integrative	-	-	-	-	-	-	-
GM19239	Blood	Integrative	-	-	-	-	Integrative	-	-
GM19240	Blood	Integrative	-	-	-	-	-	-	-
Astrocytes	Brain	Integrative	Standard	Integrative	Integrative	-	Integrative	-	Integrative (1)
Cerebellum	Brain	Standard	-	-	-	-	-	-	-
Cerebrum Frontal	Brain	Standard	-	-	-	-	-	-	-
Frontal Cortex	Brain	Standard	-	-	-	-	Integrative	-	-
HepG2	Liver	Integrative	Integrative	Integrative	Integrative (2)	Integrative	Integrative	Integrative	Integrative (61)
Hepatocytes	Liver	Integrative	-	-	-	-	-	-	-
Huh-7	Liver	Integrative	-	-	-	-	-	-	-
Pancreatic Islet	Pancreatic Islet	Integrative	-	-	-	-	Integrative	-	-

Roadmap Epigenomics Project

Sample	Tissue	DNaseI HS	H3K4me1	H3K27ac	H3K4me3	H3K9ac	FAIRE	H3K4me2	TF
Adipose Nuclei	Adipose	-	IDR	MACS2	IDR	IDR	-	-	-
Anterior Caudate	Brain	-	IDR	MACS2	IDR	MACS2	-	-	-
Mid Frontal Lobe	Brain	-	IDR	MACS2	IDR	MACS2	-	-	-
Substantia Nigra	Brain	-	IDR	-	IDR	MACS2	-	-	-
Adult Liver	Liver	-	IDR	-	IDR	IDR	-	-	-
Pancreatic Islet	Pancreatic Islet	-	MACS2	-	MACS2	MACS2	-	-	-

Method of analysis for ENCODE (integrative analysis or standard analysis) and Roadmap Epigenomics data (IDR or MAC2 alone) used in epigenomics analysis. Numbers in parentheses indicate the number of datasets when more than one is available. IDR, Irreproducible Discovery Rate; TF, Transcription Factor Binding.

Supplementary Table 19. Regulatory element overlap with variants at body fat percentage-associated loci

SNP	Position	Locus	Nearest TSS (kb)	Nearest Gene	DNase	FAIRE	H3K4me1	H3K27ac	H3K4me3	H3K9ac	H3K4me2	Transcription Factors	Number of Overlaps	Number of Tissues
rs34166952	chr1:177793822	SEC16B	103,798	RP4-798P15.2	N		N	N					3	1
rs6670873	chr1:177841685	SEC16B	55,935	RP4-798P15.2			NL					FOXA1:L	3	2
rs10913469	chr1:177913519	SEC16B	-8,018	SEC16B				N		N			3	1
rs115342375	chr2:605176	TMEM18	46,973	AC093326.3	N	LB							4	3
rs6548238	chr2:634905	TMEM18	40,870	TMEM18	NL		L						3	2
rs35142762	chr2:636790	TMEM18	38,985	TMEM18	NLB		AN	N	AN	ANI			17	5
rs35796073	chr2:636819	TMEM18	38,956	TMEM18	NL		AN	N	AN	ANI			16	4
rs6548240	chr2:636929	TMEM18	38,846	TMEM18	NILB		AN	N	AN	ANI			22	5
rs13387939	chr2:637498	TMEM18	38,277	TMEM18	N		A			AN			4	2
rs13396119	chr2:637508	TMEM18	38,267	TMEM18	NB		A			AN			5	3
rs13388043	chr2:637597	TMEM18	38,178	TMEM18	NB					AN			4	3
rs35913461	chr2:653575	TMEM18	22,200	TMEM18	L		L						2	1
rs35628463	chr2:653581	TMEM18	22,194	TMEM18	L		L						2	1
rs6713419	chr2:165508300	GRB14	-29,942	GRB14			L					MAFk:L	2	1
rs10184004	chr2:165508389	GRB14	-30,031	GRB14			L					MAFk:L	2	1
rs10195252	chr2:165513091	GRB14	31,196	SNORA70F			NL	N					3	2
rs6752978	chr2:165552942	GRB14	-8,655	SNORA70F			L	AL	L		L		5	2
rs12692737	chr2:165554309	GRB14	-10,022	SNORA70F	L	NIB	IL	AL			L	CTCF:BLN RAD21:L FOXA1:L	25	5
rs9653282	chr2:165554658	GRB14	-10,371	SNORA70F		I	IL	AL			L		6	3
rs7609045	chr2:165555207	GRB14	-10,920	SNORA70F			IL	AL			L		5	3
rs2198550	chr2:165556760	GRB14	-12,473	SNORA70F			IL	L					3	2
rs6712203	chr2:165557318	GRB14	-13,031	SNORA70F		I	L	L					3	2
rs13423088	chr2:227026864	IRS1	23,223	AC068138.1			L	AL					3	2
rs2943651	chr2:227047414	IRS1	2,673	AC068138.1	L		L	L					3	1
rs2943653	chr2:227047771	IRS1	2,316	AC068138.1			L	AL					3	2
rs2943635	chr2:227077377	IRS1	-27,290	AC068138.1			NL	AN					4	3
rs2943656	chr2:227121918	IRS1	-71,831	AC068138.1			N	N					2	1
rs1515110	chr2:227122216	IRS1	-72,129	AC068138.1		I	N	N					3	2
rs2673135	chr2:227151596	IRS1	-101,509	AC068138.1			L					FOXA1:L	2	1
rs2673136	chr2:227164653	IRS1	-114,566	AC068138.1								MAFk:L MAFf:L	3	1
rs2713556	chr2:227172598	IRS1	-122,511	AC068138.1			IL		I		L		4	2
rs35251159	chr13:80975547	SPRY2	-31,878	RNU6-61		B						TBP:B	2	1
rs9545447	chr13:81078769	SPRY2	-135,100	RNU6-61				A	A				2	1
rs149299	chr16:28485141	SH2B1	-3,273	NPIPL1	B							EBF:B EBF1C8:B	7	1
rs27741	chr16:28504181	SH2B1	-558	CLN3	I		B	B	ALB	B	LB		10	4
rs149271	chr16:28506872	SH2B1	-6	CLN3	N		LB	B	AB	NB	B		9	4
rs26528	chr16:28517709	SH2B1	446	IL27			ALB		ALB		LB		10	3
rs153109	chr16:28519096	SH2B1	-941	IL27		B	ALB	B	ALB		LB		12	3
rs62034319	chr16:28532188	SH2B1	-14,033	IL27			L				L	TR4:L	3	1
rs4788085	chr16:28535003	SH2B1	15,326	NUPR1			ALB	A			B		5	3
rs28772958	chr16:28535305	SH2B1	15,024	NUPR1			ALB	A	L	L			6	3
rs62034323	chr16:28536473	SH2B1	13,856	NUPR1			ALB	AL	L	L			7	3
rs62034324	chr16:28536762	SH2B1	13,567	NUPR1			AL	AL	L	L	L		7	2
rs2106480	chr16:28537971	SH2B1	12,358	NUPR1		L	AL	AL	L	L	L	CEBPb:L FOXA2:L USF1:L cJUN:L RXRA:L HNF4a:L FOXA1:L SP1:L HDAC2:L P300:L HNF4g:L	25	2
rs62034325	chr16:28538640	SH2B1	11,689	NUPR1			AL	L	L	L	L		8	2
rs62034326	chr16:28539293	SH2B1	11,036	NUPR1			L	L			L		4	1
rs4788084	chr16:28539848	SH2B1	10,481	NUPR1	L	L	L	L		L	L	EBF:B	9	2
rs12446550	chr16:28543381	SH2B1	6,948	NUPR1			ALB	AL	L	L	L		9	3
rs4788083	chr16:28545449	SH2B1	4,880	NUPR1			AL	A					5	2
rs3785354	chr16:28550667	SH2B1	-172	NUPR1	N		ANL	ANL	ANL	ANL	L		23	3
rs1074631	chr16:28554108	SH2B1	-3,613	NUPR1			LB	N	L	N	L	EBF:B	8	3
rs4787457	chr16:28555400	SH2B1	-4,905	NUPR1			L				L		2	1
rs7191618	chr16:28565667	SH2B1	421	CCDC101	NL	B	ANLB	ANLB	ANLB	ANILB	LB	HEY1:L	40	5
rs3859172	chr16:28566158	SH2B1	912	CCDC101		B	ANLB	NLB	ANILB	ANILB	LB		36	5
rs2077031	chr16:28603168	SH2B1	4,633	SULT1A2	N								2	1
rs113208333	chr16:28606160	SH2B1	1,641	SULT1A2			L	L			L		3	1
rs4115668	chr16:28607532	SH2B1	269	SULT1A2	NL		L	L	AL	L	L		10	3
rs762633	chr16:28608341	SH2B1	27	SULT1A2	NIL	L	L	L	AL	NL	L	TBP:L HEY1:L HDAC2:L TAF1:L ELF1:L POL2:L	22	4
rs35175818	chr16:28608746	SH2B1	-316	SULT1A2	NL		L	L	AL	L	L		12	3
rs4149398	chr16:28608938	SH2B1	-508	SULT1A2	NIB		L	L	AL	L	L		11	5
rs62031562	chr16:28609329	SH2B1	-899	SULT1A2	LB	L	L	L	AL	L	L	HEY1:L HNF4g:L FOXA1:L POL2:L	15	3
rs4788095	chr16:28831359	SH2B1	1,995	RP11-1348G14.5			L	L				POL2:L	4	1
rs72793809	chr16:28832382	SH2B1	972	RP11-1348G14.5			NL	L		AL	LB		8	4
rs62036620	chr16:28833097	SH2B1	257	RP11-1348G14.5			ANL	NLB	NLB	ANLB	LB		17	4
rs62036621	chr16:28833299	SH2B1	55	RP11-1348G14.5			ANL	NLB	NLB	ANLB	LB		20	4
rs4451951	chr16:28836205	SH2B1	1,633	ATXN2L	B		ANLB	ANLB	ANILB	ANILB	LB		31	5
rs62036622	chr16:28837203	SH2B1	2,631	ATXN2L			ANILB	LB	NLB	AILB	LB		19	5
rs8049439	chr16:28837515	SH2B1	2,943	ATXN2L			ANILB	L	NILB	ANLB	LB	CTCF:LN	19	5
rs8062405	chr16:28837906	SH2B1	3,334	ATXN2L			ANILB	L	NILB	ALB	LB		16	5
rs61737565	chr16:28855522	SH2B1	2,207	TUFM			NB		N	A	B		5	3
rs4788099	chr16:28855727	SH2B1	2,002	TUFM			NB		NB	AL	B		7	4
rs7187776	chr16:28857645	SH2B1	84	TUFM	NB	ILB	L	ANLB	ANLB	ANILB	LB	TBP:BL TAF1:B PU1:B ELF1:BL SIN3ak:L POL2:BL GABP:BL YY1:B SP1:B HEY1:L NFkb:B EGR1:B GRP20:L RXF5n:L	74	5
rs4788102	chr16:28873398	SH2B1	-726	SH2B1	L		B	ANB	B	ANB	B		11	4

rs62037367	chr16:28874547	SH2B1	114	RP11-22P6.2	NL	B	A	ANLB	ANILB	ANILB	LB	OCT2:B POUF1:B POL2:B	37	5
rs7198606	chr16:28875122	SH2B1	-3	SH2B1	NILB	B	A	ANLB	ANILB	ANILB	LB	TBP:L TAF1:BL NRF1:B ELF1:BL POL2:BL GABP:L	60	5
rs11864750	chr16:28875204	SH2B1	6	SH2B1	NL	B	A	ANLB	ANLB	ANILB	LB	TBP:L TAF1:L NRF1:B ELF1:BL POL2:BL GABP:BL	44	5
rs7193733	chr16:28875482	SH2B1	187	SH2B1	NLB		ANLB	ANLB	ANLB	ANILB	LB		36	5
rs11864107	chr16:28885931	SH2B1	-3,794	ATP2A1	N							BATF:B	3	2
rs3888190	chr16:28889486	SH2B1	-239	ATP2A1	NL		B				B		5	3
rs62037371	chr16:28890131	SH2B1	323	ATP2A1	N		NLB		B	AB	B	POL2:L	10	4
rs8055138	chr16:28891465	SH2B1	92	ATP2A1	N	B	ANLB	B	ANB	ANB	B	SP1:B	23	4
rs7185232	chr16:28933468	SH2B1	3,049	RABEP2	B		L	L					3	2
rs9937521	chr16:53799296	FTO	61,203	FTO			L	L					2	1
rs28562191	chr16:53799303	FTO	61,210	FTO			L	L					2	1
rs9937053	chr16:53799507	FTO	61,414	FTO			L	L		L	L		4	1
rs9937354	chr16:53799847	FTO	61,754	FTO			L	L		L	L		4	1
rs9928094	chr16:53799905	FTO	61,812	FTO			NL	L		L	L		5	2
rs9930333	chr16:53799977	FTO	61,884	FTO			NL	L		L	L		5	2
rs9930397	chr16:53799985	FTO	61,892	FTO			NL	L		L	L		5	2
rs9940278	chr16:53800200	FTO	62,107	FTO			NL	L		L	L		5	2
rs9939973	chr16:53800568	FTO	62,475	FTO	L		NL	NL		L	L	USF1:L USF:L RXRA:L HNF4a:L MAFk:L JUND:L P300:L TCF4:L FOSL2:L	17	2
rs9940646	chr16:53800629	FTO	62,536	FTO	L	N	NL	NL		L	L	MAFk:L JUND:L USF:L RXRA:L P300:L HNF4a:L TCF4:L FOSL2:L	16	2
rs9940128	chr16:53800754	FTO	62,661	FTO	NI		NL	NL		L	L	JUND:L TCF4:L	10	3
rs1421085	chr16:53800954	FTO	62,861	FTO			NL	NL		L	L		6	2
rs1421086	chr16:53801343	FTO	63,250	FTO			NL	NL		L	L		6	2
rs9923147	chr16:53801549	FTO	63,456	FTO			NL	L		L			4	2
rs9923544	chr16:53801985	FTO	63,892	FTO			NL	NL		N			5	2
rs11642015	chr16:53802494	FTO	64,401	FTO			NL	NL				JUND:L BATF:B	7	3
rs1558901	chr16:53803187	FTO	65,094	FTO			NIL	NL					5	3
rs62048402	chr16:53803223	FTO	65,130	FTO			NIL	NL					5	3
rs1558902	chr16:53803574	FTO	65,481	FTO			NL	L					3	2
rs11075985	chr16:53805207	FTO	67,114	FTO			N						3	1
rs56094641	chr16:53806453	FTO	68,360	FTO		N	NL	A		N			6	3
rs55872725	chr16:53809123	FTO	71,030	FTO			NL	A			L	PU1:B	8	4
rs1121980	chr16:53809247	FTO	71,154	FTO			NL	A			L	PU1:B	8	4
rs7187250	chr16:53810546	FTO	72,453	FTO			NIL	AN			L		7	4
rs7193144	chr16:53810686	FTO	72,593	FTO		I	NIL	AN			L		8	4
rs62033399	chr16:53810943	FTO	72,850	FTO			NIL	AN			L		7	4
rs62033400	chr16:53811788	FTO	73,695	FTO		N	IL	A				MAFk:L	7	4
rs8063057	chr16:53812433	FTO	74,340	FTO			L	A		N			4	3
rs17817449	chr16:53813367	FTO	75,274	FTO	N		L						3	2
rs8043757	chr16:53813450	FTO	75,357	FTO			L						2	1
rs9972653	chr16:53814363	FTO	76,270	FTO			NL	N					3	2
rs17817497	chr16:53815435	FTO	77,342	FTO			NIL					cJUN:L	4	3
rs7202116	chr16:53821615	FTO	83,522	FTO								MAFk:L MAFf:L	2	1
rs79994966	chr16:53823727	FTO	85,634	FTO		N	N						2	1
rs12149574	chr16:53840525	FTO	-80,303	FTO			NI			N			6	2
rs12149832	chr16:53842908	FTO	-77,920	FTO			NI	N		N			6	2
rs9909861	chr17:47079416	IGF2BP1	4,292	IGF2BP1			NL	NL	N	L	L		7	2
rs12945020	chr17:47082775	IGF2BP1	7,651	IGF2BP1	IL	L	L	L	N	L	L	FOXA1:L	10	3
rs35051752	chr17:47089908	IGF2BP1	1,179	RP11-501C14.6			NL	L	L	L	L		7	2
rs11079849	chr17:47090785	IGF2BP1	302	RP11-501C14.6	NLB		NL	L	L	L	L		11	3
rs9906710	chr17:47091283	IGF2BP1	-196	RP11-501C14.6	N		NL	L	L	L	L	CHD2:L	9	2
rs9906944	chr17:47091420	IGF2BP1	-333	RP11-501C14.6	NLB		NL	L		L	L		8	3
rs4794018	chr17:47093398	IGF2BP1	-2,311	RP11-501C14.6	L		NL						3	2
rs9902512	chr17:47094274	IGF2BP1	-3,187	RP11-501C14.6	NIL		NL						8	3
rs8068981	chr17:47122525	IGF2BP1	8,812	U6			N	N					2	1
rs1523136	chr17:47144532	IGF2BP1	30,819	U6	B		L	L	L	L	L		6	2
rs940088	chr17:47145848	IGF2BP1	32,135	U6			NL	L	L	L	L		7	2
rs35693910	chr18:57913965	MC4R	83,166	U4	B	B	B				B		7	1
rs7258722	chr19:18808915	CRTC1	14,491	CRTC1	N	B	B			N			4	2
rs4808844	chr19:18811966	CRTC1	17,542	CRTC1	NIB	B	ANLB	N	ANB	ANIB	B	POL2:B	43	5
rs4808845	chr19:18812024	CRTC1	17,600	CRTC1	NIB	B	ANLB	N	ANB	ANIB	B	POL2:B	41	5
rs757318	chr19:18820308	CRTC1	25,884	CRTC1	NB		A						5	3
rs9304955	chr19:18824870	CRTC1	30,446	CRTC1	N		L	L	L		L		7	2
rs11880870	chr19:18830704	CRTC1	36,280	CRTC1	N		N			N			5	1
rs10404726	chr19:18834514	CRTC1	40,090	CRTC1	N			N					2	1
rs11668500	chr19:18836674	CRTC1	42,250	CRTC1	N			N					3	1
rs84473	chr22:38557326	PLA2G6-PICK1	6,758	PLA2G6		B	B					ELF1:L	4	2
rs132971	chr22:38561911	PLA2G6-PICK1	2,173	PLA2G6			L			L	L		4	1
rs132985	chr22:38563471	PLA2G6-PICK1	613	PLA2G6			ANL						4	3
rs738320	chr22:38568715	PLA2G6-PICK1	-4,631	PLA2G6	N		ANLB	AN		NL	L		18	4
rs738322	chr22:38569006	PLA2G6-PICK1	-4,922	PLA2G6	NIL	L	ANLB	N		NL	L	CEBPb:L JUND:L	26	5
rs4379	chr22:38569171	PLA2G6-PICK1	-5,087	PLA2G6	N		ANLB	AN		NL	L		18	4
rs133009	chr22:38569653	PLA2G6-PICK1	-5,569	PLA2G6			ANLB	AN		NL	L		13	4
rs133012	chr22:38570721	PLA2G6-PICK1	-6,637	PLA2G6			AN			A			3	2
rs4381	chr22:38570791	PLA2G6-PICK1	-6,707	PLA2G6			AN			A			3	2
rs4382	chr22:38570842	PLA2G6-PICK1	-6,758	PLA2G6			AN			A			3	2
rs133014	chr22:38571087	PLA2G6-PICK1	6,668	PLA2G6	B		AN			A			4	3
rs75387	chr22:38572057	PLA2G6-PICK1	5,698	PLA2G6		B	ANLB	ANB	B	ANB	B		19	4

rs4384	chr22:38572440	PLA2G6-PICK1	5,315	PLA2G6	NB	B	ANLB	ANB	B	ANB	B	EBF:B USF1:B EBF1C8:B PAX5:B BCL11a:B IRF4:B POL2:B NFKb:B SP1:B TCF12:B NFkb:B P300:B NFKB:B IRF4m17:B ZBTB33:B	50	4
rs133015	chr22:38572526	PLA2G6-PICK1	5,229	PLA2G6	NB	B	ANLB	ANB	B	ANB	B	EBF:B NFkb:B EBF1C8:B IRF4m17:B PAX5:B ZBTB33:B IRF4:B POL2:B NFKb:B	37	4
rs133016	chr22:38572582	PLA2G6-PICK1	5,173	PLA2G6	B	B	ANLB	ANB	B	ANB	B	EBF:B EBF1C8:B IRF4m17:B IRF4:B	27	4
rs133017	chr22:38572637	PLA2G6-PICK1	5,118	PLA2G6	I	B	ANLB	ANB	B	ANB	B	EBF:B EBF1C8:B IRF4m17:B IRF4:B	26	5
rs133018	chr22:38572761	PLA2G6-PICK1	4,994	PLA2G6		B	ANLB	ANB	B	AB	B		18	4
rs133019	chr22:38573035	PLA2G6-PICK1	4,720	PLA2G6			ANLB	ANLB	B	AB	LB		18	4
rs4385	chr22:38573229	PLA2G6-PICK1	4,526	PLA2G6	B	B	ANLB	ANLB	B	AB	LB		23	4
rs133021	chr22:38573338	PLA2G6-PICK1	4,417	PLA2G6		B	ANLB	ANLB	B	AB	LB		18	4
rs133024	chr22:38574066	PLA2G6-PICK1	3,689	PLA2G6	N	L	ANLB	ANLB	B	ANB	LB	USF1:L SIN3ak:L	24	4
rs2277844	chr22:38577515	PLA2G6-PICK1	240	PLA2G6		B	ANLB	ANLB	ANILB	ANILB	LB		36	5
rs5750558	chr22:38582497	PLA2G6-PICK1	-4,660	PLA2G6			L	A			L		3	2
rs2413505	chr22:38592967	PLA2G6-PICK1	-4,921	MAFF			L				L		2	1
rs5756968	chr22:38595240	PLA2G6-PICK1	-2,648	MAFF		B	LB	A	B		B		8	3
rs5750561	chr22:38595260	PLA2G6-PICK1	-2,628	MAFF		B	LB	A	B		B		8	3
rs3761445	chr22:38595411	PLA2G6-PICK1	-2,477	MAFF	B	B	LB	A	B		B	SRF:B POL2:B YY1:B OCT2:B SP1:B POUF1:B POU2f2:B OCT2_:B	30	3
rs4374456	chr22:38597377	PLA2G6-PICK1	-511	MAFF	N	B	NL	ANL	ANLB	ANILB	LB		24	5
rs4608623	chr22:38597378	PLA2G6-PICK1	-510	MAFF	N	B	NL	ANL	ANLB	ANILB	LB		24	5
rs2267372	chr22:38598234	PLA2G6-PICK1	296	MAFF	N	B	B	ANLB	ANLB	ANILB	LB	TAF1:L POL2:B	34	5
rs4821764	chr22:38599364	PLA2G6-PICK1	338	MAFF	NLB	B	ANLB	ANLB	ANILB	ANILB	LB	POL2:BL	49	5
rs4820323	chr22:38599767	PLA2G6-PICK1	741	MAFF			ANLB	ANLB	ANILB	ANLB	LB	HNF4a:L	28	5
rs4820324	chr22:38599857	PLA2G6-PICK1	831	MAFF			ANLB	ANLB	ANILB	ANILB	LB	HNF4a:L	29	5
rs4820325	chr22:38599978	PLA2G6-PICK1	952	MAFF	N		ANLB	ANB	ANLB	ANILB	LB		28	5
rs2267373	chr22:38600542	PLA2G6-PICK1	1,516	MAFF	N	B	ANLB	ANB	ANLB	AB	LB		24	4
rs2267374	chr22:38600595	PLA2G6-PICK1	1,569	MAFF	N	B	ANLB	ANB	ANLB	AB	LB		24	4
rs2267375	chr22:38601231	PLA2G6-PICK1	2,205	MAFF	N	B	ANLB	ANB	ANB	ANB	LB		26	4
rs11914181	chr22:38602140	PLA2G6-PICK1	3,114	MAFF	N		ANLB	AN	AN	A			14	4
rs8139952	chr22:38606989	PLA2G6-PICK1	-2,552	MAFF			AB	A					3	2
rs2235264	chr22:38609950	PLA2G6-PICK1	409	MAFF	NB	B	ANLB	NB	ANB	N	B		21	4
rs9610915	chr22:38611080	PLA2G6-PICK1	1,539	MAFF	N	B	ANLB	NB	ANB		B		18	4

Variants in linkage disequilibrium ($r^2 \geq 0.7$) with body fat percentage-associated index SNPs overlapping two or more regulatory datasets in the same tissue are displayed alphabetically by locus name. Negative distance from nearest GENCODE v12 TSS indicates the variant is 5` of the TSS. Tissues with elements overlapping each SNP are indicated as A=Adipose, B=Blood, I=Pancreatic Islets, L=Liver, N=Brain; Chr, Chromosome; TSS, Transcription Start Site.

Supplementary Table 20. Pathway enrichment among the 43 loci that showed genome-wide significant or putative evidence ($P < 10^{-5}$) of association with body fat percentage in the all-ancestry analyses using DEPICT, showing the top 72 ($P \leq 0.01$) most significant genesets

Re-annotated gene set ID	Re-annotated gene set name	*P* value	False discovery rate < 0.05?	Gene set genes in assoicated BFP loci
ENSG00000100347	SAMM50 protein complex	7.75E-05	No	*, TOMM40, DCN, DTNB
ENSG00000123338	NCKAP1L protein complex	8.59E-05	No	*, FUBP1, RELB, SPRY2
ENSG00000035115	SH3YL1 protein complex	1.99E-04	No	*, RPTOR, APOE, FUBP1
GO:0051224	Negative Regulation Of Protein Transport	4.91E-04	No	*, CREB5, RELB, ENSG00000249231
GO:0045687	Positive Regulation Of Glial Cell Differentiation	4.95E-04	No	*, GPRC5B, ENSG00000245719, LMX1B
GO:0007603	Phototransduction, Visible Light	7.54E-04	No	SLC16A8
GO:0050872	White Fat Cell Differentiation	7.64E-04	No	*, PPARG, TOX3, PLA2G6
GO:0043574	Peroxisomal Transport	8.49E-04	No	*, PLA2G6, CLASRP, CLPTM1
GO:0006625	Protein Targeting To Peroxisome	9.60E-04	No	*, PLA2G6, RPTOR, IQCK
GO:0072663	Establishment Of Protein Localization To Peroxisome	9.60E-04	No	*, PLA2G6, RPTOR, IQCK
GO:0072662	Protein Localization To Peroxisome	9.60E-04	No	*, PLA2G6, RPTOR, IQCK
GO:0018195	Peptidyl-Arginine Modification	0.001	No	*, TOMM40, EYA2, ENSG00000249231
MP:0010872	Increased Trabecular Bone Mass	0.001	No	APOC1
ENSG00000092208	GEMIN2 protein complex	0.001	No	TOMM40, PSMA5
GO:0016558	Protein Import Into Peroxisome Matrix	0.002	No	PLA2G6
ENSG00000131773	KHDRBS3 protein complex	0.002	No	*, BEND5, FUBP1, ZNF664
ENSG00000004799	PDK4 protein complex	0.002	No	*, CLPTM1, TOMM40, TMEM140
MP:0005100	Abnormal Choroid Pigmentation	0.002	No	*, TMEM140, BAIAP2L2, APOE
GO:0050953	Sensory Perception Of Light Stimulus	0.002	No	SLC16A8
GO:0032391	Photoreceptor Connecting Cilium	0.002	No	IQCK, RPGRIP1L
ENSG00000171357	C1orf190 protein complex	0.002	No	*, PICK1, CLPTM1, DTNB
GO:0043046	Dna Methylation Involved In Gamete Generation	0.002	No	*, TMEM18, ENSG00000224165, PLA2G6
MP:0002310	Decreased Susceptibility To Hepatic Steatosis	0.003	No	*, APOC1, PPARG, RELB
GO:0007602	Phototransduction	0.003	No	SLC16A8
ENSG00000034693	PEX3 protein complex	0.003	No	CLPTM1
ENSG00000088247	KHSRP protein complex	0.003	No	FUBP1
GO:0007601	Visual Perception	0.003	No	SLC16A8
MP:0008528	Polycystic Kidney	0.003	No	*, IQCK, SPRY2, GIPC2
GO:0005452	Inorganic Anion Exchanger Activity	0.003	No	SLC16A8
ENSG00000121390	PSPC1 protein complex	0.004	No	*, FUBP1, CLASRP, NEXN
GO:0060347	Heart Trabecula Formation	0.004	No	*, GIPR, AGBL4, CREB5
GO:0015701	Bicarbonate Transport	0.004	No	*, ENSG00000249231, GIPC2, NEGR1
GO:0072372	Primary Cilium	0.004	No	IQCK, RPGRIP1L
GO:0090317	Negative Regulation Of Intracellular Protein Transport	0.004	No	*, CLASRP, ENSG00000249231, CREB5
GO:0031513	Nonmotile Primary Cilium	0.004	No	SLC16A8
GO:0072164	Mesonephric Tubule Development	0.004	No	*, DTNB, CRTC1, DCN
GO:0072163	Mesonephric Epithelium Development	0.004	No	*, DTNB, CRTC1, DCN
ENSG00000137324	ENSG00000137324 protein complex	0.005	No	*, C7orf49, CRTC1, HSD17B12
ENSG00000206376	EHMT2 protein complex	0.005	No	*, C7orf49, CRTC1, HSD17B12
ENSG00000204371	EHMT2 protein complex	0.005	No	*, C7orf49, CRTC1, HSD17B12
MP:0001566	Hyperphosphatemia	0.005	No	*, TOX3, LMX1B, GIPC2
GO:0042462	Eye Photoreceptor Cell Development	0.006	No	*, AGBL3, IQCH, ENSG00000245719
GO:0042551	Neuron Maturation	0.006	No	*, WDR91, LMX1B, PICK1
ENSG00000166025	AMOTL1 protein complex	0.006	No	*, FUBP1, RPGRIP1L, ZNF746
GO:0009629	Response To Gravity	0.006	No	*, IQCH, ENSG00000233715, DNAH10
GO:0042461	Photoreceptor Cell Development	0.006	No	SLC16A8
GO:0009584	Detection Of Visible Light	0.006	No	*, SLC16A8, MC4R, DNAH10
MP:0008585	Absent Photoreceptor Outer Segment	0.006	No	SLC16A8
MP:0000153	Rib Bifurcation	0.006	No	EYA2
ENSG00000100284	TOM1 protein complex	0.006	No	*, RPTOR, C7orf49, ZNF746
ENSG00000181656	GPR88 protein complex	0.007	No	CLASRP, FUBP1
GO:0035112	Genitalia Morphogenesis	0.007	No	*, PPARG, TNP1, ENSG00000249231
ENSG00000159086	GCFC1 protein complex	0.007	No	CLASRP, CLPTM1
ENSG00000065268	WDR18 protein complex	0.007	No	*, TOMM40, FUBP1, IGF2BP1
ENSG00000173465	SSSCA1 protein complex	0.007	No	*, TOMM40, CLPTM1, TNP1
MP:0000786	Abnormal Embryonic Neuroepithelial Layer Differentiation	0.008	No	*, IGF2BP1, EYA2, PSMA5
GO:0019433	Triglyceride Catabolic Process	0.008	No	PPARG
GO:0009416	Response To Light Stimulus	0.008	No	*, SLC16A8, MC4R, EYA2

GO:0015301	Anion:Anion Antiporter Activity	0.008	No	SLC16A8
GO:0046464	Acylglycerol Catabolic Process	0.008	No	PPARG
GO:0046461	Neutral Lipid Catabolic Process	0.008	No	PPARG
GO:0044269	Glycerol Ether Catabolic Process	0.008	No	PPARG
ENSG00000164751	PEX2 protein complex	0.008	No	*, APOC1, PLA2G6, HSD17B12
GO:0032924	Activin Receptor Signaling Pathway	0.009	No	*, ASXL2, CCDC92, ENSG00000245719
ENSG00000162928	PEX13 protein complex	0.009	No	*, RPTOR, CLPTM1, PSMA5
GO:0009583	Detection Of Light Stimulus	0.009	No	SLC16A8
GO:0072202	Cell Differentiation Involved In Metanephros Development	0.01	No	*, ENSG00000245719, DCN, BAIAP2L2
ENSG00000089351	GRAMD1A protein complex	0.01	No	*, PLA2G6, ENSG00000228536, SPRY2
MP:0000522	Kidney Cortex Cysts	0.01	No	*, DCN, SPRY2, IQCK
GO:0030890	Positive Regulation Of B Cell Proliferation	0.01	No	*, HIVEP3, COBLL1, CLASRP
GO:0007031	Peroxisome Organization	0.01	No	*, PLA2G6, RPTOR, C7orf49
MP:0000372	Irregular Coat Pigmentation	0.01	No	*, SLC16A8, DCN, LMX1B
ENSG00000121691	CAT protein complex	0.01	No	*, RPTOR, HSD17B12, ZNF746

Supplementary Table 21. Tissue enrichment among the 43 loci that showed genome-wide significant or putative evidence ($P < 10^{-5}$) of association with body fat percentage in the all-ancestry analyses using DEPICT, showing the top 54 ($P < 0.10$) most significant results.

Tissue annotation MeSH Term	Tissue annoation	*P* value	False discovery rate < 0.05?
A06.688.357	Hypothalamo-Hypophyseal System	0.01	No
A08.713.357.750	Pituitary Gland	0.01	No
A06.407.747	Pituitary Gland	0.01	No
A08.186.211.730.317.357.352	Hypothalamus, Middle	0.01	No
A08.186.211.730.317.357.352.435	Hypothalamo-Hypophyseal System	0.01	No
A08.713.357	Hypothalamo-Hypophyseal System	0.01	No
A06.688.357.750	Pituitary Gland	0.01	No
A08.713	Neurosecretory Systems	0.01	No
A06.688	Neurosecretory Systems	0.01	No
A08.186.211.730.317.357.352.435.500	Pituitary Gland	0.01	No
A11.251.860.180.432	Hep G2 Cells	0.02	No
A11.436.348.500	Hep G2 Cells	0.02	No
A10.690.467.491	Muscle, Smooth, Vascular	0.02	No
A02.633.570.491	Muscle, Smooth, Vascular	0.02	No
A07.231.491	Muscle, Smooth, Vascular	0.02	No
A08.186.211.730.317.357	Hypothalamus	0.03	No
A11.251.210.190.495	Jurkat Cells	0.03	No
A15.382.490.555.567.569.440	Jurkat Cells	0.03	No
A11.251.860.180.495	Jurkat Cells	0.03	No
A11.436.348	Hepatocytes	0.04	No
A11.620.520	Myocytes, Smooth Muscle	0.05	No
A11.620	Muscle Cells	0.05	No
A11.436	Epithelial Cells	0.05	No
A11.251.860.180	Cell Line, Tumor	0.05	No
A11.251.210.190	Cell Line, Tumor	0.05	No
A11.251.860	Tumor Cells, Cultured	0.05	No
A11.872.190.260	Embryoid Bodies	0.06	No
A11.627.624.249	Monocyte-Macrophage Precursor Cells	0.06	No
A11.627.624.249.500	U937 Cells	0.06	No
A11.627.482.665	Monocyte-Macrophage Precursor Cells	0.06	No
A11.627.635.675.750	Monocyte-Macrophage Precursor Cells	0.06	No
A11.627.635.675.750.500	U937 Cells	0.06	No
A11.251.860.180.880	U937 Cells	0.06	No
A11.251.210.190.880	U937 Cells	0.06	No
A11.627.482.665.500	U937 Cells	0.06	No
A02.633	Muscles	0.07	No

A10.690	Muscles	0.07	No
A15.145.846	Serum	0.09	No
A12.207.152.846	Serum	0.09	No
A15.382.490.555	Leukocytes, Mononuclear	0.09	No
A11.118.637.555	Leukocytes, Mononuclear	0.09	No
A15.145.229.637.555	Leukocytes, Mononuclear	0.09	No
A15.382.490.555.652	Monocytes	0.09	No
A15.382.812.547	Monocytes	0.09	No
A15.382.680.547	Monocytes	0.09	No
A11.118.637.555.652	Monocytes	0.09	No
A15.145.229.637.555.652	Monocytes	0.09	No
A15.378.316.580	Monocytes	0.09	No
A11.733.547	Monocytes	0.09	No
A11.148.580	Monocytes	0.09	No
A11.627.624	Monocytes	0.09	No
A08.186.211.730.317	Diencephalon	0.09	No
A11.118.188	Blood Platelets	0.10	No
A15.145.229.188	Blood Platelets	0.10	No

Supplementary Table 22. Ingenuity IPA canonical pathway analysis

Ingenuity Canonical Pathways	*P* value[a]	False-Discovery Rate	Ratio[b]	Input gene list in each pathway
Role of JAK2 in Hormone-like Cytokine Signaling	0.005	0.32	0.054	IRS1,SH2B1
N-acetylglucosamine Degradation I	0.012	0.32	0.111	GNPDA2
N-acetylglucosamine Degradation II	0.015	0.32	0.077	GNPDA2
Estrogen-Dependent Breast Cancer Signaling	0.016	0.32	0.027	HSD17B12,CREB5
Neurotrophin/TRK Signaling	0.019	0.32	0.026	SPRY2,CREB5
ERK/MAPK Signaling	0.02	0.32	0.014	PPARG,PLA2G6,CREB5
cAMP-mediated signaling	0.03	0.36	0.013	HTR1A,CREB5,MC4R
UDP-N-acetyl-D-galactosamine Biosynthesis II	0.03	0.36	0.040	GNPDA2
Protein Ubiquitination Pathway	0.04	0.37	0.011	DNAJB4,DNAJC27,PSMA5
G-Protein Coupled Receptor Signaling	0.04	0.37	0.011	HTR1A,CREB5,MC4R
Gαs Signaling	0.05	0.37	0.016	CREB5,MC4R
Type II Diabetes Mellitus Signaling	0.05	0.37	0.012	PPARG,IRS1
p38 MAPK Signaling	0.05	0.37	0.017	PLA2G6,CREB5
Insulin Receptor Signaling	0.06	0.39	0.013	IRS1,RPTOR
AMPK Signaling	0.06	0.39	0.011	IRS1,RPTOR
Polyamine Regulation in Colon Cancer	0.07	0.39	0.033	PPARG
Aldosterone Signaling in Epithelial Cells	0.08	0.39	0.012	DNAJB4,DNAJC27
MIF-mediated Glucocorticoid Regulation	0.10	0.39	0.024	PLA2G6
Serotonin Receptor Signaling	0.10	0.39	0.020	HTR1A
Circadian Rhythm Signaling	0.10	0.39	0.026	CREB5
IL-9 Signaling	0.10	0.39	0.025	IRS1
NRF2-mediated Oxidative Stress Response	0.11	0.39	0.010	DNAJB4,MAFF
Estrogen Biosynthesis	0.11	0.39	0.020	HSD17B12
ILK Signaling	0.11	0.39	0.010	IRS1,CREB5
Thyroid Cancer Signaling	0.12	0.39	0.023	PPARG
mTOR Signaling	0.12	0.39	0.009	IRS1,RPTOR
MIF Regulation of Innate Immunity	0.12	0.39	0.019	PLA2G6
Dermatan Sulfate Biosynthesis (Late Stages)	0.12	0.39	0.021	HS6ST3
Role of Oct4 in Mammalian Embryonic Stem Cell Pluripotency	0.13	0.39	0.019	IGF2BP1
Role of IL-17F in Allergic Inflammatory Airway Diseases	0.13	0.39	0.021	CREB5
Chondroitin Sulfate Biosynthesis (Late Stages)	0.13	0.39	0.018	HS6ST3
Heparan Sulfate Biosynthesis (Late Stages)	0.14	0.39	0.016	HS6ST3
Chondroitin Sulfate Biosynthesis	0.16	0.39	0.014	HS6ST3
Dermatan Sulfate Biosynthesis	0.16	0.39	0.014	HS6ST3
Phospholipase C Signaling	0.16	0.39	0.008	PLA2G6,CREB5
Heparan Sulfate Biosynthesis	0.16	0.39	0.013	HS6ST3
Phospholipases	0.16	0.39	0.015	PLA2G6
ATM Signaling	0.17	0.39	0.015	CREB5
Role of JAK1 and JAK3 in γc Cytokine Signaling	0.17	0.39	0.015	IRS1
Eicosanoid Signaling	0.18	0.39	0.012	PLA2G6
ERK5 Signaling	0.18	0.39	0.015	CREB5
Angiopoietin Signaling	0.18	0.39	0.013	GRB14
Hypoxia Signaling in the Cardiovascular System	0.18	0.39	0.015	CREB5
Role of MAPK Signaling in the Pathogenesis of Influenza	0.19	0.39	0.014	PLA2G6
GDNF Family Ligand-Receptor Interactions	0.19	0.39	0.013	IRS1
Growth Hormone Signaling	0.19	0.39	0.013	IRS1
PEDF Signaling	0.20	0.39	0.013	PPARG
Prolactin Signaling	0.20	0.39	0.012	IRS1
IL-4 Signaling	0.20	0.39	0.013	IRS1
FLT3 Signaling in Hematopoietic Progenitor Cells	0.21	0.39	0.013	CREB5
VEGF Family Ligand-Receptor Interactions	0.21	0.39	0.011	PLA2G6
Prostate Cancer Signaling	0.23	0.40	0.010	CREB5
FXR/RXR Activation	0.23	0.40	0.009	PPARG
Melanocyte Development and Pigmentation Signaling	0.23	0.40	0.011	CREB5

FGF Signaling	0.23	0.40	0.011	CREB5
Neuregulin Signaling	0.24	0.40	0.010	DCN
Crosstalk between Dendritic Cells and Natural Killer Cells	0.24	0.40	0.009	PVRL2
Fcγ Receptor-mediated Phagocytosis in Macrophages and Monocytes	0.25	0.40	0.009	PLA2G6
PPAR Signaling	0.25	0.40	0.009	PPARG
SAPK/JNK Signaling	0.25	0.40	0.010	IRS1
Antioxidant Action of Vitamin C	0.26	0.40	0.009	PLA2G6
IGF-1 Signaling	0.26	0.40	0.009	IRS1
NGF Signaling	0.28	0.41	0.008	CREB5
Fc Epsilon RI Signaling	0.29	0.41	0.009	PLA2G6
Corticotropin Releasing Hormone Signaling	0.30	0.41	0.007	CREB5
CCR3 Signaling in Eosinophils	0.30	0.41	0.007	PLA2G6
Sperm Motility	0.31	0.41	0.007	PLA2G6
Synaptic Long Term Potentiation	0.31	0.41	0.008	CREB5
p70S6K Signaling	0.31	0.41	0.008	IRS1
Atherosclerosis Signaling	0.31	0.41	0.007	PLA2G6
P2Y Purigenic Receptor Signaling Pathway	0.31	0.41	0.007	CREB5
Gαi Signaling	0.31	0.41	0.007	HTR1A
PI3K Signaling in B Lymphocytes	0.33	0.42	0.007	IRS1
Protein Kinase A Signaling	0.33	0.42	0.005	CREB5,EYA2
GNRH Signaling	0.33	0.42	0.007	CREB5
IL-12 Signaling and Production in Macrophages	0.34	0.42	0.006	PPARG
Synaptic Long Term Depression	0.35	0.44	0.006	PLA2G6
Epithelial Adherens Junction Signaling	0.36	0.44	0.006	PVRL2
Regulation of eIF4 and p70S6K Signaling	0.37	0.44	0.006	IRS1
Tight Junction Signaling	0.38	0.45	0.006	PVRL2
Germ Cell-Sertoli Cell Junction Signaling	0.38	0.45	0.006	PVRL2
B Cell Receptor Signaling	0.39	0.45	0.006	CREB5
Dopamine-DARPP32 Feedback in cAMP Signaling	0.40	0.45	0.005	CREB5
Endothelin-1 Signaling	0.41	0.45	0.005	PLA2G6
CREB Signaling in Neurons	0.42	0.45	0.005	CREB5
Ephrin Receptor Signaling	0.42	0.45	0.005	CREB5
PPARα/RXRα Activation	0.42	0.45	0.005	IRS1
Sertoli Cell-Sertoli Cell Junction Signaling	0.42	0.45	0.005	PVRL2
Dendritic Cell Maturation	0.42	0.45	0.005	CREB5
Calcium Signaling	0.43	0.45	0.005	CREB5
LPS/IL-1 Mediated Inhibition of RXR Function	0.50	0.51	0.004	HS6ST3
Huntington's Disease Signaling	0.50	0.51	0.004	CREB5
Cardiac Hypertrophy Signaling	0.50	0.51	0.004	IRS1
Xenobiotic Metabolism Signaling	0.57	0.57	0.003	HS6ST3
Role of Macrophages, Fibroblasts and Endothelial Cells in Rheumatoid Arthritis	0.61	0.61	0.003	CREB5

[a]P values were calculated based on Fisher’s right tailed exact test.

[b]Ratio of the number of genes from the input dataset in the selected pathway relative to the total number of genes in that pathway

Supplementay Table 23. Look up of genes located in each of the 12 fat percentage associated loci in a genome-wide transgenic RNAi screen in Drosophila

SNP	Chr	Position	nearby genes	Fly Ortholog Annotation Symbol	Fly Ortholog Gene Symbol	%change in whole body triglycerides[a]	Validated in second screen?
rs543874	1	176,156,103	SEC16B	CG32654	Sec16	not screened	
			RASAL2	CG42684	CG42684	not screened	
rs6755502	2	625,721	TMEM18	CG30051	CG30051	+6%, -23%	
			SH3YL1	No known orthologue			
			ACP1	CG14297	CG14297	not screened	
			FAM150B	No known orthologue			
			SNTG2	CG4905	Syn2	not screened	
rs6738627	2	165,252,696	GRB14	CG11940	jog	-27%	
			SNORA70F	No known orthologue			
			COBLL1	No known orthologue			
			SLC38A11	CG13743	CG13743	not screened	
			SCN3A	No known orthologue			
rs2943652	2	226,816,690	IRS1	CG5686	chico	-4%	
rs693839	13	79,856,289	SPRY2	CG1921	sty	-90%	yes
rs4788099	16	28,763,228	SH2B1	CG17367	LnK	not screened	
			EIF3CL/EIF3C	CG4954	eIF3-S8	+14%, -100%	
			CLN3	CG5582	cln3	-14%	
			SULT1A2	No known orthologue			
			APOBR	No known orthologue			
			SULT1A1	No known orthologue			
			IL27	No known orthologue			
			NUPR1	No known orthologue			
			CCDC101	CG30390	Sgf29	-17%	
			ATXN2L	CG5166	Atx2	-57%	
			TUFM	CG6050	EfTuM	-30%	
			ATP2A1	CG3725	Ca-P60A	not screened	
			RABEP2	No known orthologue			
			SPNS1	CG8428	spin	+35%	
			LAT	No known orthologue			
			RRN3P2	No known orthologue			
			RUNDC2C (SNX29P2)	No known orthologue			
rs1558902	16	52,361,075	FTO	No known orthologue			
			RBL2	CG7413	Rbf	not screened	
			AKTIP	CG16894	CG16894	-35%	
			IRX3	No known orthologue			
			IRX6	CG10571	ara	-8%	
			IRX6	CG10605	caup	5%	
			IRX5	CG10601	mirr	35%	
			RPGRIP1L	No known orthologue			
rs9906944	17	44,446,419	IGF2BP1	CG1691	Imp	-18%	
			PRAC	No known orthologue			

			HOXB13	CG11648	Abd-B	-32%	
			MIR3185	No known orthologue			
			CALCOCO2	No known orthologue			
			ATP5G1	CG1746	CG1746	not screened	
			UBE2Z	No known orthologue			
			SNF8	CG6637	lsn	-63%	
			GIP	No known orthologue			
			B4GALNT2	No known orthologue			
			GNGT2	No known orthologue			
			ABI3	CG9749	Abi	+50%	
			PHOSPHO1	CG14212	CG14212	-57%	
			ZNF652	No known orthologue			
			PHB	CG10691	l(2)37Cc	-62%	
			NGFR	No known orthologue			
rs6567160	18	55,980,115	MC4R	CG42244	Octβ3R	not screened	
rs757318	19	18,681,308	CRTC1	CG6064	TORC	-52%	yes
rs6857	19	50,084,094	APOE	No known orthologue			
			TOMM40	CG12157	Tom40	-22%	
			TOMM40	CG8330	tomboy40	-33%	
			TOMM40	CG4520	CG4520	+15%	
			IGSF23	No known orthologue			
			PVR	No known orthologue			
			MIR4531	No known orthologue			
			CEACAM19	No known orthologue			
			BCL3	No known orthologue			
			CBLC	CG7037	Cbl	+20%	
			BCAM	No known orthologue			
			PVRL2	No known orthologue			
			APOC1	No known orthologue			
			APOE	No known orthologue			
			CLPTM1	CG3702	CG3702	+80%, +22%	
			RELB	CG6667	dl	-8%	
			CLASRP	CG6695	CG6695	-49%	
			ZNF296	CG9650	CG9650	+9%	
			GEMIN7	No known orthologue			
			LRRC68 (PPP1R37)	CG31635	CG31635	-43%	
			BLOC1S3	No known orthologue			
			EXOC3L2	CG5341	sec6	-38%	
			MARK4	CG8201	par-1	not screened	
rs3761445	22	36,925,357	PLA2G6	CG6718	iPLA2-VIA	not screened	
			PICK1	CG6167	PICK1	+6%	
			SOX10	CG15552	Sox100B	-62%	

[a]Not screened or when a fly line is too weak to reach the screening stage.
Value represents percentage difference in whole body triglyceride content between transgenic RNAi lines and wild type flies.
When two values are listed, it means that two independent transgenic RNAi lines were availbale.

Supplementary table 24. The gene list of RNAi knockdown in adult drosophila

SNP	Chr.	Positions (hg18)	Candidate gene in locus[a,b]	Fly orthologue gene symbol	Fly orthologue annotation symbol	Transform ID	Control		RNA knockdown		Difference (% of control)		
							Mean	SE	Mean	SE	Mean	SE	*P*-value
rs543874	1	176,156,103	*SEC16B*	Sec16	CG32654	29635	-	-	N/A	N/A	N/A	N/A	N/A
			SEC16B	Sec16	CG32654	109645	-	-	N/A	N/A	N/A	N/A	N/A
rs6755502	2	625,721	*TMEM18*	CG30051	CG30051	43067	546	84	402	3	-26%	15%	0.23
			TMEM18	CG30051	CG30051	106840	546	84	447	108	-18%	25%	0.51
rs6738627	2	165,252,696	*GRB14*	pico	CG11940	16369	546	84	375	34	-31%	17%	0.13
			GRB14	pico	CG11940	16371	546	84	492	46	-10%	18%	0.61
rs2943652	2	226,816,690	*IRS1*	chico	CG5686	101329	546	84	380	54	-30%	18%	0.18
			IRS1	chico	CG5686	7777	546	84	385	44	-29%	17%	0.17
rs693839	13	79,856,289	*SPRY2*	sty	CG1921	6948	404	25	73	4	-82%	6%	0.0002
rs4788099	16	28,763,228	*SH2B1*	Lnk	CG17367	103646	546	84	475	43	-13%	17%	0.50
			SH2B1	Lnk	CG17367	32892	546	84	494	21	-9%	16%	0.59
rs9906944	17	44,446,419	*IGF2BP1*	Imp	CG1691	20322	546	84	429	53	-21%	18%	0.31
			IGF2BP1	Imp	CG1691	20321	546	84	390	25	-28%	16%	0.15
rs6567160	18	55,980,115	*MC4R*	Octβ3R	CG42244	9068	546	84	479	58	-12%	19%	0.55
			MC4R	Octβ3R	CG42244	106519	546	84	464	43	-15%	17%	0.44
rs757318	19	18,681,308	*CRTC1*	TORC	CG6064	100974	546	84	402	63	-26%	19%	0.25
rs6857	19	50,084,094	*TOMM40*	Tom40	CG12157	13178	546	84	538	95	-1%	23%	0.96
			TOMM40	Tom40	CG12157	13177	546	84	540	106	-1%	25%	0.97
			TOMM40	tomboy40	CG8330	105557	546	84	511	101	-6%	24%	0.81
			TOMM40	tomboy40	CG8330	42439	546	84	451	34	-17%	17%	0.36
			TOMM40	CG4520	CG4520	34862	546	84	345	17	-37%	16%	0.08
			TOMM40	CG4520	CG4520	34863	546	84	562	50	3%	18%	0.87
			ZNF229	ATbp	CG17172	110021	546	84	325	29	-40%	16%	0.07
			ZNF229	su(Hw)	CG8573	100395	546	84	275	84	-50%	22%	0.09
			ZNF229	su(Hw)	CG8573	10724	546	84	384	21	-30%	16%	0.14
			ZNF229	crol	CG14938	104313	546	84	650	96	19%	23%	0.46
rs3761445	22	36,925,357	*PLA2G6*	iPLA2-VIA	CG6718	108294	546	84	373	22	-32%	16%	0.12
			PICK1	PICK1	CG6167	22268	546	84	244	36	-55%	17%	0.03
			PICK1	PICK1	CG6167	22269	546	84	639	94	17%	23%	0.50
			SLC16A8	CG8468	CG8468	6452	546	84	421	49	-23%	18%	0.27
			SLC16A8	CG8468	CG8468	6453	546	84	230	31	-58%	16%	0.02
			SLC16A8	Sln	CG8271	109464	546	84	409	18	-25%	16%	0.19
			SLC16A8	Sln	CG8271	4607	546	84	342	19	-37%	16%	0.08
			SLC16A8	CG11665	CG11665	7314	546	84	369	23	-32%	16%	0.11

N/A: the fly RNAi stocks were not available.

[a]Candidate gene identified based on biological plausible roles in adipocyte metabolism or the nearest gene to the lead SNP.

[b]All flies were aged for 2 weeks except the experients for the sty gene. The triglyceride levels were too low for the *SPRY2*#6948 and aged for 1 week.

Supplementary Table 25. Association of established susceptibility loci for BMI and extreme obesity with body fat percentage in the joint GWAS and Metabochip meta-analysis

SNP	Chr	Position (bp, hg18)	Nearest gene	Effect allele[a]	Other allele	EAF (%)	Per-allele change in BF%[b]	*P*-value	N
**BMI associated loci**									
rs977747	1	47,457,264	*TAL1*	T	G	40%	0.005	0.32	100,394
rs657452	1	49,362,434	*AGBL4*	A	G	41%	0.016	7.96E-04	98,205
rs11583200	1	50,332,407	*ELAVL4*	C	T	41%	0.017	2.67E-04	99,738
rs3101336	1	72,523,773	*NEGR1*	C	T	62%	0.024	5.21E-07	100,638
rs12566985	1	74,774,781	*FPGT-TNNI3K*	G	A	47%	0.012	0.011	96,664
rs12401738	1	78,219,349	*FUBP1*	A	G	34%	0.023	3.28E-06	90,764
rs11165643	1	96,696,685	*PTBP2*	T	C	57%	0.019	3.65E-05	100,658
rs17024393	1	109,956,211	*GNAT2*	C	T	4%	0.048	3.88E-04	84,184
rs543874	1	176,156,103	*SEC16B*	G	A	19%	0.032	4.53E-08	100,705
rs2820292	1	200,050,910	*NAV1*	C	A	54%	0.015	9.62E-04	100,208
rs13021737	2	622,348	*TMEM18*	G	A	83%	0.038	6.38E-09	85,050
rs10182181	2	25,003,800	*ADCY3*	G	A	46%	0.020	1.61E-05	99,189
rs11126666	2	26,782,315	*KCNK3*	A	G	29%	0.006	0.27	100,090
rs1016287	2	59,159,129	*FLJ30838*	T	C	28%	0.017	0.001	100,654
rs11688816	2	62,906,552	*EHBP1*	G	A	53%	0.012	0.008	98,418
rs2121279	2	142,759,755	*LRP1B*	T	C	15%	0.008	0.25	90,659
rs1460676	2	164,275,935	*FIGN*	C	T	18%	0.010	0.089	100,696
rs1528435	2	181,259,207	*UBE2E3*	T	C	63%	0.011	0.017	100,639
rs17203016	2	207,963,763	*CREB1*	G	A	19%	0.017	0.004	96,121
rs7599312	2	213,121,476	*ERBB4*	G	A	73%	0.011	0.030	100,136
rs492400	2	219,057,996	*USP37*	C	T	42%	0.002	0.71	100,494
rs2176040	2	226,801,046	*LOC646736*	A	G	35%	0.033	1.07E-11	99,348
rs6804842	3	25,081,441	*RARB*	G	A	56%	0.009	0.049	100,664
rs2365389	3	61,211,502	*FHIT*	C	T	57%	0.018	2.43E-04	85,423
rs3849570	3	81,874,802	*GBE1*	A	C	35%	0.009	0.073	92,477
rs13078960	3	85,890,280	*CADM2*	G	T	20%	0.014	0.022	89,086
rs16851483	3	142,758,126	*RASA2*	T	G	9%	0.041	7.75E-05	73,922
rs1516725	3	187,306,698	*ETV5*	C	T	87%	0.019	0.007	99,356
rs10938397	4	44,877,284	*GNPDA2*	G	A	43%	0.025	1.36E-07	100,137
rs17001654	4	77,348,592	*SCARB2*	G	C	15%	0.001	0.91	75,761
rs13107325	4	103,407,732	*SLC39A8*	T	C	7%	0.028	0.0069	86,539
rs11727676	4	145,878,514	*HHIP*	T	C	91%	0.018	0.070	78,583
rs2112347	5	75,050,998	*POC5*	T	G	60%	0.015	0.0015	100,660
rs7715256	5	153,518,086	*GALNT10*	G	T	43%	0.006	0.22	100,628
rs205262	6	34,671,142	*C6orf106*	G	A	27%	0.015	0.0029	100,165
rs2033529	6	40,456,631	*TDRG1*	G	A	29%	0.005	0.35	98,904
rs2207139	6	50,953,449	*TFAP2B*	G	A	18%	0.018	0.00	99,380
rs9400239	6	109,084,356	*FOXO3*	C	T	67%	0.009	0.052	100,632
rs9374842	6	120,227,364	*LOC285762*	T	C	75%	0.007	0.17	100,609
rs13201877	6	137,717,234	*IFNGR1*	G	A	14%	0.005	0.46	100,555
rs13191362	6	162,953,340	*PARK2*	A	G	88%	0.020	0.0092	90,299
rs1167827	7	75,001,105	*HIP1*	G	A	55%	0.017	0.00	85,582
rs2245368	7	76,446,079	*PMS2L11*	C	T	22%	0.018	0.014	78,858
rs9641123	7	93,035,668	*CALCR*	C	G	42%	0.014	0.013	74,340
rs6465468	7	95,007,450	*ASB4*	T	G	30%	0.007	0.18	89,083
rs17405819	8	76,969,139	*HNF4G*	T	C	69%	0.016	0.0013	100,510
rs16907751	8	81,538,012	*ZBTB10*	C	T	92%	0.032	0.0011	95,712
rs2033732	8	85,242,264	*RALYL*	C	T	75%	0.013	0.017	98,859
rs4740619	9	15,624,326	*C9orf93*	T	C	53%	0.016	3.61E-04	100,650
rs10968576	9	28,404,339	*LINGO2*	G	A	31%	0.021	2.27E-05	100,690
rs6477694	9	110,972,163	*EPB41L4B*	C	T	38%	0.001	0.80	100,626
rs1928295	9	119,418,304	*TLR4*	T	C	55%	0.010	0.022	100,676
rs10733682	9	128,500,735	*LMX1B*	A	G	49%	0.009	0.059	98,469
rs7899106	10	87,400,884	*GRID1*	G	A	6%	0.015	0.17	90,804
rs17094222	10	102,385,430	*HIF1AN*	C	T	21%	0.018[b]	0.0015	97,104

rs11191560	10	104,859,028	*NT5C2*	C	T	11%	0.013	0.10	100,554
rs7903146	10	114,748,339	*TCF7L2*	C	T	72%	0.017	0.0013	100,633
rs4256980	11	8,630,515	*TRIM66*	G	C	63%	0.013	0.006	99,357
rs11030104	11	27,641,093	*BDNF*	A	G	78%	0.021	2.19E-04	97,903
rs2176598	11	43,820,854	*HSD17B12*	T	C	25%	0.020	1.84E-04	100,691
rs3817334	11	47,607,569	*MTCH2*	T	C	40%	0.020	2.91E-05	100,566
rs12286929	11	114,527,614	*CADM1*	G	A	51%	0.009	0.061	99,529
rs7138803	12	48,533,735	*BCDIN3D*	A	G	38%	0.019	5.27E-05	100,667
rs11057405	12	121,347,850	*CLIP1*	G	A	90%	0.030	8.72E-04	79,881
rs12016871	13	26,915,782	*MTIF3*	T	C	20%	0.006	0.42	76,081
rs12429545	13	53,000,207	*OLFM4*	A	G	14%	0.026	2.42E-04	94,240
rs9540493	13	65,103,705	*MIR548X2*	A	G	47%	0.012	0.017	98,739
rs1441264	13	78,478,920	*MIR548A2*	A	G	62%	0.012	0.017	95,911
rs10132280	14	24,998,019	*STXBP6*	C	A	68%	0.002	0.63	98,739
rs12885454	14	28,806,589	*PRKD1*	C	A	65%	0.014	0.0042	100,393
rs11847697	14	29,584,863	*PRKD1*	T	C	7%	0.029	0.021	85,962
rs7141420	14	78,969,207	*NRXN3*	T	C	53%	0.016	3.49E-04	100,649
rs3736485	15	49,535,902	*DMXL2*	A	G	47%	0.011	0.018	100,674
rs16951275	15	65,864,222	*MAP2K5*	T	C	75%	0.014	0.011	99,391
rs7164727	15	70,881,044	*LOC100287559*	T	C	67%	0.016	0.0013	100,691
rs758747	16	3,567,359	*NLRC3*	T	C	28%	0.013	0.019	92,550
rs12446632	16	19,842,890	*GPRC5B*	G	A	86%	0.026	1.70E-04	89,516
rs2650492	16	28,240,912	*SBK1*	A	G	30%	0.018	8.58E-04	93,316
rs3888190	16	28,796,987	*ATP2A1*	A	C	38%	0.027	1.60E-08	99,622
rs4787491	16	29,922,838	*INO80E*	G	A	50%	0.008	0.10	81,508
rs9925964	16	31,037,396	*KAT8*	A	G	62%	0.017	0.0013	80,083
rs2080454	16	47,620,091	*CBLN1*	C	A	40%	0.015	0.0011	100,686
rs1558902	16	52,361,075	*FTO*	A	T	40%	0.051	3.79E-27	99,328
rs9914578	17	1,951,886	*SMG6*	G	C	23%	0.004	0.50	100,365
rs1000940	17	5,223,976	*RABEP1*	G	A	33%	0.013	0.009	98,892
rs12940622	17	76,230,166	*RPTOR*	G	A	58%	0.014	0.0018	100,629
rs1808579	18	19,358,886	*C18orf8*	C	T	53%	0.017	3.45E-04	98,887
rs7239883	18	38,401,669	*LOC284260*	G	A	39%	0.014	0.0035	100,233
rs7243357	18	55,034,299	*GRP*	T	G	81%	0.011	0.059	100,633
rs6567160	18	55,980,115	*MC4R*	C	T	25%	0.034	1.33E-10	100,642
rs17724992	19	18,315,825	*PGPEP1*	A	G	73%	0.011	0.051	85,540
rs29941	19	39,001,372	*KCTD15*	G	A	66%	0.009	0.061	97,970
rs2075650	19	50,087,459	*TOMM40*	A	G	85%	0.037	1.44E-07	88,215
rs2287019	19	50,894,012	*QPCTL*	C	T	81%	0.027	2.77E-05	84,821
rs3810291	19	52,260,843	*ZC3H4*	A	G	63%	0.012	0.023	97,658
rs6091540	20	50,521,269	*ZFP64*	C	T	73%	0.013	0.014	100,128
rs2836754	21	39,213,610	*ETS2*	C	T	58%	0.011	0.022	100,374
**Extreme obesity associated loci**									
rs11208659	1	65,751,868	*LEPR*	C	T	12%	0.006	0.52	74,333
rs17381664	1	77,820,919	*ZZZ3*	C	T	38%	0.018	1.89E-04	96,057
rs17150703	8	9,783,208	*MSRA*	A	G	11%	0.007	0.42	73,481
rs10508503	10	16,339,957	*PTER*	C	T	91%	0.017	0.14	61,505
rs564343	11	65,651,742	*PACS1*	A	G	43%	0.020	0.20	9,208
rs11109072	12	96,425,401	*RMST*	A	C	6%	-0.003	0.84	54,740
rs7989336	13	95,815,549	*HS6ST3*	A	G	49%	0.020	1.74E-04	76,115
rs1957894	14	60,977,864	*PRKCH*	T	G	12%	0.006	0.50	72,140
rs2531995	16	3,953,468	*ADCY9*	T	C	58%	0.017	0.0042	73,767
rs1424233	16	78,240,252	*MAF*	A	G	50%	0.003	0.64	75,812
rs1805081	18	19,394,430	*NPC1*	A	G	59%	0.019	5.23E-05	97,110
rs13041126	20	50,526,403	*MRPS33P4*	T	C	72%	0.013	0.023	76,087

Chr, chromosome; EAF, Effect allele frequency.

[a] The effect allele is the allele associated with increased BMI and extreme obesity risk.

[b] Effects sizes are expressed in SD, based on inverse normally transformed outcomes (mean 0, SD 1).

Table 26 Distribution of the imputation quality metrics (proper_info, rsq) for the 12 BF%-associated loci

SNP	chr	Pos	Nearby gene	Percentile				
				5%	25%	50%	75%	95%
rs1558902	16	52,361,075	*FTO*	0.88	0.99	1.00	1.00	1.00
rs2943652	2	226,816,690	*IRS1*	0.94	0.99	1.00	1.00	1.00
rs6567160	18	55,980,115	*MC4R*	0.92	0.98	1.00	1.00	1.00
rs6755502	2	625,721	*TMEM18*	0.94	1.00	1.00	1.00	1.00
rs6738627	2	165,252,696	*COBLL1*	0.94	0.98	0.99	1.00	1.00
rs693839	13	79,856,289	*SPRY2*	0.94	1.00	1.00	1.00	1.00
rs6857	19	50,084,094	*TOMM40*	0.52	0.87	0.95	0.98	1.00
rs4788099	16	28,763,228	*TUFM*	0.97	1.00	1.00	1.00	1.00
rs9906944	17	44,446,419	*IGF2BP1*	0.82	0.92	0.95	0.98	1.00
rs543874	1	176,156,103	*SEC16B*	0.94	0.99	1.00	1.00	1.00
**Loci identified in sex-specific all ancestry analyses**								
rs3761445	22	36,925,357	*PLA2G6*	0.98	0.99	1.00	1.00	1.00
rs757318	19	18,681,308	*CRTC1*	0.80	0.93	0.99	1.00	1.00

SUPPLEMENTARY NOTES

DETAILED ACKNOWLEDGEMENTS

1. General acknowledgements

D. Timothy Bishop, Mark M. Iles and Julia A. Newton Bishop are supported by: Cancer Research UK Award C588/A19167 and NIH CA083115.

Claude Bouchard is supported by the John W. Barton Sr Chair in Genetics and Nutrition

Martin L Buchkovich is supported by T32GM067553 and American Heart Association 13PRE16930025.

Abbas Dehghan is supported by NWO grant (veni, 916.12.154) and the EUR Fellowship.

Panos Deloukas's work forms part of the research themes contributing to the translational research portfolio of Barts Cardiovascular Biomedical Research Unit which is supported and funded by the National Institute for Health Research.

Alexander W Drong is supported by Wellcome Trust (093933Z/10/Z).

David M Evans is funded by an Australian Research Council Future Fellowship (FT130101709).

Mario Falchi is supported by the British Skin Foundation (grant 5044i).

Terrence S. Furey acknowledges support by the University Cancer Research Fund at UNC Chapel Hill.

Erik Ingelsson is supported by grants from the Swedish Research Council (VR; grant no. 2012-1397), Swedish Diabetes Foundation (Diabetesfonden; grant no. 2013-024), Swedish Heart-Lung Foundation (20140422), Knut och Alice Wallenberg Foundation, European Research Council (ERC-StG-335395) and Göran Gustafsson Foundation.

Anne E Justice is funded through NIH grant T32HL007055-36 and an AHA Postdoctoral Fellowship.

Douglas P. Kiel was supported by grant R01 AR 41398 from the National Institute for Arthritis, Musculoskeletal and Skin Diseases

Tuomas O. Kilpeläinen was supported by the Danish Council for Independent Research (DFF-1333-00124 and Sapere Aude program grant DFF-1331-00730B)

Mika Kivimaki is supported by the UK Medical Research Council (K013351) and the Economic and Social Research Council.

Cecilia Lindgren is supported by Wellcome Trust (086596/Z/08/Z) and the Li Ka Shing Foundation.

Reedik Mägi is funded by European Commission under the Marie Curie Intra-European Fellowship, Estonian Government (grant #SF0180142s08), and the Development Fund of the University of Tartu (grant SP1GVARENG).

Mark McCarthy is supported by Wellcome Trust Senior Investigator Wellcome #098381, Wellcome #090532, Framework VII: HEALTH-F4-2007-201413 (ENGAGE), MRC: MR/J010642/1, NIH: R01-MH101814

CGL (MrOS) receives support from a VA Clinical Science Research and Development Career Development Award, Project number 5IK2CW000729-02.

Karen L Mohlke is supported by National Institutes of Health grants R01DK072193, R01DK093757 and R21DA027040.

Andrew P Morris is a Wellcome Trust Senior Fellow in Basic Biomedical Science and acknowledges funding from the Wellcome Trust under awards WT098017 and WT090532.

Carrie M Nielson is supported by NIAMS K01 AR062655

Kari E North is supoorted by NIH/NHLBI R01DK089256.

Laura Pascoe is supported by a joint BBSRC and Unilever UK Ltd case PhD studentship.

Tune H Pers is supported by The Danish Council for Independent Research Medical Sciences (FSS), The Alfred Benzon Foundation and The Lundbeck Foundation.

J Brent Richards acknowledges funding from the Canadian Institute of Health Research

Jian Yang is supported by the Australian Research Council (DP130102666) and the Sylvia & Charles Viertel Charitable Foundation.

2. Cohort acknowledgements

AGES-Reykjavik: This study has been funded by NIH contract N01-AG-1-2100, the NIA Intramural Research Program, Hjartavernd (the Icelandic Heart Association), and the Althingi (the Icelandic Parliament). The study is approved by the Icelandic National Bioethics Committee, VSN: 00-063. The researchers are indebted to the participants for their willingness to participate in the study.

Amish: We gratefully acknowledge our Amish liaisons, field workers and clinic staff and the extraordinary cooperation and support of the Amish community without which these studies would not have been possible. The Amish studies are supported by grants and contracts from the NIH, including U01 HL072515-06, U01 HL84756, U01HL105198, U01 GM074518, F32AR059469, the University of Maryland General Clinical Research Center, grant M01 RR 16500, by the T32 training grant AG000219, and by National Research Initiative Competitive Grant no. 2007-35205-17883 from the USDA National Institute of Food and Agriculture. We thank our Amish research volunteers for their long-standing partnership in research, and the research staff at the Amish Research Clinic for their hard work and dedication.

ARIC: The Atherosclerosis Risk in Communities Study is carried out as a collaborative study supported by National Heart, Lung, and Blood Institute contracts (HHSN268201100005C, HHSN268201100006C, HHSN268201100007C, HHSN268201100008C, HHSN268201100009C, HHSN268201100010C, HHSN268201100011C, and HHSN268201100012C), R01HL087641, R01HL59367 and R01HL086694; National HumanGenome Research Institute contract U01HG004402; and National Institutes of Health contract HHSN268200625226C. The authors thank the staff and participants of the ARIC study for their important contributions. Infrastructure was partly supported by Grant Number UL1RR025005, a component of the National Institutes of Health and NIH Roadmap for Medical Research.

BASEII (Berlin Aging Study II): The Berlin Aging Study II is supported by the German Federal Ministry of Education and Research (Bundesministerium für Bildung und Forschung, BMBF) under grant numbers #16SV5536K, #16SV5537, #16SV5538, and #16SV5837).

BLSA: The BLSA was supported by the Intramural Research Program of the NIH, National Institute on Aging.

B-PROOF: B-PROOF is supported and funded by The Netherlands Organization for Health Research and Development (ZonMw, Grant 6130.0031), the Hague; unrestricted grant from NZO (Dutch Dairy Association), Zoetermeer; NCHA (Netherlands Consortium Healthy Ageing) Leiden/Rotterdam; Ministry of Economic Affairs, Agriculture and Innovation (project KB-15-004-003), the Hague; Wageningen University, Wageningen; VU University Medical Center, Amsterdam; Erasmus MC, Rotterdam. All organizations are based in the Netherlands. The sponsors do not have any role in the design or implementation of the study, data collection, data management, data analysis, data interpretation, or in the preparation, review, or approval of the manuscript.

Cardiovascular Health Study: This CHS research was supported by NHLBI contracts HHSN268201200036C, HHSN268200800007C, N01HC55222, N01HC85079, N01HC85080,

N01HC85081, N01HC85082, N01HC85083, N01HC85086; and NHLBI grants U01HL080295, R01HL087652, R01HL105756, R01HL103612, 1R01AG031890 and R01HL120393 with additional contribution from the National Institute of Neurological Disorders and Stroke (NINDS). Additional support was provided through R01AG023629 from the National Institute on Aging (NIA). A full list of principal CHS investigators and institutions can be found at CHS-NHLBI.org. The provision of genotyping data was supported in part by the National Center for Advancing Translational Sciences, CTSI grant UL1TR000124, and the National Institute of Diabetes and Digestive and Kidney Disease Diabetes Research Center (DRC) grant DK063491 to the Southern California Diabetes Endocrinology Research Center. The content is solely the responsibility of the authors and does not necessarily represent the official views of the National Institutes of Health. All analyses conform to CHS Genetics Policies, which were revised in 1998-99. Individuals who did not consent to genetic studies were excluded.

CoLaus: The CoLaus study was and is supported by research grants from GlaxoSmithKline, the Faculty of Biology and Medicine of Lausanne, and the Swiss National Science Foundation (grants 33CSCO-122661, 33CS30-139468 and 33CS30-148401). The authors also express their gratitude to the participants in the Lausanne CoLaus study and to the investigators who have contributed to the study in particular Gérard Waeber, Vincent Mooser and Dawn Waterworth, and the research nurses for data collection.

COROGENE: This study has been funded by the Academy of Finland (grant numbers 139635, 129494, 118065, 129322, 250207), the Orion-Farmos Research Foundation, the Finnish Foundation for Cardiovascular Research, and the Sigrid Jusélius Foundation. We are grateful for the THL DNA laboratory for its skillful work to produce the DNA samples used in this study. We thank the Sanger Institute genotyping facilities for genotyping the samples.

CROATIA-Vis: The CROATIA-Vis study in the Croatian island of Vis was supported through the grants from the Medical Research Council UK to H.C., A.F.W. and I.R.; and Ministry of Science, Education and Sport of the Republic of Croatia to I.R. (number 108-1080315-0302) and the European Union framework program 6 EUROSPAN project (contract no. LSHG-CT-2006-018947). We would like to acknowledge the invaluable contributions of the recruitment team (including those from the Institute of Anthropological Research in Zagreb) in Vis, the administrative teams in Croatia and Edinburgh and the people of Vis. The SNP genotyping for the CROATIA-Vis cohort was performed in the core genotyping laboratory of the Wellcome Trust Clinical Research Facility at the Western General Hospital, Edinburgh, Scotland.

DESIR: This study was supported in part by grants from SFD ("Société Francophone du Diabète"), CPER ("Contrat de Projets État-Région"), and ANR ("Agence Nationale de la Recherche"). The D.E.S.I.R. study has been supported by INSERM contracts with CNAMTS, Lilly, Novartis Pharma and Sanofi-Aventis; by INSERM (Réseaux en Santé Publique, Interactions entre les déterminants de la santé), Cohortes Santé TGIR, the Association Diabète Risque Vasculaire, the Fédération Française de Cardiologie, La Fondation de France, ALFEDIAM, ONIVINS, Ardix Medical, Bayer Diagnostics, Becton Dickinson, Cardionics, Merck Santé, Novo Nordisk, Pierre Fabre, Roche, Topcon. The D.E.S.I.R. Study Group. INSERM U780: B. Balkau, MA Charles, P. Ducimetière, E. Eschwège; INSERM U367: F. Alhenc-Gelas; CHU D'Angers: Y. Gallois, A. Girault; Bichat Hospital: F. Fumeron, M. Marre, R Roussel; CHU Rennes: F Bonnet; CNRS UMR8090, Lille: S. Cauchi, P. Froguel; Centres d'Examens de Santé: Alençon, Angers, Blois, Caen, Chartres, Chateauroux, Cholet, Le Mans, Orleans Tours; Institute de Recherche Médecine Générale: J. Cogneau; General practitioners of the region; Institute inter-Regional pour la Santé: C. Born, E. Caces, M. Cailleau, N Copin, O Lantieri, J.G. Moreau, F. Rakotozafy, J. Tichet, S. Vol.

DILGOM: The DILGOM study has been funded by the Academy of Finland (grant numbers 139635, 129494, 118065, 129322, 250207, 136895, 263836), the Orion-Farmos Research Foundation, the Finnish Foundation for Cardiovascular Research, and the Sigrid Jusélius Foundation. We are grateful for the THL DNA laboratory for its skillful work to produce the DNA samples used in this study.

Ely: We are grateful to all the volunteers and to the staff of St. Mary's Street Surgery, Ely and the study team. The Ely Study was funded by the MRC (MC_U106179471) and Diabetes UK. Genotyping in the Ely and Fenland studies was supported in part by an MRC-GlaxoSmithKline pilot programme grant (G0701863).

EPIC Norfolk: The EPIC Norfolk diabetes case cohort study is nested within the EPIC Norfolk Study, which is supported by programme grants from the Medical Research Council, and Cancer Research UK and with additional support from the European Union, Stroke Association, British Heart Foundation, Research into Ageing, Department of Health, The Wellcome Trust and the Food Standards Agency. Genotyping was in part supported by the MRC-GSK pilot programme grant. We acknowledge the contribution of the staff and participants of the EPIC-Norfolk Study.

ERF: The ERF study as a part of EUROSPAN (European Special Populations Research Network) was supported by European Commission FP6 STRP grant number 018947 (LSHG-CT-2006-01947) and also received funding from the European Community's Seventh Framework Programme (FP7/2007-2013)/grant agreement HEALTH-F4-2007-201413 by the European Commission under the programme "Quality of Life and Management of the Living Resources" of 5th Framework Programme (no. QLG2-CT-2002-01254). The ERF study was further supported by ENGAGE consortium and CMSB. High-throughput analysis of the ERF data was supported by joint grant from Netherlands Organisation for Scientific Research and the Russian Foundation for Basic Research (NWO-RFBR 047.017.043). ERF was further supported by the ZonMw grant (project 91111025). We are grateful to all study participants and their relatives, general practitioners and neurologists for their contributions and to P. Veraart for her help in genealogy, J. Vergeer for the supervision of the laboratory work and P. Snijders for his help in data collection.

FamHS: 1R01DK8925601 and 5R01DK075681 (IBB).

Fels Longitudinal Study: This Fels Longitudinal Study research was supported by National Institutes of Health grants R01-HD012252, R01-HD053685, R01-DK064391, and R01-AR052147. We also thank the Fels Longitudinal Study participants and data collection staff.

Fenland: The Fenland Study is funded by the Wellcome Trust and the Medical Research Council (MC_U106179471). We are grateful to all the volunteers for their time and help, and to the General Practitioners and practice staff for assistance with recruitment. We thank the Fenland Study Investigators and the Fenland Study co-ordination team, field epidemiology team, data team, and laboratory team as well as the MRC Epidemiology Unit Functional Group team members for business operations, IT and research governance.

Framingham Study: This research was conducted in part using data and resources from the Framingham Heart Study of the National Heart Lung and Blood Institute of the National Institutes of Health and Boston University School of Medicine. The analyses reflect intellectual input and resource development from the Framingham Heart Study investigators participating in

the SNP Health Association Resource (SHARe) project. This work was partially supported by the National Heart, Lung and Blood Institute's Framingham Heart Study (Contract No. N01-HC-25195) and its contract with Affymetrix, Inc for genotyping services (Contract No. N02-HL-6-4278). A portion of this research utilized the Linux Cluster for Genetic Analysis (LinGAII) funded by the Robert Dawson Evans Endowment of the Department of Medicine at Boston University School of Medicine and Boston Medical Center. Whole body Dual Energy X-ray Absorptiometry data was supported by the National Institute for Arthritis, Musculoskeletal and Skin Diseases and the National Institute on Aging R01 AR/AG 41398.

GxE: Our chief acknowledgement is to the participants in these studies for their willingness to contribute. We also thank Nurses Orgen Brown and Diedre Thomas for assistance with recruitment as well as past and present Laboratory technologists and drivers at TMRU for their invaluable technical assistance. This work was supported by NIH Grants R01HL53353 and R01DK075787.

GOOD: Financial support was received from the Swedish Research Council, the Swedish Foundation for Strategic Research, the ALF/LUA research grant in Gothenburg, the Lundberg Foundation, the Torsten and Ragnar Söderberg's Foundation, the Novo Nordisk Foundation, and the European Commission grant HEALTH-F2-2008-201865-GEFOS.

GOYA: This study was conducted as part of the activities of the 'Gene-diet Interactions in Obesity' project (GENDINOB, www.gendinob.dk) and the MRC centre for Causal Analyses in Translational Epidemiology (MRC CAiTE). We thank the staff of the Copenhagen City Heart Study for their skillful examination of the study subjects in collection of baseline and follow-up data.

H2000: The Health 2000 Study was funded by the National Institute for Health and Welfare (THL), the Finnish Centre for Pensions (ETK), the Social Insurance Institution of Finland (KELA), the Local Government Pensions Institution (KEVA) and other organizations listed on the website of the survey (http://www.terveys2000.fi). We are grateful for the THL DNA laboratory for its skillful work to produce the DNA samples used in this study. We thank the Sanger Institute genotyping facilities for genotyping the GenMets subcohort.

HABC (Health ABC): The Health ABC Study was supported by NIA contracts N01AG62101, N01AG62103, and N01AG62106 and, in part, by the NIA Intramural Research Program. The genome-wide association study was funded by NIA grant 1R01AG032098-01A1 to Wake Forest University Health Sciences and genotyping services were provided by the Center for Inherited Disease Research (CIDR). CIDR is fully funded through a federal contract from the National Institutes of Health to The Johns Hopkins University, contract number HHSN268200782096C. This study utilized the high-performance computational capabilities of the Biowulf Linux cluster at the National Institutes of Health, Bethesda, Md. (http://biowulf.nih.gov).

HBCS: We thank all study participants as well as everybody involved in the Helsinki Birth Cohort Study. Helsinki Birth Cohort Study has been supported by grants from the Academy of Finland (130326, 134791, 263924), the Finnish Diabetes Research Society, Folkhälsan Research Foundation, Novo Nordisk Foundation, Finska Läkaresällskapet, Signe and Ane Gyllenberg Foundation, Ahokas Foundation, Emil Aaltonen Foundation, Juho Vainio Foundation, Finnish Foundation for Pediatric Research, the Finnish Special Governmental Subsidy for Health Sciences, Samfundet Folkhälsan, Liv och Hälsa, and Wellcome Trust (grant number WT089062). We are grateful for the THL DNA laboratory for its skillful work to produce

the DNA samples used in this study. We thank the Sanger Institute genotyping facilities for genotyping the samples.

HEALTH2006: The Health2006 study was financially supported by grants from the Velux Foundation; the Danish Medical Research Council, Danish Agency for Science, Technology and Innovation; the Aase and Ejner Danielsens Foundation; ALK-Abello´ A/S (Hørsholm, Denmark), Timber Merchant Vilhelm Bangs Foundation, MEKOS Laboratories Denmark) and Research Centre for Prevention and Health, the Capital Region of Denmark. This project was also funded by the Lundbeck Foundation and produced by The Lundbeck Foundation Centre for Applied Medical Genomics in Personalised Disease Prediction, Prevention and Care (LuCamp, www.lucamp.org). The Novo Nordisk Foundation Center for Basic Metabolic Research is an independent Research Center at the University of Copenhagen partially funded by an unrestricted donation from the Novo Nordisk Foundation (www.metabol.ku.dk).

HERITAGE Family Study: The HERITAGE Family Study is supported by the National Heart, Lung, and Blood Institute Grant HL-45670.

Johnston County Osteoarthritis Project: The Johnston County Osteoarthritis Project is supported in part by cooperative agreements S043, S1734, and S3486 from the Centers for Disease Control and Prevention/Association of Schools of Public Health; the NIAMS Multipurpose Arthritis and Musculoskeletal Disease Center grant 5-P60-AR30701; and the NIAMS Multidisciplinary Clinical Research Center grant 5P60 AR49465-03. Genotyping services were provided by Algynomics company.

KoGES: This work was supported by the National Genome Research Institute, Korean Center for Disease Control and Prevention (2001-2003-348-6111-221, 2004-347-6111-213, and 2005-347-2400-2440-215). This research was supported by the National Genome Research Institute, the Korean Center for Disease Control and Prevention (contract #2001-347-6111-221, 2002-347-6111-221, 2003-347-6111-221, 2004-E71001-00, 2005-E71001-00, 2006-E71005-00, 2006-E71006-00, 2007-E71001-00, 2007-E71003-00, 2008-E71001-00, 2008-E71005-00, 2009-E71002-00, 2009-E71007-00, 2010-E71001-00, 2010-E71004-00, 2011-E71004-00, 2011-E71008-00, 2012-E71008-00, 2012-E71005-00).

KORA (F3 and S4/F4): The KORA research platform (KORA, Cooperative Health Research in the Region of Augsburg) was initiated and financed by the Helmholtz Zentrum München-German Research Center for Environmental Health, which is funded by the German Federal Ministry of Education and Research and by the State of Bavaria. Furthermore, KORA research was supported within the Munich Center of Health Sciences (MC Health), Ludwig-Maximilians-Universität, as part of LMUinnovativ.

LEIPZIG_ADULT_IFB: This work was supported by the Kompetenznetz Adipositas (Competence network for Obesity) funded by the Federal Ministry of Education and Research (German Obesity Biomaterial Bank; FKZ 01GI1128; AD2-6E, AD2-6E95, AD2-6E96, AD2-6E97), a grant from Deutsche Forschungsgemeinschaft the SFB 1052 “Obesity mechanisms” (A01, B01, B03, C01).

LOLIPOP: The LOLIPOP study is supported by the National Institute for Health Research (NIHR) Comprehensive Biomedical Research Centre Imperial College Healthcare NHS Trust, the British Heart Foundation (SP/04/002), the Medical Research Council (G0601966,G0700931), the Wellcome Trust (084723/Z/08/Z), the NIHR (RP-PG-0407-10371), European Union FP7 (EpiMigrant, 279143) and Action on Hearing Loss (G51). The work was

carried out in part at the NIHR/Wellcome Trust Imperial Clinical Research Facility. The views expressed are those of the author(s) and not necessarily those of the Imperial College Healthcare NHS Trust, the NIHR or the Department of Health. We thank the participants and research staff who made the study possible.

MAP (Rush Memory and Aging Project): The MAP data used in this analysis was supported by National Institute on Aging grants R01AG17917, R01AG15819, R01AG30146, the Illinois Department of Public Health, and the Translational Genomics Research Institute.

METSIM: This work was supported by the Academy of Finland (A.S. and M.L.), the Finnish Diabetes Research Foundation (M.L.), the Finnish Cardiovascular Research Foundation (M.L.), Strategic Research Funding from the University of Eastern Finland (M.L.), EVO Grant 5263 from the Kuopio University Hospital (M.L.).

MrOS: The Osteoporotic Fractures in Men (MrOS) Study is supported by National Institutes of Health funding. The following institutes provide support: the National Institute on Aging (NIA), the National Institute of Arthritis and Musculoskeletal and Skin Diseases (NIAMS), the National Center for Advancing Translational Sciences (NCATS), and NIH Roadmap for Medical Research under the following grant numbers: U01 AG027810, U01 AG042124, U01 AG042139, U01 AG042140, U01 AG042143, U01 AG042145, U01 AG042168, U01 AR066160, UL1 TR000128, R01 AR051124, and RC2 AR058973.

ORCADES: ORCADES was supported by the Chief Scientist Office of the Scottish Government, the Royal Society, the MRC Human Genetics Unit, Arthritis Research UK and the European Union framework program 6 EUROSPAN project (contract no. LSHG-CT-2006-018947). DNA extractions were performed at the Wellcome Trust Clinical Research Facility in Edinburgh. We would like to acknowledge the invaluable contributions of Lorraine Anderson and the research nurses in Orkney, the administrative team in Edinburgh and the people of Orkney.

PIVUS: Genotyping was performed by the SNP&SEQ Technology Platform in Uppsala (www.genotyping.se). We thank Tomas Axelsson, Ann-Christine Wiman and Caisa Pöntinen for their excellent assistance with genotyping. The SNP Technology Platform is supported by Uppsala University, Uppsala University Hospital and the Swedish Research Council for Infrastructures.

PREVEND: PREVEND genetics is supported by the Dutch Kidney Foundation (Grant E033), the EU project grant GENECURE (FP-6 LSHM CT 2006 037697), the National Institutes of Health (grant 2R01LM010098), The Netherlands organisation for health research and development (NWO-Groot grant 175.010.2007.006, NWO VENI grant 916.761.70, ZonMw grant 90.700.441), the Netherlands Heart Foundation (grant NHS2010B280) and the Dutch Inter University Cardiology Institute Netherlands (ICIN).

QFS: The Quebec Family Study (QFS) was funded by multiple grants from the Medical Research Council of Canada and the Canadian Institutes of Health Research. This work was supported by a team grant from the Canadian Institutes for Health Research (FRCN-CCT-83028).

RISC: The RISC Study is supported by European Union grant QLG1-CT-2001-01252 and AstraZeneca.

RSI and RSIII: RSI and RSIII: The generation and management of GWAS genotype data for the Rotterdam Study (RS I, RS II, RS III) was executed by the Human Genotyping Facility of the Genetic Laboratory of the Department of Internal Medicine, Erasmus MC, Rotterdam, The Netherlands. The GWAS datasets are supported by the Netherlands Organisation of Scientific Research NWO Investments (nr. 175.010.2005.011, 911-03-012), the Genetic Laboratory of the Department of Internal Medicine, Erasmus MC, the Research Institute for Diseases in the Elderly (014-93-015; RIDE2), the Netherlands Genomics Initiative (NGI)/Netherlands Organisation for Scientific Research (NWO) Netherlands Consortium for Healthy Aging (NCHA), project nr. 050-060-810. We thank Pascal Arp, Mila Jhamai, Marijn Verkerk, Lizbeth Herrera and Marjolein Peters, MSc, and Carolina Medina-Gomez, MSc, for their help in creating the GWAS database, and Karol Estrada, PhD, Yurii Aulchenko, PhD, and Carolina Medina-Gomez, MSc, for the creation and analysis of imputed data. We would like to thank Karol Estrada PhD, Fernando Rivadeneira PhD, and Anis Abuseiris (Erasmus MC Rotterdam, The Netherlands), for their help in creating GRIMP, and we thank BigGRID for access to their grid computing resources.
The Rotterdam Study is funded by Erasmus Medical Center and Erasmus University, Rotterdam, Netherlands Organization for the Health Research and Development (ZonMw), the Research Institute for Diseases in the Elderly (RIDE), the Ministry of Education, Culture and Science, the Ministry for Health, Welfare and Sports, the European Commission (DG XII), and the Municipality of Rotterdam. The authors are grateful to the study participants, the staff from the Rotterdam Study and the participating general practitioners and pharmacists.

SHIP/SHIP-TREND: SHIP is part of the Community Medicine Research net of the University of Greifswald, Germany, which is funded by the Federal Ministry of Education and Research (grants no. 01ZZ9603, 01ZZ0103, and 01ZZ0403), the Ministry of Cultural Affairs as well as the Social Ministry of the Federal State of Mecklenburg-West Pomerania, and the network 'Greifswald Approach to Individualized Medicine (GANI_MED)' funded by the Federal Ministry of Education and Research (grant 03IS2061A). Genome-wide data have been supported by the Federal Ministry of Education and Research (grant no. 03ZIK012) and a joint grant from Siemens Healthcare, Erlangen, Germany and the Federal State of Mecklenburg-West Pomerania. The University of Greifswald is a member of the 'Center of Knowledge Interchange' program of the Siemens AG and the Caché Campus program of the InterSystems GmbH.

SOF: The Study of Osteoporotic Fractures (SOF) is supported by National Institutes of Health funding. The National Institute on Aging (NIA) provides support under the following grant numbers: R01 AG005407, R01 AR35582, R01 AR35583, R01 AR35584, R01 AG005394, R01 AG027574, and R01 AG027576.

Sorbs: This work was supported by grants from the German Research Council (SFB- 1052 "Obesity mechanisms" to Michael Stumvoll, Anke Tönjes and Peter Kovacs), from the German Diabetes Association (to Anke Tönjes and Peter Kovacs) and from the DHFD (Diabetes Hilfs- und Forschungsfonds Deutschland to Michael Stumvoll and Peter Kovacs). Peter Kovacs is funded by the Boehringer Ingelheim Foundation. We thank all those who participated in the study. Sincere thanks are given to Knut Krohn (Microarray Core Facility of the Interdisciplinary Centre for Clinical Research, University of Leipzig) for the genotyping support.

SPT: Our chief acknowledgement is to the participants in these studies for their willingness to contribute. We also thank Nurses Orgen Brown and Diedre Thomas for assistance with recruitment as well as past and present Laboratory technologists and drivers at TMRU for their invaluable technical assistance. This work was supported by NIH Grants R01HL53353 and R01DK075787.

TwinsUK: The study was funded by the Wellcome Trust; European Community's Seventh Framework Programme (FP7/2007-2013). The study also receives support from the National Institute for Health Research (NIHR) Clinical Research Facility at Guy's & St Thomas' NHS Foundation Trust and NIHR Biomedical Research Centre based at Guy's and St Thomas' NHS Foundation Trust and King's College London. Tim Spector is an NIHR senior Investigator and is holder of an ERC Advanced Principal Investigator award. SNP Genotyping was performed by The Wellcome Trust Sanger Institute and National Eye Institute via NIH/CIDR.

ULSAM: Genotyping was performed by the SNP&SEQ Technology Platform in Uppsala (www.genotyping.se). We thank Tomas Axelsson, Ann-Christine Wiman and Caisa Pöntinen for their excellent assistance with genotyping. The SNP Technology Platform is supported by Uppsala University, Uppsala University Hospital and the Swedish Research Council for Infrastructures.

Whitehall II: The WHII study has been supported by grants from the Medical Research Council; British Heart Foundation; Health and Safety Executive; Department of Health; National Heart Lung and Blood Institute (NHLBI: HL36310) and National Institute on Aging (AG13196), US, NIH; Agency for Health Care Policy Research (HS06516); Medical Research Council (K013351); and the John D and Catherine T MacArthur Foundation Research Networks on Successful Midlife Development and Socio-economic Status and Health.

YFS: The Young Finns Study has been financially supported by the Academy of Finland: grants 134309 (Eye), 126925, 121584, 124282, 129378 (Salve), 117787 (Gendi), and 41071 (Skidi), the Social Insurance Institution of Finland, Kuopio, Tampere and Turku University Hospital Medical Funds (grant 9M048 and 9N035 for TeLeht), Juho Vainio Foundation, Paavo Nurmi Foundation, Finnish Foundation of Cardiovascular Research and Finnish Cultural Foundation, Tampere Tuberculosis Foundation and Emil Aaltonen Foundation (T.L). The THL DNA laboratory for its skillful work to produce the DNA samples used in this study and the expert technical assistance in the statistical analyses by Ville Aalto and Irina Lisinen are gratefully acknowledged.

3. Contributing Consortia

The ADIPOGen Consortium

Dastani, Z. et al. Novel loci for adiponectin levels and their influence on type 2 diabetes and metabolic traits: a multi-ethnic meta-analysis of 45,891 individuals. PLoS Genet 8, e1002607 (2012).

Zari Dastani, Marie-France Hivert, Nicholas Timpson, John R.B Perry, Xin Yuan, Robert A. Scott, Peter Henneman, Iris M. Heid, Jorge R. Kizer, Leo-Pekka Lyytikainen, Christian Fuchsberger, Toshiko Tanaka, Andrew P. Morris, Kerrin Small, Aaron Isaacs, Marian Beekman, Stefan Coassin, Kurt Lohman, Lu Qi, Stavroula Kanoni, James S. Pankow, Hae-Won Uh, Ying Wu, Aurelian Bidulescu, Laura J. Rasmussen-Torvik, Celia M.T. Greenwood, Martin Ladouceur, Jonna Grimsby, Alisa K. Manning, Ching-Ti Liu, Jaspal Kooner, Vincent E. Mooser, Peter Vollenweider, Karen A. Kapur, John Chambers, Nicholas J. Wareham, Claudia Langenberg, Rune Frants, Ko Willems-vanDijk, Ben A. Oostra, Sara M. Willems, Claudia Lamina, Thomas Winkler, Bruce M. Psaty, Russell P. Tracy, Jennifer Brody, Ida Chen, Jorma Viikari, Mika Kähönen, Peter P. Pramstaller, -46 David M. Evans, Beate St Pourcain, Naveed Sattar, Andy Wood, Stefania Bandinelli, Olga D. Carlson, Josephine M.

Egan, Stefan Böhringer, Diana van Heemst, Lyudmyla Kedenko, Kati Kristiansson, Marja-Liisa Nuotio, Britt-Marie Loo, Tamara Harris, Melissa Garcia, Alka Kanaya, Margot Haun, Norman Klopp, H. Erich Wichmann, -60 Panos Deloukas, Efi Katsareli, David J. Couper, Bruce B. Duncan, Margreet Kloppenburg, Linda S. Adair, Judith B. Borja, DIAGRAM+ Consortium, MAGIC Consortium, GLGC Investigators, MuTHER Consortium, James G. Wilson, Solomon Musani, Xiuqing Guo, Toby Johnson, Robert Semple, Tanya M. Teslovich, Matthew A. Allison, Susan Redline, Sarah G. Buxbaum, Karen L. Mohlke, Ingrid Meulenbelt, Christie M. Ballantyne, George V. Dedoussis, Frank B. Hu, Yongmei Liu, Bernhard Paulweber, Timothy D. Spector, P. Eline Slagboom, Luigi Ferrucci, Antti Jula, Markus Perola, Olli Raitakari, Jose C. Florez, -82 Veikko Salomaa, Johan G. Eriksson, Timothy M. Frayling, Andrew A Hicks, Terho Lehtimäki, George Davey Smith, David S. Siscovick, Florian Kronenberg, Cornelia van Duijn, Ruth J.F. Loos, Dawn M. Waterworth, James B. Meigs, Josee Dupuis, John Brent Richards.

The CARDIoGRAMplusC4D Consortium

The CARDIoGRAMplusC4D Consortium, A comprehensive 1000 Genomes-based genome-wide association meta-analysis of coronary artery disease. Nat Genet 47, 1121-30 (2015).

Panos Deloukas, Stavroula Kanoni, Christina Willenborg, Martin Farrall, Themistocles L Assimes, John R Thompson, Erik Ingelsson, Danish Saleheen, Jeanette Erdmann, Benjamin A Goldstein, Kathleen Stirrups, Inke R König, Jean-Baptiste Cazier, Åsa Johansson, Alistair S Hall, Jong-Young Lee, Cristen J Willer, John C Chambers, Tõnu Esko, Lasse Folkersen, Anuj Goel, Elin Grundberg, Aki S Havulinna, Weang K Ho, Jemma C Hopewell, Niclas Eriksson, Marcus E Kleber, Kati Kristiansson, Per Lundmark, Leo-Pekka Lyytikäinen, Suzanne Rafelt, Dmitry Shungin, Rona J Strawbridge, Gudmar Thorleifsson, Emmi Tikkanen, Natalie Van Zuydam, Benjamin F Voight, Lindsay L Waite, Weihua Zhang, Andreas Ziegler, Devin Absher, David Altshuler-, Anthony J Balmforth, Inês Barroso, Peter S Braund, Christof Burgdorf, Simone Claudi-Boehm, David Cox, Maria Dimitriou, Ron Do, CARDIOGENICS Consortium, DIAGRAM Consortium, Alex S F Doney, NourEddine El Mokhtari, Per Eriksson, Krista Fischer, Pierre Fontanillas, Anders Franco-Cereceda, Bruna Gigante, Leif Groop, Stefan Gustafsson, Jörg Hager, Göran Hallmans, Bok-Ghee Han, Sarah E Hunt, Hyun M Kang, Thomas Illig, Thorsten Kessler, Joshua W Knowles, Genovefa Kolovou, Johanna Kuusisto, Claudia Langenberg, Cordelia Langford, Karin Leander, Marja-Liisa Lokki, Anders Lundmark, Mark I McCarthy, Christa Meisinger, Olle Melander, Evelin Mihailov, Seraya Maouche, Andrew D Morris, Martina Müller-Nurasyid–, MuTHER Consortium, Kjell Nikus, John F Peden, N William Rayner, Asif Rasheed, Silke Rosinger, Diana Rubin, Moritz P Rumpf, Arne Schäfer, Mohan Sivananthan, Ci Song, Alexandre F R Stewart, Sian-Tsung Tan, Gudmundur Thorgeirsson, C Ellen van der Schoot, Peter J Wagner, Wellcome Trust Case Control Consortium, George A Wells, Philipp S Wild, Tsun-Po Yang, Philippe Amouyel, Dominique Arveiler, Hanneke Basart, Michael Boehnke, Eric Boerwinkle, Paolo Brambilla, Francois Cambien, Adrienne L Cupples, Ulf de Faire, Abbas Dehghan, Patrick Diemert, Stephen E Epstein, Alun Evans, Marco M Ferrario, Jean Ferrières, Dominique Gauguier, Alan S Go, Alison H Goodall, Villi Gudnason, Stanley L Hazen, Hilma Holm, Carlos Iribarren, Yangsoo Jang, Mika Kähönen, Frank Kee, Hyo-Soo Kim, Norman Klopp, Wolfgang Koenig, Wolfgang Kratzer, Kari Kuulasmaa, Markku Laakso, Reijo Laaksonen, Ji-Young Lee, Lars Lind, Willem H Ouwehand, Sarah Parish, Jeong E Park, Nancy L Pedersen, Annette Peters, Thomas Quertermous, Daniel J Rader, Veikko Salomaa, Eric Schadt, Svati H Shah, Juha Sinisalo, Klaus Stark, Kari Stefansson, David-Alexandre Trégouët, Jarmo Virtamo, Lars Wallentin, Nicholas Wareham, Martina E Zimmermann, Markku S Nieminen, Christian Hengstenberg, Manjinder S Sandhu, Tomi Pastinen, Ann-Christine Syvänen, G Kees Hovingh, George Dedoussis, Paul W Franks–, Terho Lehtimäki, Andres Metspalu, Pierre A Zalloua, Agneta Siegbahn, Stefan Schreiber, Samuli Ripatti, Stefan S Blankenberg, Markus Perola,

Robert Clarke, Bernhard O Boehm, Christopher O'Donnell, Muredach P Reilly, Winfried März, Rory Collins, Sekar Kathiresan, Anders Hamsten, Jaspal S Kooner, Unnur Thorsteinsdottir, John Danesh, Colin N A Palmer, Robert Roberts, Hugh Watkins, Heribert Schunkert & Nilesh J Samani

The CHARGE Inflammation working group:

Dehghan, A. et al. Meta-analysis of genome-wide association studies in >80 000 subjects identifies multiple loci for C-reactive protein levels. Circulation 123, 731-8 (2011).

Dehghan A, Dupuis J, Barbalic M, Bis JC, Eiriksdottir G, Lu C, Pellikka N, Wallaschofski H, Kettunen J, Henneman P, Baumert J, Strachan DP, Fuchsberger C, Vitart V, Wilson JF, Pare G, Naitza S, Rudock ME, Surakka I, de Geus EJ, Alizadeh BZ, Guralnik J, Shuldiner A, Tanaka T, Zee RY, Schnabel RB, Nambi V, Kavousi M, Ripatti S, Nauck M, Smith NL, Smith AV, Sundvall J, Scheet P, Liu Y, Ruokonen A, Rose LM, Larson MG, Hoogeveen RC, Freimer NB, Teumer A, Tracy RP, Launer LJ, Buring JE, Yamamoto JF, Folsom AR, Sijbrands EJ, Pankow J, Elliott P, Keaney JF, Sun W, Sarin AP, Fontes JD, Badola S, Astor BC, Hofman A, Pouta A, Werdan K, Greiser KH, Kuss O, Meyer zu Schwabedissen HE, Thiery J, Jamshidi Y, Nolte IM, Soranzo N, Spector TD, Volzke H, Parker AN, Aspelund T, Bates D, Young L, Tsui K, Siscovick DS, Guo X, Rotter JI, Uda M, Schlessinger D, Rudan I, Hicks AA, Penninx BW, Thorand B, Gieger C, Coresh J, Willemsen G, Harris TB, Uitterlinden AG, Jarvelin MR, Rice K, Radke D, Salomaa V, Willems van Dijk K, Boerwinkle E, Vasan RS, Ferrucci L, Gibson QD, Bandinelli S, Snieder H, Boomsma DI, Xiao X, Campbell H, Hayward C, Pramstaller PP, van Duijn CM, Peltonen L, Psaty BM, Gudnason V, Ridker PM, Homuth G, Koenig W, Ballantyne CM, Witteman JC, Benjamin EJ, Perola M, Chasman DI

The CHARGE testosterone meta-analysis:

Ohlsson, C. et al. Genetic determinants of serum testosterone concentrations in men. PLoS Genet 7, e1002313 (2011).

Ohlsson C, Wallaschofski H, Lunetta KL, Stolk L, Perry JR, Koster A, Petersen AK, Eriksson J, Lehtimäki T, Huhtaniemi IT, Hammond GL, Maggio M, Coviello AD; EMAS Study Group, Ferrucci L, Heier M, Hofman A, Holliday KL, Jansson JO, Kähönen M, Karasik D, Karlsson MK, Kiel DP, Liu Y, Ljunggren O, Lorentzon M, Lyytikäinen LP, Meitinger T, Mellström D, Melzer D, Miljkovic I, Nauck M, Nilsson M, Penninx B, Pye SR, Vasan RS, Reincke M, Rivadeneira F, Tajar A, Teumer A, Uitterlinden AG, Ulloor J, Viikari J, Völker U, Völzke H, Wichmann HE, Wu TS, Zhuang WV, Ziv E, Wu FC, Raitakari O, Eriksson A, Bidlingmaier M, Harris TB,Murray A, de Jong FH, Murabito JM, Bhasin S, Vandenput L, Haring R

The DIAGRAM Consortium

Morris, A.P. et al. Large-scale association analysis provides insights into the genetic architecture and pathophysiology of type 2 diabetes. Nat Genet 44, 981-90 (2012).

Mahajan A, Go MJ, Zhang W, Below JE, Gaulton KJ, Ferreira T, Horikoshi M, Johnson AD, Ng MC, Prokopenko I, Saleheen D, Wang X, Zeggini E, Abecasis GR, Adair LS, Almgren P, Atalay M, Aung T, Baldassarre D, Balkau B, Bao Y, Barnett AH, Barroso I, Basit A, Been LF, Beilby J, Bell GI, Benediktsson R, Bergman RN, Boehm BO, Boerwinkle E, Bonnycastle LL, Burtt N, Cai Q, Campbell H, Carey J, Cauchi S, Caulfield M, Chan JC, Chang LC, Chang TJ, Chang YC, Charpentier G, Chen CH, Chen H, Chen YT, Chia KS, Chidambaram M, Chines PS, Cho NH, Cho YM, Chuang LM, Collins FS, Cornelis MC, Couper DJ, Crenshaw AT, van Dam RM, Danesh J, Das D, de Faire U, Dedoussis G, Deloukas P, Dimas AS, Dina C, Doney AS, Donnelly PJ, Dorkhan M, van Duijn C, Dupuis J, Edkins S, Elliott P, Emilsson V, Erbel R, Eriksson JG, Escobedo J, Esko T, Eury E, Florez JC, Fontanillas P, Forouhi NG, Forsen T, Fox C, Fraser RM, Frayling TM, Froguel P, Frossard P, Gao Y, Gertow K, Gieger C, Gigante B,

Grallert H, Grant GB, Grrop LC, Groves CJ, Grundberg E, Guiducci C, Hamsten A, Han BG, Hara K, Hassanali N, Hattersley AT, Hayward C, Hedman AK, Herder C, Hofman A, Holmen OL, Hovingh K, Hreidarsson AB, Hu C, Hu FB, Hui J, Humphries SE, Hunt SE, Hunter DJ, Hveem K, Hydrie ZI, Ikegami H, Illig T, Ingelsson E, Islam M, Isomaa B, Jackson AU, Jafar T, James A, Jia W, Jöckel KH, Jonsson A, Jowett JB, Kadowaki T, Kang HM, Kanoni S, Kao WH, Kathiresan S, Kato N, Katulanda P, Keinanen-Kiukaanniemi KM, Kelly AM, Khan H, Khaw KT, Khor CC, Kim HL, Kim S, Kim YJ, Kinnunen L, Klopp N, Kong A, Korpi-Hyövälti E, Kowlessur S, Kraft P, Kravic J, Kristensen MM, Krithika S, Kumar A, Kumate J, Kuusisto J, Kwak SH, Laakso M, Lagou V, Lakka TA, Langenberg C, Langford C, Lawrence R, Leander K, Lee JM, Lee NR, Li M, Li X, Li Y, Liang J, Liju S, Lim WY, Lind L, Lindgren CM, Lindholm E, Liu CT, Liu JJ, Lobbens S, Long J, Loos RJ, Lu W, Luan J, Lyssenko V, Ma RC, Maeda S, Mägi R, Männisto S, Matthews DR, Meigs JB, Melander O, Metspalu A, Meyer J, Mirza G, Mihailov E, Moebus S, Mohan V, Mohlke KL, Morris AD, Mühleisen TW, Müller-Nurasyid M, Musk B, Nakamura J, Nakashima E, Navarro P, Ng PK, Nica AC, Nilsson PM, Njølstad I, Nöthen MM, Ohnaka K, Ong TH, Owen KR, Palmer CN, Pankow JS, Park KS, Parkin M, Pechlivanis S, Pedersen NL, Peltonen L, Perry JR, Peters A, Pinidiyapathirage JM, Platou CG, Potter S, Price JF, Qi L, Radha V, Rallidis L, Rasheed A, Rathman W, Rauramaa R, Raychaudhuri S, Rayner NW, Rees SD, Rehnberg E, Ripatti S, Robertson N, Roden M, Rossin EJ, Rudan I, Rybin D, Saaristo TE, Salomaa V, Saltevo J, Samuel M, Sanghera DK, Saramies J, Scott J, Scott LJ, Scott RA, Segrè AV, Sehmi J, Sennblad B, Shah N, Shah S, Shera AS, Shu XO, Shuldiner AR, Sigurđsson G, Sijbrands E, Silveira A, Sim X, Sivapalaratnam S, Small KS, So WY, Stančáková A, Stefansson K, Steinbach G, Steinthorsdottir V, Stirrups K, Strawbridge RJ, Stringham HM, Sun Q, Suo C, Syvänen AC, Takayanagi R, Takeuchi F, Tay WT, Teslovich TM, Thorand B, Thorleifsson G, Thorsteinsdottir U, Tikkanen E, Trakalo J, Tremoli E, Trip MD, Tsai FJ, Tuomi T, Tuomilehto J, Uitterlinden AG, Valladares-Salgado A, Vedantam S, Veglia F, Voight BF, Wang C, Wareham NJ, Wennauer R, Wickremasinghe AR, Wilsgaard T, Wilson JF, Wiltshire S, Winckler W, Wong TY, Wood AR, Wu JY, Wu Y, Yamamoto K, Yamauchi T, Yang M, Yengo L, Yokota M, Young R, Zabaneh D, Zhang F, Zhang R, Zheng W, Zimmet PZ, Altshuler D, Bowden DW, Cho YS, Cox NJ, Cruz M, Hanis CL, Kooner J, Lee JY, Seielstad M, Teo YY, Boehnke M, Parra EJ, Chambers JC, Tai ES, McCarthy MI, Morris AP.

The Early Growth Genetics Consortium (Birth weight)

*Horikoshi, M. et al. New loci associated with birth weight identify genetic links between intrauterine growth and adult height and metabolism. Nat Genet **45**, 76-82 (2013).*

Horikoshi M, Mägi R, van de Bunt M, Surakka I, Sarin AP, Mahajan A, Marullo L, Thorleifsson G, Hägg S, Hottenga JJ, Ladenvall C, Ried JS, Winkler TW, Willems SM, Pervjakova N, Esko T, Beekman M, Nelson CP, Willenborg C, Wiltshire S, Ferreira T, Fernandez J, Gaulton KJ, Steinthorsdottir V, Hamsten A, Magnusson PK, Willemsen G, Milaneschi Y, Robertson NR, Groves CJ, Bennett AJ, Lehtimäki T, Viikari JS, Rung J, Lyssenko V, Perola M, Heid IM, Herder C, Grallert H, Müller-Nurasyid M, Roden M, Hypponen E, Isaacs A, van Leeuwen EM, Karssen LC, Mihailov E, Houwing-Duistermaat JJ, de Craen AJ, Deelen J, Havulinna AS, Blades M, Hengstenberg C, Erdmann J, Schunkert H, Kaprio J, Tobin MD, Samani NJ, Lind L, Salomaa V, Lindgren CM, Slagboom PE, Metspalu A, van Duijn CM, Eriksson JG, Peters A, Gieger C, Jula A, Groop L, Raitakari OT, Power C, Penninx BW, de Geus E, Smit JH, Boomsma DI, Pedersen NL, Ingelsson E, Thorsteinsdottir U, Stefansson K, Ripatti S, Prokopenko I, McCarthy MI, Morris AP; ENGAGE Consortium

The Early Growth Genetics Consortium (pubertal height)

Cousminer, D.L. et al. Genome-wide association and longitudinal analyses reveal genetic loci linking pubertal height growth, pubertal timing and childhood adiposity. Hum Mol Genet 22, 2735-47 (2013).

Diana L Cousminer, Diane J Berry, Nicholas J Timpson, Wei Ang, Elisabeth Thiering, Enda M Byrne, H Rob Taal, Ville Huikari, Jonathan P Bradfield, Marjan Kerkhof, Maria M Groen-Blokhuis, Eskil Kreiner-Møller, Marcella Marinelli, Claus Holst, Jaakko T Leinonen, John R B Perry, Ida Surakka, Olli Pietiläinen, Johannes Kettunen, Verneri Anttila, Marika Kaakinen, Ulla Sovio, Anneli Pouta, Shikta Das, Vasiliki Lagou, Chris Power, Inga Prokopenko, David M Evans, John P Kemp, Beate St Pourcain, Susan Ring, Aarno Palotie, Eero Kajantie, Clive Osmond, Terho Lehtimäki, Jorma S Viikari, Mika Kähönen, Nicole M Warrington, Stephen J Lye, Lyle J Palmer, Carla M T Tiesler, Claudia Flexeder, Grant W Montgomery, Sarah E Medland, Albert Hofman, Hakon Hakonarson, Mònica Guxens, Meike Bartels, Veikko Salomaa, The ReproGen Consortium, Joanne M Murabito, Jaakko Kaprio, Thorkild I A Sørensen, Ferran Ballester, Hans Bisgaard, Dorret I Boomsma, Gerard H Koppelman, Struan F A Grant, Vincent W V Jaddoe, Nicholas G Martin, Joachim Heinrich, Craig E Pennell, Olli T Raitakari, Johan G Eriksson, George Davey Smith, Elina Hyppönen, Marjo-Riitta Järvelin, Mark I McCarthy, Samuli Ripatti & Elisabeth Widén

GenoMEL

Falchi, M. et al. Genome-wide association study identifies variants at 9p21 and 22q13 associated with development of cutaneous nevi. Nat Genet 41, 915-9 (2009).

*Barrett, J.H. et al. Genome-wide association study identifies three new melanoma susceptibility loci. Nat Genet **43**, 1108-13 (2011).*

*Nan, H. et al. Genome-wide association study identifies nidogen 1 (NID1) as a susceptibility locus to cutaneous nevi and melanoma risk. Hum Mol Genet **20**, 2673-9 (2011).*

Barcelona: Paula Aguilera, Beatriz Alejo, Celia Badenas, Abel Caño, Cristina Carrera, Francisco Cuellar, Mireia Dominguez, Daniel Gabriel, Pol Gomenez, Pablo Iglesias, Josep Malvehy, Rosa Marti-Laborda, Montse Mila, Zighe Ogbah, Miriam Potrony, Joan-Anton Puig Butille, Susana Puig, Gemma Tell and Other members of the Melanoma Unit: Llúcia Alós, Ana Arance, Pedro Arguís, Antonio Bennassar, Oscar Chirife, Carlos Conill, Ramon Rull, Marcelo Sánchez, Sergi Vidal-Sicart, Antonio Vilalta.

Emilia-Romagna: Maria Teresa Landi, Donato Calista, Giorgio Landi, Paola Minghetti, Fabio Arcangeli, Pier Alberto Bertazzi.

Genoa: Department of Internal Medicine and Medical Specialties, Laboratory of Genetics of Rare Cancers, University of Genoa/ IRCCS AOU San Martino-IST Istituto Nazioanle epr la Ricerca sul Cancro: Giovanna Bianchi Scarrà, Paola Ghiorzo, Lorenza Pastorino, William Bruno,Virginia Andreotti, Claudia Martinuzzi, Linda Battistuzzi, Paola Origone. Medical Oncology Unit, IRCCS AOU San Martino-IST Istituto Nazionale per la Ricerca sul Cancro, Genoa :Paola Queirolo.

Glasgow: Rona Mackie, Julie Lang.

Leeds: Julia A Newton Bishop, Paul Affleck, Jennifer H Barrett, D Timothy Bishop, Jane Harrison, Mark M Iles, Juliette Randerson-Moor, Mark Harland, John C Taylor, Linda Whittaker, Kairen Kukalizch, Susan Leake, Birute Karpavicius, Sue Haynes, Tricia Mack, May Chan, Yvonne Taylor, John Davies, Paul King.

Leiden: Department of Dermatology, Leiden University Medical Centre: Nelleke A Gruis, Frans A van Nieuwpoort, Coby Out, Clasine van der Drift, Wilma Bergman, Nicole Kukutsch, Jan Nico Bouwes Bavinck. Department of Clinical Genetics, Centre of Human and Clinical Genetics, Leiden University Medical Centre: Bert Bakker, Nienke van der Stoep, Jeanet ter Huurne. Department of Dermatology, HAGA Hospital, The Hague: Han van der Rhee. Department of Dermatology, Reinier de Graaf Groep, Delft: Marcel Bekkenk. Department of Dermatology, Sint

Franciscus Gasthuis, Rotterdam: Dyon Snels, Marinus van Praag. Department of Dermatology, Ghent University Hospital, Ghent, Belgium: Lieve Brochez and colleagues. Department of Dermatology, St. Radboud University Medical Centre, Nijmegen: Rianne Gerritsen and colleagues. Department of Dermatology, Rijnland Hospital, Leiderdorp: Marianne Crijns and colleagues. Dutch patient organisation, Stichting Melanoom, Purmerend. The Netherlands Foundation for the detection of Hereditary Tumors, Leiden: Hans Vasen. ServiceXS: Wilbert van Workum, Bart Janssen, Marjolein Janssen and Suzanne Mulder
Lund: Lund Melanoma Study Group: Håkan Olsson, Christian Ingvar, Göran Jönsson, Åke Borg, Anna Måsbäck, Lotta Lundgren, Katja Baeckenhorn, Kari Nielsen, Anita Schmidt Casslén.
Norway: Oslo University Hospital: Per Helsing, Per Arne Andresen, Helge Rootwelt. University of Bergen: Lars A. Akslen, Anders Molven.
Paris: Florence Demenais, Marie-Françoise Avril, Brigitte Bressac-de Paillerets, Eve Maubec, Myriam Brossard, Amaury Vaysse, Hamida Mohamdi, Patricia Jeannin, Valérie Chaudru, Nicolas Chateigner, Eve Corda, Fabienne Lesueur, Mahaut de Lichy and the French Family Study Group including the following oncogeneticists and Dermatologists: Pascale Andry-Benzaquen, Bertrand Bachollet, Frédéric Bérard, Pascaline Berthet, Françoise Boitier, Valérie Bonadona, Jean-Louis Bonafé, Jean-Marie Bonnetblanc, Frédéric Cambazard, Olivier Caron, Frédéric Caux, Jacqueline Chevrant-Breton, Agnès Chompret (deceased), Stéphane Dalle, Liliane Demange, Olivier Dereure, Martin-Xavier Doré, Marie-Sylvie Doutre, Catherine Dugast, Laurence Faivre, Florent Grange, Philippe Humbert, Pascal Joly, Delphine Kerob, Christine Lasset, Marie Thérèse Leccia, Gilbert Lenoir, Dominique Leroux, Julien Levang, Dan Lipsker, Sandrine Mansard, Ludovic Martin, Tanguy Martin-Denavit, Christine Mateus, Jean-Loïc Michel, Patrice Morel, Laurence Olivier-Faivre, Jean-Luc Perrot, Caroline Robert, Sandra Ronger-Savle, Bruno Sassolas, Pierre Souteyrand, Dominique Stoppa-Lyonnet, Luc Thomas, Pierre Vabres, Eva Wierzbicka.
Philadelphia: David Elder, Peter Kanetsky, Jillian Knorr, Michael Ming, Nandita Mitra, Althea Ruffin, Patricia Van Belle
Poland: Tadeusz Dębniak, Jan Lubiński, Aneta Mirecka, Sławomir Ertmański. Slovenia: Srdjan Novakovic, Marko Hocevar, Barbara Peric, Petra Cerkovnik. Stockholm: Veronica Höiom, Johan Hansson.
Sydney: Graham J. Mann, Richard F. Kefford, Helen Schmid, Elizabeth A. Holland
Tel Aviv: Esther Azizi, Gilli Galore-Haskel, Eitan Friedman, Orna Baron-Epel, Alon Scope, Felix Pavlotsky, Emanuel Yakobson, Irit Cohen-Manheim, Yael Laitman, Roni Milgrom, Iris Shimoni, Evgeniya Kozlovaa.

The GLGC Consortium

Willer, C.J. et al. Discovery and refinement of loci associated with lipid levels. Nat Genet (2013).

Cristen J. Willer, Ellen M. Schmidt, Sebanti Sengupta, Gina M. Peloso, Stefan Gustafsson, Stavroula Kanoni, Andrea Ganna, Jin Chen, Martin L. Buchkovich, Samia Mora, Jacques S. Beckmann, Jennifer L. Bragg-Gresham, Hsing-Yi Chang, Ayşe Demirkan, Heleen M. Den Hertog, Ron Do, Louise A. Donnelly, Georg B. Ehret, Tõnu Esko, Mary F. Feitosa, Teresa Ferreira, Krista Fischer, Pierre Fontanillas, Ross M. Fraser, Daniel F. Freitag, Deepti Gurdasani, Kauko Heikkilä, Elina Hyppönen, Aaron Isaacs, Anne U. Jackson, Åsa Johansson, Toby Johnson, Marika Kaakinen, Johannes Kettunen, Marcus E. Kleber, Xiaohui Li, Jian'an Luan, Leo-Pekka Lyytikäinen, Patrik K.E. Magnusson, Massimo Mangino, Evelin Mihailov, May E. Montasser, Martina Müller-Nurasyid, Ilja M. Nolte, Jeffrey R. O'Connell, Cameron D. Palmer, Markus Perola, Ann-Kristin Petersen, Serena Sanna, Richa Saxena, Susan K. Service, Sonia Shah, Dmitry Shungin, Carlo Sidore, Ci Song, Rona J. Strawbridge, Ida Surakka, Toshiko Tanaka, Tanya M. Teslovich, Gudmar Thorleifsson, Evita G. Van den Herik, Benjamin F. Voight, Kelly A. Volcik, Lindsay L. Waite, Andrew Wong, Ying Wu, Weihua Zhang, Devin Absher,

Gershim Asiki, Inês Barroso, Latonya F. Been, Jennifer L. Bolton, Lori L Bonnycastle, Paolo Brambilla, Mary S. Burnett, Giancarlo Cesana, Maria Dimitriou, Alex S.F. Doney, Angela Döring, Paul Elliott, Stephen E. Epstein, Gudmundur Ingi Eyjolfsson, Bruna Gigante, Mark O. Goodarzi, Harald Grallert, Martha L. Gravito, Christopher J. Groves, Göran Hallmans, Anna-Liisa Hartikainen, Caroline Hayward, Dena Hernandez, Andrew A. Hicks, Hilma Holm, Yi-Jen Hung, Thomas Illig, Michelle R. Jones, Pontiano Kaleebu, John J.P. Kastelein, Kay-Tee Khaw, Eric Kim, Norman Klopp, Pirjo Komulainen, Meena Kumari, Claudia Langenberg, Terho Lehtimäki, Shih-Yi Lin, Jaana Lindström, Ruth J.F. Loos, François Mach, Wendy L McArdle, Christa Meisinger, Braxton D. Mitchell, Gabrielle Müller, Ramaiah Nagaraja, Narisu Narisu, Tuomo V.M. Nieminen, Rebecca N. Nsubuga, Isleifur Olafsson, Ken K. Ong, Aarno Palotie, Theodore Papamarkou, Cristina Pomilla, Anneli Pouta, Daniel J. Rader, Muredach P. Reilly, Paul M. Ridker, Fernando Rivadeneira, Igor Rudan, Aimo Ruokonen, Nilesh Samani, Hubert Scharnagl, Janet Seeley, Kaisa Silander, Alena Stančáková, Kathleen Stirrups, Amy J. Swift, Laurence Tiret, Andre G. Uitterlinden, L. Joost van Pelt, Sailaja Vedantam, Nicholas Wainwright, Cisca Wijmenga, Sarah H. Wild, Gonneke Willemsen, Tom Wilsgaard, James F. Wilson, Elizabeth H. Young, Jing Hua Zhao, Linda S. Adair, Dominique Arveiler, Themistocles L. Assimes, Stefania Bandinelli, Franklyn Bennett, Murielle Bochud, Bernhard O. Boehm, Dorret I. Boomsma, Ingrid B. Borecki, Stefan R. Bornstein, Pascal Bovet, Michel Burnier, Harry Campbell, Aravinda Chakravarti, John C. Chambers, Yii-Der Ida Chen, Francis S. Collins, Richard S. Cooper, John Danesh, George Dedoussis, Ulf de Faire, Alan B. Feranil, Jean Ferrières, Luigi Ferrucci, Nelson B. Freimer, Christian Gieger, Leif C. Groop, Vilmundur Gudnason, Ulf Gyllensten, Anders Hamsten, Tamara B. Harris, Aroon Hingorani, Joel N. Hirschhorn, Albert Hofman, G. Kees Hovingh, Chao Agnes Hsiung, Steve E. Humphries, Steven C. Hunt, Kristian Hveem, Carlos Iribarren, Marjo-Riitta Järvelin, Antti Jula, Mika Kähönen, Jaakko Kaprio, Antero Kesäniemi, Mika Kivimaki, Jaspal S. Kooner, Peter J. Koudstaal, Ronald M. Krauss, Diana Kuh, Johanna Kuusisto, Kirsten O. Kyvik, Markku Laakso, Timo A. Lakka, Lars Lind, Cecilia M. Lindgren, Nicholas G. Martin, Winfried März, Mark I. McCarthy, Colin A. McKenzie, Pierre Meneton, Andres Metspalu, Leena Moilanen, Andrew D. Morris, Patricia B. Munroe, Inger Njølstad, Nancy L. Pedersen, Chris Power, Peter P. Pramstaller, Jackie F. Price, Bruce M. Psaty, Thomas Quertermous, Rainer Rauramaa, Danish Saleheen, Veikko Salomaa, Dharambir K. Sanghera, Jouko Saramies, Peter E.H. Schwarz, Wayne H-H Sheu, Alan R. Shuldiner, Agneta Siegbahn, Tim D. Spector, Kari Stefansson, David P. Strachan, Bamidele O. Tayo, Elena Tremoli, Jaakko Tuomilehto, Matti Uusitupa, Cornelia M. van Duijn, Peter Vollenweider, Lars Wallentin, Nicholas J. Wareham, John B. Whitfield, Bruce H.R. Wolffenbuttel, Jose M. Ordovas, Eric Boerwinkle, Colin N.A. Palmer, Unnur Thorsteinsdottir, Daniel I. Chasman, Jerome I. Rotter, Paul W. Franks, Samuli Ripatti, L. Adrienne Cupples, Manjinder S. Sandhu, Stephen S. Rich, Michael Boehnke, Panos Deloukas, Sekar Kathiresan, Karen L. Mohlke, Erik Ingelsson, Gonçalo R. Abecasis

MAGIC

Scott, R.A. et al. Large-scale association analyses identify new loci influencing glycemic traits and provide insight into the underlying biological pathways. Nat Genet 44, 991-1005 (2012).

Robert A. Scott, Vasiliki Lagou, Ryan P. Welch, Eleanor Wheeler, May E. Montasser, Jian'an Luan, Reedik Mägi, Rona J. Strawbridge, Emil Rehnberg, Stefan Gustafsson, Stavroula Kanoni, Laura J. Rasmussen-Torvik, Loïc Yengo, Cecile Lecoeur, Dmitry Shungin, Serena Sanna, Carlo Sidore, Paul C. D. Johnson, J. Wouter Jukema, Toby Johnson, Anubha Mahajan, Niek Verweij, Gudmar Thorleifsson, Jouke-Jan Hottenga, Sonia Shah, Albert V. Smith, Bengt Sennblad, Christian Gieger, Perttu Salo, Markus Perola, Nicholas J. Timpson, David M. Evans, Beate St. Pourcain, Ying Wu, Jeanette S. Andrews, Jennie Hui, Lawrence F. Bielak, Wei Zhao, Momoko Horikoshi, Pau Navarro, Aaron Isaacs, Jeffrey R. O'Connell, Kathleen Stirrups, Veronique Vitart,

Caroline Hayward, Tõnu Esko, Evelin Mihailov, Ross M. Fraser, Tove Fall, Benjamin F. Voight, Soumya Raychaudhuri, Han Chen, Cecilia M. Lindgren, Andrew P. Morris, Nigel W. Rayner, Neil Robertson, Denis Rybin, Ching-Ti Liu, Jacques S. Beckmann, Sara M. Willems, Peter S. Chines, Anne U. Jackson, Hyun Min Kang, Heather M. Stringham, Kijoung Song, Toshiko Tanaka, John F. Peden, Anuj Goel, Andrew A. Hicks, Ping An, Martina Müller-Nurasyid, Anders Franco-Cereceda, Lasse Folkersen, Letizia Marullo, Hanneke Jansen, Albertine J. Oldehinkel, Marcel Bruinenberg, James S. Pankow, Kari E. North, Nita G. Forouhi, Ruth J. F. Loos, Sarah Edkins, Tibor V. Varga, Göran Hallmans, Heikki Oksa, Mulas Antonella, Ramaiah Nagaraja, Stella Trompet, Ian Ford, Stephan J. L. Bakker, Augustine Kong, Meena Kumari, Bruna Gigante, Christian Herder, Patricia B. Munroe, Mark Caulfield, Jula Antti, Massimo Mangino, Kerrin Small, Iva Miljkovic, Yongmei Liu, Mustafa Atalay, Wieland Kiess, Alan L. James, Fernando Rivadeneira, Andre G. Uitterlinden, Colin N. A. Palmer, Alex S. F. Doney, Gonneke Willemsen, Johannes H. Smit, Susan Campbell, Ozren Polasek, Lori L. Bonnycastle, Serge Hercberg, Maria Dimitriou, Jennifer L. Bolton, Gerard R. Fowkes, Peter Kovacs, Jaana Lindström, Tatijana Zemunik, Stefania Bandinelli, Sarah H. Wild, Hanneke V. Basart, Wolfgang Rathmann, Harald Grallert, DIAGRAM consortium, Winfried Maerz, Marcus E. Kleber, Bernhard O. Boehm, Annette Peters, Peter P. Pramstaller, Michael A. Province, Ingrid B. Borecki, Nicholas D. Hastie, Igor Rudan, Harry Campbell, Hugh Watkins, Martin Farrall, Michael Stumvoll, Luigi Ferrucci, Dawn M. Waterworth, Richard N. Bergman, Francis S. Collins, Jaakko Tuomilehto, Richard M. Watanabe, Eco J. C. de Geus, Brenda W. Penninx, Albert Hofman, Ben A. Oostra, Bruce M. Psaty, Peter Vollenweider, James F. Wilson, Alan F. Wright, G. Kees Hovingh, Andres Metspalu, Matti Uusitupa, Patrik K. E. Magnusson, Kirsten O. Kyvik, Jaakko Kaprio, Jackie F. Price, George V. Dedoussis, Panos Deloukas, Pierre Meneton, Lars Lind, Michael Boehnke, Alan R. Shuldiner, Cornelia M. van Duijn, Andrew D. Morris, Anke Toenjes, Patricia A. Peyser, John P. Beilby, Antje Körner, Johanna Kuusisto, Markku Laakso, Stefan R. Bornstein, Peter E. H. Schwarz, Timo A. Lakka, Rainer Rauramaa, Linda S. Adair, George Davey Smith, Tim D. Spector, Thomas Illig, Ulf de Faire, Anders Hamsten, Vilmundur Gudnason, Mika Kivimaki, Aroon Hingorani, Sirkka M. Keinanen-Kiukaanniemi, Timo E. Saaristo, Dorret I. Boomsma, Kari Stefansson, Pim van der Harst, Josée Dupuis, Nancy L. Pedersen, Naveed Sattar, Tamara B. Harris, Francesco Cucca, Samuli Ripatti, Veikko Salomaa, Karen L. Mohlke, Beverley Balkau, Philippe Froguel, Anneli Pouta, Marjo-Riitta Jarvelin, Nicholas J. Wareham, Nabila Bouatia-Naji, Mark I. McCarthy, Paul W. Franks, James B. Meigs, Tanya M. Teslovich, Jose C. Florez, Claudia Langenberg, Erik Ingelsson, Inga Prokopenko, Inês Barroso

The ReproGen Consortium

Perry, J.R. et al. Parent-of-origin-specific allelic associations among 106 genomic loci for age at menarche. Nature 514, 92-7 (2014).

Perry JR, Day F, Elks CE, Sulem P, Thompson DJ, Ferreira T, He C, Chasman DI, Esko T, Thorleifsson G, Albrecht E, Ang WQ, Corre T, Cousminer DL, Feenstra B, Franceschini N, Ganna A, Johnson AD, Kjellqvist S, Lunetta KL, McMahon G, Nolte IM, Paternoster L, Porcu E, Smith AV, Stolk L, Teumer A, Tšernikova N, Tikkanen E, Ulivi S, Wagner EK, Amin N, Bierut LJ, Byrne EM, Hottenga JJ, Koller DL, Mangino M, Pers TH, Yerges-Armstrong LM, Hua Zhao J, Andrulis IL, Anton-Culver H, Atsma F, Bandinelli S, Beckmann MW, Benitez J, Blomqvist C, Bojesen SE, Bolla MK, Bonanni B, Brauch H, Brenner H, Buring JE, Chang-Claude J, Chanock S, Chen J, Chenevix-Trench G, Collée JM, Couch FJ, Couper D, Coviello AD, Cox A, Czene K, D'adamo AP, Davey Smith G, De Vivo I, Demerath EW, Dennis J, Devilee P, Dieffenbach AK, Dunning AM, Eiriksdottir G, Eriksson JG, Fasching PA, Ferrucci L, Flesch-Janys D, Flyger H, Foroud T, Franke L, Garcia ME, García-Closas M, Geller F, de Geus EE, Giles GG, Gudbjartsson DF, Gudnason V, Guénel P, Guo S, Hall P, Hamann U, Haring R, Hartman CA,

Heath AC, Hofman A, Hooning MJ, Hopper JL, Hu FB, Hunter DJ, Karasik D, Kiel DP, Knight JA, Kosma VM, Kutalik Z, Lai S, Lambrechts D, Lindblom A, Mägi R, Magnusson PK, Mannermaa A, Martin NG, Masson G, McArdle PF, McArdle WL, Melbye M, Michailidou K, Mihailov E, Milani L, Milne RL, Nevanlinna H, Neven P, Nohr EA, Oldehinkel AJ, Oostra BA, Palotie A, Peacock M, Pedersen NL, Peterlongo P, Peto J, Pharoah PD, Postma DS, Pouta A, Pylkäs K, Radice P, Ring S, Rivadeneira F, Robino A, Rose LM, Rudolph A, Salomaa V, Sanna S, Schlessinger D, Schmidt MK, Southey MC, Sovio U, Stampfer MJ, Stöckl D, Storniolo AM, Timpson NJ, Tyrer J, Visser JA, Vollenweider P, Völzke H, Waeber G, Waldenberger M, Wallaschofski H, Wang Q, Willemsen G, Winqvist R, Wolffenbuttel BH, Wright MJ; Australian Ovarian Cancer Study; GENICA Network; kConFab; LifeLines Cohort Study; InterAct Consortium; Early Growth Genetics (EGG) Consortium, Boomsma DI, Econs MJ, Khaw KT, Loos RJ, McCarthy MI, Montgomery GW, Rice JP, Streeten EA, Thorsteinsdottir U, van Duijn CM, Alizadeh BZ, Bergmann S, Boerwinkle E, Boyd HA, Crisponi L, Gasparini P, Gieger C, Harris TB, Ingelsson E, Järvelin MR, Kraft P, Lawlor D, Metspalu A, Pennell CE, Ridker PM, Snieder H, Sørensen TI, Spector TD, Strachan DP, Uitterlinden AG, Wareham NJ, Widen E, Zygmunt M, Murray A, Easton DF, Stefansson K, Murabito JM, Ong KK.

The VAT/SAT Consortium

Fox, C.S. et al. Genome-wide association for abdominal subcutaneous and visceral adipose reveals a novel locus for visceral fat in women. PLoS Genet 8, e1002695 (2012).

Fox CS, Liu Y, White CC, Feitosa M, Smith AV, Heard-Costa N, Lohman K; GIANT Consortium; MAGIC Consortium; GLGC Consortium, Johnson AD, Foster MC, Greenawalt DM, Griffin P, Ding J, Newman AB, Tylavsky F, Miljkovic I, Kritchevsky SB, Launer L, Garcia M, Eiriksdottir G, Carr JJ, Gudnason V, Harris TB, Cupples LA, Borecki IB.

DETAILED AUTHORSHIP CONTRIBUTIONS

Steering Committee Overseeing the Consortium

Ruth J.F. Loos

Writing Group

Yingchang Lu, Felix R. Day, Stefan Gustafsson, Martin L. Buchkovich, Jianbo Na, Karen L Mohlke, M Carola Zillikens, John Andrew Pospisilik, Cecilia Lindgren, Tuomas Oskari Kilpeläinen, Ruth J.F. Loos

Data Cleaning and Preparation

Felix R. Day, Stefan Gustafsson, Ruth J.F. Loos, Yingchang Lu, Ryan W Walker

GWAS and Metabochip Meta-analyses

Felix R. Day, Stefan Gustafsson, Yingchang Lu

GWAS Look-ups in Other Consortia

(ADIPOGen Consortium) Zari Dastani, Rui Li, John Brent Richards, ADIPOGen Consortium; (CARDIOGRAMplusC D) Stavroula Kanoni, Panos Deloukas, CARDIOGRAMplusC D; (CHARGE: CRP meta-analysis) Abbas Dehghan, Chen Lu, CHARGE Inflammation working group; (CHARGE: IGF GWAS meta-analysis) Alexander Teumer, Qibin Qi, Robert C. Kaplan; (CHARGE: testosterone meta-analysis) Robin Haring, Claes Ohlson, CHARGE sex hormone working group; (Cutaneous nevi and melanoma risk) Mingfeng Zhang, Xin Li, David Hunter, Jiali Han; (Cutaneous nevi and melanoma risk) Pirro G Hysi, Veronique Bataille, Tim David Spector, Mario Falchi, Marianna Sanna; (Cutaneous nevi and melanoma risk) Mark M Iles, D Timothy Bishop, Julia A Newton Bishop, GenoMEL Consortium; (DIAGRAM consortium) Teresa Ferreira, Andrew P Morris, DIAGRAM consortium; (Early Growth Genetics Consortium) Diana L Cousminer, Elisabeth Widén, Early Growth Genetics Consortium; (GIANT Consortium) Adam Locke, Tõnu Esko, Dmitry Shungin, GIANT Consortium; (GLOBAL LIPIDS) Ellen M Schmidt, Cristen J Willer, Global Lipids Genetics Consortium; (LEPGen Consortium) Tuomas O. Kilpeläinen; (Liver enzyme GWAS meta-analysis) Weihua Zhang, William R Scott, John C Chambers, Jaspal S Kooner; (MAGIC) Inga Prokopenko, Reedik Mägi, Vasiliki Lagou, MAGIC Consortium; (ReproGen Consortium) John R.B. Perry, Joanne M. Murabito, Ken K. Ong, Reprogen Consortium; (VATGen consortium) Charles C. White, Douglass P Kiel, Adrienne Cupples, Caroline S Fox, VATGen consortium; (Tooth development in infancy meta-analysis) David M Evans, Ghazaleh Fatemifar.

Gene Expression (eQTL) Analyses

(Cortical brain study) Jinghua Zhao; (Blood-Eqtl meta-analysis) Tonu Esko, Andres Metspalu, Harm-Jan Westra, Lude Franke; (Bariatric Surgery Study) Eric E. Schadt; (MolOBB) Alexander W Drong, Fredrik Karpe, Åsa K Hedman, Mark I McCarthy.

Other Analyses and Contributions

(DEPICT) Tune H Pers, Juha Karjalainen, Lude Franke, Joel N Hirschhorn; (Drosophila knockdown and screening) Jianbo Na, John Andrew Pospisilik; (ENCODE) Martin L. Buchkovich, Terrence S Furey, Karen L Mohlke; (Functional Annotation) Ruth J.F. Loos, Yingchang Lu; (GCTA-COJO analysis) Zhihong Zhu, Anna AE Vinkhuyzen, Jian Yang; (GWAS Catalog Updates) Anne E Justice, Yingchang Lu, Kari E North; (IPA Pathway and Network Analysis) Yingchang Lu.

Project Design, Management and Coordination of Contributing Studies

(AGES); (Amish) Alan R Shudiner; (BASEII) Lars Bertram, Ilja Demuth, Rahel Eckardt, Elisabeth Steinhagen-Thiessen; (BLSA) Luigi Ferrucci; (B-PROOF) Lisette CPGM de Groot, Rosalie AM Dhonukshe-Rutten, Nathalie van der Velde, Natasja M van Schoor; (CHS); (CoLaus) Peter Vollenweider; (COROGENE CTRLS) Katja Borodulin, Markus Perola, Veikko Salomaa, Erkki Vartiainen; (CROATIA-Vis) Igor Rudan; (DESIR) Philippe Froguel; (DILGOM) Pekka Jousilahti, Aarno Palotie; (Ely/Fenland/EPIC (metabochip)) Nita G Forouhi, Claudia Langenberg, Ruth J.F. Loos, Ken K Ong, Robert A Scott, Nicholas J Wareham; (ERF) Ben A. Oostra, Cornelia M. van Duijn; (FamHS) Ingrid B Borecki; (Fels Longitudinal Study) Stefan A Czerwinski, Bradford Towne; (Fenland (GWAS)) Nicholas J Wareham; (FRAM) Douglass P Kiel, Caroline S Fox; (GxE); (GOOD) Claes Ohlsson; (H) Markku Heliövaara, Antti Jula, Paul B Knekt, Seppo Koskinen, Samuli Ripatti; (HABC); (HBCS) Johan G Eriksson, Tom Forsen, Eero Kajantie, Clive Osmond; (HEALTH) Torben Hansen, Torben Jørgensen, Allan Linneberg, Oluf Borbye Pedersen; (HERITAGE Family Study) Tuomo Rankinen, Claude Bouchard, DC Rao, **Treva K Rice**; (Johnston County Osteoarthritis Project) Joanne M. Jordan; (KoGES) Nam H Cho; (KORA F + KORA S /F (GWAS)) Angela Döring; (KORA F + KORA S /F (metabochip)) Christian Gieger, Annette Peters, Konstantin Strauch; (LEIPZIG_ADULT_IFB) Michael Stumvoll, Yvonne Boettcher; (LOLIPOP) John C Chambers, Jaspal S Kooner; (GOYA) Torben Hansen, Thorkild I A Sørensen; (MAP) David A Bennett; (METSIM); (MrOS) Eric S. Orwoll; (ORCADES) Harry Campbell, Nicholas Hastie, Sarah Wild, James F Wilson, Alan F Wright; (PIVUS) Erik Ingelsson, Lars Lind; (PREVEND) Ron T Gansevoort, Pim van der Harst; (QFS) Claude Bouchard, Louis Pérusse, Angelo Tremblay, Marie-Claude Vohl; (RISC); (RS) Albert Hofman, André G. Uitterlinden; (SHIP/SHIP-TREND) Henry Völzke, Henri Wallaschofski; (SOF) Steven R. Cummings, Gregory J. Tranah; (Sorbs) Michael Stumvoll; (SPT) Colin A McKenzie, Richard S Cooper, Joel N Hirschhorn; (TwinsUK) Tim D Spector; (ULSAM) Erik Ingelsson; (Whitehall II) Aroon Hingorani, Mika Kivimaki, Nicholas Wareham; (YFS) Mika Kähönen, Terho Lehtimäki, Olli T Raitakari, Jorma S Viikari.

Genotyping of Contributing Studies

(AGES); (Amish) Jeffrey R O'Connell; (BASEII) Lars Bertram, Tian Liu; (BLSA) Dena Hernandez; (B-PROOF); (CHS); (CoLaus); (COROGENE CTRLS); (CROATIA-Vis) Caroline Hayward, Nina Smolej Narančić, Veronique Vitart; (DESIR) Elodie Eury, Frédéric Fumeron, Stéphane Lobbens; (DILGOM) Peter J Wagner; (Ely/Fenland/EPIC (metabochip)) Claudia Langenberg, Ruth J.F. Loos, Ken K Ong, Nicholas J Wareham; (ERF); (FamHS) Ingrid B Borecki; (Fels Longitudinal Study) John Blangero, Joanne E Curran, Stefan A Czerwinski, Bradford Towne; (Fenland (GWAS)) Nicholas J Wareham; (FRAM) Douglass P Kiel, Caroline S

Fox; (GxE); (GOOD) Claes Ohlsson; (H); (HABC); (HBCS) Elisabeth Widén; (HEALTH) Niels Grarup; (HERITAGE Family Study) Tuomo Rankinen; (Johnston County Osteoarthritis Project) Joanne M. Jordan; (KoGES) Nam H Cho, Bok-Ghee Han, Jong-Young Lee; (KORA F + KORA S /F (GWAS)); (KORA F + KORA S /F (metabochip)); (LEIPZIG_ADULT_IFB) Michael Stumvoll, Yvonne Boettcher; (LOLIPOP) John C Chambers, Jaspal S Kooner; (GOYA); (MAP) Philip L De Jager; (METSIM); (MrOS) Carrie M. Nielson, Eric S. Orwoll, Joseph M Zmuda; (ORCADES) Harry Campbell, James F Wilson; (PIVUS); (PREVEND) Pim van der Harst; (QFS) Claire Bellis, John Blangero; (RISC) Laura Pascoe, Mark Walker; (RS) Carolina Medina-Gomez, Fernando Rivadeneira; (SHIP/SHIP-TREND) Nele Friedrich; (SOF) Gregory J. Tranah; (Sorbs) Peter Kovacs, Michael Stumvoll, Anke Tönjes; (SPT); (TwinsUK) Pirro G Hysi, Cristina Menni; (ULSAM); (Whitehall II) Meena Kumari, Claudia Langenberg; (YFS) Terho Lehtimäki.

Phenotype Coordination of Contributing Studies

(AGES); (Amish) Alan R Shudiner; (BASEII) Ilja Demuth, Rahel Eckardt, Dominik Spira, Elisabeth Steinhagen-Thiessen; (BLSA) Luigi Ferrucci; (B-PROOF) Anke W Enneman, Karin MA Swart; (CHS); (CoLaus); (COROGENE CTRLS) Katja Borodulin, Aki S Havulinna, Tiina Laatikainen, Veikko Salomaa; (CROATIA-Vis) Ivana Kolcic, Ozren Polašek; (DESIR); (DILGOM) Satu Männistö; (Ely/Fenland/EPIC (metabochip)) Nita G Forouhi, Nicholas J Wareham; (ERF) M Carola Zillikens; (FamHS) Ingrid B Borecki; (Fels Longitudinal Study) Audrey C Choh, Stefan A Czerwinski; (Fenland (GWAS)) Nicholas J Wareham; (FRAM) Douglass P Kiel, Caroline S Fox; (GxE); (GOOD) Claes Ohlsson, Liesbeth Vandenput; (H); (HABC); (HBCS) Johan G Eriksson, Jari Lahti; (HEALTH) Torben Jørgensen, Allan Linneberg; (HERITAGE Family Study) Tuomo Rankinen, Claude Bouchard; (Johnston County Osteoarthritis Project) Joanne M. Jordan; (KoGES) Nam H Cho, Hyung Jin Choi, Bok-Ghee Han, Jong-Young Lee, Chan Soo Shin; (KORA F + KORA S /F (metabochip)) Harald Grallert, Peter Lichtner; (LEIPZIG_ADULT_IFB) Anke Tönjes, Matthias Blüher, Yvonne Boettcher; (LOLIPOP) Uzma Afzal, John C Chambers, Jaspal S Kooner, Laticia Oozageer; (GOYA) Thorkild I A Sørensen; (MAP) Aron S Buchman; (METSIM) Johanna Kuusisto, Markku Laakso; (MrOS) Christine G. Lee, Eric S. Orwoll; (ORCADES) Sarah Wild, James F Wilson; (PIVUS) Karl Michaëlsson; (PREVEND) Stephan JL Bakker, Ron T Gansevoort, Hans Hillege; (QFS) Claude Bouchard, Angelo Tremblay; (RISC) Laura Pascoe, Mark Walker; (RS) Carolina Medina-Gomez, Fernando Rivadeneira, M Carola Zillikens; (SHIP/SHIP-TREND) Simone Gärtner, Till Ittermann; (SOF) Steven R. Cummings; (Sorbs) Matthias Blüher, Peter Kovacs, Anke Tönjes; (SPT) Terrence Forrester; (TwinsUK) Pirro G Hysi, Massimo Mangino; (ULSAM) Tommy Cederholm, Karl Michaëlsson; (Whitehall II) Meena Kumari, Mika Kivimaki; (YFS) Mika Kähönen, Olli T Raitakari, Leena Taittonen, Jorma S Viikari.

Data Analysis

(AGES); (Amish) Jeffrey R O'Connell; (BASEII) Lars Bertram, Tian Liu; (BLSA) Toshiko Tanaka; (B-PROOF) Anke W Enneman, Karin MA Swart; (CHS) Robert C Kaplan, Barbara McKnight; (CoLaus); (COROGENE CTRLS) Markus Perola; (CROATIA-Vis) Caroline Hayward, Veronique Vitart; (DESIR) Amélie Bonnefond, Loïc Yengo; (DILGOM) Niina Eklund, Kati Kristiansson; (Ely/Fenland/EPIC (metabochip)) Jian'an Luan; (ERF) Ayse Demirkan; (FamHS) Mary F Feitosa; (Fels Longitudinal Study) John Blangero, Audrey C Choh, Jack W Kent; (Fenland

(GWAS)) Jian'an Luan; (FRAM) Douglass P Kiel, Caroline S Fox; (GxE); (GOOD) Claes Ohlsson, Liesbeth Vandenput; (H) Emmi Tikkanen; (HABC); (HBCS) Jari Lahti; (HEALTH) Tarunveer Singh Ahluwalia; (HERITAGE Family Study) Yun Ju Sung, DC Rao, Tuomo Rankinen; (Johnston County Osteoarthritis Project) Youfang Liu; (KoGES) Nam H Cho, Hyung Jin Choi, Bok-Ghee Han, Jong-Young Lee, Chan Soo Shin; (KORA F + KORA S /F (GWAS)) Brigitte Kühnel, Janina S. Ried; (LEIPZIG_ADULT_IFB) Anubha Mahajan, Inga Prokopenko; (LOLIPOP) Marie Loh, Weihua Zhang; (GOYA) Tarunveer Singh Ahluwalia, Tuomas Oskari Kilpeläinen, Lavinia Paternoster; (MAP) Lei Yu; (METSIM) Michael Boehnke, Anne U Jackson, Teemu Kuulasmaa; (MrOS) Christine G. Lee, Carrie M. Nielson, Priya Srikanth; (ORCADES); (PIVUS) Stefan Gustafsson; (PREVEND) Irene Mateo Leach, Pim van der Harst, Niek Verweij; (QFS) John Blangero, Louis Pérusse; (RISC) Laura Pascoe, Mark Walker; (RS) Carolina Medina-Gomez, Fernando Rivadeneira; (SHIP/SHIP-TREND) Alexander Teumer; (SOF) Daniel S. Evans; (Sorbs) Reedik Mägi, Inga Prokopenko; (SPT) Cameron D Palmer; (TwinsUK) Massimo Mangino; (ULSAM) Stefan Gustafsson; (Whitehall II) Jian'An Luan; (YFS) Terho Lehtimäki, Leo-Pekka Lyytikäinen, Olli T Raitakari.
